# Synthesis of Sterically Congested Carbonyl Compounds via an *ipso*-Selective Sulfonium Rearrangement

**DOI:** 10.1021/jacs.5c13777

**Published:** 2025-10-08

**Authors:** Immo Klose, Christian Knittl-Frank, Nicolas G.-Simonian, Boris Maryasin, Daniel Kaiser, Nuno Maulide

**Affiliations:** † Institute of Organic Chemistry, 27258 University of Vienna, Währinger Straße 38, 1090 Vienna, Austria; ‡ Institute of Theoretical Chemistry, University of Vienna, Währinger Straße 17, 1090 Vienna, Austria; § Research Platform “NeGeMac”, University of Vienna, Währinger Straße 38, 1090 Vienna, Austria

## Abstract

Despite the success of metal-catalyzed cross-coupling strategies to assemble a variety of C–C bonds, the synthesis of sterically congested α-arylated carbonyl compounds remains a difficult task. Herein, we present an *ipso*-rearrangement approach relying on electron redistribution in sulfonium intermediates. This modular method enables the synthesis of a variety of α-arylated carbonyl compounds and is orthogonal to traditional metal-catalyzed cross-coupling strategies. More importantly, it demonstrated remarkable robustness toward steric hindrance and allowed us to efficiently forge congested C–C bonds.

## Graphical abstract

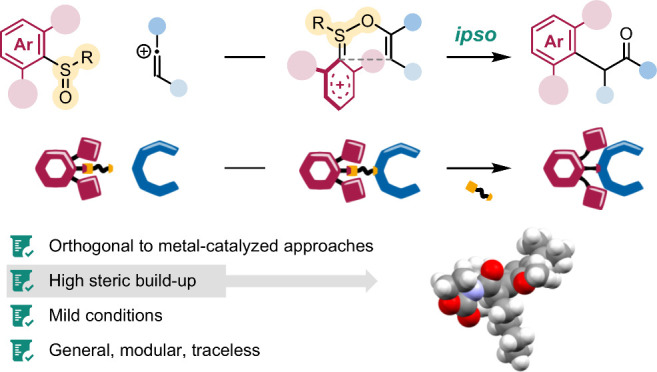

Carbonyl compounds bearing an aromatic substituent at the α-position are often of biological relevance, and developing methods for their efficient preparation has been a focal point of contemporary synthesis.^1^ The search for methods allowing for the formation of α-arylated carbonyl compounds naturally invoked metal-catalyzed approaches, perhaps the most notable of which are the Hartwig enolate coupling^2–7^ as well as polarity-reversed α-arylations (Figure 1A).^8–10^ Unfortunately, while transition metal-catalyzed cross-coupling has become a workhorse in organic synthesis, allowing for the assembly of a variety of C–C bonds, its classical oxidative addition/reductive elimination logic means that metal complexes—typically carrying already bulky ligands—face considerable challenges when attempting to couple precursors that are themselves sterically demanding.^2,11–15^

**Figure 1:**
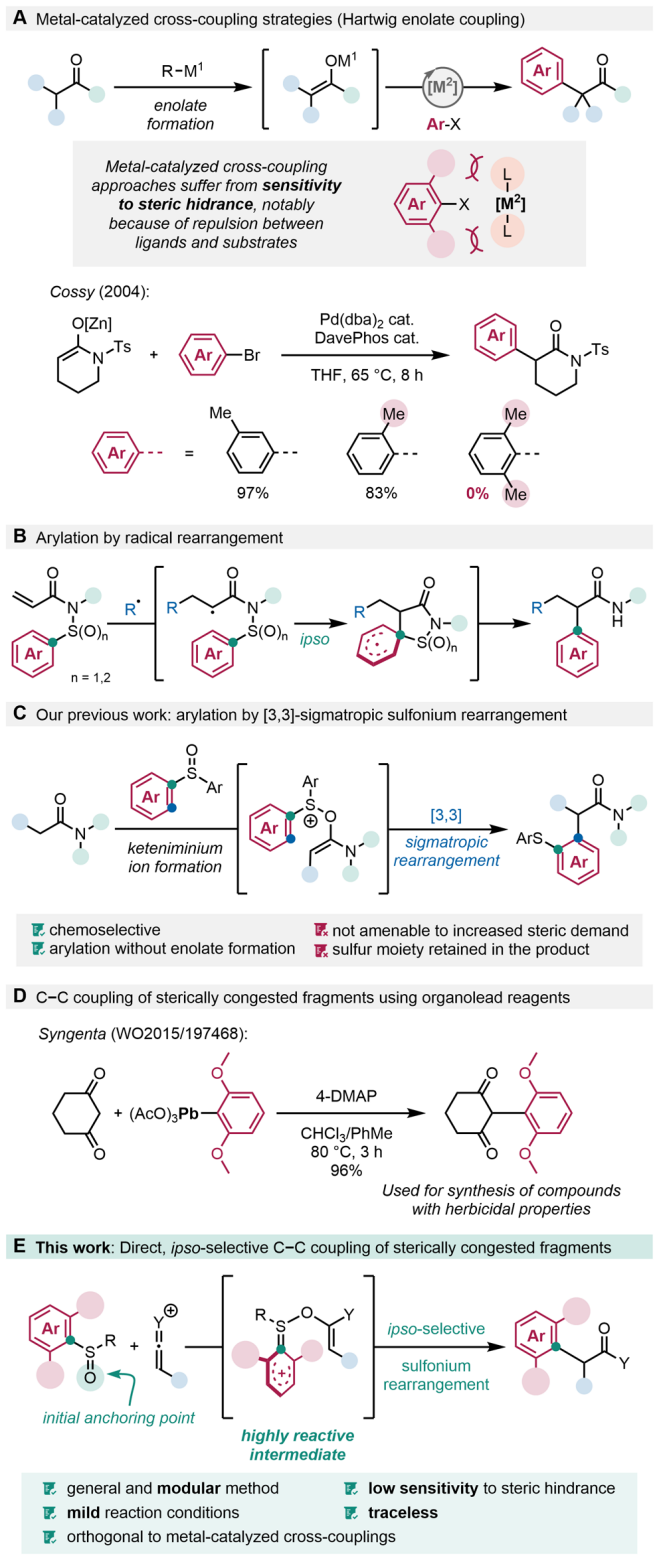
Challenges and solutions for the synthesis of α-arylated carbonyl compounds. (A) Metal-catalyzed approaches: the Hartwig enolate coupling; (B) Formation of α-aryl amides by radical rearrangement; (C) Our previous work on [3,3]-sigmatropic sulfonium rearrangements; (D) Use of organolead reagents for the assembly of congested C–C bonds; (E) This work: coupling of sterically hindered fragments by *ipso*-selective rearrangement.

On the other hand, the recent renaissance of radical chemistry and photoredox catalysis has shown that open-shell intermediates are less sensitive to the constraints of steric hindrance.^16–23^ However, control of the reactivity of these highly energetic species can be challenging. This is often addressed by using one reactant in a large excess,^24–26^ or by rendering the process intramolecular, which in turn reintroduces the problem of steric hindrance (Figure 1B).^27–32^

Throughout the past decade, Yorimitsu, Procter, ourselves and others have demonstrated the ability of sulfonium rearrangements to form highly congested C–C bonds between sp^3^-hybridized tertiary-quaternary centers or sp^2^–sp^3^-hybridized aryl-tertiary centers (Figure 1C).^33–40^ We thus became intrigued by the prospect of developing a sulfonium rearrangement of sp^2^-hybridized aryl sulfoxides capable of tolerating an increased steric demand in the context of a formal carbonyl arylation transformation.

Herein, we present a C–C coupling reaction for the formation of α-aryl carbonyl compounds through an unusual *ipso*-type rearrangement of a sulfonium intermediate arising from the reaction between an aryl sulfoxide and a vinyl cation (Figure 1E). The sulfoxide’s oxygen atom serves as the initial anchoring point for the two reactants, similar to a native chemical ligation, with this anchoring event giving rise to a highly reactive sulfonium intermediate, with a notable driving force toward rearrangement. The redirected process toward the *ipso*-position is related to [2,3]-sigmatropic rearrangements of sulfonium species which have been widely applied for C–heteroatom and C–C bonds formations.^41–58^ This is in contrast to transition metal-catalyzed cross-coupling reactions, as this assembly takes place one atom removed from the point of highest steric congestion, thereby facilitating proximity between the two reactive sites.

The transformation we describe herein is orthogonal to transition metal-catalyzed cross-coupling reactions and offers both traceless^59^ and modular access to previously challenging molecular architectures. It is noteworthy that, despite their seemingly simple structures and well-documented bioactivity,^60–63^ these compounds require arduous synthetic routes—often involving stoichiometric amounts of toxic heavy metals such as lead (Figure 1D).^64,65^

During our studies on the reactivity of vinyl cation intermediates with sulfoxides,^35,66^ we observed that *o,o’*-disubstituted aryl sulfoxides strayed from the established reactivity—which would direct C–C bond formation to the *ortho*-position through [3,3]-sigmatropic rearrangement.

Indeed, initial experiments yielded products of desulfitative coupling to the *ipso*-position in poor yield (Figure 2, top-right box).^67–70^ As an example, treating an ynamide such as **2A** with acid in the presence of sterically hindered aryl sulfoxide **1a**, we initially observed the formation of **4aA** only in traces. Optimization of this process (see SI for details) provided an increase in yield (96%, even on a gram scale). Arguably the key finding of the optimization process was the realization that the use of a thiol additive completely suppressed undesired side reactions and allowed the use of catalyst loadings as low as 2 mol % (see Figure 4 and Supporting Information (SI)). This screening of conditions also revealed that the reaction is very robust to changes in scale, temperature, solvent, and concentration. Additionally, this transformation can be performed in ethyl acetate (93% NMR yield), an important alternative solvent from the vantage point of green chemistry,^71^ and does not require aqueous workup.^72^

**Figure 2:**
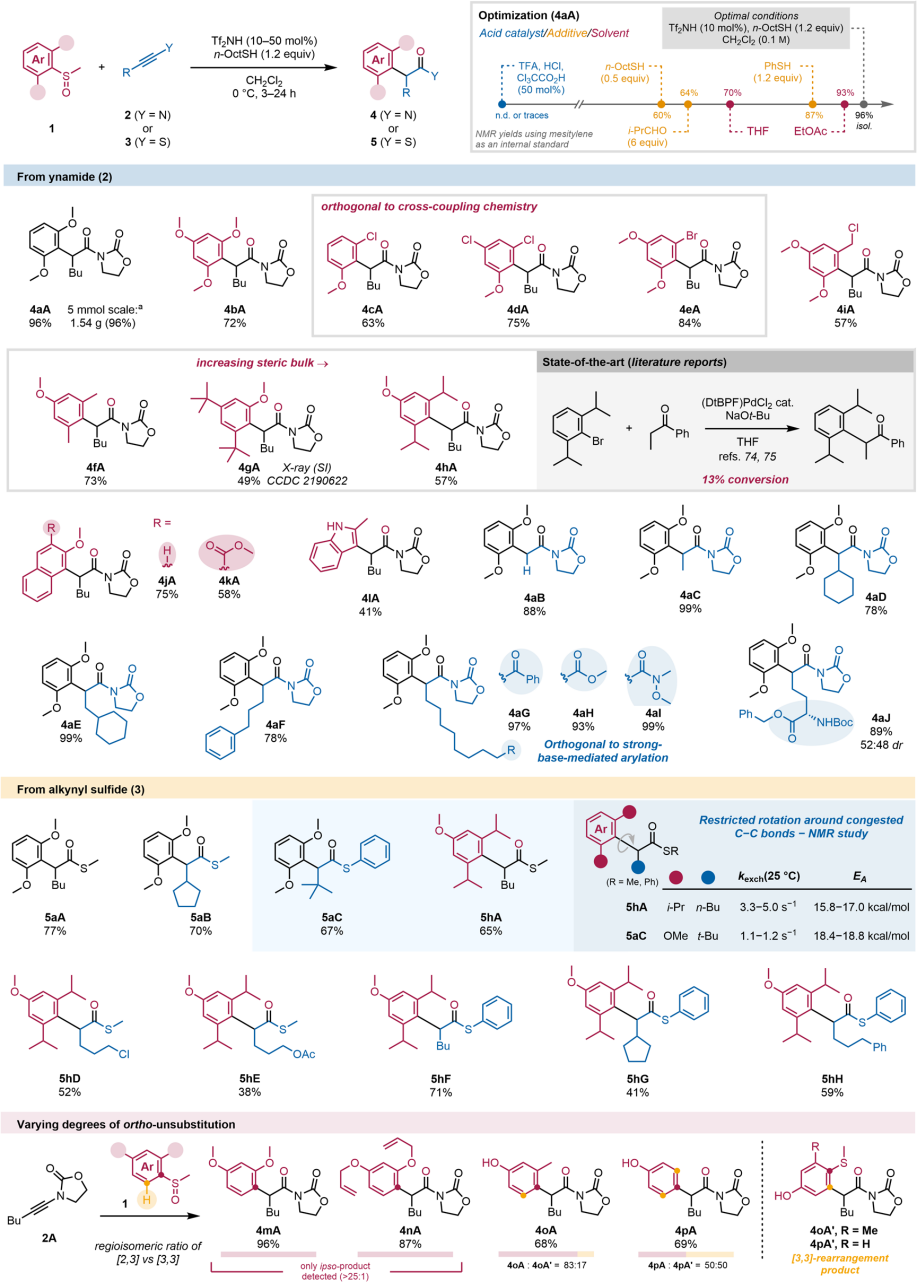
Optimization and substrate scope. Unless stated otherwise, yields refer to isolated material. Reactions were performed on a 0.05–0.25 mmol scale with Tf_2_NH (10–50 mol %) and *n*-OctSH (1.2 equiv) in CH_2_Cl_2_ (0.1 M) at 0 °C for 3–24 h. ^a^ Scale-up experiment: 5 mmol scale, ynamide (1.0 equiv), sulfoxide (1.0 equiv), *n*-OctSH (1.0 equiv), Tf_2_NH (2 mol %), 30 h, 96% yield. DtBPF = 1,1′-bis­(di-*tert*-butylphosphino)­ferrocene.

We first set out to study the scope of sulfoxides and were eager to explore how the method would fare in comparison with transition-metal-catalyzed processes. Pleasingly, we found that highly electron-rich aryl moieties, which are challenging substrates due to their impeded oxidative addition, work efficiently and afford the products in good yields (e.g., **4bA**).^73^ Moreover, a variety of halide-substituted sulfoxides were found to be tolerated by this protocol and delivered the corresponding α-arylated products **4cA–4eA** (63–84%), further showcasing the orthogonality of this method to conventional metal-catalyzed cross-couplings.

Furthermore, the sensitivity of the reaction toward steric hindrance was assessed by reacting sulfoxides bearing sterically demanding groups at both *ortho*-positions. Gratifyingly, *o,o’*-dimethylbenzene-substituted sulfoxide **1f** delivered α-arylated carbonyl product **4fA** in 73% yield. Remarkably, an aryl sulfoxide bearing two *tert*-butyl groups still afforded **4gA** in 49% yield (see SI for the X-ray structure of **4gA**). Even the highly sterically demanding *o,o’*-diisopropylbenzene-substituted sulfoxide **1h** yielded product **4hA** in 57% yield. This contrasts sharply with reported methods for preparing an *o*,*o*’-diisopropylphenyl-α-arylated carbonyl compound, using Pd-catalysis, which proceeds with poor conversion (13%).^74,75^

Functional-group tolerance and the effects of varying substitutions on the electrophilic reaction partner were then explored. Even excellent electrophiles such as benzylic chlorides are well tolerated (**4iA**, 57% yield), as well as bicyclic structures **4jA** and **4kA** (75% and 58% yield). An indole sulfoxide could also be coupled, affording product **4lA** in a 41% yield. Moreover, a wide range of ynamides (**2**) with varying substitution pattern could be coupled successfully, forming products **4aB–4aF** in high yields (78%–99%). The method also proved to be tolerant of other sensitive functional groups contained within the ynamide substrate, such as a ketone (**4aG**, 97%), an ester (**4aH**, 93%) or a Weinreb amide (**4aI**, 99%). Notably, the synthesis of such products, possessing multiple carbonyl groups, using enolate-based arylation (in the presence of strong bases) would be very challenging due to chemoselectivity issues. Strikingly, despite the acidic reaction conditions, an *N*-Boc-*O*-Bn protected amino acid was tolerated under the reaction conditions and provided the corresponding product **4aJ** in an excellent yield (89%, 52:48 *dr*). This result opens an avenue toward the construction of complex amino acid derivatives. Importantly, the successful reaction demonstrates that the threat of intramolecular interception of the highly reactive vinyl cation intermediate can be prevented by careful design of protecting groups.

We next turned our attention to another family of readily available vinyl cation precursors, alkynyl sulfides (**3**), and found that the reaction between **3A** and **1a** delivered α-arylated thioester **5aA** in 77% yield. Sterically more hindered cyclopentyl- and *tert*-butyl-substituted alkynyl sulfides **3B** and **3C** also smoothly underwent this *ipso*-selective coupling to furnish thioesters **5aB** (70%) and **5aC** (67%), respectively.

As this indicates that these reaction partners are also competent to form congested C–C bonds, we reacted our *o,o’*-diisopropylphenyl-substituted sulfoxide with different alkynyl sulfides, obtaining the corresponding thioesters **5hA**, **5hD**–**5hH** in yields ranging from 38% to 71%. Remarkably, thioester **5hG**, bearing two *o,o’*-isopropyl groups on the aryl and a secondary β-center, was obtained in a respectable 41% yield.

Such steric congestion could be diagnosed by NMR studies, which showed a relatively slow rotation around the newly formed C–C bond at room temperature. For the *o,o’*-diisopropylphenyl product **5hA**, the exchange constant (*k*_exch,_(25 °C)) was measured to be in the region of 4 s^–1^, whereas for α-*tert*-butyl product **5aC**, *k*_*exch*_ was measured to be roughly 1.1 s^–1^ (see SI).

To investigate whether steric or electronic factors drive the *ipso*-selective rearrangement, we tested several sulfoxides bearing a hydrogen atom in the *ortho*-position. To our surprise, sulfoxides **1m** and **1n** exclusively afforded products of *ipso*-coupling (**4mA** and **4nA**, 96% and 87% yield, respectively) with no observable amounts of the potentially competing products resulting from [3,3]-sigmatropic rearrangement onto the *ortho*-position (>25:1). This demonstrates that the well-developed *ortho*-functionalization of aryl sulfoxides can be completely rerouted toward the *ipso*-coupling by suitable electron distribution of the aryl moiety. However, upon decreasing electron density by replacement or omittance of oxygen substituents, the product of *ortho*-rearrangement reappeared, leading to mixtures of the two products. Thus, whereas **4oA** still showed a preference for *ipso*-rearrangement (83:17), an aryl sulfoxide bearing a sole *p*-hydroxy substituent resulted in a 1:1 mixture of [2,3]- and [3,3]-rearrangement products **4pA** and **4pA’**.

The generality of this coupling is highlighted by the fact that hindered aryl sulfoxides were readily *ipso*-coupled with a variety of donor-substituted alkynes without further optimization (Figure 3A–C).

**Figure 3:**
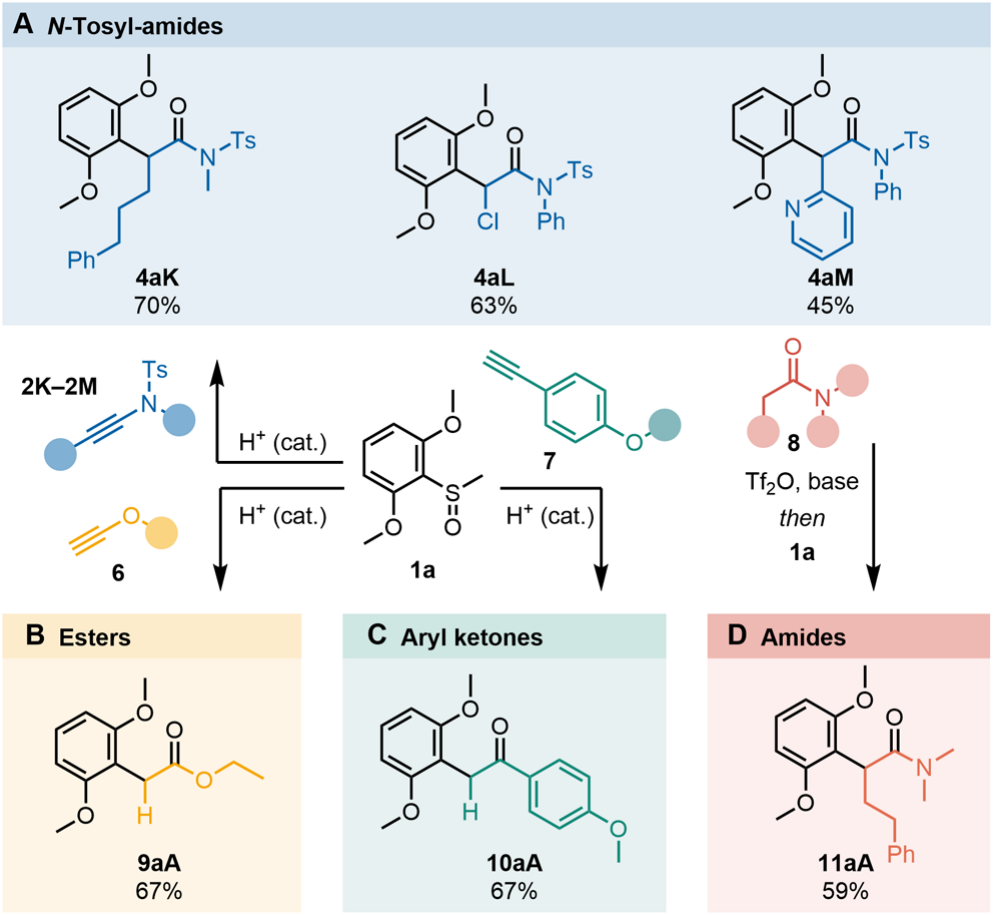
Generality of the *ipso*-rearrangement. Suitable precursors include (A) *N*-tosyl ynamides, (B) aryl alkynes, (C) ynol ethers (all through Brønsted acid catalyzed activation), and (D) amides via amide activation. Yields refer to isolated material. Reaction conditions: A: **4aK**, **4aL**, **4aM** (1.2 equiv), **1a** (1.0 equiv), Tf_2_NH (10, 30, 120 mol %, respectively), CH_2_Cl_2_, 0 °C, 24 h. B: **6** (1.2 equiv), **1a** (1.0 equiv), Tf_2_NH (10 mol %), CH_2_Cl_2_, 0 °C, 24 h. C: **7** (1.0 equiv), **1a** (1.1 equiv), TfOH (45 mol %), *n*-OctSH (1.1 equiv), MeNO_2_, 23 °C, 14 h. D: **8** (1.1 equiv), 2-fluoropyridine (2.2 equiv), Tf_2_O (1.1 equiv), CH_2_Cl_2_, 0 °C, 15 min, **1a** (1.0 equiv), *n*-OctSH (1.1 equiv), CH_2_Cl_2_, −78 °C, 30 min, 0 °C, 30 min.

As shown, sulfonamide-derived ynamides (**2**) afforded the corresponding α-aryl amides **4aK–4aL** (Figure 3A). The reaction was even amenable to a Brønsted-basic pyridine moiety, affording **4aM** in 45% yield when using 1.2 equiv of a Brønsted acid. Similarly, ynol ether **6** ^76^ and aryl alkyne **7** ^77,78^ readily afforded the *ipso*-coupling products **9aA** and **10aA**, respectively, each in 67% yield (Figure 3B,C).

Although the treatment of amides with an electrophilic activator leads to intermediates akin to those detected upon reaction of ynamides with an acid,^79,80^ diverging behavior is very often observed in their reactivity toward nucleophiles.^81,82^ We were pleased to find that amides such as **8** could nonetheless also be employed for this *ipso*-coupling process (Figure 3D), delivering α-branched aryl amide **11aA** in good yield.

Aware of the ability of enantioenriched substrates to transfer their stereogenic information through sigmatropic rearrangements,^35,36^ we investigated the potential stereoselectivity of this transformation using both a chiral ynamide (in combination with a racemic sulfoxide) and a chiral sulfoxide (in combination with an achiral ynamide). Consistent with previous findings,^66^ the use of a chiral ynamide did not lead to significant stereoselectivity, affording the product with a 1.5:1 *dr* (see SI, section 6.2, for details). Pleasingly, employing enantioenriched sulfoxides ((+)- or (−)-**1a**, both >99:1 *er*) in combination with **2D** or **3A**, the reaction proceeded with promising levels of chirality transfer, delivering (+)**-4aD** and (−)-**5aA** with 90:10 and 86:14 enantiomeric ratio, respectively (Figure 4A).

**Figure 4:**
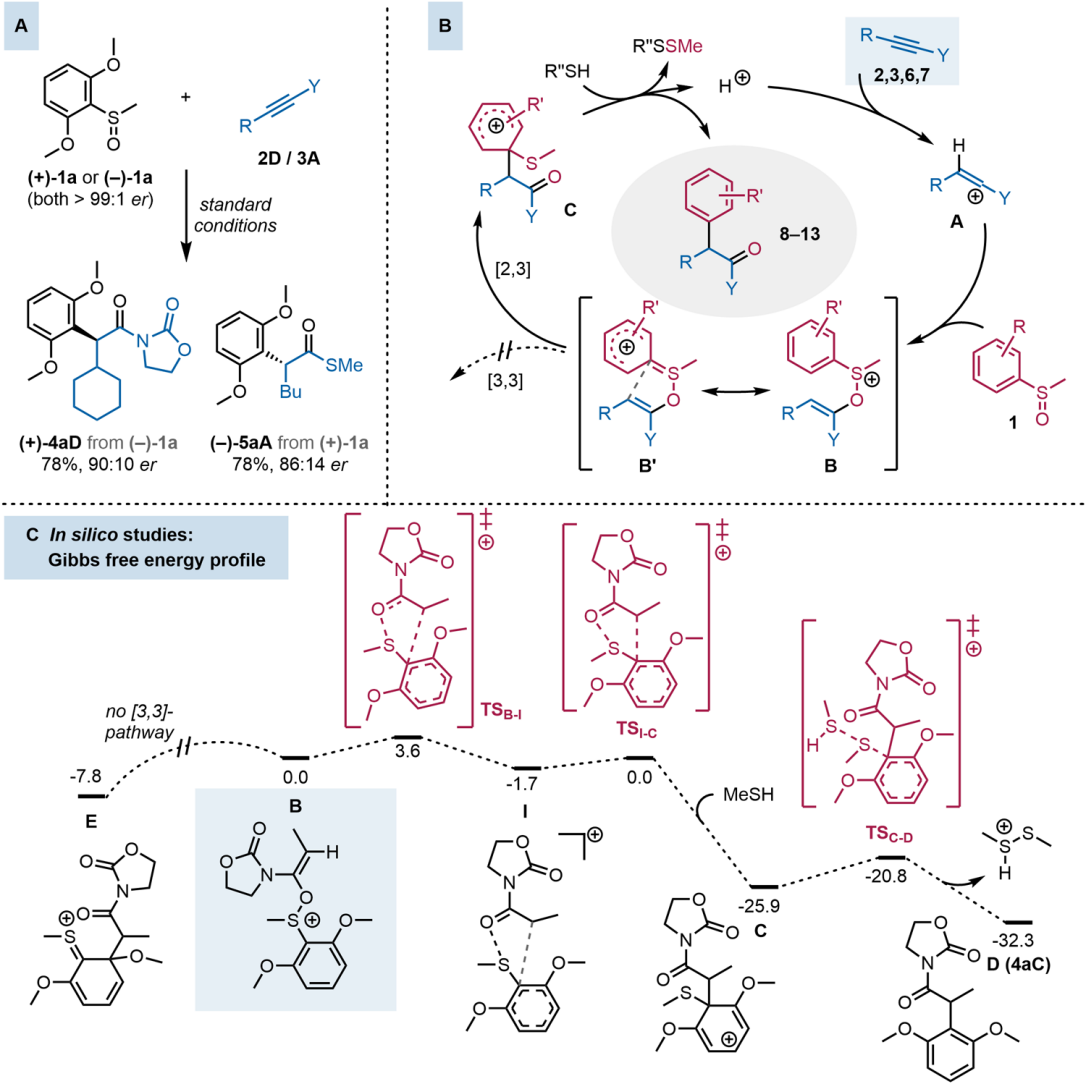
Exploration of an enantioselective variant and mechanistic proposal. (A) Examples of stereoselectivity in the *ipso*-selective sulfonium rearrangement; (B) Proposed mechanism of this highly selective arylation reaction, including the necessity for added sulfide; (C) Computational studies: the computed Gibbs free energy profile (RI-MP2-COSMO/def2-QZVP//RI-MP2/def2-SVP, Δ*G*_273,DCM_) for the conversion of the intermediate **B** (taken as a reference, 0.0 kcal mol^–1^) into the [2,3]-rearrangement intermediate **C** and the formal [3,3]-rearrangement intermediate **E**. Transition states are depicted in burgundy color.

From a mechanistic standpoint, we initially proposed that vinyl cation derivative **A**, formed by protonation of an electron-rich alkyne (**2**, **3**, **6** or **7**), reacts with a sulfoxide nucleophile (**1**) to form oxasulfonium adduct **B** (Figure 4B). Given the results of Figure 3E, it appears that redistribution of electron density and polarization of the aryl moiety (represented by the resonance structure **B′**) is what allows circumvention of [3,3]-rearrangement reactivity. The representation as **B′** highlights the ylidic nature of the C–S bond, foreshadowing the formation of **C** through a [2,3]-sigmatropic event.

To gain a deeper understanding of this rerouted sulfonium-rearrangement, computational studies were performed (RI-MP2-COSMO/def2-QZVP//RI-MP2/def2-SVP^83–87^ level of theory, see SI for the details). Importantly, considering our previous results,^88^ we found it necessary to go beyond the standard density functional theory (DFT) calculations to the more advanced and less system-dependent wave function-based method (RI-MP2).

Figure 4C shows the computed Gibbs free energy profile starting from intermediate **B** (akin to an *N*,*O*-ketene acetal), which is formed by the attack of a sulfoxide on the transient keteniminium intermediate (**A**, where Y = N) in the initial stages of the reaction.^35,89^ Interestingly, in contrast to our initial proposal, the rearrangement was found not to proceed by a concerted mechanism.^88^ Instead, cleavage of the S–O bond of intermediate **B** (via transition state **TS**_**B–I**_) first leads to the formation of transient intermediate **I**, an initially unforeseen species (cf. Figure 4B), where the recently formed fragments are still closely associated. Even though many sulfonium rearrangements are believed to proceed through an asynchronous, concerted rearrangement in which S–O cleavage slightly precedes C–C bond formation, several examples exist in which both processes are separated by an intermediate.^88,90–92^ The rearrangement described herein seems to belong to the same category, a feature likely responsible for the regio-diverted outcome of the reaction.^20–23,93,94^

Following the formation of intermediate **I**, a very low activation barrier (1.7 kcal mol^–1^) allows C–C bond formation, giving rise to intermediate **C** via **TS**_**I–C**_. Early transition state **TS**_**I–C**_ is characterized by the concurrent formation of a new C–C bond at the *ipso*-position and the breaking of weak interactions between the newly formed carbonyl and sulfide in transient intermediate **I**. Ultimately, *ipso*-substituted cationic intermediate **C** evolves into re-aromatized product **D** by S_N_2 attack of a thiol on the sulfide, with the simultaneous formation of a disulfide species (detected experimentally; see SI).

Furthermore, calculations strongly suggest that a direct [3,3]-sigmatropic rearrangement starting from this intermediate (**B**) is not possible, as no transition state leading to a potential intermediate **E** could be found (Figure 4C, see SI).

In conclusion, we have developed an acid-catalyzed strategy for the synthesis of α-arylated carbonyl compounds that allows buildup of sterically congested motifs. Our approach hinges on electron redistribution in an *ipso*-selective sulfonium rearrangement and is orthogonal to conventional transition metal-catalyzed cross-coupling logic. Computational studies revealed an unexpected intermediate in the proposed rearrangement process.

## Supplementary Material



## Notes

Supplementary experimental procedures, figures S1–S11, and tables S1–S10, computational details, temperature-dependent NMR study, NMR spectra, HPLC traces and supplementary references. The Supporting Information is available free of charge at https://pubs.acs.org/doi/10.1021/jacs.5c13777.

## Abbreviations

Tf: triflyl
cat: catalytic
equiv: equivalent

## References

[1] Johansson C. C. C., Colacot T. J. (2010). Metal-Catalyzed α-Arylation of Carbonyl and Related
Molecules: Novel Trends in C–C Bond Formation by C–H
Bond Functionalization. Angew. Chem., Int. Ed..

[2] Hamann B. C., Hartwig J. F. (1997). Palladium-Catalyzed Direct α-Arylation of Ketones.
Rate Acceleration by Sterically Hindered Chelating Ligands and Reductive
Elimination from a Transition Metal Enolate Complex. J. Am. Chem. Soc..

[3] Hamann B. C. H., Hartwig J. F. (1997). Palladium-Catalyzed
Direct α-Arylation of Ketones.
Rate Acceleration by Sterically Hindered Chelating Ligands and Reductive
Elimination from a Transition Metal Enolate Complex. J. Am. Chem. Soc..

[4] Shaughnessy K. H., Hamann B. C., Hartwig J. F. (1998). Palladium-Catalyzed
Inter- and Intramolecular
α-Arylation of Amides. Application of Intramolecular Amide Arylation
to the Synthesis of Oxindoles. J. Org. Chem..

[5] Culkin D. A., Hartwig J. F. (2003). Palladium-Catalyzed
α-Arylation of Carbonyl Compounds
and Nitriles. Acc. Chem. Res..

[6] Hao Y.-J., Hu X.-S., Zhou Y., Zhou J., Yu J.-S. (2020). Catalytic
Enantioselective α-Arylation of Carbonyl Enolates and Related
Compounds. ACS Catal..

[7] Ostrowska S., Scattolin T., Nolan S. P. (2021). N-Heterocyclic Carbene Complexes
Enabling the α-Arylation of Carbonyl Compounds. Chem. Commun..

[8] Lou S., Fu G. C. (2010). Nickel/Bis­(Oxazoline)-Catalyzed
Asymmetric Kumada Reactions of Alkyl
Electrophiles: Cross-Couplings of Racemic α-Bromoketones. J. Am. Chem. Soc..

[9] Lundin P. M., Fu G. C. (2010). Asymmetric Suzuki
Cross-Couplings of Activated Secondary Alkyl Electrophiles:
Arylations of Racemic α-Chloroamides. J. Am. Chem. Soc..

[10] Iwamoto T., Okuzono C., Adak L., Jin M., Nakamura M. (2019). Iron-Catalysed
Enantioselective Suzuki–Miyaura Coupling of Racemic Alkyl Bromides. Chem. Commun..

[11] Kuwajima I., Urabe H. (1982). Regioselective Arylation
of Silyl Enol Ethers of Methyl Ketones with
Aryl Bromides. J. Am. Chem. Soc..

[12] de
Filippis A., Gomez Pardo D., Cossy J. (2004). Palladium-Catalyzed
α-Arylation of *N*-Protected 2-Piperidinones. Tetrahedron.

[13] Su W., Raders S., Verkade J. G., Liao X., Hartwig J. F. (2006). Pd-Catalyzed
α-Arylation of Trimethylsilyl Enol Ethers with Aryl Bromides
and Chlorides: A Synergistic Effect of Two Metal Fluorides as Additives. Angew. Chem., Int. Ed..

[14] Escudero-Casao M., Licini G., Orlandi M. (2021). Enantioselective
α-Arylation
of Ketones via a Novel Cu­(I)–Bis­(Phosphine) Dioxide Catalytic
System. J. Am. Chem. Soc..

[15] Wu T., Castro A. J., Ganguli K., Rotella M. E., Ye N., Gallou F., Wu B., Weix D. J. (2025). Cross-Electrophile
Coupling to Form Sterically Hindered C­(sp^2^)–C­(sp^3^) Bonds: Ni and Co Afford Complementary Reactivity. J. Am. Chem. Soc..

[16] Jamison C. R., Overman L. E. (2016). Fragment Coupling
with Tertiary Radicals Generated
by Visible-Light Photocatalysis. Acc. Chem.
Res..

[17] Jeffrey J. L., Petronijević F.
R., MacMillan D. W. C. (2015). Selective
Radical–Radical Cross-Couplings: Design of a Formal β-Mannich
Reaction. J. Am. Chem. Soc..

[18] Kizu T., Uraguchi D., Ooi T. (2016). Independence
from the Sequence of
Single-Electron Transfer of Photoredox Process in Redox-Neutral Asymmetric
Bond-Forming Reaction. J. Org. Chem..

[19] Petronijević F. R., Nappi M., MacMillan D. W. C. (2013). Direct β-Functionalization
of Cyclic Ketones with Aryl Ketones via the Merger of Photoredox and
Organocatalysis. J. Am. Chem. Soc..

[20] Pirnot M.
T., Rankic D. A., Martin D. B. C., MacMillan D. W. C. (2013). Photoredox
Activation for the Direct β-Arylation of Ketones and Aldehydes. Science.

[21] Shaw M. H., Twilton J., MacMillan D. W. C. (2016). Photoredox
Catalysis in Organic Chemistry. J. Org. Chem..

[22] Uraguchi D., Kinoshita N., Kizu T., Ooi T. (2015). Synergistic Catalysis
of Ionic Bro̷nsted Acid and Photosensitizer for a Redox Neutral
Asymmetric α-Coupling of N-Arylaminomethanes with Aldimines. J. Am. Chem. Soc..

[23] Zhao H., Cuomo V. D., Tian W., Romano C., Procter D. J. (2025). Light-Assisted
Functionalization of Aryl Radicals towards Metal-Free Cross-Coupling. Nat. Rev. Chem..

[24] Li S., Wei J., Ren Q., Yue G., Qiu D., Fagnoni M., Protti S. (2023). Synthesis of α-Aryl
Carbonyls via Photoinduced
Formation of C–C Bonds. ChemCatChem..

[25] de
Souza A. A. N., Silva N. S., Müller A. V., Polo A. S., Brocksom T. J., de Oliveira K. T. (2018). Porphyrins
as Photoredox Catalysts in Csp^2^–H Arylations: Batch
and Continuous Flow Approaches. J. Org. Chem..

[26] Hossain M. M., Shaikh A. C., Moutet J., Gianetti T. L. (2022). Photocatalytic α-Arylation
of Cyclic Ketones. Nat. Synth..

[27] Whalley D. M., Greaney M. F. (2022). Recent Advances
in the Smiles Rearrangement: New Opportunities
for Arylation. Synthesis.

[28] G.-Simonian N., Guérinot A., Cossy J. (2023). SO_2_-Extrusive 1,4-(Het)­Aryl
Migration: Synthesis of α-Aryl Amides and Related Reactions. Synthesis.

[29] Hervieu C., Kirillova M. S., Suárez T., Müller M., Merino E., Nevado C. (2021). Asymmetric,
Visible Light-Mediated
Radical Sulfinyl-Smiles Rearrangement to Access All-Carbon Quaternary
Stereocentres. Nat. Chem..

[30] Gillaizeau-Simonian N., Barde E., Guérinot A., Cossy J. (2021). Cobalt-Catalyzed 1,4-Aryl
Migration/Desulfonylation Cascade: Synthesis of α-Aryl Amides. Chem. - Eur. J..

[31] Li Y., Hu B., Dong W., Xie X., Wan J., Zhang Z. (2016). Visible Light-Induced
Radical Rearrangement to Construct C–C Bonds via an Intramolecular
Aryl Migration/Desulfonylation Process. J. Org.
Chem..

[32] Wu X., Zhang X., Ji X., Deng G.-J., Huang H. (2023). Visible-Light-Induced
Cascade Arylazidation of Activated Alkenes with Trimethylsilyl Azide. Org. Lett..

[33] Kaiser D., Klose I., Oost R., Neuhaus J., Maulide N. (2019). Bond-Forming
and -Breaking Reactions at Sulfur­(IV): Sulfoxides, Sulfonium Salts,
Sulfur Ylides, and Sulfinate Salts. Chem. Rev..

[34] Peng B., Geerdink D., Farès C., Maulide N. (2014). Chemoselective Intermolecular
α-Arylation of Amides. Angew. Chem., Int.
Ed..

[35] Kaldre D., Maryasin B., Kaiser D., Gajsek O., González L., Maulide N. (2017). An Asymmetric Redox Arylation: Chirality Transfer from
Sulfur to Carbon through a Sulfonium [3,3]-Sigmatropic Rearrangement. Angew. Chem., Int. Ed..

[36] Kaldre D., Klose I., Maulide N. (2018). Stereodivergent
Synthesis of 1,4-Dicarbonyls
by Traceless Charge–Accelerated Sulfonium Rearrangement. Science.

[37] Gonçalves C. R., Klose I., Placidi S., Kaiser D., Maulide N. (2024). Sulfonium
Rearrangements Enable the Direct Preparation of Sulfenyl Imidinium
Salts. Angew. Chem., Int. Ed..

[38] Pulis A. P., Procter D. J. (2016). C–H Coupling
Reactions Directed by Sulfoxides:
Teaching an Old Functional Group New Tricks. Angew. Chem., Int. Ed..

[39] Hori M., Yanagi T., Murakami K., Nogi K., Yorimitsu H. (2019). Annulative
Synthesis of Benzofurans from General Alkenyl Sulfoxides and Phenols
via Pummerer/Sigmatropic Cascade. Bull. Chem.
Soc. Jpn..

[40] Murakami K., Imoto J., Matsubara H., Yoshida S., Yorimitsu H., Oshima K. (2013). Copper-Catalyzed Extended Pummerer Reactions of Ketene
Dithioacetal Monoxides with Alkynyl Sulfides and Ynamides with an
Accompanying Oxygen Rearrangement. Chem. - Eur.
J..

[41] Velado M., Fernández De La Pradilla R., Viso A. (2021). Diastereodivergent
Synthesis of 2-Ene-1,4-Hydroxy Sulfides from 2-Sulfinyl Dienes via
Tandem Sulfa-Michael/Sulfoxide-Sulfenate Rearrange-ment. Org. Lett..

[42] Miura M., Nakakita T., Toriyama M., Motohashi S. (2018). An Efficient
Synthesis of *exo*/*endo*-Hydroxylated
Cyclohexenones by Thiol/Amine-Mediated Tandem Aldol–[2,3]-Sigmatropic
Rearrangement: Amine-Dependent Complementary Regioselectivity. Asian. J. Org. Chem..

[43] Colomer I., Velado M., Fernández
De La Pradilla R., Viso A. (2017). From Allylic Sulfoxides to Allylic
Sulfenates: Fifty Years of a Never-Ending
[2,3]-Sigmatropic Rearrangement. Chem. Rev..

[44] Porte V., Nascimento V. R., Sirvent A., Tiefenbrunner I., Feng M., Kaiser D., Maulide N. (2024). Asymmetric Synthesis
of β-Ketoamides by Sulfonium Rearrangement. Angew. Chem., Int. Ed..

[45] Feng M., Tinelli R., Meyrelles R., González L., Maryasin B., Maulide N., Feng M., Tinelli R., Meyrelles R., Maryasin B., Maulide N., González L. (2023). Direct Synthesis
of α-Amino Acid Derivatives by Hydrative Amination of Alkynes. Angew. Chem., Int. Ed..

[46] Feng M., Mosiagin I., Kaiser D., Maryasin B., Maulide N. (2022). Deployment
of Sulfinimines in Charge-Accelerated Sulfonium Rearrangement Enables
a Surrogate Asymmetric Mannich Reaction. J.
Am. Chem. Soc..

[47] Feng M., Fernandes A. J., Sirvent A., Spinozzi E., Shaaban S., Maulide N. (2023). Free Amino
Group Transfer via α-Amination of
Native Carbonyls. Angew. Chem., Int. Ed..

[48] Feng M., Fernandes A. J., Meyrelles R., Maulide N. (2023). Direct Enantioselective
α-Amination of Amides Guided by DFT Prediction of *E*/*Z* Selectivity in a Sulfonium Intermediate. Chem.

[49] Li C. T., Liu H., Yao Y., Lu C. D. (2019). Rearrangement
of N-Tert-Butanesulfinyl
Enamines for Synthesis of Enantioenriched α-Hydroxy Ketone Derivatives. Org. Lett..

[50] Yisimayili N., Liu T., Xiong T. Z., Lu C. D. (2024). Stereodivergent Conjugate Reduction
of α-Substituted α,β-Unsaturated *N*-Sulfinyl Ketimines: Flexible Access to Challenging Acyclic β,β-Disubstituted
Enesulfinamides. Org. Chem. Front..

[51] Zhu C. L., Lu C. D. (2023). Stereoselective
Formal Alkenylation of β,β-Disubstituted
Enesulfinamides for Constructing 1,5- and 1,4-Dicarbonyl Derivatives
Bearing Less-Accessible Acyclic α-Quaternary Stereocenters. Org. Chem. Front..

[52] Zhang X., Li J., Rotella M. E., Zhang R., Kozlowski M. C., Jia T. (2025). Dearomative Mislow-Braverman-Evans
Rearrangement of Aryl Sulfoxides. JACS Au.

[53] Ma P.-J., Tang F., Yao Y., Lu C.-D. (2019). Addition–Rearrangement
of Ketenes with Lithium N-Tert-Butanesulfinamides: Enantioselective
Synthesis of α,α-Disubstituted α-Hydroxycarboxylic
Acid Derivatives. Org. Lett..

[54] Yisimayili N., Liu H., Yao Y., Lu C. D. (2021). Stereodivergent Construction of Vicinal
Acyclic Quaternary-Tertiary Carbon Stereocenters by Michael-Type Alkylation
of α,α-Disubstituted *N*-*tert*-Butanesulfinyl Ketimines. Org. Lett..

[55] Velado M., Martinović M., Alonso I., Tortosa M., Fernández
de la Pradilla R., Viso A. (2023). Base-Induced Sulfoxide-Sulfenate
Rearrangement of 2-Sulfinyl Dienes for the Regio- and Stereoselective
Synthesis of Enantioenriched Dienyl Diols. J.
Org. Chem..

[56] Liu T.-F., Yao Y., Lu C.-D. (2023). Enantioselective Formation of α-Amino Acid Derivatives
via [2,3]-Sigmatropic Rearrangement of *N*-Acyl Iminosulfinamides. Org. Lett..

[57] Liu T.-F., Yao Y., Lu C.-D. (2024). Enantioselective
Formal 1,2-Diamination of Ketenes
with Iminosulfinamides: Asymmetric Synthesis of Unnatural α,α-Disubstituted
α-Amino Acid Derivatives. Org. Lett..

[58] Tang F., Yao Y., Xu Y. J., Lu C. D. (2018). Diastereoselective Aza-Mislow–Evans
Rearrangement of N-Acyl Tert-Butanesulfinamides into α-Sulfenyloxy
Carboxamides. Angew. Chem., Int. Ed..

[59] Hu M., Liang Y., Ru L., Ye S., Zhang L., Huang X., Bao M., Kong L., Peng B. (2023). Defluorinative Multi-Functionalization of Fluoroaryl Sulfoxides Enabled
by Fluorine-Assisted Temporary Dearomatization. Angew. Chem., Int. Ed..

[60] Numata, A. ; Iwawaki, Y. ; Furukawa, Y. ; Yoshino, Y. ; Miyakado, Y. ; Furuhashi, T. ; Miyazaki, T. Ketone or Oxime Compound, and Herbicide. WO2016098899 (A1), June 23, 2016.

[61] Hennessy, A. ; Jones, E. ; Hachisu, S. ; Willetts, N. ; Dale, S. ; Gregory, A. ; Houlsby, I. ; Bhonoah, Y. ; Comas-Barcelo, J. Herbicidal 3-Azaspiro[5.5]­Undecane-8,10-Dione Compounds. WO2019158666 (A1), August 22, 2019.

[62] Fenkl, F. ; Helmke, H. ; Rembiak, A. ; Angermann, A. ; Lehr, S. ; Fischer, R. ; Bojack, G. ; Dietrich, H. ; Gatzweiler, E. ; Rosinger, C. Anellated 3-Phenyl Tetramic Acid Derivatives Having a Herbicidal Effect. WO2017178314 (A1), October 19, 2017.

[63] Hachisu, S. ; Scutt, J. N. ; Willetts, N. J. Herbicidally Active 2-Halogen-4-Alkynyl-Phenyl-Pyrazolidine-Dione or Pyrrolidine-Dione Derivatives. WO2015040114 (A1), March 26, 2015.

[64] Elliott G. I., Konopelski J. P. (2001). Arylation
with Organolead and Organobismuth Reagents. Tetrahedron.

[65] James J., Guiry P. J. (2017). Highly Enantioselective Construction
of Sterically
Hindered α-Allyl-α-Aryl Lactones via Palladium-Catalyzed
Decarboxylative Asymmetric Allylic Alkylation. ACS Catal..

[66] Peng B., Huang X., Xie L.-G., Maulide N. (2014). A Bro̷nsted
Acid
Catalyzed Redox Arylation. Angew. Chem., Int.
Ed..

[67] Akai S., Kakiguchi K., Nakamura Y., Kuriwaki I., Dohi T., Harada S., Kubo O., Morita N., Kita Y. (2007). Total Synthesis
of (±)-γ-Rubromycin on the Basis of Two Aromatic Pummerer-Type
Reactions. Angew. Chem., Int. Ed..

[68] Akai S., Takeda Y., Iio K., Yoshida Y., Kita Y. (1995). An Efficient
Synthesis of *p*-Quinones Utilizing a Novel Pummerer-Type
Rearrangement of *p*-Sulfinylphenols. J. Chem. Soc. Chem. Commun..

[69] Akai S., Iio K., Takeda Y., Ueno H., Yokogawa K., Kita Y. (1995). Pummerer-Type
Rearrangement on Aromatic Rings: An Unprecedented Ipso -Substitution
of the Sulfinyl Group of p -Sulfinylphenyl Ethers into Oxygen Functional
Groups Leading to Protected Dihydroquinone Derivatives. J. Chem. Soc. Chem. Commun..

[70] Akai S., Takeda Y., Iio K., Takahashi K., Fukuda N., Kita Y. (1997). Novel *ipso*-Substitution
of p-Sulfinylphenols through the Pummerer-Type Reaction: A Selective
and Efficient Synthesis of p-Quinones and Protected p-Dihydroquinones. J. Org. Chem..

[71] Ahluwalia, V. K. ; Kidwai, M. Basic Principles of Green Chemistry. In New Trends in Green Chemistry; Ahluwalia, V. K. , Kidwai, M. , Eds.; Springer Netherlands: Dordrecht, 2004; pp 5–14.

[72] Prat D., Hayler J., Wells A. (2014). A Survey of
Solvent Selection Guides. Green Chem..

[73] Fauvarque J.-F., Pflüger F., Troupel M. (1981). Kinetics of Oxidative Addition of
Zerovalent Palladium to Aromatic Iodides. J.
Organomet. Chem..

[74] Grasa G. A., Colacot T. J. (2008). A Highly Practical and General Route
for α-Arylations
of Ketones Using Bis-Phosphinoferrocene-Based Palladium Catalysts. Org. Process Res. Dev..

[75] Grasa G. A., Colacot T. J. (2007). α-Arylation
of Ketones Using Highly Active, Air-Stable
(DtBPF)­PdX2 (X = Cl, Br) Catalysts. Org. Lett..

[76] Hu L., Gui Q., Chen X., Tan Z., Zhu G. (2016). HOTf-Catalyzed, Solvent-Free
Oxyarylation of Ynol Ethers and Thioethers. J. Org. Chem..

[77] Kaiser D., Veiros L. F., Maulide N. (2017). Redox-Neutral
Arylations of Vinyl
Cation Intermediates. Adv. Synth. Catal..

[78] Kaiser D., Veiros L. F., Maulide N. (2016). Bro̷nsted Acid-Mediated Hydrative
Arylation of Unactivated Alkynes. Chem. - Eur.
J..

[79] Kaiser D., Maulide N. (2016). Making the Least Reactive Electrophile the First in
Class: Domino Electrophilic Activation of Amides. J. Org. Chem..

[80] Kaiser D., Bauer A., Lemmerer M., Maulide N. (2018). Amide Activation: An
Emerging Tool for Chemoselective Synthesis. Chem. Soc. Rev..

[81] Tona V., de la Torre A., Padmanaban M., Ruider S., González L., Maulide N. (2016). Chemo- and Stereoselective Transition-Metal-Free Amination
of Amides with Azides. J. Am. Chem. Soc..

[82] Tona V., Ruider S. A., Berger M., Shaaban S., Padmanaban M., Xie L.-G., González L., Maulide N. (2016). Divergent Ynamide Reactivity
in the Presence of Azides–an Experimental and Computational
Study. Chem. Sci..

[83] Weigend F., Häser M. (1997). RI-MP2: First
Derivatives and Global Consistency. Theor. Chem.
Acc..

[84] Hättig C., Hellweg A., Köhn A. (2006). Distributed Memory Parallel Implementation
of Energies and Gradients for Second-Order Mo̷ller–Plesset
Perturbation Theory with the Resolution-of-the-Identity Approximation. Phys. Chem. Chem. Phys..

[85] Weigend F., Häser M., Patzelt H., Ahlrichs R. (1998). RI-MP2: Optimized Auxiliary
Basis Sets and Demonstration of Efficiency. Chem. Phys. Lett..

[86] Weigend F., Ahlrichs R. (2005). Balanced Basis Sets of Split Valence,
Triple Zeta Valence
and Quadruple Zeta Valence Quality for H to Rn: Design and Assessment
of Accuracy. Phys. Chem. Chem. Phys..

[87] Weigend F. (2006). Accurate Coulomb-Fitting
Basis Sets for H to Rn. Phys. Chem. Chem. Phys..

[88] Maryasin B., Kaldre D., Galaverna R., Klose I., Ruider S., Drescher M., Kählig H., González L., Eberlin M. N., Jurberg I. D., Maulide N. (2018). Unusual Mechanisms
in Claisen Rearrangements: An Ionic Fragmentation Leading to a Meta-Selective
Rearrangement. Chem. Sci..

[89] G.-Simonian N., Spieß P., Riomet M., Maryasin B., Klose I., Beaton Garcia A., Pollesböck L., Kaldre D., Todorovic U., Liu J. M., Kaiser D., González L., Maulide N. (2024). Stereodivergent Synthesis of 1,4-Dicarbonyl Compounds
through Sulfonium Rearrangement: Mechanistic Investigation, Stereocontrolled
Access to γ-Lactones and γ-Lactams, and Total Synthesis
of Paraconic Acids. J. Am. Chem. Soc..

[90] Yanagi T., Yorimitsu H. (2021). Mechanistic Investigation of a Synthetic Route to Biaryls
by the Sigmatropic Rearrangement of Arylsulfonium Species. Chem. - Eur. J..

[91] Bisht R., Popescu M. V., He Z., Ibrahim A. M., Crisenza G. E. M., Paton R. S., Procter D. J. (2023). Metal-Free
Arylation of Benzothiophenes
at C4 by Activation as Their Benzothiophene S-Oxides. Angew. Chem., Int. Ed..

[92] Wakabayashi R., Wang S., Kurogi T., Yorimitsu H. (2024). Arylation
of Benzazoles at the 4 Positions by Activation of Their 2-Methylsulfinyl
Groups. Chem. Commun..

[93] Šiaučiulis M., Sapmaz S., Pulis A. P., Procter D. J. (2018). Dual Vicinal Functionalisation
of Heterocycles via an Interrupted Pummerer Coupling/[3,3]-Sigmatropic
Rearrangement Cascade. Chem. Sci..

[94] He Z., Shrives H. J., Fernández-Salas J. A., Abengózar A., Neufeld J., Yang K., Pulis A. P., Procter D. J. (2018). Synthesis
of C2 Substituted Benzothiophenes via an Interrupted Pummerer/[3,3]-Sigmatropic/1,2-Migration
Cascade of Benzothiophene S-Oxides. Angew. Chem.,
Int. Ed..

