# Supplementary material for: Synthesis of Sterically Congested Carbonyl Compounds via an ipso-Selective Sulfonium Rearrangement
Source: J Am Chem Soc. 2025 Oct 8;147(42):37899–906. doi: 10.1021/jacs.5c13777 (PMC12550846; doi:10.1021/jacs.5c13777)
Supplement: Supplementary file 1 [file ja5c13777_si_001.pdf]

Supporting Information for

Synthesis of sterically congested carbonyl compounds *via* an *ipso*-selective sulfonium rearrangement

Immo Klose,† Christian Knittl-Frank,† Nicolas G.-Simonian,† Boris Maryasin, Daniel Kaiser and Nuno Maulide*

† These authors contributed equally to this work; Correspondence to: nuno.maulide@univie.ac.at

1. Table of Contents

2. General information 3
3. Optimization 4
4. Synthesis of Sulfoxides 6
5. Synthesis of ynamides and alkynyl sulfides 21
6. *ipso*-Coupling 26
7. Temperature dependent NMR study 62
8. Computational Study 69
9. NMR Spectra 90
10. HPLC Traces 153
11. X-Ray Analysis 156
12. References 159

2. General information

General procedures. All reactions were performed in round bottom flasks or vials fitted with rubber septa with magnetic stirring, unless otherwise stated. Reaction vessels were flushed with argon prior to use, unless otherwise stated. Liquids and solutions were transferred via syringe. All reactions were performed using anhydrous solvents. Reaction progress was monitored by thin layer chromatography (TLC) performed on aluminum plates coated with silica gel F_{254} with 0.2 mm thickness. Chromatograms were visualized by fluorescence quenching with UV light at 254 nm or by staining using potassium permanganate followed by heating. Flash column chromatography was carried out on 230–400 mesh silica gel (Merck and co.) using reagent grade solvents. prepTLC purifications were performed on SIL G-200 UV_{254} 20cmx20cm 2mm thickness plates (Macherey-Nagel) using reagent grade solvents.

Materials. All commercial reagents and solvents were used without further purification. Racemic products were synthesized according to the same procedure from the racemic sulfoxides.

Instrumentation. ^{1}H NMR and ^{13}C NMR spectra were recorded using a Bruker AV-400, AV-500, AV-600 or AV-700 spectrometer at 300 K. Chemical shifts (δ) were given in parts per million (ppm), referenced to the solvent peak of $CDCl_3$, defined at δ = 7.26 ppm (^{1}H NMR) and δ = 77.16 ppm (^{13}C NMR). Coupling constants (J) are reported in Hertz (Hz). ^{1}H NMR splitting patterns are designated as singlet (s), doublet (d), triplet (t), quartet (q), septet (sept) as they appear in the spectrum. If the appearance of a signal differs from the expected splitting pattern, the observed pattern is designated as apparent (app). Splitting patterns that could not be interpreted or easily visualized are designated as multiplet (m) or broad (br). Infrared (IR) spectra were obtained using Perkin-Elmer Spectrum 100 FT-IR spectrometer. Wavenumbers ($\nu = \lambda^{-1}$) are reported in cm^{-1}. Mass spectra were obtained using a Bruker maXis UHR--TOF spectrometer (70 eV), using electrospray ionization (ESI) or atmospheric-pressure chemical ionization (APCI) or an Agilent 7200B GC/Q-TOF spectrometer (70 eV), using electron ionization (EI). Optical rotations were measured on a Perkin Elmer 341 polarimeter using a 100 mm path-length cell at 589 nm (c given in g / (100 ml)). Chiral HPLC was performed using an AGILENT Infinity 1260 with Lux-Cellulose1 or Lux-Cellulose3 columns. Details of chromatographic conditions are indicated under each compound.

3. Optimization

Table S 1 to **Table S 3** present selected experiments performed for optimization of reaction conditions. All optimization reactions were performed on a 0.05–0.1 mmol scale unless stated otherwise.

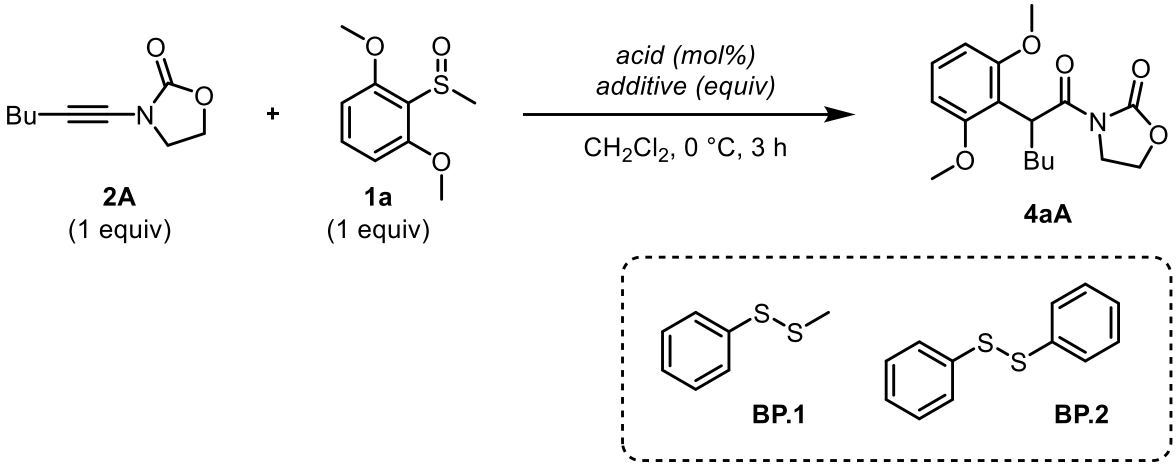

entry	acid (mol%)	additive (equiv)	yield[a]
**1**[c]	Tf_2NH (20)	PhSH (1)	86%(85%)[b]
**2**	Tf_2NH (10)	PhSH (1)	89%
**3**	Tf_2NH (5)	PhSH (1)	89%
**4**	Tf_2NH (2.5)	PhSH (1)	40%(45% conv.)
**5**	Tf_2NH (20)	PhSH (1.2)	87%
**6**	Tf_2NH (20)	OctSH (1.2)	91%
**7**	Tf_2NH (10)	OctSH (1.2)	95%
**8**	Tf_2NH (20)	OctSH (0.5)	60%
**9**	TFA (50)	OctSH (1.2)	n.d.[d]
**10**	Cl_3CO_2H (50)	OctSH (1.2)	n.d.[d]
**11**	HCl (2 M in Et_2O) (50)	OctSH (1.2)	n.d.[d]
**12**	Tf_2NH (20)	*i*-PrCHO (6)	64%

Table S 1: Preliminary tests on racemic version of the reaction: [a] Yields are determined by ^1H NMR analysis of the reaction crude using mesitylene as internal standard. [b] Isolated yield in parenthesis. [c] A mixture of **BP.1** and **BP.2** was also isolated. Formation of **BP.2** is likely the result of an acid-catalyzed disulfide exchange. [d] n.d. = not detected. Tf = trifluoromethanesulfonate. TFA = trifluoroacetic acid.

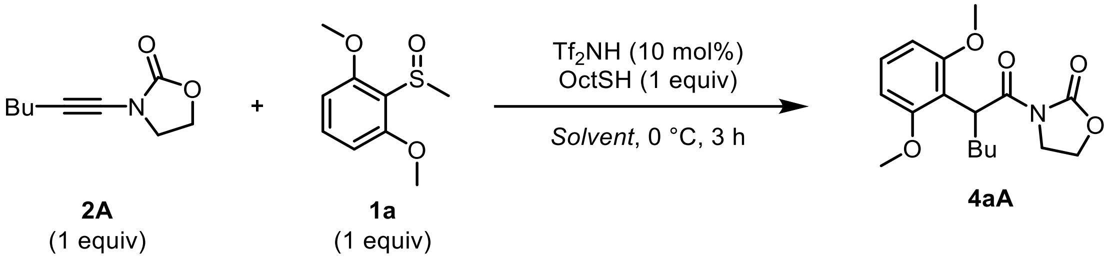

2A (1 equiv) **1a** (1 equiv)

entry	solvent (concentration)	yield[a]	entry	solvent (concentration)	yield[a]
**1**	PhMe (0.1 M)	89%	**7**	$MeNO_2$ (0.1 M)	86%
**2**	Et_2O (0.1 M)	90%	**8**	CH_2Cl_2 (0.1 M)	91%
**3**	THF (0.1 M)	76%	**9**	CH_2Cl_2 (0.4 M)	83%
**4**	EtOAc (0.1 M)	93%	**10**	CH_2Cl_2 (0.25 M)	88%
**5**	1,2-DCE (0.1 M)	90%	**11**	CH_2Cl_2 (0.05 M)	92%
**6**	MeCN (0.1 M)	80%			

Table S 2: Solvent influence: [a] Yields refer to NMR yields using mesitylene as internal standard.

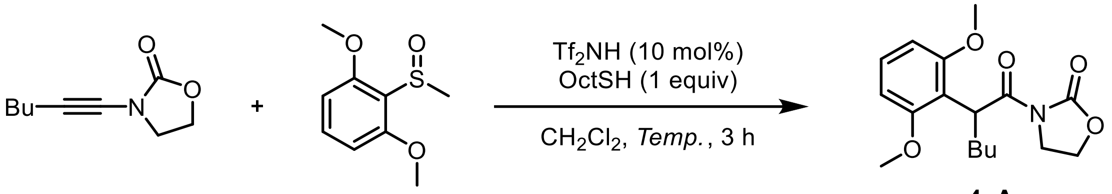

2A (1 equiv) **1a** (1 equiv)

entry	Temperature	yield[a]
**1**	0 °C	92%
**2**	-20 °C	100%
**3**	-10 °C	98%

Table S 3: Temperature influence: [a] Yields refer to NMR yields using mesitylene as internal standard.

4. Synthesis of Sulfoxides

Sulfoxides **1a** (2,6-$(OMe)_2$),[1] **1b** (2,4,6-$(OMe)_3$),[1] **1f** (2,6-Me_2-4-OMe),[2] **1j** (2-OMe naphthyl),[3] **1k** (3-CO_2Me-2-OMe naphthyl),[4] **1l** (2-Me indolyl),[5] **1m** (2,4-OMe_2),[5] 1o (2-Me,4-OH),[6] 1p (4-OH),[7] have been previously reported and were prepared according to the literature.

General procedure A: Preparation of sulfinates

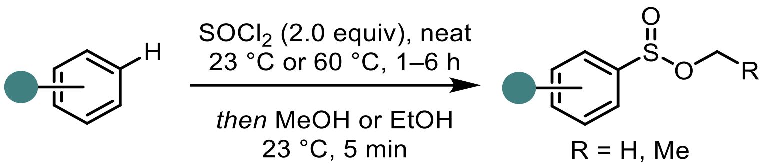

Following an adapted method developed by Bell:[6] To a two-neck flask equipped with a $CaCl_2$ guard and containing arene (1.0 equiv) was added thionyl chloride (2.0 equiv) at 23 °C. The mixture was stirred at 23 °C or 60 °C for 1 to 6 h until solidification of the suspension. Excess methanol or ethanol (40 equiv) was added and the resulting mixture was further stirred at 23 °C for 5 min. The solvent was removed *in vacuo* and the residue was purified by column chromatography (EtOAc/heptane) or crystallization from methanol to afford the alkyl sulfinate. Certain alkyl sulfinates have been found to degrade at 23 °C, therefore alkyl sulfinates were immediately converted into the corresponding sulfoxides after their purity was ensured by ^{1}H NMR.

General procedure B: Methyl sulfenylation

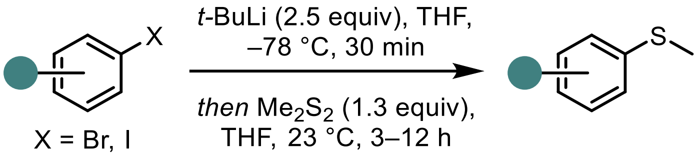

To a flame dried Schlenk flask charged with aryl bromide or iodide (1.0 equiv) in THF (0.2 M) and cooled to –78 °C was added *t*-BuLi (1.7 M in pentane, 2.5 equiv) and the reaction was stirred for 30 min at that temperature. Dimethyl disulfide (1.3 equiv) was added, the cooling bath was then removed, and the mixture was further stirred for 12 h at 23 °C. A saturated aqueous solution of NH_4Cl was added and the mixture was extracted with CH_2Cl_2 (3×). The organic phases were combined, dried over $MgSO_4$, filtered, and concentrated under reduced pressure (rotary evaporator in a fume hood). The residue was purified by column chromatography (Et_2O/heptane) to afford the methyl sulfide.

General procedure C-1: Preparation of sulfoxides from sulfides with *m*-CPBA

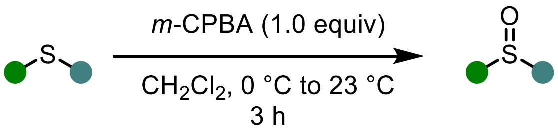

To a solution of sulfide (1.0 mmol, 1.0 equiv) in CH_2Cl_2 (5.0 ml) at 0 °C was added *m*-CPBA (0.95–1.05 mmol, 0.95–1.05 equiv) portion wise and the reaction mixture was allowed to warm to 23 °C over the course of 3 h. A saturated, aqueous solution of $NaHCO_3$ (5.0 ml) was added and the mixture was extracted with CH_2Cl_2 (3 × 5.0 ml). The combined organic phases were dried over $MgSO_4$, filtered, and concentrated *in vacuo*. Pure sulfoxides were obtained by column chromatography (acetone/heptane) or by crystallization from methyl *tert*-butyl ether or diethyl ether.

General procedure C-2: Preparation of sulfoxides from sulfides with $NaIO_4$

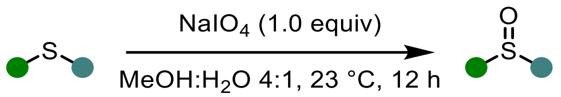

To a solution of sulfide (1.0 mmol, 1.0 equiv) in a MeOH/water mixture (4:1, 5 ml) was added sodium periodate (1.1 mmol, 1.1 equiv) and the reaction mixture was stirred for 12 h at 23 °C. The suspension was filtered, and solvent removed under reduced pressure. The residue was dissolved in CH_2Cl_2 (5.0 ml) and saturated, aqueous solution of sodium bicarbonate (5.0 ml). The mixture was extracted with CH_2Cl_2 (2 × 5.0 ml). The combined organic phases were dried over $MgSO_4$, filtered, and concentrated *in vacuo*. Pure sulfoxides were obtained by column chromatography (acetone/heptane) or by crystallization from methyl *tert*-butyl ether or diethyl ether.

General procedure C-3: Preparation of sulfoxides from sulfinates

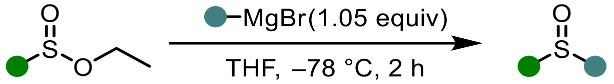

A solution of sulfinate (1.2 mmol, 1.0 equiv) in dry THF (6.0 ml) was cooled to –78 °C and treated with methylmagnesium bromide (0.42 ml of a 3 M solution in Et_2O, 1.3 mmol, 1.05 equiv). The solution was stirred for 1 h at –78 °C and 20 min at 0 °C before the cooling bath was removed and a saturated, aqueous solution of NH_4Cl (5.0 ml) was added. The mixture was extracted with CH_2Cl_2 (3 × 5.0 ml). The combined organic phases were dried over $MgSO_4$, filtered, and concentrated *in vacuo*. Pure sulfoxides were obtained by column chromatography (acetone/heptane) or by crystallization from methyl *tert*-butyl ether or diethyl ether.

pre-1c – (2-Chloro-6-methoxyphenyl)(methyl)sulfane

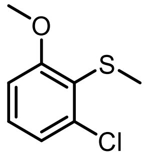

Prepared on a 17 mmol scale from 3-chlorophenol in 3 steps analogous to the procedures used below for **pre3-1d** (MOM-protection, 86%), **pre2-1d** (methylsulfenylation/deprotection, 59%) and **pre1-d** (*O*-methylation, 96%, 1.55 g) as a colorless oil.

^{1}H NMR (600 MHz, $CDCl_3$): δ 7.19 (app t, J = 8.2 Hz, 1H), 7.07 (dd, J = 8.1, 1.0 Hz, 1H), 6.80 (d, J = 8.3 Hz, 1H), 3.91 (s, 3H), 2.40 (s, 3H);

^{13}C NMR (151 MHz, $CDCl_3$): δ 161.2, 139.7, 129.5, 123.9, 122.3, 109.6, 56.5, 18.1;

HRMS (EI^+): exact mass calculated for $[M]^+$ ($C_8H_9ClOS^+$) requires *m/z* 188.0057, found *m/z* 188.0056;

IR (thin film): ν 2924, 1456, 1426, 1258, 1034, 770 cm^{-1}.

pre3-1d – 1,3-Dichloro-5-(methoxymethoxy)benzene

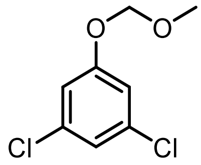

To a solution of 3,5-dichlorophenol (20 mmol, 1.0 equiv) in dry THF (30 ml) was added chloromethyl methyl ether (30 mmol, 1.5 equiv) at 0 °C. Subsequently, *N*,*N*-diisopropylethylamine (40 mmol, 2.0 equiv) was added dropwise at 0 °C. The reaction was warmed to 25 °C and stirred for another 14 h before excess chloromethyl methyl ether was quenched by addition of HCl solution (0.5 M in H_2O, 15 ml). After phase separation, the organic phase was washed with NaOH solution (1 M in H_2O, 15 ml) and with NaCl solution (saturated in H_2O, 15 ml). The organic layer was dried over $MgSO_4$, filtered, and

solvents were removed under reduced pressure. Purification by column chromatography (silica gel, EtOAc(0→5%)/heptane), afforded **pre3-1d**, in 93% yield (3.85 g) as a colorless liquid.

^{1}H-NMR (600 MHz, $CDCl_3$): δ 7.01 (t, J = 1.8 Hz, 1H), 7.01 (d, J = 1.8 Hz, 2H), 5.14 (s, 2H), 3.47 (s, 3H);

^{13}C-NMR (151 MHz, $CDCl_3$): δ 158.4, 135.4 (2C), 122.3, 115.4 (2C), 94.7, 56.4;

HRMS (EI$^+$): exact mass calculated for $[M]^+$ ($C_8H_8Cl_2O_2^+$) requires *m/z* 205.9896, found *m/z* 205.9890;

IR (thin film): ν 3089, 3000, 2958, 2934, 2905, 2827, 2780, 1587, 1571, 1154, 996 cm^{-1}.

pre2-1d – (1,5-Dichloro-3-methoxyphenyl)(methyl)sulfane

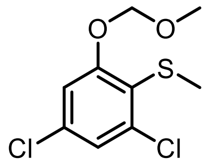

Prepared from **pre3-1d** according to an adjusted General Procedure B (using 1.1 equiv *n*-BuLi, 1.1 equiv TMEDA and 1.2 equiv dimethyl disulfide) on a 10 mmol scale in 85% yield (2.14 g) as a colorless liquid.

^{1}H-NMR (600 MHz, $CDCl_3$): δ 7.15 (t, J = 2.1 Hz, 1H), 7.09 (t, J = 2.1 Hz, 1H), 5.26 (s, 2H), 3.52 (s, 3H), 2.40 (s, 3H);

^{13}C-NMR (150 MHz, $CDCl_3$): δ 159.3, 140.0, 134.9, 123.8, 123.3, 114.3, 95.4, 56.7, 18.3.

HRMS (EI$^+$): exact mass calculated for $[M]^+$ ($C_9H_{10}Cl_2O_2S^+$) requires *m/z* 251.9773, found *m/z* 251.9769;

IR (thin film): ν 3079, 2995, 2956, 2925, 2857, 2829, 1566, 1552, 1417, 1182, 994 cm^{-1}.

pre1-1d – 3,5-Dichloro-2-(methylthio)phenol

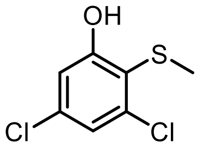

To a solution of **pre2-1d** (8.46 mmol, 1.00 equiv) in MeOH (12.7 ml) was added an HCl solution (37 % in H_2O, 84.6 mmol, 10.0 equiv) at 25 °C. The reaction was left stirring at 25°C for 22 h, before it was extracted with Et_2O (5 × 17 ml). The combined organic layers were washed with NaCl solution (saturated in H_2O, 17 ml). The organic layer was dried over $MgSO_4$, filtered, and solvents were removed under reduced pressure. Purification by LPLC (silica gel, EtOAc(0→8%)/heptane) afforded phenol **pre1-1c** in 97% yield (1.72 g) as a colorless liquid.

^{1}H-NMR (600 MHz, $CDCl_3$): δ 7.16 (s, 1H), 7.04 (d, J = 2.1 Hz, 1H), 6.94 (t, J = 2.1 Hz, 1H), 2.32 (s, 3H);

^{13}C-NMR (151 MHz, $CDCl_3$): δ 158.5, 140.5, 136.7, 121.8, 118.7, 114.0, 18.3;

HRMS (EI^+): exact mass calculated for $[M]^+$ ($C_7H_6Cl_2OS^+$) requires m/z 207.9511, found m/z 207.9508;

IR (thin film): ν 3363 (br), 3135, 3085, 2994, 2925, 2857, 2828, 1611, 1585, 1439, 944 cm^{-1}.

pre-1d – (1,5-Dichloro-3-methoxyphenyl)(methyl)sulfane

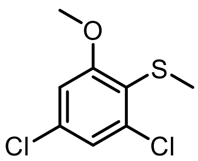

To a solution of **pre1-1d** (8.25 mmol, 1.00 equiv) dry acetone (16.5 ml) was added K_2CO_3 (12.4 mmol, 1.50 equiv) at 25 °C. After stirring the suspension at 25 °C for 30 min, MeI (12.4 mmol, 1.50 equiv) was added dropwise at 25 °C. Subsequently, the reaction was heated at reflux (56 °C) for 21 h. After cooling to 25 °C, the suspension was filtered through a Celite plug and afterwards washed with acetone (2 × 4.0 ml). Volatiles were evaporated under reduced pressure, and the resulting residue was extracted from H_2O (8.0 ml) with Et_2O (2 × 8.0 ml). The combined organic phases were washed with NaOH

solution (2.5 M in H_2O, 8.0 ml) and H_2O (2 × 8.0 ml). The organic layer was dried over $MgSO_4$ and solvents were removed under reduced pressure. Purification by LPLC (silica gel, EtOAc(1→4%)/heptane), afforded **pre-1c** in 58% yield (1.06 g) as a colorless liquid.

^{1}H-NMR (600 MHz, $CDCl_3$): δ 7.09 (d, J = 2.1 Hz, 1H), 6.79 (d, J = 2.1 Hz, 1H), 3.90 (s, 3H), 2.37 (s, 3H);

^{13}C-NMR (151 MHz, $CDCl_3$): δ 161.3, 140.1, 135.0, 122.7, 122.1, 110.5, 56.7, 18.1;

HRMS (EI$^+$): exact mass calculated for $[M]^+$ ($C_8H_8Cl_2OS^+$) requires m/z 221.9667, found m/z 221.9665;

IR (thin film): ν 3121, 3078, 3005, 2967, 2924, 2857, 2828, 1564, 1551, 1381, 1255, 1035 cm^{-1}.

pre-1e – Ethyl 2-bromo-4,6-dimethoxybenzenesulfinate

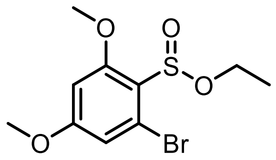

Prepared from 1-bromo-3,5-dimethoxybenzene according to General Procedure A on a 7.3 mmol scale in 38% yield (857 mg, 13:1 ratio of regioisomers) as a colorless oil.

Due to the instability of the sulfinates, only ^{1}H NMR analysis was performed, and, after confirmation of identity and purity, the material was immediately used in further reactions.

^{1}H NMR (400 MHz, $CDCl_3$): δ 6.69 (d, J = 2.3 Hz, 1H), 6.46 (d, J = 2.3 Hz, 1H), 4.26–4.10 (m, 2H), 3.89 (s, 3H), 3.83 (d, J = 1.8 Hz, 3H), 1.38 (t, J = 7.1 Hz, 3H).

pre-1h – 3,5-Diisopropyl-4-(methylsulfinyl)phenol

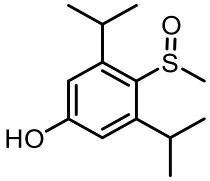

Prepared by oxidation from 3,5-diisopropyl-4-(methylthio)phenol[9] according to General Procedure C-2 on a 1.7 mmol scale in 74% yield (285 mg) as a colorless crystalline solid.

^{1}H NMR (400 MHz, DMSO): *δ* 9.82 (s br, 1H), 6.66 (s, 2H), 3.88 (s br, 2H), 2.86 (s, 3H), 1.22 (d, *J* = 6.8 Hz, 6H), 1.12 (d, *J* = 6.7 Hz, 6H);

^{13}C NMR (101 MHz, DMSO): *δ* 160.0, 151.3 (2C), 128.36, 111.8 (2C), 40.8, 27.3 (2C), 24.3 (2C), 23.8 (2C);

HRMS (ESI$^+$): exact mass calculated for $[M+Na]^+$ ($C_{13}H_{20}O_2SNa^+$) requires *m/z* 263.1076, found *m/z* 263.1073;

IR (thin film): *ν* 3160 br, 1598, 1439, 1280, 1047, 1017, 751 cm^{-1}.

pre-1i – Ethyl 2-(chloromethyl)-4,6-dimethoxybenzenesulfinate

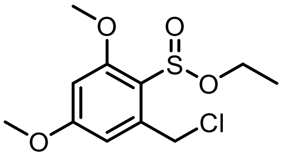

Prepared from 1-(chloromethyl)-3,5-dimethoxybenzene according to General Procedure A on a 3.0 mmol scale in 19% yield (159 mg) as a pale yellowish oil, alongside 5,7-dimethoxy-3H-benzo[c][1,2]oxathiole 1-oxide (44%).

Due to the instability of the sulfinate, only ^{1}H NMR analysis was performed, and, after confirmation of identity and purity, the material was immediately used in further reactions.

^{1}H NMR (400 MHz, $CDCl_3$): δ 6.73 (d, J = 1.6 Hz, 1H), 6.40 (d, J = 1.6 Hz, 1H), 5.26 (d, J = 12.6 Hz, 1H), 5.10 (d, J = 12.6 Hz, 1H), 4.13 (app qd, J = 7.0, 1.9 Hz, 2H), 3.84 (s, 6H), 1.35 (t, J = 7.1 Hz, 3H).

pre-1n – Ethyl 2,4-bis(allyloxy)benzenesulfinate

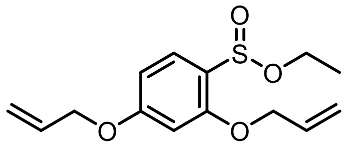

Prepared from 1,3-bis(allyloxy)benzene according to General Procedure A on a 2.6 mmol scale in 39% yield (289 mg) as a yellow oil.

Due to the instability of the sulfinate, only ^{1}H NMR analysis was performed, and, after confirmation of identity and purity, the material was immediately used in further reactions.

^{1}H NMR (600 MHz, $CDCl_3$): δ 7.76 (d, J = 8.6 Hz, 1H), 6.62 (dd, J = 8.6, 2.2 Hz, 1H), 6.49 (d, J = 2.1 Hz, 1H), 6.08–5.96 (m, 2H), 5.47–5.38 (m, 2H), 5.35–5.28 (m, 2H), 4.64–4.54 (m, 4H), 4.10 (dq, J = 10.1, 7.1 Hz, 1H), 3.84 (dq, J = 10.1, 7.1 Hz, 1H), 1.27 (t, J = 7.1 Hz, 3H).

1a, (+)-1a and (−)-1a – 1,3-Dimethoxy-2-(methylsulfinyl)benzene

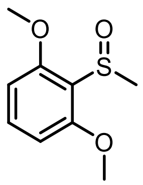

Prepared from 1,3-dimethoxy-2-(methylsulfinyl)benzene according to General Procedure C-1 on a 11.6 mmol scale in 91% yield (2.12 g) as a colorless crystalline solid. All spectroscopic data was consistent with the literature.[1]

^{1}H NMR (400 MHz, $CDCl_3$): δ 7.37 (t, J = 8.5 Hz, 1H), 6.59 (d, J = 8.5 Hz, 2H), 3.90 (s, 6H), 3.06 (s, 3H);

A racemic mixture of **1a** was separated by preparative chiral column chromatography to afford both enantiomers:

$[\alpha]_D^{20}$ = +83.0 (c = 1.0, $CHCl_3$); Enantiomeric ratio >99.5:0.5 was determined by chiral HPLC analysis: Lux-Cellulose1, (*n*-heptane+0.1%IPA)/IPA 75:25, 1.0 ml/min, 25 °C, detection at 254 nm, retention time (min): 12.6 (major) and 11.5 (minor).

$[\alpha]_D^{20}$ = −80.5 (c = 1.0, $CHCl_3$); Enantiomeric ratio >99.5:0.5 was determined by chiral HPLC analysis: Lux-Cellulose1, (*n*-heptane+0.1%IPA)/IPA 75:25, 1.0 ml/min, 25 °C, detection at 254 nm, retention time (min): 11.5 (major) and 12.7 (minor).

1c – 1-Chloro-3-methoxy-2-(methylsulfinyl)benzene

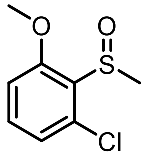

Prepared from **pre-1c** according to General Procedure C-1 on a 5.0 mmol scale in 70% yield (937 mg) as an off-white crystalline solid.

^{1}H NMR (400 MHz, $CDCl_3$): *δ* 7.34 (app t, *J* = 8.3 Hz, 1H), 7.01 (d, *J* = 8.1 Hz, 1H), 6.91 (d, *J* = 8.4 Hz, 1H), 3.95 (s, 3H), 3.06 (s, 3H);

^{13}C NMR (101 MHz, $CDCl_3$): *δ* 160.3, 135.2, 133.3, 129.0, 123.1, 111.2, 56.6, 37.9;

HRMS (ESI$^+$): exact mass calculated for $[M+Na]^+$ ($C_8H_9O_2SClNa^+$) requires *m/z* 226.9904, found *m/z* 226.9902;

IR (thin film): *ν* 1571, 1456, 1263, 1021, 782 cm^{-1}.

1d – 1,5-Dichloro-3-methoxy-2-(methylsulfinyl)benzene

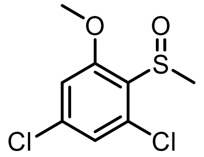

Prepared from **pre-1d** according to General Procedure C-1 on a 4.75 mml scale in 88% yield (994 mg) as a colorless solid.

^{1}H-NMR (600 MHz, $CDCl_3$): δ 7.04 (d, J = 1.9 Hz, 1H), 6.90 (d, J = 1.9 Hz, 1H), 3.95 (s, 3H), 3.05 (s, 3H);

^{13}C-NMR (150 MHz, $CDCl_3$): δ 160.4, 139.1, 135.9, 127.8, 122.9, 112.1, 56.9, 38.0;

HRMS (ESI$^+$) exact mass calculated for $[M+Na]^+$ ($C_8H_8Cl_2NaO_2S^+$) requires m/z 260.9514, found m/z 260.9512;

IR (thin film): ν 3472 (br), 3128, 3063, 3014, 2986, 2944, 2918, 2680, 1566, 1387, 1262, 1059, 1028 cm^{-1}.

1e – 1-Bromo-3,5-dimethoxy-2-(methylsulfinyl)benzene

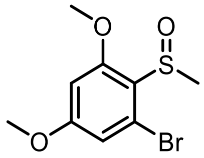

Prepared from **pre-1e** according to General Procedure C-3 on a 1.2 mmol scale in 47% yield (158 mg, >30:1 ratio of regioisomers), after crystallization from MTBE, as a colorless crystalline solid.

^{1}H NMR (400 MHz, $CDCl_3$): δ 6.71 (d, J = 2.3 Hz, 1H), 6.47 (d, J = 2.3 Hz, 1H), 3.92 (s, 3H), 3.82 (s, 3H), 3.03 (s, 3H);

^{13}C NMR (101 MHz, $CDCl_3$): δ 163.5, 161.5, 125.3, 122.9, 110.3, 99.9, 56.5, 56.0, 38.1;

HRMS (ESI$^+$): exact mass calculated for $[M+H]^+$ ($C_9H_{12}O_3SBr^+$) requires m/z 280.9665, found m/z 280.9663;

IR (thin film): *v* 2921, 1593, 1558, 1215, 1087, 1052, 1021, 830, 813 cm^{-1}.

1g – 1,5-Di-*tert*-butyl-3-methoxy-2-(methylsulfinyl)benzene

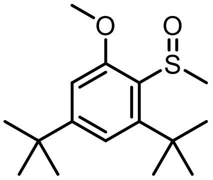

Preparation of (2,4-di-*tert*-butyl-6-methoxyphenyl)(methyl)sulfane (**pre-1g**) from 2-bromo-1,5-di-*tert*-butyl-3-methoxybenzene[10] was performed according to General Procedure B on a 0.72 mmol scale in 42% yield (79.9 mg) as a mixture with the starting material which was used directly for the oxidation without further purification.

Prepared from **pre-1g** according to General Procedure C-1 in 72% yield (61.2 mg) as a colorless crystalline solid.

^{1}H NMR (400 MHz, $CDCl_3$): δ 7.07 (d, J = 1.8 Hz, 1H), 6.94 (d, J = 1.7 Hz, 1H), 3.99 (s, 3H), 3.12 (s, 3H), 1.48 (s, 9H), 1.32 (s, 9H);

^{13}C NMR (101 MHz, $CDCl_3$): δ 161.2, 156.1, 151.3, 127.1, 116.2, 109.3, 56.4, 37.2, 36.3, 35.6, 32.8 (3C), 31.3 (3C);

HRMS (ESI^+): exact mass calculated for $[M+Na]^+$ ($C_{16}H_{26}O_2SNa^+$) requires *m/z* 305.1546, found *m/z* 305.1548;

IR (thin film): *v* 2957, 1592, 1557, 1462, 1401, 1301, 1238, 1056, 1038, 948 cm^{-1}.

1h – 1,3-Diisopropyl-5-methoxy-2-(methylsulfinyl)benzene

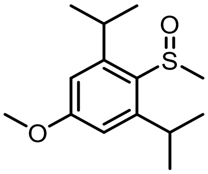

Prepared from **pre-1h** by methylation:

To a solution of **pre-1h** (149 mg, 0.62 mmol, 1 equiv) in dry acetone (1.2 ml) was added K_2CO_3 (171 mg, 1.24 mmol, 2 equiv) at 25 °C. After stirring the suspension at 25 °C for 30 min, MeI (60 µL, 0.95 mmol, 1.5 equiv) was added dropwise at 25 °C and the reacting mixture was left stirring at the same temperature for 36 h. The suspension was filtered through a Celite® plug and afterwards washed with acetone (2 × 4.0 ml). Volatiles were evaporated under reduced pressure, and the resulting residue was extracted from H_2O (8.0 ml) with Et_2O (2 × 8.0 ml). The combined organic phases were washed with NaOH solution (2.5 M in H_2O, 8.0 ml) and H_2O (2 × 8.0 ml). The organic layer was dried over $MgSO_4$ and solvents were removed under reduced pressure. Purification by flash column chromatography on silica gel (EtOAc(1→4%)/heptane), afforded **1h** in 99% yield (157 mg) as a colorless crystalline solid.

^{1}H NMR (400 MHz, $CDCl_3$): δ 6.74 (s, 2H), 3.99 (s br, 2H), 3.82 (s, 3H), 2.91 (s, 3H), 1.30 (d, J = 6.9 Hz, 6H), 1.21 (d, J = 6.7 Hz, 6H);

^{13}C NMR (101 MHz, $CDCl_3$): δ 162.0, 152.2 (2C), 129.9, 110.6 (2C), 55.2, 41.0, 28.3 (2C), 24.8 (2C), 24.1 (2C);

HRMS (ESI$^+$)**:** exact mass calculated for $[M+Na]^+$ ($C_{14}H_{22}O_2SNa^+$) requires m/z 277.1233, found m/z 277.1233;

IR (thin film): ν 2960, 1589, 1571, 1462, 1275, 1056, 1034, 1025, 872 cm^{-1}.

1i – 1-(Chloromethyl)-3,5-dimethoxy-2-(methylsulfinyl)benzene

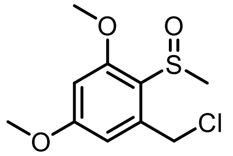

Prepared from **pre-1i** according to General Procedure C-3 on a 0.57 mmol scale in 87% yield (108 mg) as an off-white crystalline solid.

^{1}H NMR (400 MHz, $CDCl_3$): *δ* 6.55 (d, *J* = 2.4 Hz, 1H), 6.48 (d, *J* = 2.4 Hz, 1H), 5.15 (d, *J* = 11.3 Hz, 1H), 4.87 (d, *J* = 11.3 Hz, 1H), 3.91 (s, 3H), 3.85 (s, 3H), 3.05 (s, 3H);

^{13}C NMR (101 MHz, $CDCl_3$): *δ* 163.2, 160.1, 141.0, 121.6, 108.3, 100.0, 56.4, 55.8, 42.3, 39.2;

HRMS (ESI^+): exact mass calculated for $[M+Na]^+$ ($C_{10}H_{13}O_3SClNa^+$) requires *m/z* 271.0166, found *m/z* 271.0167;

IR (thin film): *v* 2944, 1594, 1457, 1329, 1205, 1165, 1086, 1053, 954 cm^{-1}.

1n – 2,4-Bis(allyloxy)-1-(methylsulfinyl)benzene

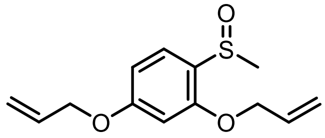

Prepared from **pre-1m** according to General Procedure C-3 on a 0.83 mmol scale in 85% yield (177 mg) as a colorless oil.

^{1}H NMR (600 MHz, $CDCl_3$): *δ* 7.70 (d, *J* = 8.6 Hz, 1H), 6.69 (dd, *J* = 8.6, 2.2 Hz, 1H), 6.49 (d, *J* = 2.2 Hz, 1H), 6.09–5.93 (m, 2H), 5.46–5.36 (m, 2H), 5.35–5.27 (m, 2H), 4.63–4.51 (m, 4H), 2.75 (s, 3H);

^{13}C NMR (151 MHz, $CDCl_3$): *δ* 162.3, 155.1, 132.8, 132.2, 126.0, 125.1, 118.4, 118.4, 106.6, 100.6, 69.4, 69.3, 41.8;

HRMS (ESI$^+$): exact mass calculated for $[M+H]^+$ ($C_{13}H_{17}O_3S^+$) requires *m/z* 253.0893, found *m/z* 253.0894;

IR (thin film): *v* 1581, 1413, 1280, 1175, 1065, 995, 929, 831 cm^{-1}.

5. Synthesis of ynamides and alkynyl sulfides

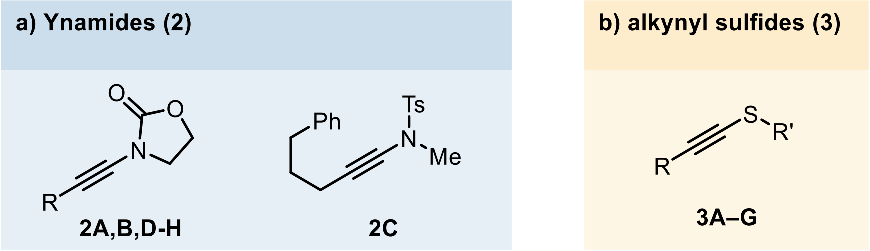

Ynamides **2A** (R = *n*-Bu),[11] **2B** (R = H),[12] **2C** (R = Me),[13] **2D** (R = Cy),[11] **2E** (R = CH_2Cy),[11] **2F** (R = $(CH_2)_3Ph$),[11] **2G** (R = $(CH_2)_8COPh$),[13] **2H** (R = $(CH_2)_8CO_2Me$),[14,15] **2L** (R = Cl, R' = Ph)[16] and **2M** (R = 2-Py, R' = Ph)[17] have been previously reported and were prepared according to the literature.

Alkynyl sulfides **3A** (R = *n*-Bu, R' = Me),[18] **3B** (R = *cyclo*-Pentyl),[19] **3C** (R = *t*-Bu, R' = Ph),[20] **3D** (R = $(CH_2)_3Cl$, R' = Me),[21] **3E** (R = $(CH_2)_3OAc$, R' = Me),[21] and **3F** (R = *n*-Bu, R' = Ph)[22] have been previously reported and were prepared according to the literature. $PhSSO_2Ph$ (**Pre-SPh**) was prepared according to the literature.[23]

Ynol ether **6** and aryl alkyne **7** are commercially available and were used as received. Amide **8** has been previously reported and was prepared according to the literature.[24]

General procedure D-1: Preparation of ynamides

Following the method developed by Stahl,[25] to a Schlenk flask were added $CuCl_2$ (20 mol%), 2-oxazolidone (5.0 equiv) and Na_2CO_3 (2.0 equiv). The reaction flask was purged with oxygen for 15 min. Pyridine (2.0 equiv) in dry toluene (0.2 M) was then added, before a balloon filled with oxygen was connected to the flask and the flask was heated at 70–80 °C. After 15 min, a solution of alkyne (1.0 equiv) in dry toluene (0.2 M) was added over 2 h using an addition funnel. After this addition, the mixture was allowed to stir at 80 °C for another 12–24 h and was then cooled to 23 °C. The reaction mixture was quenched with saturated NH_4Cl and diluted with diethyl ether. The organic layer was separated, dried with Na_2SO_4, concentrated under reduced pressure and the residue was purified by flash chromatography on silica gel with EtOAc/hexane.

2I – *N*-Methoxy-*N*-methyl-11-(2-oxooxazolidin-3-yl)undec-10-ynamide

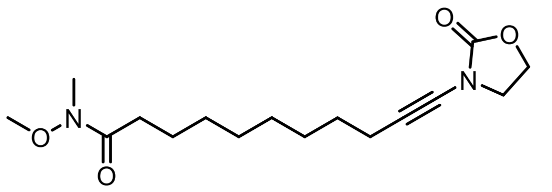

pre-2I: Prepared by amide coupling of 10-undecynoic acid with *N*,*O*-dimethylhydroxylamine hydrochloride according to a procedure by Tsukuda *et al.*[26] on an 11.5 mmol scale, yielding 97% (2.52 g) of *N*-methoxy-*N*-methylundec-10-ynamide (**pre-2I**).

Prepared from **pre-2I** according to General Procedure D-1 on a 1.2 mmol scale in 55% yield (266 mg) as a yellow solid.

^{1}H NMR (600 MHz, $CDCl_3$): *δ* 4.45–4.31 (m, 2H), 3.94–3.80 (m, 2H), 3.67 (s, 3H), 3.17 (s, 3H), 2.40 (t, *J* = 6.8 Hz, 2H), 2.28 (t, *J* = 7.2 Hz, 2H), 1.64–1.56 (m, 2H), 1.55–1.45 (m, 2H), 1.41–1.25 (m, 8H);

^{13}C NMR (151 MHz, $CDCl_3$): *δ* 174.9, 156.8, 71.3, 70.2, 62.9, 61.3, 47.2, 32.3, 32.0, 29.5, 29.4, 29.1, 28.9, 28.9, 24.7, 18.5;

HRMS (ESI$^+$): exact mass calculated for $[M+Na]^+$ ($C_{16\ h26}N_2O_4Na^+$) requires *m/z* 333.1785, found *m/z* 333.1786;

IR (thin film): *v* 2928, 2269, 1765, 1657, 1413, 1202, 1112, 1035, 751 cm^{-1}.

2J – Benzyl (*S*)-2-((*tert*-butoxycarbonyl)amino)-6-(2-oxooxazolidin-3-yl)hex-5-ynoate

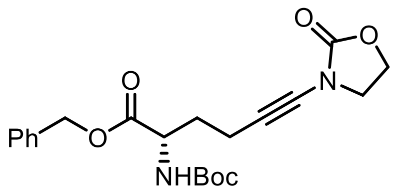

Prepared from benzyl (*S*)-2-((*tert*-butoxycarbonyl)amino)hex-5-ynoate (**pre-2J**)[27] according to General Procedure D-1 on a 1.2 mmol scale in 55% yield (266 mg) as a yellow solid.

^{1}H NMR (600 MHz, $CDCl_3$): *δ* 7.41–7.30 (m, 5H), 5.22–5.13 (m, 2H), 5.09 (s, 1H), 4.46–4.34 (m, 3H), 3.88–3.78 (m, 2H), 2.45–2.32 (m, 2H), 2.16–2.04 (m, 1H), 1.93–1.82 (m, 1H), 1.43 (s, 9H);

^{13}C NMR (151 MHz, $CDCl_3$): *δ* 172.3, 156.6, 155.5, 135.4, 128.8 (2C), 128.6, 128.4 (2C), 80.2, 71.2, 69.6, 67.4, 63.0, 53.0, 47.0, 31.8, 28.4 (3C), 15.1;

HRMS (ESI^+): exact mass calculated for $[M+Na]^+$ ($C_{21}H_{26}N_2O_6Na^+$) requires *m/z* 425.1683, found *m/z* 425.1684;

IR (thin film): *v* 3361, 2979, 2271, 1788, 1706, 1500, 1416, 1159, 1030, 733, 698 cm^{-1}.

2K – *N*,4-Dimethyl-*N*-(5-phenylpent-1-yn-1-yl)benzenesulfonamide

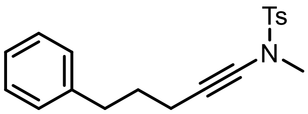

Prepared from 5-phenyl-1-pentyne according to a modified General procedure D-1 (oxazolidinone changed for *N*-methyl-*para*-toluenesulfonamide) on a 10 mmol scale in 70% yield (2.28 g) as a yellow solid.

^{1}H NMR (600 MHz, $CDCl_3$): *δ* 7.79 (d, *J* = 8.3 Hz, 2H), 7.34 (d, *J* = 8.2 Hz, 2H), 7.28 (t, *J* = 7.5 Hz, 2H), 7.19 (t, *J* = 7.4 Hz, 1H), 7.16 (d, *J* = 7.1 Hz, 2H), 3.04 (s, 3H), 2.69–2.64 (m, 2H), 2.44 (s, 3H), 2.26 (t, *J* = 6.9 Hz, 2H), 1.83–1.75 (m, 2H);

^{13}C NMR (151 MHz, $CDCl_3$): *δ* 144.6, 141.7, 133.4, 129.8 (2C), 128.7 (2C), 128.5 (2C), 128.0 (2C), 126.0, 75.5, 68.3, 39.5, 34.8, 30.6, 21.8, 18.0;

HRMS (ESI^+)**:** exact mass calculated for $[M+H]^+$ ($C_{19}H_{22}NO_2S^+$) requires *m/z* 328.1366, found *m/z* 328.1365;

IR (thin film): *v* 2927, 1687, 1361, 1167, 1087, 813, 667 cm^{-1}.

General procedure D-2: Preparation of *S*-phenyl alkynyl sulfides

Following a procedure already described in the literature.[28]

To a -78 °C solution of alkyne (1.1 equiv) in THF (0.22 M) was added dropwise a solution of *n*-BuLi in hexanes (2.3 M, 1.15 equiv) and the mixture was left stirring at -78 °C for 1 h. Then, a solution of $PhSSO_2Ph$ (**Pre-SPh**) (1 equiv) in THF (1 M) was added dropwise and the mixture was warmed to rt and left stir at this temperature for 16 h. A sat. aq. solution of NH_4Cl was added and the phases were separated. The aqueous phase was extracted with Et_2O and the combined organic phases were washed with brine, dried over $MgSO_4$, filtered and concentrated *in vacuo*. The residue was purified by flash column chromatography on silica gel (pentane) to afford the pure alkynyl sulfide.

3G – (Cyclopentylethynyl)(phenyl)sulfane

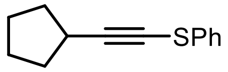

Prepared from cyclopentylacetylene according to General procedure D-2 on 2 mmol scale in 89% yield (362 mg) as a colorless oil.

^{1}H NMR (400 MHz, $CDCl_3$): δ 7.42–7.37 (m, 2H), 7.35–7.26 (m, 2H), 7.19 (m, 1H), 2.87 (m, 1H), 2.06–1.93 (m, 2H), 1.84–1.67 (m, 4H), 1.66–1.55 (m, 2H);

^{13}C NMR (101 MHz, $CDCl_3$): δ 134.1, 129.2 (2C), 126.1, 125.7 (2C), 104.3, 64.2, 34.0 (2C), 31.8, 25.2 (2C);

HRMS (APCI$^+$)**:** exact mass calculated for $[M+H]^+$ ($C_{13}H_{14}SH^+$) requires *m/z* 203.0889, found *m/z* 203.0890;

IR (thin film): *v* 3060, 2958, 2868, 1582, 1477, 1440, 1331, 1298, 1081, 1023, 897, 734, 686 cm^{-1}.

3H – Phenyl(5-phenylpent-1-yn-1-yl)sulfane

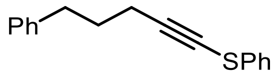

Prepared from 5-phenyl-1-pentyne according to General procedure D-2 on 2 mmol scale in 92% yield (465 mg) as a colorless oil.

^{1}H NMR (400 MHz, $CDCl_3$): *δ* 7.45–7.41 (m, 2H), 7.36–7.27 (m, 4H), 7.24–7.17 (m, 4H), 2.78 (t, *J* = 7.9 Hz, 2H), 2.47 (t, *J* = 6.9 Hz, 2H), 1.98–1.89 (m, 2H);

^{13}C NMR (101 MHz, $CDCl_3$): *δ* 141.5, 133.8, 129.2 (2C), 128.7 (2C), 128.5 (2C), 126.3, 126.1, 126.0 (2C), 99.6, 65.4, 35.0, 30.4, 19.9;

HRMS (APCI$^+$)**:** exact mass calculated for $[M+H]^+$ ($C_{17}H_{16}SH^+$) requires *m/z* 253.1045, found *m/z* 253.1043;

IR (thin film): *v* 3060, 3025, 2939, 2859, 1602, 1582, 1495, 1477, 1453, 1440, 1324, 1081, 1023, 734, 696, 686 cm^{-1}.

6. *ipso*-Coupling

General procedure E: *ipso*-Coupling

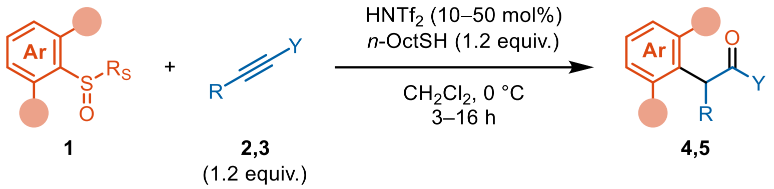

A solution of sulfoxide (**1**) (1.0 equiv), donor-substituted alkynes (**2** or **3**) (1.2 equiv) and *n*-octanethiol (1.2 equiv) in CH_2Cl_2 (0.1 M) was cooled to 0 °C. A solution of Tf_2NH in CH_2Cl_2 (0.1 M) (0.1 equiv) was added via syringe and the reaction was stirred at 0 °C until the starting material had been consumed (as determined by TLC analysis; usually 3–24 h). Solid $NaHCO_3$ (100 mg mmol^{-1}) was added, and the reaction was stirred at 23 °C for 5 min. The suspension was filtered over a plug of cotton and the solvent was evaporated under reduced pressure. The crude residue was purified by column chromatography or preparative TLC (EtOAc/heptane) to afford the arylated product (**4** or **5** respectively).

Troubleshooting:

- Triflimide (Tf_2NH) was bought from TCI and was used without further purification. As it is highly hygroscopic, and in order to ensure reproducible results, it was stored at –20 °C in a glovebox and weighed into 4 ml vials with septa caps to prepare 0.1 M stock solutions.
- Alternatively, outside the glovebox handling is possible, with storage in the freezer and fast handling being recommended to facilitate the weighing process.
- The reaction was found to be tolerant to traces of water and oxygen and was reproducible in multiple runs. To ensure reproducible results, reactions were run with dry solvents and under Ar atmosphere.
- Reactions were monitored by TLC. In most cases, the reaction is done in 3 h. For slower reactions, stirring was continued at 0 °C for 16–24 h, using a cryostat.

- In most cases, the reactions with ynamides were performed with 10 mol% triflimide. If the reaction is slow (e.g. with extremely hindered sulfoxides), competing hydrothiolation of the ynamide can be observed. In these cases (e.g., synthesis of **4gA** and **4hA**), a higher loading of 30 mol% was used). Reactions with alkynyl sulfides were performed with 50 mol% of triflimide.

4aA – 3-(2-(2,6-Dimethoxyphenyl)hexanoyl)oxazolidin-2-one

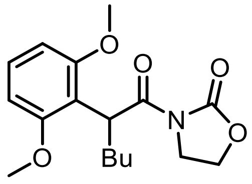

Prepared from **1a** and **2A** according to General Procedure E on a 0.2 mmol scale in 96% yield (61.9 mg) as a colorless crystalline solid.

Large scale reaction: Performed on a 5.0 mmol scale with 1.0 equiv of **1a**, 1.0 equiv of *n*-octanethiol, 2 mol% of Tf_2NH (28.2 mg) and 30 h reaction time at 0 °C using a cryostat, delivering the title compound in 96% yield (1.54 g) as a colorless crystalline solid.

^{1}H NMR (400 MHz, $CDCl_3$): δ 7.13 (t, *J* = 8.3 Hz, 1H), 6.51 (d, *J* = 8.3 Hz, 2H), 4.75 (t, *J* = 6.8 Hz, 1H), 4.28 (app td, *J* = 8.9, 4.8 Hz, 1H), 4.15 (app q, *J* = 8.7 Hz, 1H), 4.12–4.03 (m, 1H), 3.83 (ddd, *J* = 10.6, 8.8, 4.8 Hz, 1H), 3.78 (s, 6H), 2.16–2.05 (m, 1H), 1.67–1.51 (m, 1H), 1.43–1.14 (m, 4H), 0.86 (t, *J* = 7.1 Hz, 3H);

^{13}C NMR (101 MHz, $CDCl_3$): δ 175.3, 158.1 (2C), 152.8, 128.1, 117.9, 104.6 (2C), 62.0, 56.1 (2C), 43.4, 41.6, 30.5, 29.7, 23.0, 14.2;

HRMS (ESI$^+$)**:** exact mass calculated for [M+Na]$^+$ ($C_{17}H_{23}NO_5Na^+$) requires *m/z* 344.1468, found *m/z* 344.1467;

IR (thin film): *v* 2949, 2930, 1772, 1701, 1591, 1471, 1228, 1105, 1037, 724 cm^{-1}.

4bA – 3-(2-(2,4,6-Trimethoxyphenyl)hexanoyl)oxazolidin-2-one

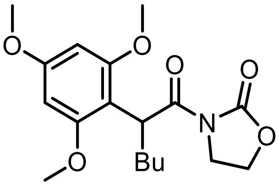

Prepared from **1b** and **2A** according to General Procedure E on a 0.2 mmol scale in 72% yield (50.9 mg) as a colorless oil.

^{1}H NMR (400 MHz, $CDCl_3$): δ 6.09 (s, 2H), 4.67 (t, *J* = 6.8 Hz, 1H), 4.28 (app td, *J* = 9.0, 4.6 Hz, 1H), 4.17 (app q, *J* = 8.8 Hz, 1H), 4.07–4.00 (m, 1H), 3.83 (ddd, *J* = 10.6, 8.9, 4.7 Hz, 1H), 3.78 (s, 3H), 3.76 (s, 6H), 2.14–1.96 (m, 1H), 1.65–1.53 (m, 1H), 1.40–1.12 (m, 4H), 0.85 (t, *J* = 7.1 Hz, 3H);

^{13}C NMR (100 MHz, $CDCl_3$): δ 175.5, 160.1, 158.8 (2C), 152.8, 110.3, 91.2 (2C), 62.0, 56.0, 56.0, 55.3, 43.4, 41.3, 30.6, 29.6, 22.9, 14.1;

HRMS (ESI^+): exact mass calculated for $[M+Na]^+$ ($C_{18}H_{25}NNaO_6^+$) requires *m/z* 374.1574, found *m/z* 374.1573;

IR (thin film): *ν* 2994, 1777, 1702, 1606, 1592, 1203, 1119, 816 cm^{-1}.

4cA – 3-(2-(2-Chloro-6-methoxyphenyl)hexanoyl)oxazolidin-2-one

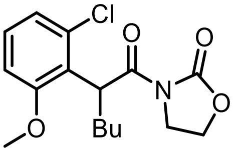

Prepared from **1c** and **2A** according to General Procedure E on a 0.25 mmol scale in 63% yield (51.2 mg) as a colorless oil.

^{1}H NMR (400 MHz, $CDCl_3$): δ 7.11 (app t, *J* = 8.1 Hz, 1H), 6.99 (d, *J* = 8.0 Hz, 1H), 6.75 (d, *J* = 8.2 Hz, 1H), 4.80 (dd, *J* = 7.6, 5.8 Hz, 1H), 4.33 (app td, *J* = 9.0, 4.9 Hz, 1H), 4.22 (app q, *J* = 8.7 Hz, 1H), 4.15–4.01 (m,

1H), 3.88 (ddd, *J* = 10.6, 9.0, 4.9 Hz, 1H), 3.76 (s, 3H), 2.25–2.11 (m, 1H), 1.72–1.57 (m, 1H), 1.52–1.19 (m, 4H), 0.87 (t, *J* = 7.1 Hz, 3H);

^{13}C NMR (101 MHz, $CDCl_3$): δ 173.7, 158.0, 152.8, 135.6, 128.2, 127.7, 122.6, 110.0, 62.1, 56.2, 45.4, 43.4, 30.4, 29.8, 22.9, 14.1;

HRMS (ESI$^+$)**:** exact mass calculated for $[M+Na]^+$ (C_{16} $_{h20}NO_4ClNa^+$) requires *m/z* 348.0973, found *m/z* 348.0972;

IR (thin film): *v* 2930, 1776, 1702, 1463, 1383, 1255, 1221, 1190, 1035, 758, 722 cm^{-1}.

4dA – 3-(2-(2,4-Dichloro-6-methoxyphenyl)hexanoyl)oxazolidin-2-one

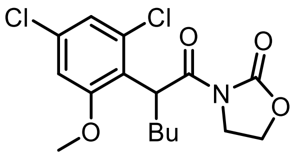

Prepared from **1d** and **2A** according to General Procedure E on a 0.1 mmol scale in 75% yield (26.9 mg) as a colorless thick oil.

^{1}H NMR (600 MHz, $CDCl_3$): δ 7.02 (d, *J* = 2.0 Hz, 1H), 6.75 (d, *J* = 2.0 Hz, 1H), 4.78 (dd, *J* = 7.6, 6.0 Hz, 1H), 4.36 (app td, *J* = 9.1, 4.9 Hz, 1H), 4.26 (app q, *J* = 8.8 Hz, 1H), 4.14–4.07 (m, 1H), 3.89 (ddd, *J* = 10.7, 8.9, 4.9 Hz, 1H), 3.78 (s, 3H), 2.21–2.13 (m, 1H), 1.64 (ddt, *J* = 13.5, 11.0, 5.6 Hz, 1H), 1.45–1.37 (m, 1H), 1.37–1.29 (m, 2H), 1.29–1.19 (m, 1H), 0.88 (t, *J* = 7.2 Hz, 3H);

^{13}C NMR (150 MHz, $CDCl_3$): δ 173.3, 158.4, 152.8, 136.0, 133.4, 126.4, 122.4, 110.8, 62.2, 56.4, 45.1, 43.4, 30.2, 29.7, 22.9, 14.1;

HRMS (ESI$^+$): exact mass calculated for $[M+Na]^+$ (C_{16} $_{h19}Cl_2NNaO_4^+$) requires *m/z* 382.0583, found *m/z* 382.0587;

IR (thin film): *v* 2956, 2930, 2870, 2860, 1782, 1707, 1582, 1567, 1042 cm^{-1}.

4eA – 3-(2-(2-Bromo-4,6-dimethoxyphenyl)hexanoyl)oxazolidin-2-one

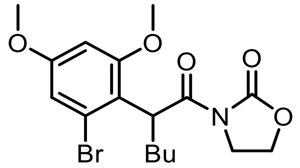

Prepared from **1e** and **2A** according to General Procedure E on a 0.12 mmol scale in 84% yield (40.5 mg) as a colorless oil.

^{1}H NMR (400 MHz, $CDCl_3$): δ 6.74 (d, *J* = 2.5 Hz, 1H), 6.36 (d, *J* = 2.4 Hz, 1H), 4.71 (dd, *J* = 7.9, 5.5 Hz, 1H), 4.33 (app td, *J* = 9.3, 4.9 Hz, 1H), 4.22 (app q, *J* = 8.7 Hz, 1H), 4.13–4.02 (m, 1H), 3.92–3.83 (m, 1H), 3.76 (s, 3H), 3.72 (s, 3H), 2.21–2.09 (m, 1H), 1.71–1.60 (m, 1H), 1.49–1.38 (m, 1H), 1.37–1.19 (m, 3H), 0.88 (t, *J* = 7.1 Hz, 3H);

^{13}C NMR (101 MHz, $CDCl_3$): δ 173.9, 159.4, 158.5, 152.8, 126.8, 121.8, 110.0, 99.0, 62.1, 56.1, 55.6, 47.7, 43.5, 30.7, 29.9, 23.0, 14.1;

HRMS (ESI$^+$)**:** exact mass calculated for $[M+Na]^+$ ($C_{17}H_{22}NO_5BrNa^+$) requires *m/z* 422.0574, found *m/z* 422.0573;

IR (thin film): *v* 2956, 1780, 1705, 1602, 1566, 1385, 1213, 1032 cm^{-1}.

4fA – 3-(2-(4-Methoxy-2,6-dimethylphenyl)hexanoyl)oxazolidin-2-one

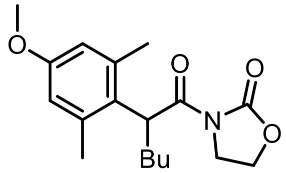

Prepared from **1f** and **2A** according to General Procedure E on a 0.11 mmol scale in 73% yield (25.4 mg) as a pale-yellow oil.

^{1}H NMR (400 MHz, $CDCl_3$): δ 6.52 (s, 2H), 4.68 (dd, J = 9.0, 4.5 Hz, 1H), 4.40–4.32 (m, 1H), 4.25 (app q, J = 8.8 Hz, 1H), 4.15–4.04 (m, 1H), 3.94 (ddd, J = 10.9, 8.9, 4.7 Hz, 1H), 3.74 (s, 3H), 2.39–2.23 (m, 7H), 1.60–1.42 (m, 2H), 1.39–1.29 (m, 2H), 1.29–1.15 (m, 1H), 0.89 (t, J = 7.2 Hz, 3H);

^{13}C NMR (101 MHz, $CDCl_3$): δ 175.5, 157.7, 152.7, 138.6 (br, 2C), 129.5, 114.5 (2C), 62.0, 55.1, 46.7, 43.5, 31.1, 30.6, 23.0, 21.3 (2C), 14.1;

HRMS (ESI$^+$)**:** exact mass calculated for $[M+Na]^+$ ($C_{18}H_{25}NO_4Na^+$) requires *m/z* 342.1676, found *m/z* 342.1676;

IR (thin film): *v* 2958, 1783, 1697, 1603, 1385, 1195 cm^{-1}.

4gA – 3-(2-(2,4-Di-*tert*-butyl-6-methoxyphenyl)hexanoyl)oxazolidin-2-one

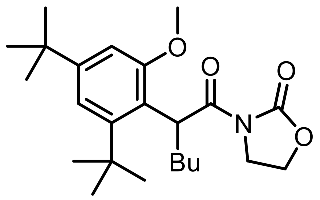

Prepared from **1g** and **2A** according to General Procedure E on a 0.09 mmol scale using 20 mol% of Tf_2NH affording the title compound in 49% yield (17.3 mg) as a colorless crystalline solid. The reaction was stirred for 16 h at 0 °C to 23 °C.

^{1}H NMR (400 MHz, $CDCl_3$): δ 7.08 (d, J = 1.9 Hz, 1H), 6.85 (d, J = 1.8 Hz, 1H), 5.49 (dd, J = 10.0, 5.3 Hz, 1H), 4.40 (app dd, J = 8.7, 7.4 Hz, 2H), 4.10–3.94 (m, 2H), 3.80 (s, 3H), 2.48–2.36 (m, 1H), 2.04 (tt, J = 12.3, 4.9 Hz, 1H), 1.39 (s, 9H), 1.32 (s, 9H), 1.42–1.14 (m, 3H), 1.07–0.95 (m, 1H), 0.82 (t, J = 7.3 Hz, 3H);

^{13}C NMR (101 MHz, $CDCl_3$): δ 174.0, 160.6, 153.5, 150.1, 149.5, 121.9, 116.8, 108.3, 62.1, 56.1, 45.4, 43.5, 36.3, 35.1, 32.4 (3C), 31.5 (3C), 30.9, 30.1, 23.3, 14.0;

HRMS (ESI$^+$)**:** exact mass calculated for $[M+Na]^+$ ($C_{24}H_{37}NO_4Na^+$) requires *m/z* 426.2615, found *m/z* 426.2606;

IR (thin film): *v* 2957, 1774, 1708, 1384, 1217 cm^{-1}.

4hA – 3-(2-(2,6-Diisopropyl-4-methoxyphenyl)hexanoyl)oxazolidin-2-one

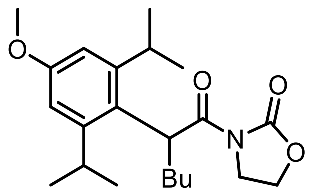

Prepared from **1h** and **2A** according to General Procedure E on a 0.1 mmol scale using 30 mol% of Tf_2NH affording the title compound in 57% yield (21.2 mg) as a colorless oil. The reaction was stirred for 16 h at 0 °C using a cryostat.

^{1}H NMR (400 MHz, $CDCl_3$): δ 6.68 (s, 2H), 5.11–4.97 (m, 1H), 4.37 (app td, *J* = 8.9, 5.5 Hz, 1H), 4.26 (app q, *J* = 8.6 Hz, 1H), 4.11–4.00 (m, 1H), 3.98–3.87 (m, 1H), 3.80 (s, 3H), 3.30 (s br, 2H), 2.31–2.18 (m, 1H), 1.73–1.60 (m, 1H), 1.52–1.42 (m, 1H), 1.38–1.19 (m, 9H), 1.18 (d, *J* = 6.7 Hz, 6H), 0.87 (t, *J* = 7.2 Hz, 3H);

^{13}C NMR (101 MHz, $CDCl_3$): δ 175.9, 158.6, 152.8, 150.2 (br, 2C), 125.9, 109.6 (br, 2C), 62.0, 55.0, 45.2, 43.8, 33.4, 31.0, 29.9 (br, 2C), 24.9 (br, 4C), 23.0, 14.1;

HRMS (ESI$^+$)**:** exact mass calculated for $[M+Na]^+$ ($C_{22}H_{33}NO_4Na^+$) requires *m/z* 398.2302, found *m/z* 398.2305;

IR (thin film): *v* 2961, 1777, 1590, 1572, 1463, 1275, 1261, 1056, 1034, 873, 680 cm^{-1}.

4iA – 3-(2-(2-(Chloromethyl)-4,6-dimethoxyphenyl)hexanoyl)oxazolidin-2-one

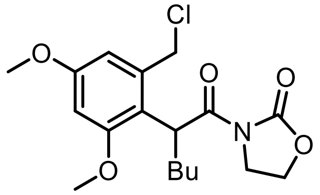

Prepared from **1i** and **2A** according to General Procedure E on a 0.1 mmol scale in 57% yield (21.2 mg) as a pale-yellow oil.

^{1}H NMR (600 MHz, $CDCl_3$): δ 6.56 (d, J = 2.5 Hz, 1H), 6.39 (d, J = 2.5 Hz, 1H), 4.91 (d, J = 11.9 Hz, 1H), 4.63–4.53 (m, 2H), 4.33 (app td, J = 9.1, 4.5 Hz, 1H), 4.20 (app q, J = 8.9 Hz, 1H), 4.11–4.04 (m, 1H), 3.92–3.85 (m, 1H), 3.80 (s, 3H), 3.74 (s, 3H), 2.26–2.18 (m, 1H), 1.69–1.62 (m, 1H), 1.49–1.40 (m, 1H), 1.38–1.22 (m, 3H), 0.87 (t, J = 7.2 Hz, 3H);

^{13}C NMR (151 MHz, $CDCl_3$): δ 174.5, 159.4, 158.6, 153.1, 138.5, 120.5, 107.2, 99.6, 62.2, 55.9, 55.4, 45.0, 44.2, 43.6, 31.4, 30.4, 23.0, 14.1;

HRMS (ESI^+)**:** exact mass calculated for $[M+Na]^+$ ($C_{18}H_{24}NO_5ClNa^+$) requires m/z 392.1235, found m/z 392.1243;

IR (thin film): ν 2957, 1777, 1707, 1606, 1463, 1385, 1222, 1202, 1155, 760 cm^{-1}.

4jA – 3-(2-(2-Methoxynaphthalen-1-yl)hexanoyl)oxazolidin-2-one

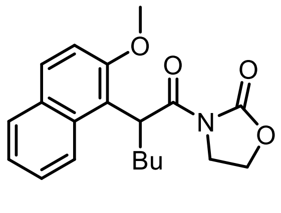

Prepared from **1j** and **2A** according to General Procedure E on a 0.11 mmol scale in 75% yield (28.0 mg) as a colorless oil.

^{1}**H NMR** (400 MHz, $CDCl_3$): δ 8.04 (d, J = 8.6 Hz, 1H), 7.80–7.71 (m, 2H), 7.49 (ddd, J = 8.5, 6.8, 1.4 Hz, 1H), 7.34 (ddd, J = 8.0, 6.8, 1.0 Hz, 1H), 7.21 (d, J = 9.0 Hz, 1H), 5.21 (dd, J = 8.0, 5.6 Hz, 1H), 4.34–4.21 (m, 1H), 4.18–4.05 (m, 2H), 3.96–3.82 (m, 4H), 2.40–2.25 (m, 1H), 1.82–1.69 (m, 1H), 1.53–1.42 (m, 1H), 1.39–1.25 (m, 3H), 0.86 (t, J = 7.1 Hz, 3H);

^{13}**C NMR** (101 MHz, $CDCl_3$): δ 175.2, 154.4, 152.8, 133.1, 129.8, 129.3, 128.8, 126.8, 123.6, 123.5, 122.5, 114.3, 62.0, 57.2, 43.8, 43.4, 31.4, 30.2, 23.0, 14.1;

HRMS (ESI$^+$)**:** exact mass calculated for $[M+Na]^+$ ($C_{20}H_{23}NO_4Na^+$) requires *m/z* 364.1519, found *m/z* 364.1518;

IR (thin film): *v* 2956, 1779, 1704, 1385, 1259, 750 cm^{-1}.

4kA – Methyl 3-methoxy-4-(1-oxo-1-(2-oxooxazolidin-3-yl)hexan-2-yl)-2-naphthoate

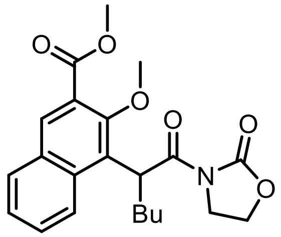

Prepared from **1k** and **2A** according to General Procedure E on a 0.1 mmol scale in 58% yield (23.3 mg) as a colorless oil.

^{1}**H NMR** (400 MHz, $CDCl_3$): δ 8.32 (s, 1H), 8.02 (d, J = 8.7 Hz, 1H), 7.85 (d, J = 7.6 Hz, 1H), 7.64 (ddd, J = 8.4, 6.9, 1.2 Hz, 1H), 7.47 (app t, J = 7.5 Hz, 1H), 5.11 (dd, J = 10.1, 2.1 Hz, 1H), 4.36–4.22 (m, 2H), 4.18–4.09 (m, 1H), 4.04 (ddd, J = 10.5, 9.1, 5.4 Hz, 1H), 3.95 (s, 3H), 3.78 (s, 3H), 2.45–2.31 (m, 1H), 1.70–1.59 (m, 2H), 1.42–1.32 (m, 3H), 0.91 (t, J = 7.1 Hz, 3H);

^{13}**C NMR** (101 MHz, $CDCl_3$): δ 173.3, 166.5, 153.2, 153.0, 135.0, 133.5, 130.6, 130.1, 130.0, 129.0, 125.6, 123.9, 123.4, 63.7, 62.2, 52.4, 44.4, 43.3, 32.7, 31.2, 23.0, 14.1;

HRMS (ESI$^+$)**:** exact mass calculated for [M+Na]$^+$ ($C_{22}H_{25}NO_6Na^+$) requires *m/z* 422.1574, found *m/z* 422. 1574;

IR (thin film): *v* 2956, 1775, 1729, 1702, 1387, 1298, 1263, 1234, 1204, 909, 759 cm^{-1}.

4lA – 3-[2-(2-methyl-1*H*-indol-3-yl)hexanoyl]oxazolidin-2-one

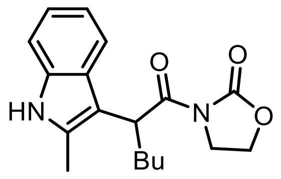

Prepared from **1l** and **2A** according to General Procedure E on a 0.2 mmol scale in 41% yield (25.8 mg) as a yellow solid.

^{1}**H NMR** (400 MHz, $CDCl_3$) δ 7.84 (s, 1H), 7.76 (dd, *J* = 7.8, 1.3 Hz, 1H), 7.23 (dd, *J* = 7.0, 1.1 Hz, 1H), 7.09 (app td, *J* = 7.6, 1.5 Hz, 1H), 7.05 (app td, *J* = 7.4, 1.4 Hz, 1H), 5.12 (dd, *J* = 8.5, 6.6 Hz, 1H), 4.31 (ddd, *J* = 9.3, 8.5, 5.4 Hz, 1H), 4.20 – 4.11 (m, 1H), 4.06 (ddd, *J* = 10.9, 9.4, 8.1 Hz, 1H), 3.86 (ddd, *J* = 10.9, 9.0, 5.3 Hz, 1H), 2.49 (s, 3H), 2.24 – 2.13 (m, 1H), 2.03 – 1.90 (m, 1H), 1.40 – 1.16 (m, 4H), 0.85 (t, *J* = 7.1 Hz, 3H);

^{13}**C NMR** (101 MHz, $CDCl_3$) δ 174.7, 153.2, 135.4, 134.0, 127.6, 121.0, 119.6, 119.5, 110.3, 108.2, 61.8, 43.1, 41.0, 31.7, 29.8, 22.7, 14.1, 12.5;

HRMS (ESI$^+$): exact mass calculated for [M+Na]$^+$ ($C_{18}H_{22}N_2O_3Na^+$) requires *m/z* 337.1523, found *m/z* 337.1510;

IR (thin film) 3386, 2928, 1776, 1695, 1460, 1386, 1221, 745 cm^{-1}.

4aB – 3-(2-(2,6-Dimethoxyphenyl)acetyl)oxazolidin-2-one

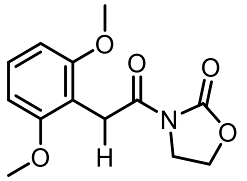

Prepared from **1a** and **2B** according to General Procedure E on a 0.26 mmol scale in 88% yield (60.8 mg) as a colorless crystalline solid.

^{1}H NMR (400 MHz, $CDCl_3$): *δ* 7.22 (t, *J* = 8.3 Hz, 1H), 6.56 (d, *J* = 8.4 Hz, 2H), 4.43–4.37 (m, 2H), 4.28 (s, 2H), 4.08–3.98 (m, 2H), 3.78 (s, 6H);

^{13}C NMR (101 MHz, $CDCl_3$): *δ* 171.4, 158.6 (2C), 154.0, 128.5, 111.0, 103.8 (2C), 62.2, 55.9 (2C), 42.8, 30.3;

HRMS (ESI$^+$)**:** exact mass calculated for $[M+Na]^+$ ($C_{13}H_{15}NO_5Na^+$) requires *m/z* 288.0842, found *m/z* 288.0841;

IR (thin film): *v* 2940, 1771, 1703, 1597, 1474, 1258, 1105, 731 cm^{-1}.

4aC – 3-(2-(2,6-Dimethoxyphenyl)propanoyl)oxazolidin-2-one

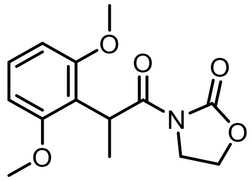

Prepared from **1a** and **2C** according to General Procedure E on a 0.1 mmol scale in 99% yield (27.6 mg) as a colorless oil.

^{1}H NMR (600 MHz, $CDCl_3$): δ 7.14 (t, J = 8.3 Hz, 1H), 6.53 (d, J = 8.3 Hz, 2H), 4.81 (q, J = 6.9 Hz, 1H), 4.31 (app td, J = 9.0, 4.6 Hz, 1H), 4.19 (app q, J = 8.8 Hz, 1H), 4.12–4.05 (m, 1H), 3.86 (ddd, J = 10.6, 8.9, 4.6 Hz, 1H), 3.80 (s, 6H), 1.36 (d, J = 6.9 Hz, 3H);

^{13}C NMR (151 MHz, $CDCl_3$): δ 175.8, 157.6 (2C), 152.8, 128.0, 119.1, 104.6 (2C), 62.0, 56.2 (2C), 43.4, 36.6, 15.8;

HRMS (ESI$^+$)**:** exact mass calculated for $[M+Na]^+$ ($C_{14}H_{17}NNaO_5^+$) requires m/z 302.0999, found m/z 302.1007;

IR (thin film): ν 2979, 2936, 2839, 1774, 1701, 1593, 1474, 1384, 1242, 1104 cm^{-1}.

4aD – 3-(2-Cyclohexyl-2-(2,6-dimethoxyphenyl)acetyl)oxazolidin-2-one

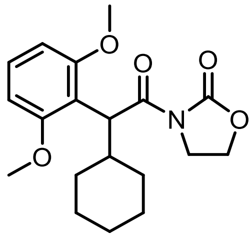

Prepared from **1a** and **2D** according to General Procedure E on a 0.2 mmol scale in 78% yield (54.1 mg) as a colorless oil.

^{1}**H NMR** (400 MHz, $CDCl_3$): *δ* 7.14 (t, *J* = 8.3 Hz, 1H), 6.50 (d, *J* = 8.3 Hz, 2H), 4.68 (d, *J* = 7.5 Hz, 1H), 4.26 (app td, *J* = 8.9, 4.3 Hz, 1H), 4.12 (app q, *J* = 8.9 Hz, 1H), 4.08–3.97 (m, 1H), 3.81 (ddd, *J* = 10.6, 8.8, 4.3 Hz, 1H), 3.78 (s, 6H), 2.30–2.17 (m, 1H), 1.94–1.85 (m, 1H), 1.74–1.56 (m, 3H), 1.39–1.03 (m, 5H), 0.96 (qd, *J* = 12.5, 3.1 Hz, 1H);

^{13}**C NMR** (100 MHz, $CDCl_3$): *δ* 174.6, 158.8 (2C), 152.6, 128.2, 115.7, 104.3 (2C), 61.8, 56.0, 46.9, 43.5, 38.6, 32.6, 29.5, 26.9, 26.9, 26.7;

HRMS (ESI^+): exact mass calculated for $[M+Na]^+$ ($C_{19}H_{25}NNaO_5^+$) requires *m/z* 370.1625, found *m/z* 370.1622;

IR (thin film): *v* 2925, 2850, 1777, 1702, 1592, 1474, 1239, 1104, 731 cm^{-1};

$[\alpha]_D^{20}$ = +1.048 (c = 0.8, $CHCl_3$); Starting from **(−)-1a**. Enantiomeric ratio 90:10 was determined by chiral HPLC analysis: Chiralpak IH-3, (*n*-heptane+0.1%IPA)/IPA 90:10, 1.0 ml/min, 25 °C, detection at 230 nm, retention time (min): 8.04 (major) and 6.55 (minor).

4aE – 3-(3-Cyclohexyl-2-(2,6-dimethoxyphenyl)propanoyl)oxazolidin-2-one

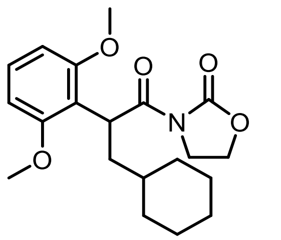

Prepared from **1a** and **2E** according to General Procedure E on a 0.2 mmol scale in 99% yield (71.7 mg) as a colorless oil.

^{1}**H-NMR** (600 MHz, $CDCl_3$): *δ* 7.14 (t, *J* = 8.3 Hz, 1H), 6.52 (d, *J* = 8.3 Hz, 2H), 4.89 (dd, *J* = 7.6, 6.1 Hz, 1H), 4.31 (app td, *J* = 9.0, 4.8 Hz, 1H), 4.19 (app q, *J* = 8.8 Hz, 1H), 4.11–4.05 (m, 1H), (ddd, *J* = 10.6, 8.9, 4.6 Hz, 1H), 3.79 (s, 6H), 2.11 (ddd, *J* = 13.5, 7.7, 6.5 Hz, 1H), 1.89 (bd, *J* = 12.7 Hz, 1H), 1.74 (bd, *J* = 13.0 Hz, 1H), 1.71–1.63 (m, 2H), 1.63–1.58 (m, 1H), 1.39–1.33 (m, 1H), 1.32–1.22 (m, 1H), 1.22–1.09 (m, 3H), 0.97–0.80 (m, 2H);

^{13}C-NMR (150 MHz, $CDCl_3$): δ 175.4, 158.0 (2C), 152.8, 128.0, 118.4, 104.6 (2C), 62.0, 56.1 (2C), 43.4, 39.2, 38.5, 35.5, 33.8, 33.4, 22.9, 26.48, 26.46;

HRMS (ESI^+) exact mass calculated for $[M + Na]^+$ ($C_{20}H_{27}NNaO_5^+$) requires *m/z* 384.1781, found *m/z* 384.1787;

IR (thin film): *v* 2920, 2849, 1777, 1703, 1593, 1474, 1384, 1245, 1106 cm^{-1}.

4aF – 3-(2-(2,6-Dimethoxyphenyl)-5-phenylpentanoyl)oxazolidin-2-one

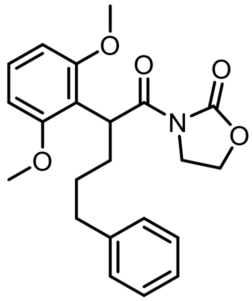

Prepared from **1a** and **2F** according to General Procedure E on a 0.2 mmol scale in 78% yield (59.8 mg) as a colorless crystalline solid.

^{1}H NMR (400 MHz, $CDCl_3$): δ 7.39–7.29 (m, 2H), 7.29–7.19 (m, 4H), 6.61 (d, *J* = 8.3 Hz, 2H), 4.92 (t, *J* = 6.5 Hz, 1H), 4.42–4.31 (m, 1H), 4.24 (app q, *J* = 8.7 Hz, 1H), 4.18–4.08 (m, 1H), 3.93 (ddd, *J* = 10.6, 8.8, 4.8 Hz, 1H), 3.86 (s, 6H), 2.84–2.63 (m, 2H), 2.35–2.18 (m, 1H), 1.94–1.62 (m, 3H);

^{13}C NMR (101 MHz, $CDCl_3$): δ 175.0, 158.1 (2C), 152.7, 142.8, 128.5 (2C), 128.2 (2C), 128.1, 125.5, 117.4, 104.5 (2C), 61.9, 56.0 (2C), 43.3, 41.5, 36.0, 30.4, 28.9;

HRMS (ESI^+)**:** exact mass calculated for $[M+Na]^+$ ($C_{22}H_{25}NO_5Na^+$) requires *m/z* 406.1625, found *m/z* 406.1626;

IR (thin film): *v* 2933, 1777, 1702, 1592, 1473, 1241, 1103, 1038, 728, 701 cm^{-1}.

4aG – 2-(2,6-Dimethoxyphenyl)-1-(2-oxooxazolidin-3-yl)-11-phenylundecane-1,11-dione

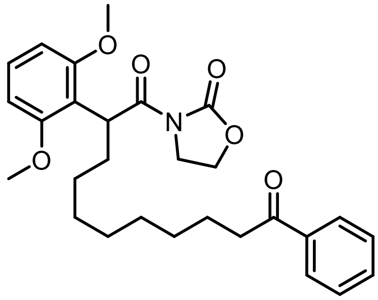

Prepared from **1a** and **2G** according to General Procedure E on a 0.2 mmol scale in 97% yield (93.7 mg) as a colorless oil.

^{1}H NMR (600 MHz, $CDCl_3$): δ 7.98–7.92 (m, 2H), 7.56–7.51 (m, 1H), 7.48–7.42 (m, 2H), 7.14 (t, *J* = 8.3 Hz, 1H), 6.52 (d, *J* = 8.3 Hz, 2H), 4.74 (t, *J* = 6.8 Hz, 1H), 4.29 (app td, *J* = 9.2, 4.7 Hz, 1H), 4.18 (app q, *J* = 8.8 Hz, 1H), 4.08–4.01 (m, 1H), 3.85 (ddd, *J* = 10.6, 9.0, 4.7 Hz, 1H), 3.78 (s, 6H), 2.97–2.89 (m, 2H), 2.16–2.03 (m, 1H), 1.75–1.65 (m, 2H), 1.64–1.56 (m, 1H), 1.42–1.18 (m, 10H);

^{13}C NMR (151 MHz, $CDCl_3$): δ 200.8, 175.2, 158.1 (2C), 152.9, 137.2, 133.0, 128.7 (2C), 128.2 (2C), 128.1, 117.7, 104.5 (2C), 62.0, 56.1 (2C), 43.4, 41.7, 38.8, 30.7, 29.8, 29.6, 29.5 (2C), 27.4, 24.5;

HRMS (ESI$^+$)**:** exact mass calculated for $[M+Na]^+$ ($C_{28}H_{35}NO_6Na^+$) requires *m/z* 504.2357, found *m/z* 504.2358;

IR (thin film): *v* 2931, 1782, 1704, 1687, 1594, 1474, 1240, 1108 cm^{-1}.

4aH – Methyl 10-(2,6-dimethoxyphenyl)-11-oxo-11-(2-oxooxazolidin-3-yl)undecanoate

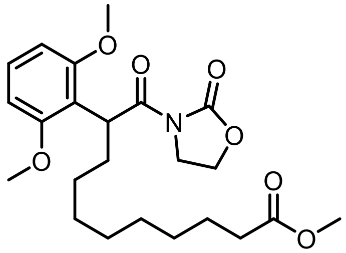

Prepared from **1a** and **2H** according to General Procedure E on a 0.2 mmol scale in 93% yield (81.1 mg) as a colorless oil.

^{1}H NMR (600 MHz, $CDCl_3$): δ 7.13 (t, J = 8.3 Hz, 1H), 6.51 (d, J = 8.3 Hz, 2H), 4.74 (t, J = 6.8 Hz, 1H), 4.29 (app td, J = 9.2, 4.7 Hz, 1H), 4.17 (app q, J = 8.8 Hz, 1H), 4.09–4.03 (m, 1H), 3.84 (ddd, J = 10.6, 8.9, 4.7 Hz, 1H), 3.78 (s, 6H), 3.65 (s, 3H), 2.27 (t, J = 7.6 Hz, 2H), 2.13–2.03 (m, 1H), 1.62–1.55 (m, 3H), 1.39–1.19 (m, 10H);

^{13}C NMR (151 MHz, $CDCl_3$): δ 175.2, 174.5, 158.1 (2C), 152.8, 128.0, 117.8, 104.5 (2C), 62.0, 56.1 (2C), 51.5, 43.4, 41.6, 34.2, 30.7, 29.8, 29.4, 29.3, 29.2, 27.4, 25.1;

HRMS (ESI$^+$)**:** exact mass calculated for $[M+Na]^+$ ($C_{23}H_{33}NO_7Na^+$) requires *m/z* 458.2149, found *m/z* 458.2155;

IR (thin film): *v* 2931, 1782, 1736, 1705, 1594, 1474, 1245, 1108 cm^{-1}.

4al – 10-(2,6-Dimethoxyphenyl)-*N*-methoxy-*N*-methyl-11-oxo-11-(2-oxooxazolidin-3-yl)undecane-amide

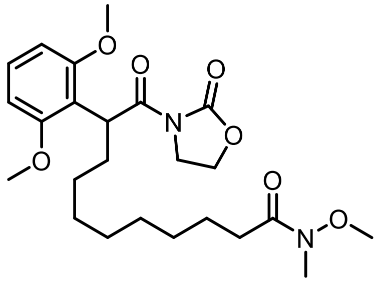

Prepared from **1a** and **2l** according to General Procedure E on a 0.1 mmol scale in 99% yield (46.4 mg) as a colorless oil.

^{1}H NMR (600 MHz, $CDCl_3$): *δ* 7.14 (t, *J* = 8.3 Hz, 1H), 6.52 (d, *J* = 8.3 Hz, 2H), 4.74 (t, *J* = 6.8 Hz, 1H), 4.30 (app td, *J* = 9.0, 4.7 Hz, 1H), 4.18 (app q, *J* = 8.8 Hz, 1H), 4.10–4.02 (m, 1H), 3.85 (ddd, *J* = 10.6, 9.0, 4.7 Hz, 1H), 3.78 (s, 6H), 3.67 (s, 3H), 3.16 (s, 3H), 2.38 (t, *J* = 7.3 Hz, 2H), 2.13–2.04 (m, 1H), 1.63–1.56 (m, 3H), 1.42–1.19 (m, 10H);

^{13}C NMR (151 MHz, $CDCl_3$): *δ* 175.2, 175.0 (br) ,158.1 (2C), 152.8, 128.1, 117.8, 104.5 (2C), 62.0, 61.3, 56.1 (2C), 43.4, 41.7, 32.3 (br), 32.0 (br), 30.7, 29.8, 29.6, 29.5 (2C), 27.4, 24.8;

HRMS (ESI$^+$)**:** exact mass calculated for $[M+Na]^+$ ($C_{24}H_{36}N_2O_7Na^+$) requires *m/z* 487.2415, found *m/z* 487.2413;

IR (thin film): *v* 2930, 1781, 1704, 1660, 1474, 1384, 1107 cm^{-1}.

4aJ – Benzyl (*S*)-2-[(*tert*-butoxycarbonyl)amino]-5-(2,6-dimethoxyphenyl)-6-oxo-6-(2-oxo-oxazolidin-3-yl)hexanoate

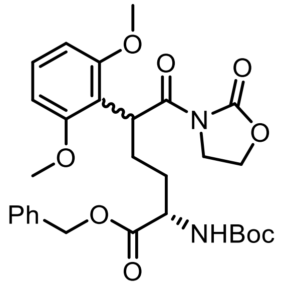

Prepared from **1a** and **2J** according to General Procedure E on a 0.1 mmol scale in 89% yield (43.5 mg, as a 1.1:1 mixture of diastereomers) as a colorless oil.

^{1}H NMR (500 MHz, $CDCl_3$): δ 7.40-7.22 (m, 5H), 7.19–7.11 (m, 1H), 6.61–6.42 (m, 2H), 5.25–4.79 (m, 3H), 4.79–4.69 (m, 1H), 4.47–4.23 (m, 2H), 4.22–4.13 (m, 1H), 4.10–3.97 (m, 1H), 3.92–3.64 (m, 7H), 2.20–2.08 (m, 1H), 1.93–1.80 (m, 1H), 1.79–1.64 (m, 2H), 1.46–1.32 (m, 9H);

^{13}C NMR (126 MHz, $CDCl_3$): δ 174.6, 174.6, 173.0, 173.0, 158.0 (2C), 157.9 (2C), 155.6*, 152.7*, 135.7, 135.7, 128.6 (2C), 128.6 (2C), 128.5 (2C)*, 128.3, 128.3, 128.3, 128.2, 116.6, 116.4, 104.5 (2C), 104.4 (2C), 79.8, 79.8, 66.9*, 62.0*, 56.1 (2C), 56.0 (2C), 54.1, 53.7, 43.3*, 41.3, 40.9, 29.8, 29.6, 28.5 (3C), 28.3 (3C), 26.5, 26.1; * = same ^{13}C shift for both diastereomers;

HRMS (ESI^{+})**:** exact mass calculated for $[M+Na]^{+}$ ($C_{29}H_{36}N_2O_9Na^{+}$) requires *m/z* 579.2313, found *m/z* 579.2318;

IR (thin film): ν 3367, 2972, 1782, 1741, 1705, 1594, 1475, 1386, 1365, 1167, 1107, 732 cm^{-1}.

5aA – *S*-Methyl 2-(2,6-dimethoxyphenyl)hexanethioate

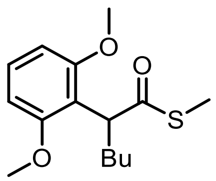

Adapted from a literature procedure:[29] A solution of hex-1-yn-1-yl(methyl)sulfane (**5A**) (25.6 mg, 0.2 mmol, 1.0 equiv) and sulfoxide (48.1 mg, 0.24 mmol, 1.2 equiv) in CH_2Cl_2 (2.0 ml) was cooled to 0 °C in an ice bath, after which *n*-octanethiol (35 µL, 0.2 mmol, 1.0 equiv) and a solution of Tf_2NH (1.0 ml, 28.1 mg/mL in CH_2Cl_2, 0.5 equiv) were added and reaction was stirred at 0 °C for 3 h. Solid $NaHCO_3$ (20 mg) was added and the mixture was filtered, evaporated *in vacuo* and purified by flash column chromatography on silica gel (EtOAc(0→10%)/heptane) to give the desired product in 77% yield (43.2 mg) as a colorless oil.

^{1}H NMR (400 MHz, $CDCl_3$): δ 7.24 (t, *J* = 8.3 Hz, 1H), 6.57 (d, *J* = 8.3 Hz, 2H), 4.28 (dd, *J* = 9.3, 5.0 Hz, 1H), 3.79 (s, 6H), 2.31–2.14 (m, 4H), 1.83 (dtd, *J* = 14.6, 9.6, 5.2 Hz, 1H), 1.39–1.13 (m, 3H), 1.13–0.99 (m, 1H), 0.83 (t, *J* = 7.2 Hz, 3H);

^{13}C NMR (101 MHz, $CDCl_3$): δ 202.3, 159.0 (2C), 129.0, 116.5, 104.4 (2C), 55.9 (2C), 49.3, 29.7, 28.8, 22.7, 14.1, 11.7;

HRMS (ESI$^+$)**:** exact mass calculated for $[M+Na]^+$ ($C_{15}H_{22}O_3SNa^+$) requires *m/z* 305.1182, found *m/z* 305.1187;

IR (thin film): *v* 2956, 2930, 1692, 1594, 1474, 1251, 1101, 788 cm^{-1};

$[\alpha]_D^{20}$ = −100.0 (c = 0.4, $CHCl_3$); Starting from **(+)-1a**. Enantiomeric ratio 86:14 was determined by chiral HPLC analysis: Lux-3 Cellulose-3, *n*-heptane/IPA 98:2, 0.7 mL/min, 25 °C, detection at 230 nm, retention time (min): 22.9 (major) and 17.0 (minor).

5aB – *S*-Methyl 2-cyclopentyl-2-(2,6-dimethoxyphenyl)ethanethioate

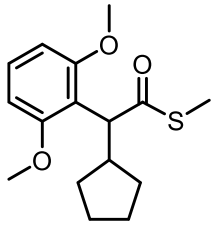

Prepared from **3B** and **1a** according to General Procedure E on a 0.2 mmol scale using 50 mol% of Tf_2NH in 70% yield (41.0 mg) as a colorless oil.

^{1}H NMR (600 MHz, $CDCl_3$): δ 7.23 (t, J = 8.3 Hz, 1H), 6.56 (d, J = 8.3 Hz, 2H), 4.20 (d, J = 9.7 Hz, 1H), 3.80 (s, 6H), 2.85–2.76 (m, 1H), 2.27–2.20 (m, 1H), 2.18 (s, 3H), 1.56–1.58 (m, 2H), 1.54–1.45 (m, 1H), 1.41–1.32 (m, 1H), 1.31–1.20 (m, 2H), 0.96–0.87 (m, 1H);

^{13}C NMR (151 MHz, $CDCl_3$): δ 201.4, 158.9 (2C), 128.9, 116.8, 104.4 (2C), 56.0 (2C), 55.0, 39.7, 33.2, 30.9, 25.4, 24.6, 11.7;

HRMS (ESI$^+$)**:** exact mass calculated for $[M+Na]^+$ (C_{16} $_{h22}NaO_3S$ $^+$) requires *m/z* 317.1182, found *m/z* 317.1182;

IR (thin film): *v* 2949, 2866, 2837, 1693, 1591, 1472, 1233, 1103, 1134 cm^{-1}.

5aC – *S*-Phenyl 2-(2,6-dimethoxyphenyl)-3,3-dimethylbutanethioate

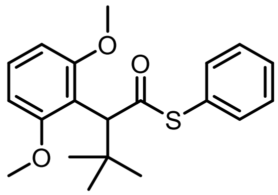

Prepared from **3C** and **1a** according to General Procedure E on a 0.1 mmol scale using 50 mol% of Tf_2NH in 67% yield (23.1 mg) as a colorless oil. The reaction was stirred for 16 h at 0 °C using a cryostat.

^{1}H NMR (600 MHz, $CDCl_3$): *δ* 7.38–7.28 (m, 6H), 6.63 (d, *J* = 8.2 Hz, 1H), 6.59 (d, *J* = 8.2 Hz, 1H), 4.39 (s, 1H), 3.89 (s, 3H), 3.79 (s, 3H), 1.03 (s, 9H);

^{13}C NMR (151 MHz, $CDCl_3$): *δ* 196.9, 159.3, 158.9, 135.0 (2C), 130.0, 129.4, 129.0 (2C), 128.7, 115.2, 104.6, 104.0, 58.0, 56.1, 55.1, 36.7, 28.8 (3C);

HRMS (ESI$^+$)**:** exact mass calculated for $[M+Na]^+$ ($C_{20}H_{24}O_3SNa^+$) requires *m/z* 367.1338, found *m/z* 367.1333;

IR (thin film): *v* 2993, 2956, 2906, 2868, 2837, 1720, 1592, 1473, 1438, 1241, 1104, 1043, 1010, 837, 783, 742, 712, 689 cm^{-1}.

5hA – *S*-Methyl 2-(2,6-diisopropyl-4-methoxyphenyl)hexanethioate

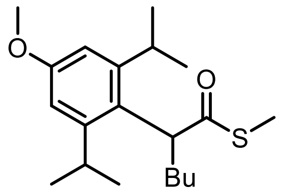

Prepared from **3A** and **1h** according to general procedure E on a 0.1 mmol scale using 50 mol% Tf_2NH in 65% yield (21.9 mg) as a colorless oil.

^{1}H NMR (700 MHz, $CDCl_3$): *δ* 6.73 (d, *J* = 2.5 Hz, 1H), 6.67 (d, *J* = 2.5 Hz, 1H), 4.11 (dd, *J* = 7.9, 5.8 Hz, 1H), 3.82 (s, 3H), 3.23 (m, 1H), 2.78 (m, 1H), 2.43 (m, 1H), 2.22 (s, 3H), 1.58 (m, 1H), 1.37–1.26 (m, 3H), 1.33 (d, *J* = 6.7 Hz, 3H), 1.26 (d, *J* = 6.7 Hz, 3H), 1.15 (d, *J* = 6.7 Hz, 3H), 1.13 (m, 4H), 0.85 (t, *J* = 7.2 Hz, 3H);

^{13}C NMR (176 MHz, $CDCl_3$): *δ* 203.6, 159.2, 150.2, 149.6, 126.4, 110.3, 109.0, 55.0, 53.8, 32.0, 30.6, 30.5, 30.0, 25.4, 25.1, 24.1, 23.4, 23.1, 14.1, 11.9;

HRMS (ESI$^+$)**:** exact mass calculated for $[M+Na]^+$ ($C_{20}H_{32}O_2SNa^+$) requires *m/z* 359.2015, found *m/z* 359.2010;

IR (thin film): *v* 2958, 2928, 2869, 1681, 1602, 1581, 1464, 1383, 1337, 1299, 1283, 1199, 1037, 923, 869, 849, 741 cm^{-1}.

5hD – *S*-Methyl 5-chloro-2-(2,6-diisopropyl-4-methoxyphenyl)pentanethioate

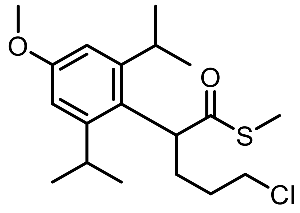

Prepared from **3D** and **1h** according to general procedure E on a 0.1 mmol scale using 50 mol% Tf_2NH in 52% yield (18.6 mg) as a colorless oil.

^{1}H NMR (600 MHz, $CDCl_3$): δ 6.73 (d, J = 2.5 Hz, 1H), 6.68 (d, J = 2.5 Hz, 1H), 4.13 (t, J = 7.2 Hz, 1H), 3.82 (s, 3H), 3.55–3.45 (m, 2H), 3.20 (m, 1H), 2.75 (m, 1H), 2.53 (m, 1H), 2.23 (s, 3H), 1.88 (m, 1H), 1.75 (m, 1H), 1.62 (m, 1H), 1.33 (d, J = 6.7 Hz, 3H), 1.27 (d, J = 6.7 Hz, 3H), 1.17 (d, J = 6.8 Hz, 3H), 1.13 (d, J = 6.8 Hz, 3H);

^{13}C NMR (151 MHz, $CDCl_3$): δ 203.2, 159.5, 150.3, 149.5, 125.6, 110.5, 109.2, 55.0, 53.1, 45.1, 31.4, 30.7, 30.0, 29.8, 25.4, 25.1, 24.1, 23.4, 11.9;

HRMS (ESI$^+$)**:** exact mass calculated for $[M+Na]^+$ ($C_{19}H_{29}ClO_2SNa^+$) requires *m/z* 379.1469, found *m/z* 379.1469;

IR (thin film): *v* 2961, 2928, 2869, 1679, 1601, 1580, 1464, 1284, 1199, 1121, 938, 868, 850, 794, 759, 612 cm^{-1}.

5hE – 4-(2,6-Diisopropyl-4-methoxyphenyl)-5-(methylthio)-5-oxopentyl acetate

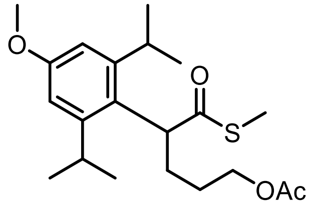

Prepared from **3E** and **1h** according to general procedure E on a 0.1 mmol scale using 50 mol% Tf_2NH in 38% yield (14.4 mg) as a colorless oil.

^{1}H NMR (600 MHz, $CDCl_3$): *δ* 6.73 (d, *J* = 2.6 Hz, 1H), 6.68 (d, *J* = 2.6 Hz, 1H), 4.14 (t, *J* = 6.6 Hz, 1H), 4.08–3.99 (m, 2H), 3.82 (s, 3H), 3.21 (sept, *J* = 6.7 Hz, 1H), 2.74 (sept, *J* = 6.7 Hz, 1H), 2.47 (m, 1H), 2.22 (s, 3H), 2.00 (s, 3H), 1.75–1.64 (m, 2H), 1.46 (m, 1H), 1.33 (d, *J* = 6.7 Hz, 3H), 1.27 (d, *J* = 6.7 Hz, 3H), 1.16 (d, *J* = 6.7 Hz, 3H), 1.13 (d, *J* = 6.7 Hz, 3H);

^{13}C NMR (151 MHz, $CDCl_3$): *δ* 203.3, 171.2, 159.4, 150.3, 149.6, 125.6, 110.5, 109.2, 64.5, 55.0, 53.2, 30.7, 30.0, 28.7, 27.3, 25.4, 25.1, 24.1, 23.3, 21.0, 11.9;

HRMS (ESI^+)**:** exact mass calculated for $[M+Na]^+$ ($C_{21}H_{32}O_4SNa^+$) requires *m/z* 403.1914, found *m/z* 403.1919;

IR (thin film): *v* 2962, 2930, 2870, 1737, 1679, 1601, 1465, 1363, 1234, 1200, 1122, 1035, 869, 793, 759 cm^{-1}.

5hF – *S*-Phenyl 2-(2,6-diisopropyl-4-methoxyphenyl)hexanethioate

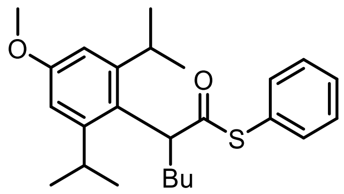

Prepared from **3F** and **1h** according to general procedure E on a 0.05 mmol scale using 50 mol% Tf_2NH in 71% yield (14.1 mg) as a colorless oil.

^{1}H NMR (600 MHz, $CDCl_3$): *δ* 7.39 -7.35 (m, 3H), 7.34–7.30 (m, 2H), 6.78 (d, *J* = 2.8 Hz, 1H), 6.73 (d, *J* = 2.8 Hz, 1H), 4.24 (dd, *J* = 7.2, 6.2 Hz, 1H), 3.85 (s, 3H), 3.32 (sept, *J* = 6.7 Hz, 1H), 2.88 (sept, *J* = 6.7 Hz, 1H), 2.41 (m, 1H), 1.58 (m, 1H), 1.41 (d, *J* = 6.7 Hz, 3H), 1.39 -1.23 (m, 3H), 1.30 (d, *J* = 6.7 Hz, 6H), 1.18 (d, *J* = 6.7 Hz, 3H), 1.15 (m, 1H), 0.85 (t, *J* = 7.2 Hz, 3H);

^{13}C NMR (151 MHz, $CDCl_3$): δ 201.1, 159.5, 150.4, 149.8, 134.7 (2C), 129.14 (2C), 129.10, 129.06, 125.8, 110.4, 109.2, 55.0, 54.0, 32.2, 30.7, 30.5, 30.2, 25.6, 25.1, 24.2, 23.7, 23.1, 14.0;

HRMS (ESI$^+$)**:** exact mass calculated for $[M+Na]^+$ ($C_{25}H_{34}O_2SNa^+$) requires *m/z* 421.2172, found *m/z* 421.2177;

IR (thin film): *v* 2958, 2928, 2868, 1698, 1602, 1464, 1439, 1383, 1337, 1299, 1283, 1199, 1122, 1036, 919, 743 cm^{-1}.

5hG – *S*-phenyl 2-cyclopentyl-2-(2,6-diisopropyl-4-methoxyphenyl)ethanethioate

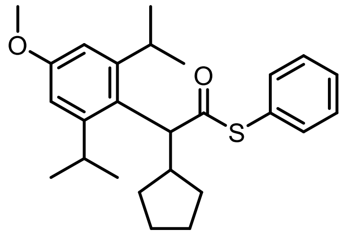

Prepared from **3G** and **1h** according to general procedure E on a 0.05 mmol scale using 50 mol% Tf_2NH in 41% yield (8.4 mg) as a colorless oil.

^{1}H NMR (700 MHz, $CDCl_3$): δ 7.41–7.34 (m, 3H), 7.33–7.30 (m, 2H), 6.77 (d, *J* = 2.8 Hz, 1H), 6.72 (d, *J* = 2.8 Hz, 1H), 4.18 (d, *J* = 10.0 Hz, 1H), 3.85 (s, 3H), 3.34 (sept, *J* = 6.8 Hz, 1H), 3.02 (sept, *J* = 6.8 Hz, 1H), 2.61 (m, 1H), 2.33 (m, 1H), 1.68 -1.58 (m, 2H), 1.50 (m, 1H), 1.43 (d, *J* = 6.8 Hz, 3H), 1.36 (m, 1H), 1.32 (d, *J* = 6.8 Hz, 3H), 1.29–1.22 (m, 5H),1.17 (d, *J* = 6.8 Hz, 3H), 0.90 (m, 1H);

^{13}C NMR (176 MHz, $CDCl_3$): δ 200.6, 159.4, 150.3, 149.9, 134.7 (2C), 129.16, 129.12 (2C), 129.05, 125.5, 110.3, 109.4, 60.0, 55.0, 42.4, 34.0, 31.6, 30.8, 30.1, 25.6, 25.5, 25.3, 24.7, 24.2, 23.9;

HRMS (ESI$^+$)**:** exact mass calculated for $[M+Na]^+$ ($C_{26}H_{34}O_2SNa^+$) requires *m/z* 433.2172, found *m/z* 433.2162;

IR (thin film): *v* 2957, 2927, 2867, 1700, 1601, 1581, 1462, 1439, 1284, 1200, 1036, 981, 868, 849, 741, 688 cm^{-1}.

5hH – *S*-Phenyl 2-(2,6-diisopropyl-4-methoxyphenyl)-5-phenylpentanethioate

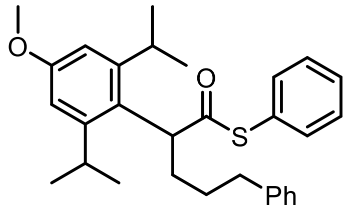

Prepared from **3H** and **1h** according to general procedure E on a 0.05 mmol scale using 50 mol% Tf_2NH in 59% yield (13.7 mg) as a colorless oil.

^{1}H NMR (700 MHz, $CDCl_3$): δ 7.39–7.34 (m, 3H), 7.33–7.29 (m, 2H), 7.22 (t, *J* = 7.3 Hz, 2H), 7.13 (t, *J* = 7.1 Hz, 1H), 7.09 (d, *J* = 7.3 Hz, 2H), 6.76 (d, *J* = 2.5 Hz, 1H), 6.71 (d, *J* = 2.5 Hz, 1H), 4.26 (t, *J* = 6.8 Hz, 1H), 3.84 (s, 3H), 3.29 (sept, *J* = 6.8 Hz, 1H), 2.83 (sept, *J* = 6.8 Hz, 1H), 2.63 (m ,1H), 2.55 (m, 1H), 2.44 (m, 1H), 1.75 (m, 1H), 1.62 (m, 1H), 1.47 (m, 1H), 1.40 (d, *J* = 6.7 Hz, 3H), 1.28 (d, *J* = 6.7 Hz, 3H), 1.27 (d, *J* = 6.7 Hz, 3H), 1.08 (d, *J* = 6.7 Hz, 3H);

^{13}C NMR (176 MHz, $CDCl_3$): δ 201.0, 159.5, 150.4, 149.7, 142.1, 134.7 (2C), 129.1 (3C), 129.0, 128.5 (2C), 128.4 (2C), 125.9, 125.5, 110.5, 109.2, 55.0, 53.9, 36.2, 32.2, 30.7, 30.14, 30.05, 25.4, 25.1, 24.2, 23.6;

HRMS (ESI^+)**:** exact mass calculated for $[M+Na]^+$ ($C_{30}H_{36}O_2SNa^+$) requires *m/z* 483.2328, found *m/z* 483.2324;

IR (thin film): *v* 2959, 2926, 2867, 1697, 1601, 1581, 1463, 1439, 1284, 1200, 1038, 868, 745, 699, 689 cm^{-1}.

4aK – 2-(2,6-Dimethoxyphenyl)-*N*-methyl-5-phenyl-*N*-tosylpentanamide

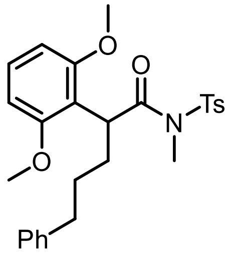

Prepared from **2K** and **1a** according to general procedure E on a 0.2 mmol scale in 70% yield (67.0 mg) as a colorless oil.

^{1}H NMR (400 MHz, $CDCl_3$): δ 7.85 (d, *J* = 8.3 Hz, 2H), 7.29 (d, *J* = 8.0 Hz, 2H), 7.24–7.17 (m, 3H), 7.15–7.04 (m, 3H), 6.51 (d, *J* = 8.4 Hz, 2H), 4.11 (dd, *J* = 8.4, 5.4 Hz, 1H), 3.64 (s, 6H), 2.98 (s, 3H), 2.62–2.44 (m, 2H), 2.42 (s, 3H), 2.14–2.03 (m, 1H), 1.75–1.63 (m, 1H), 1.61–1.48 (m, 1H), 1.46–1.33 (m, 1H);

^{13}C NMR (101 MHz, $CDCl_3$): δ 174.6, 157.9 (2C), 144.0, 142.8, 137.0, 129.2 (2C), 128.9, 128.8 (2C), 128.4 (2C), 128.2 (2C), 125.6, 115.1, 104.0 (2C), 55.7 (2C), 41.7, 36.0, 32.7, 30.1, 28.5, 21.7;

HRMS (ESI$^+$)**:** exact mass calculated for $[M+H]^+$ ($C_{27}H_{31}NO_5S^+$) requires *m/z* 482.1996, found *m/z* 482.1994;

IR (thin film): *ν* 2934, 1703, 1593, 1474, 1349, 1241, 1164, 1104, 909, 727 cm^{-1}.

4aL – 2-Chloro-2-(2,6-dimethoxyphenyl)-*N*-phenyl-*N*-tosylacetamide

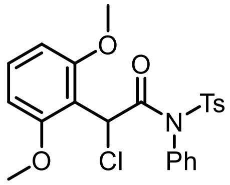

Prepared from **2L** and **1a** according to general procedure E on a 0.1 mmol scale using 30 mol% of Tf_2NH in 63% yield (29.0 mg) as a white solid.

^{1}H NMR (400 MHz, $CDCl_3$): δ 7.97–7.87 (m, 2H), 7.33 (d, *J* = 8.0 Hz, 2H), 7.30–7.25 (m, 1H), 7.17 (t, *J* = 8.4 Hz, 1H), 7.12 (s br, 2H), 6.70 (s br, 2H), 6.30 (d, *J* = 8.4 Hz, 2H), 5.78 (s, 1H), 3.54 (s, 6H), 2.45 (s, 3H);

^{13}C NMR (101 MHz, $CDCl_3$): δ 167.8, 158.0 (2C), 144.9, 136.0, 135.1, 131.3, 129.8 (2C), 129.4 (2C), 129.1, 128.8 (2C), 112.9, 103.4 (2C), 55.6 (2C), 54.4, 21.8; 2C in *ortho* to *N*-Ph not observed;

HRMS (ESI$^+$)**:** exact mass calculated for $[M+Na]^+$ ($C_{23}H_{22}NO_5SClNa^+$) requires *m/z* 482.0799, found *m/z* 482.0798;

IR (thin film): *v* 1730, 1597, 1479, 1363, 1259, 1188, 1108, 695 cm^{-1}.

4aM – 2-(2,6-Dimethoxyphenyl)-*N*-phenyl-2-(pyridin-2-yl)-*N*-tosylacetamide

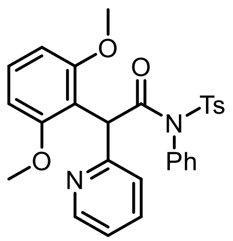

Prepared from **2M** and **1a** according to general procedure E on a 0.05 mmol scale using 120 mol% of Tf_2NH in 45% yield (11.4 mg) as an off-white solid.

^{1}H NMR (400 MHz, $CDCl_3$): δ 8.39–8.34 (m, 2H), 7.87 (d, *J* = 8.3 Hz, 2H), 7.75 (dt, *J* = 8.0, 1.9 Hz, 1H), 7.32–7.26 (m, 2H), 7.37–6.44 (m br, 5H), 7.14 (ddd, *J* = 8.0, 4.8, 0.6 Hz, 1H), 7.10 (t, *J* = 8.3 Hz, 1H), 6.31 (d, *J* = 8.4 Hz, 2H), 5.08 (s, 1H), 3.57 (s, 6H), 2.43 (s, 3H);

^{13}C NMR (101 MHz, $CDCl_3$): δ 172.7, 157.7 (2C), 150.8, 148.3, 144.6, 137.9, 136.6, 135.8, 133.9, 129.5 (2C), 129.4, 129.4 (2C), 128.8, 128.5 (2C), 123.3, 114.4, 103.5 (2C), 55.5 (2C), 45.7, 21.8; 2C in *ortho* to *N*-Ph not observed;

HRMS (ESI$^+$)**:** exact mass calculated for $[M+H]^+$ ($C_{28}H_{27}N_2O_5S^+$) requires *m/z* 503.1635, found *m/z* 503.1635;

IR (thin film): *v* 1712, 1595, 1476, 1360, 1245, 1172, 1154, 1107, 696 cm^{-1}.

9aA – Ethyl 2-(2,6-dimethoxyphenyl)acetate

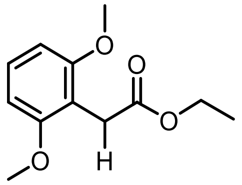

Prepared form **1a** (1.0 equiv) and ethoxyethyne (**6**) (0.14 ml, 50% in toluene, 1.5 equiv) according to General Procedure E on a 0.5 mmol scale in 67% yield (75.5 mg) as a colorless oil.

^{1}H NMR (600 MHz, $CDCl_3$): *δ* 7.20 (t, *J* = 8.3 Hz, 1H), 6.56 (d, *J* = 8.3 Hz, 2H), 4.15 (q, *J* = 7.1 Hz, 2H), 3.80 (s, 6H), 3.69 (s, 2H), 1.25 (t, *J* = 7.1 Hz, 3H);

^{13}C NMR (151 MHz, $CDCl_3$): *δ* 172.3, 158.5 (2C), 128.3, 111.7, 103.7 (2C), 60.4, 55.8 (2C), 29.0, 14.3;

HRMS (ESI$^+$)**:** exact mass calculated for $[M+Na]^+$ ($C_{12}H_{16}O_4Na^+$) requires *m/z* 247.0941, found *m/z* 247.0939;

IR (thin film): *v* 2923, 1734, 1598, 1474, 1258, 1106, 778 cm^{-1}.

10aA – 2-(2,6-Dimethoxyphenyl)-1-(4-methoxyphenyl)ethan-1-one

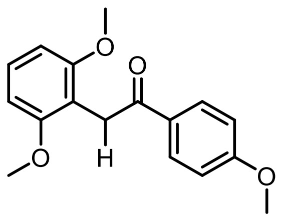

Following an adapted literature procedure:[30] To a solution of **1a** (44.1 mg, 0.22 mmol, 1.1 equiv) and 1-ethynyl-4-methoxybenzene (**7**) (26.4 mg, 0.2 mmol, 1.0 equiv) in nitromethane (0.5 ml) was added *n*-octanethiol (38 µL, 0.22 mmol, 1.1 equiv) and triflic acid (8 µL, 0.09 mmol, 45 mol%). The solution was

stirred at 23 °C for 14 h. Solid $NaHCO_3$ (30 mg) was added, and the reaction was further stirred for 5 min before filtration and evaporation of the solvent. Purification by column chromatography (EtOAc(10%→50%)/heptane) afforded the desired compound in 67% yield (38.3 mg) as a yellow crystalline solid.

^{1}H NMR (400 MHz, $CDCl_3$): δ 8.07–7.97 (m, 2H), 7.21 (t, J = 8.3 Hz, 1H), 6.97–6.90 (m, 2H), 6.57 (d, J = 8.3 Hz, 2H), 4.29 (s, 2H), 3.87 (s, 3H), 3.76 (s, 6H);

^{13}C NMR (101 MHz, $CDCl_3$): δ 196.7, 163.2, 158.6 (2C), 130.7, 130.6 (2C), 128.2, 113.7 (2C), 112.8, 103.9 (2C), 55.9 (2C), 55.6, 33.7;

HRMS (ESI$^+$)**:** exact mass calculated for $[M+Na]^+$ ($C_{17}H_{18}O_4Na^+$) requires *m/z* 309.1097, found *m/z* 309.1098;

IR (thin film): ν 2918, 1673, 1596, 1573, 1474, 1260, 1174, 1099, 1027, 787 cm^{-1}.

11aA – 2-(2,6-Dimethoxyphenyl)-*N*,*N*-dimethyl-4-phenylbutanamide

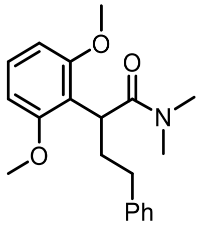

In a flame-dried Schlenk tube, *N*,*N*-dimethyl-4-phenylbutanamide (**8**) (63.1 mg, 0.33 mmol, 1.1 equiv) and 2-fluoropyridine (57 µl, 0.66 mmol, 2.2 equiv) were dissolved in CH_2Cl_2 (3.0 ml) and cooled to 0 °C. Trifluoromethanesulfonic anhydride (56 µl, 0.33 mmol, 1.1 equiv) was added and the reaction was stirred for 15 min at 0 °C. A dry-ice bath was applied and the reaction cooled to –78 °C over the course of 5 min. A solution of **1a** (60.1 mg, 0.3 mmol, 1.0 equiv) and *n*-octanethiol (57 µl, 0.33 mmol, 1.1 equiv) in CH_2Cl_2 (1.5 ml) was added at that temperature and stirred for 30 min. The cooling bath was exchanged for an ice bath and stirring was continued for 30 minutes. Sodium bicarbonate (150 mg) was added and stirred for 5 min at 23 °C. The mixture was filtered and concentrated *in vacuo*. Purification

by column chromatography (EtOAc(50→100%)/heptane) afforded the title compound 59% (58.3 mg) as a colorless oil.

^{1}H NMR (600 MHz, $CDCl_3$): δ 7.22 (t, J = 7.5 Hz, 2H), 7.19–7.14 (m, 3H), 7.12 (t, J = 7.2 Hz, 1H), 6.54 (d, J = 8.3 Hz, 2H), 4.09 (t, J = 6.6 Hz, 1H), 3.76 (s, 6H), 2.90 (s, 3H), 2.73–2.65 (m, 1H), 2.62–2.44 (m, 5H), 1.98–1.86 (m, 1H);

^{13}C NMR (151 MHz, $CDCl_3$): δ 174.0, 158.0 (2C), 143.2, 128.7 (2C), 128.1 (2C), 128.0, 125.5, 117.9, 104.3 (2C), 55.9 (2C), 38.9, 36.7, 36.1, 34.0, 32.7;

HRMS (ESI$^+$)**:** exact mass calculated for $[M+H]^+$ ($C_{20}H_{26}O_3N^+$) requires *m/z* 328.1907, found *m/z* 328.1907;

IR (thin film): ν 2934, 1645, 1591, 1472, 1247, 1229, 1102, 728 cm^{-1}.

4mA – 3-(2-(2,4-Dimethoxyphenyl)hexanoyl)oxazolidin-2-one

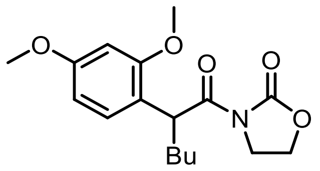

Prepared from **1m** and **2A** according to General Procedure E on a 0.21 mmol scale in 96% yield (64.8 mg) as a colorless crystalline solid.

^{1}H NMR (400 MHz, $CDCl_3$): δ 7.18–7.07 (m, 1H), 6.50–6.41 (m, 2H), 5.22 (t, J = 7.3 Hz, 1H), 4.35 (app td, J = 9.0, 6.4 Hz, 1H), 4.28 (app td, J = 9.0, 7.4 Hz, 1H), 4.08–3.90 (m, 2H), 3.80 (s, 3H), 3.78 (s, 3H), 2.06–1.91 (m, 1H), 1.77–1.66 (m, 1H), 1.40–1.21 (m, 4H), 0.86 (t, J = 7.0 Hz, 3H);

^{13}C NMR (101 MHz, $CDCl_3$): δ 175.3, 159.9, 158.4, 152.9, 128.6, 120.3, 104.4, 98.9, 61.8, 55.8, 55.4, 43.1, 41.8, 32.6, 29.8, 22.8, 14.0;

HRMS (ESI$^+$)**:** exact mass calculated for $[M+Na]^+$ ($C_{17}H_{23}NO_5Na^+$) requires *m/z* 344.1468, found *m/z* 344.1467;

IR (thin film): *v* 2934, 1777, 1694, 1506, 1384, 1208, 1038 cm^{-1}.

4nA – 3-(2-(2,4-Bis(allyloxy)phenyl)hexanoyl)oxazolidin-2-one

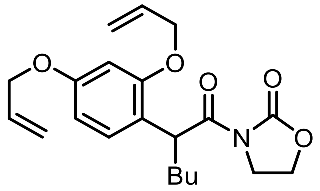

Prepared from **1n** and **2A** according to General Procedure E on a 0.1 mmol scale in 87% yield (32.4 mg) as a colorless oil.

^{1}H NMR (600 MHz, $CDCl_3$): δ 7.14 (d, *J* = 8.2 Hz, 1H), 6.51–6.39 (m, 2H), 6.11–5.98 (m, 2H), 5.41 (ddd, *J* = 17.3, 9.9, 1.5 Hz, 2H), 5.31–5.22 (m, 3H), 4.55–4.44 (m, 4H), 4.36 (app td, *J* = 9.1, 6.2 Hz, 1H), 4.32–4.24 (m, *J* = 8.9 Hz, 1H), 4.04 (ddd, *J* = 10.7, 9.4, 7.6 Hz, 1H), 3.95 (ddd, *J* = 10.9, 9.3, 6.1 Hz, 1H), 2.03–1.92 (m, 1H), 1.81–1.72 (m, 1H), 1.38–1.27 (m, 4H), 0.87 (t, *J* = 7.0 Hz, 3H);

^{13}C NMR (151 MHz, $CDCl_3$): δ 175.5, 158.8, 157.3, 153.0, 133.5, 133.4, 128.7, 120.8, 117.8, 117.3, 105.6, 100.6, 69.3, 69.1, 61.8, 43.1, 41.9, 32.4, 29.8, 22.8, 14.1;

HRMS (ESI$^+$)**:** exact mass calculated for $[M+Na]^+$ ($C_{21}H_{27}NO_5Na^+$) requires *m/z* 396.1781, found *m/z* 396.1785;

IR (thin film): *v* 2929, 1779, 1696, 1610, 1504, 1384, 1184, 1033 cm^{-1}.

4oA – 3-[2-(4-hydroxy-2-methylphenyl)hexanoyl]oxazolidin-2-one

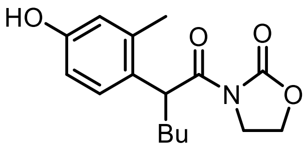

Prepared from **1o** and **2A** according to general procedure E on a 0.2 mmol scale. Analysis of the crude product revealed a mixture of *ipso*- and *ortho*-rearrangement products **4oA** and **4oA'** in a ratio of 83:17 in a combined NMR yield of 68% yield using mesitylene as internal standard. Purification by column chromatography led to mixtures of the two products as a colourless oil in ratios of 7:1 and 1:2.

^{1}H NMR (600 MHz, $CDCl_3$) δ 7.09 (d, *J* = 8.4 Hz, 1H), 6.64 (d, *J* = 2.8 Hz, 1H), 6.60 (dd, *J* = 8.4, 2.8 Hz, 1H), 5.08 (dd, *J* = 8.8, 5.8 Hz, 1H), 4.66 (s, 1H), 4.38 (app td, *J* = 9.1, 6.0 Hz, 1H), 4.30 (app td, *J* = 9.1, 7.5 Hz, 1H), 4.09 (ddd, *J* = 11.0, 9.4, 7.5 Hz, 1H), 3.96 (ddd, *J* = 11.0, 9.3, 6.1 Hz, 1H), 2.38 (s, 3H), 2.08 – 1.99 (m, 1H), 1.64 – 1.57 (m, 1H), 1.39 – 1.28 (m, 3H), 1.26 – 1.22 (m, 1H), 0.87 (t, *J* = 7.1 Hz, 3H);

^{13}C NMR (151 MHz, $CDCl_3$) δ 174.8, 154.4, 153.2, 138.9, 130.1, 128.0, 117.5, 113.0, 61.9, 44.6, 43.2, 34.3, 30.2, 22.8, 19.8, 14.0;

HRMS (ESI$^+$): exact mass calculated for $[M+Na]^+$ ($C_{16}H_{21}NO_4Na^+$) requires *m/z* 314.1363, found *m/z* 314.1361;

IR (thin film) 3415 (br), 2955, 2929, 1780, 1696, 1388, 1222, 1201, 1041 cm^{-1}.

4oA' – 3-{2-[5-hydroxy-3-methyl-2-(methylthio)phenyl]hexanoyl}oxazolidin-2-one

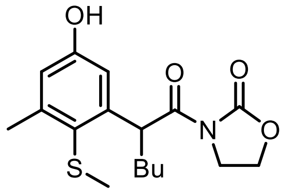

Minor isomer of the reaction above obtained as 2:1 mixture with **4oA**.

^{1}H NMR (600 MHz, $CDCl_3$) δ 6.68 – 6.63 (m, 2H), 5.74 (dd, *J* = 8.0, 6.2 Hz, 1H), 5.38 (s, 1H), 4.44 – 4.32 (m, 2H), 4.13 – 4.05 (m, 1H), 4.05 – 3.94 (m, 1H), 2.52 (s, 3H), 2.22 (s, 3H), 2.07 – 1.97 (m, 1H), 1.72 – 1.65 (m, 1H), 1.37 – 1.28 (m, 4H), 0.88 (t, *J* = 6.8 Hz, 3H);

^{13}C NMR (151 MHz, $CDCl_3$) δ 175.2, 155.9, 152.9, 145.5, 145.2, 127.1, 116.5, 112.4, 61.9, 47.9, 43.2, 34.0, 30.3, 22.8, 21.9, 19.3, 14.1;

HRMS (ESI+): exact mass calculated for $[M+Na]^+$ ($C_{16}H_{21}NO_4Na^+$) requires *m/z* 360.1240, found *m/z* 360.1239;

IR (thin film) 3385 (br), 2929, 1780, 1696, 1386, 1222, 1201, 1040 cm^{-1}.

4pA – 3-[2-(4-hydroxyphenyl)hexanoyl]oxazolidin-2-one

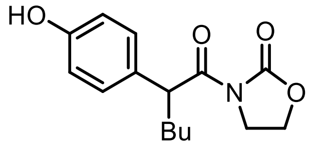

Performed according to general procedure on a 0.14 mmol scale (22.3 mg sulfoxide, 26.3 mg ynamide). Analysis of the crude product revealed a mixture of *ipso*- and *ortho*-rearrangement products **4pA** and **4pA'** in a ratio of 50:50 in a combined NMR yield of 69% yield using mesitylene as internal standard. Purification by column chromatography led to mixtures of the two products as a colourless oil in ratios of 10:1 and 1:1.5.

^{1}H NMR (600 MHz, $CDCl_3$) δ 7.24 (d, *J* = 8.6 Hz, 2H), 6.76 (d, *J* = 8.6 Hz, 2H), 5.08 (s, 1H), 4.93 (t, *J* = 7.6 Hz, 1H), 4.37 (ddd, *J* = 9.4, 8.8, 6.4 Hz, 1H), 4.29 (td, *J* = 9.1, 7.3 Hz, 1H), 4.05 (ddd, *J* = 11.1, 9.5, 7.3 Hz, 1H), 3.92 (ddd, *J* = 11.0, 9.3, 6.3 Hz, 1H), 2.08 – 1.94 (m, 1H), 1.80 – 1.71 (m, 1H), 1.36 – 1.13 (m, 4H), 0.85 (t, *J* = 7.3 Hz, 3H);

^{13}C NMR (151 MHz, $CDCl_3$) δ 174.8, 155.0, 153.3, 131.1, 130.0 (2C), 115.5 (2C), 61.8, 47.6, 43.0, 33.9, 29.7, 22.7, 14.0;

HRMS (ESI+): exact mass calculated for $[M+Na]^+$ ($C_{15}H_{19}NO_4Na^+$) requires *m/z* 300.1206, found *m/z* 300.1201;

IR (thin film) 3396 (br), 2926, 1778, 1697, 1514, 1387, 1266, 1219, 1042, 740 cm^{-1}.

4pA' – 3-(2-(5-hydroxy-2-(methylthio)phenyl)hexanoyl)oxazolidin-2-one

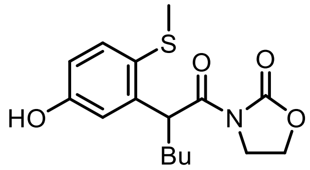

Isomer of the reaction above obtained as 1.5:1 mixture with **4pA**.

^{1}H NMR (600 MHz, $CDCl_3$) δ 7.29 (d, *J* = 8.5 Hz, 1H), 6.78 (d, *J* = 2.8 Hz, 1H), 6.72 (dd, *J* = 8.4, 2.8 Hz, 1H), 5.67 (s, 1H), 5.47 (dd, *J* = 8.1, 6.2 Hz, 1H), 4.41 – 4.31 (m, 2H), 4.09 – 3.95 (m, 2H), 2.40 (s, 3H), 2.04 – 1.97 (m, 1H), 1.81 – 1.62 (m, 1H), 1.38 – 1.30 (m, 4H), 0.87 (t, *J* = 7.2 Hz, 3H);

^{13}C NMR (151 MHz, $CDCl_3$) δ 174.7, 155.0, 152.9, 141.1, 132.8, 128.7, 115.2, 114.6, 62.0, 46.7, 43.1, 33.7, 30.1, 22.8, 19.2, 14.1;

HRMS (ESI+): exact mass calculated for $[M+Na]^+$ ($C_{16}H_{21}NO_4SNa^+$) requires *m/z* 346.1083, found *m/z* 346.1074;

IR (thin film) 3393 (br), 2927, 1779, 1697, 1475, 1387, 1220, 1041, 759 cm^{-1}.

6.1. Limitations and unsuccessful substrates

As discussed in the manuscript, the redirection of [3,3]-sigmatropic rearrangement to [2,3]-*ipso* rearrangement relies on electron redistribution in the sulfoxide partner. Indeed, sulfoxides bearing a halide substituent (**S6a**–**S6d**) only underwent [3,3]-sigmatropic rearrangement (**Fig. S 1**), which is in line with our previous reports.[11,30] Interestingly, *ortho*-iodo aryl sulfoxide **S6d** also gave product of sulfoxide reduction. Similarly, naphthalene sulfoxide **S6e** produced only the product of [3,3]-rearrangement and sulfoxides **S6f** and **S6g**, bearing alkyl groups, did not follow a [2,3]-rearrangement pathway. Surprisingly, with extremely sterically congested tri(*tert*-butyl)benzenesulfoxide **S6h**, we could detect the formation of an α-thioether instead of the expected arylation product.

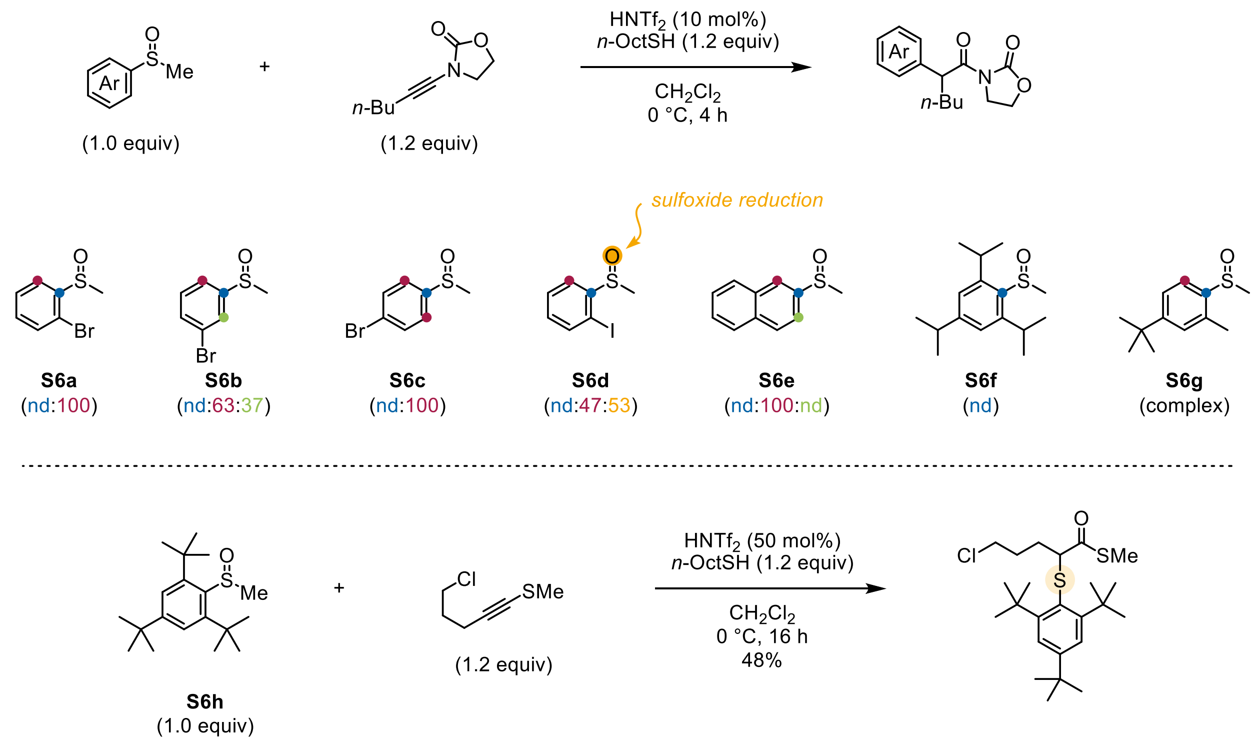

Fig. S 1: Limitation and unsuccessful substrates.

6.2. Reaction of chiral ynamide with racemic sulfoxide

The ynamide **2N** was prepared according to the literature.[14]

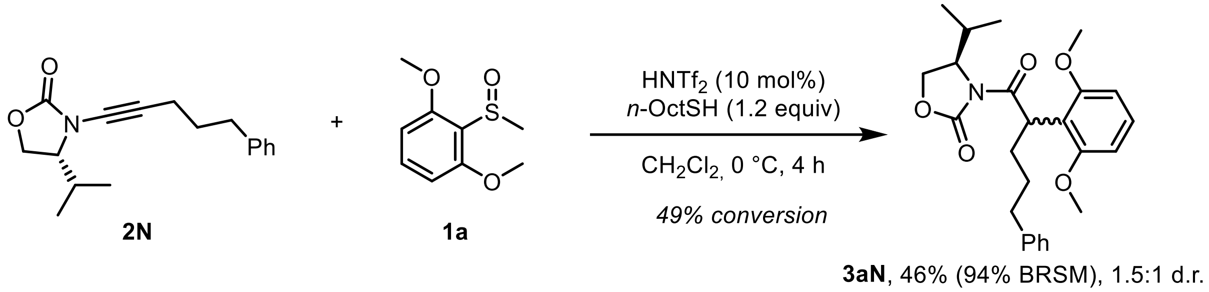

The reaction was conducted on 0.2 mmol scale using **1a** and **2A** according to General Procedure E. Analysis of the crude material revealed a clean reaction profile, showing 46% NMR yield (49% conversion of sulfoxide) with a 1.5:1 ratio of diastereomers. H_{Ar} Signals were used to determine the d.r. ratio as a small signal was overlapping in the region of the hydrogens attached to the stereogenic carbon (see below). The observed diasteroselectivity is consistent with a low selectivity of addition of either enantiomer of the sulfoxide to the chiral keteniminium ion, followed by a matched-mismatched scenario during the [2,3]-rearrangement process.

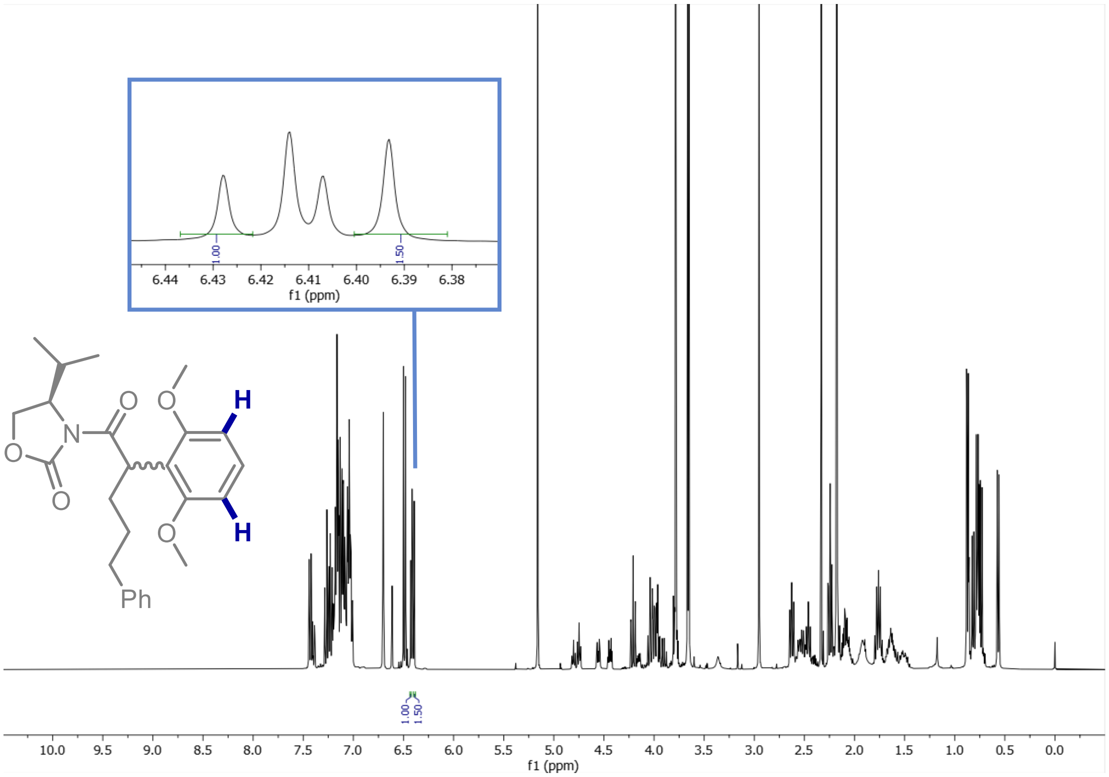

7. Temperature dependent NMR study

We observed that some of the sterically hindered α-aryl carbonyl compounds showed NMR spectra which are characteristic of hindered rotation around the newly formed C–C bond. In spectra measured at 25 °C, two methoxy signals of **5aC** were observed at 3.89 ppm and 3.79 ppm, respectively, and two separate *i*-Pr septets for **5hA** were found at 3.23 ppm and 2.78 ppm, respectively. To determine the energy barrier for their bond rotation, we performed temperature dependent NMR studies.

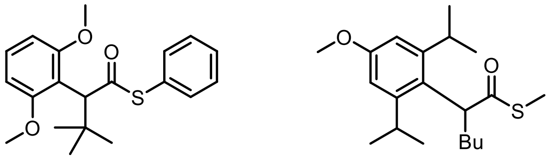

5aC **5hA**

Starting with **5aC**, the spectra at various temperatures are shown in **Fig. S 2**. A clear coalescence is seen at 75–85 °C. Focusing on the protons of the methoxy groups H[b], and a coalescence temperature of T_c = 85 °C, we used the following two equations:

$$k_{ex} = \frac{\pi}{\sqrt{2}}\Delta\nu_0$$

where $\Delta\nu_0$ is the frequency difference at 25 °C and k_{ex} is the rate of exchange at coalescence temperature; as well as Eyring's equation:

$$k_{ex} = \frac{k_B T_c}{h}\,\exp\left\{\frac{-\Delta G^{\neq}}{RT_c}\right\}$$

$$\Delta G^{\neq} = -RT_c\,\ln\left\{k_{ex}\frac{h}{k_B\,T_c}\right\} = -8.31\times 358\times\ln\left\{118\times\frac{4.8\times 10^{-11}}{358}\right\} \approx 18\ \mathrm{kcal/mol}$$

We can calculate the activation energy of bond ration of *ca* 18 kcal/mol.

Line-shape analysis (using Dynamic NMR (DNMR) module for TopSpin) for each spectrum allowed us to estimate k_{ex} for each temperature (simulated spectra **Fig. S C** and **E**) and plotting ln(k/k_0) *vs* 1/*T* allowed us to deduce the value of the activation energy (see **Fig. S 3**). When measured on the methoxy protons (H[b]), a value of 18.4 kcal/mol was found, and when measured on the aromatic protons (H[a]), a value of 18.8 kcal/mol was found.

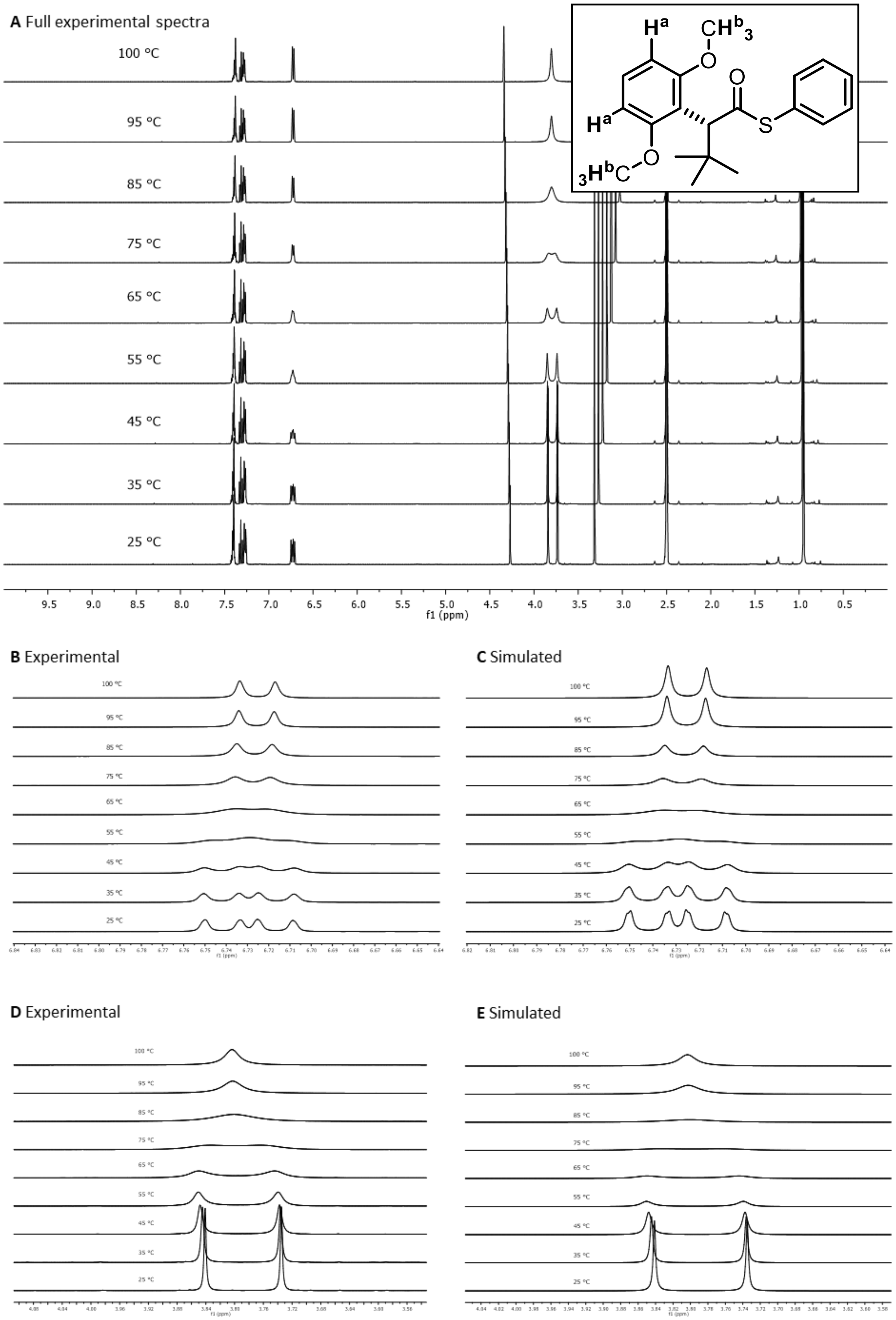

Fig. S 2: Temperature dependent ^{1}H NMR (DMSO-d$_6$, 500 MHz) spectra of 5aB. A, full experimental spectra; **B**, zoom on the *meta* aromatic proton signals (H^a); **C**, simulated spectra for the *meta* aromatic proton signals; **D**, zoom on the methoxy proton signals (H^b); **E**, simulated spectra for the methoxy proton signals.

Entry	T (K)	*k* (Hz)
1	298	1.15
2	308	3.32
3	318	9.41
4	328	22.0
5	338	50.4
6	348	113
7	358	262
8	368	521
9	373	647

Table S 4: Values of k computed for simulation of spectra for *meta* aromatic protons of **5aC** (H^a), see **Fig. S** for proton labelling.

Entry	T (K)	*k* (Hz)
1	298	1.11
2	308	3.53
3	318	9.40
4	328	22.59
5	338	50.2
6	348	106
7	358	238
8	368	440
9	373	619

Table S 5: Values of k computed for simulation of spectra for methoxy protons of **5aC** (H^b), see **Fig. S** for proton labelling.

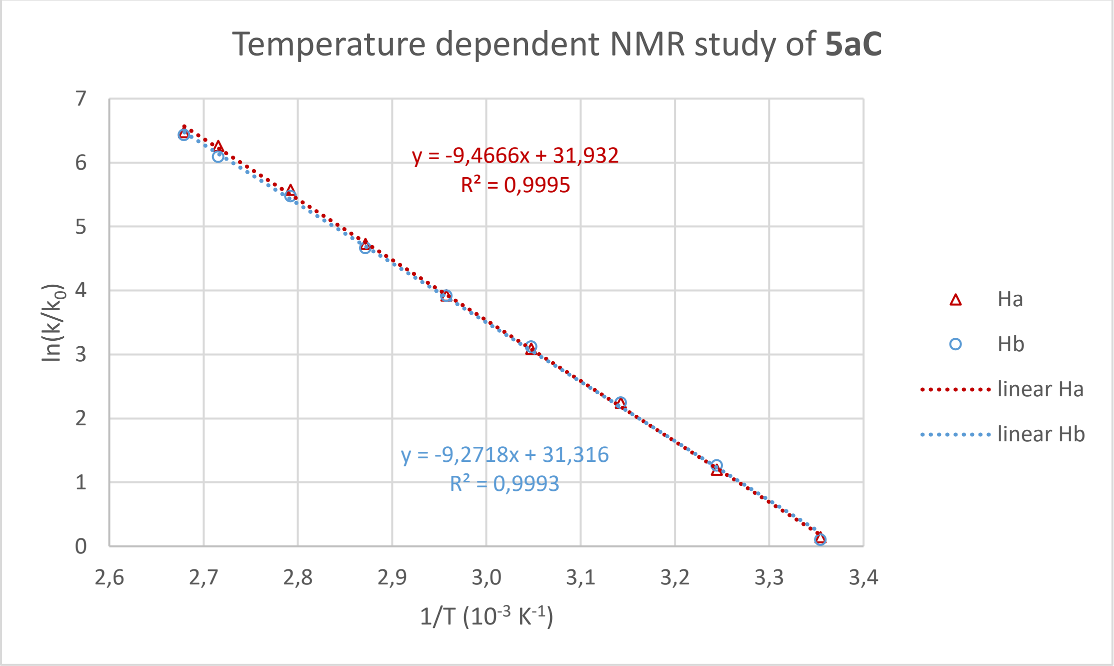

Fig. S 3: Plot of $\ln(k/k_0)$ vs 1/T for compound **5aC**.

This slow rotation around this newly formed C–C bond in **5aC** can also be observed on NOESY spectrum (**Fig. S**), in which the two methoxy groups are shown to undergo chemical exchange during the mixing time.

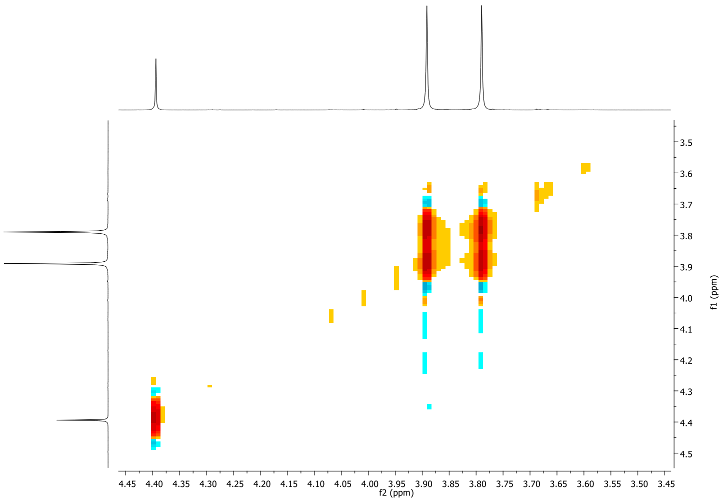

Fig. S 4: NOESY spectrum of 5aC ($CDCl_3$, 600 MHz) showing a chemical exchange between the two methoxy protons (H^b)

The same analysis was performed on **5hA** (**Fig. S** and **S 6**). Its activation energy of bond rotation was calculated to be *ca.* 17 kcal/mol and line-shape analysis gave a value of 17.0 kcal/mol when calculated using aromatic proton signals (H^a), and 15.8 kcal/mol when calculated on the *i*-Pr protons (H^b). Line-shape analysis of H^b signals is slightly hindered by the water and the solvent residual peaks.

Again, this slow chemical exchange can also be observed on the NOESY spectrum of **5hA** (**Fig. S**).

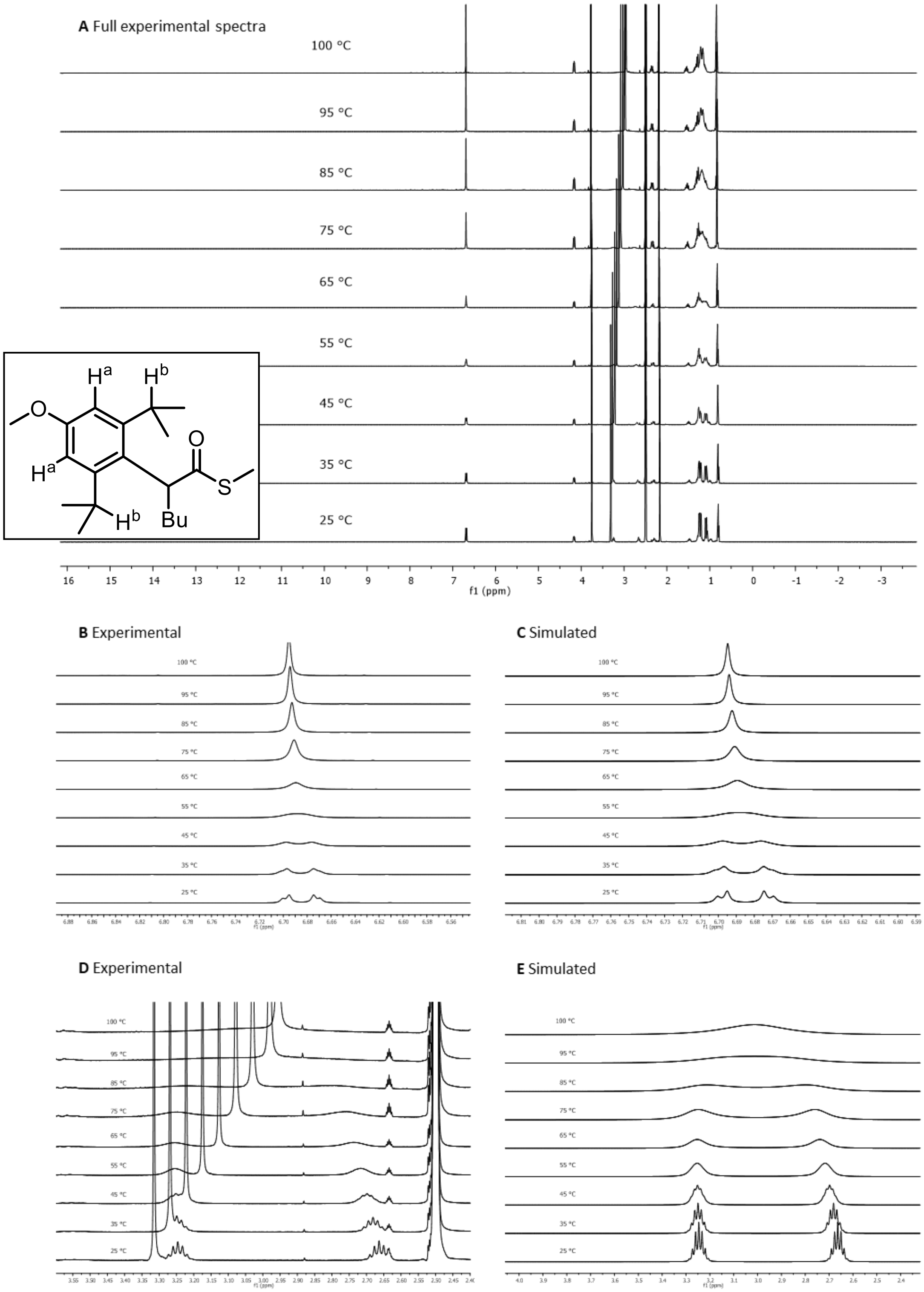

Fig. S 5: Temperature dependent ^{1}H NMR (DMSO-d$_6$, 500 MHz) spectra of 5eA. A, full experimental spectra; **B**, zoom on the *meta* aromatic proton signals (H^a); **C**, simulated spectra for the *meta* aromatic proton signals; **D**, zoom on the *i*-Pr proton signals (H^b); **E**, simulated spectra for the *i*-Pr proton signals.

Entry	T (K)	*k* (Hz)
1	298	3.34
2	308	6.85
3	318	18.2
4	328	40.7
5	338	89.0
6	348	187
7	358	370
8	368	670
9	373	1010

Table S 6: Values of *k* computed for simulation of spectra for *meta* aromatic protons of **5hA** (H^a), see **Fig. S** for proton labelling.

Entry	T (K)	*k* (Hz)
1	298	5.00
2	308	8.68
3	318	17.0
4	328	50.5
5	338	94.9
6	348	180
7	358	369
8	368	630
9	373	927

Table S 7: Values of *k* computed for simulation of spectra for *i*-Pr protons of **5hA** (H^b), see **Fig. S** for proton labelling.

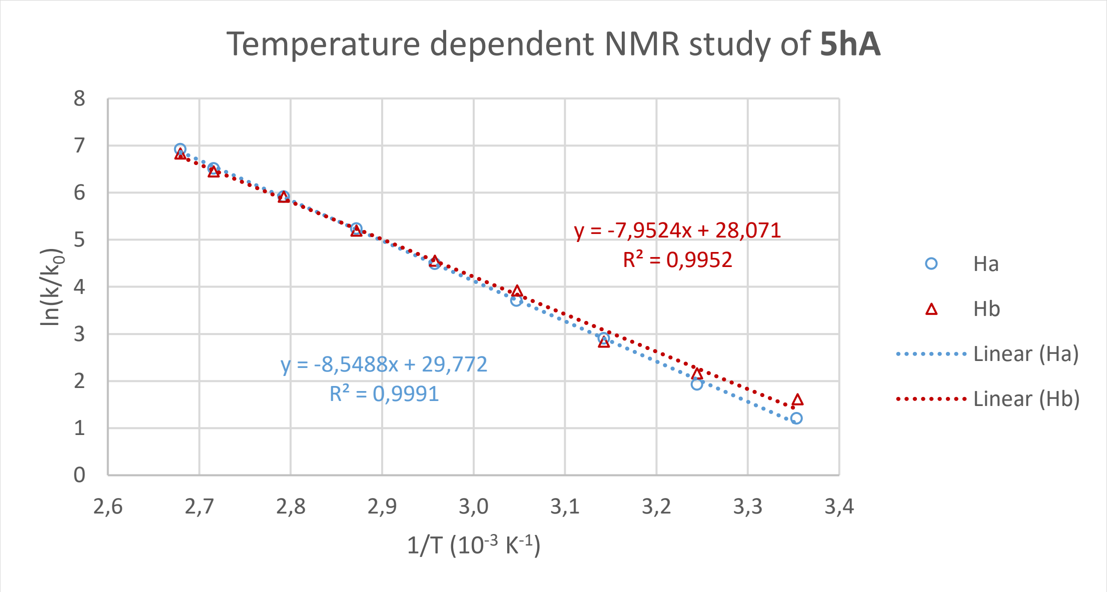

Fig. S 6: Plot of $\ln(k/k_0)$ vs 1/T for compound **5hA**.

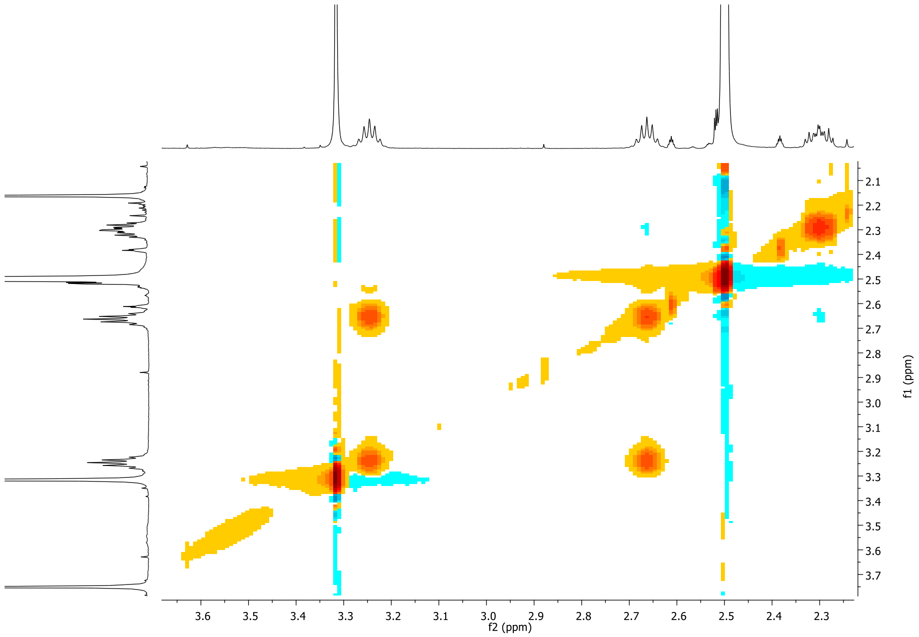

Fig. S 7: NOESY spectrum of 5hA (DMSO-d$_6$, 600 MHz) showing a chemical exchange between the two *i*-Pr protons (H^b).

8. Computational Study

Computational details

The conformational space of all molecules was initially searched using meta-dynamics simulations based on generic force field GFN-FF[31] calculations as implemented in Conformer-Rotamer Ensemble Sampling Tool CREST.[32,33]

The structures located with the CREST were then subjected to RI-MP2/def2-SVP geometry optimisation.[34–38] The nature of all stationary points (minima and transition states) was verified through the computation of the vibrational frequencies. The thermal corrections to the Gibbs free energy were combined with the single-point energies calculated at the RI-MP2/def2-QZVP to yield RI-MP2/def2-QZVP//RI-MP2/def2-SVP Gibbs free energies ("G_{273}") at 273.15 K.

A conductor-like screening model COSMO[39] was applied to consider solvent effects for single-point energy calculations. Free energies in solution were corrected to a reference state of 1 mol l^{-1} at 273.15 K through the addition of $RT\ln(22.41) = +1.69$ kcal mol^{-1} to the gas phase (1 atm) free energies.

The auxiliary calculations, including relaxed potential energy scans and frontier molecular orbital analysis, were conducted using density functional theory (DFT) at the following levels: PBE0-D3(BJ), SMD(DCM)/def2-SVP and PBE0-D3(BJ),CPCM(DCM)/def2-TZVP//PBE0-D3(BJ),SMD(DCM)/def2-SVP.[40-43]

The RI-MP2 calculations were performed with the Turbomole V7.2 program package.[44] The DFT calculations have been performed with the ORCA 5.0.1 and Gaussian16 program packages.[45,46]

[2,3]- and [3,3]-pathways in comparison, chirality transfer and comment on the mechanism

When comparing [2,3] and [3,3]-rearrangement pathways, it is worth noting that the calculations deny a direct [3,3]-sigmatropic rearrangement starting from the intermediate **B** (**Fig. S 8**). Alternatively, after the [2,3]-rearrangement, instead of rearomatization, the intermediate **C** could undergo an alkyl shift reaction via transition state $\mathbf{TS_{C\text{-}E}}$ leading to product **E**, which is formally the product of a [3,3]-

sigmatropic rearrangement. However, the alkyl shift event **C**$\rightarrow$**E** is both thermodynamically and kinetically less favorable than the **C**$\rightarrow$**D** step. In fact, in case of the product **D**: ΔG(**C**$\rightarrow$**D**) = −6.4 kcal mol^{-1} and $\Delta G^{\ddagger}$(**C**$\rightarrow$**D**) = 5.1 kcal mol^{-1}, while for the formation of the product **E**: ΔG(**C**$\rightarrow$**E**) = 18.1 kcal mol^{-1} (endergonic reaction) and $\Delta G^{\ddagger}$(**C**$\rightarrow$**E**) = 25.0 kcal mol^{-1}. Thus, calculations indeed favor the formation of [2,3]-rearrangement product over the [3,3]-rearrangement product, in agreement with the experimental evidence.

Also worth of note is the fact that, owing to the involvement of an intermediate showing S−O bond cleavage (intermediate I), this process is strictly speaking not pericyclic and thus does not conform to the definition of a “sigmatropic rearrangement” in *stricto sensu*.

In silico studies: Gibbs free energy profile

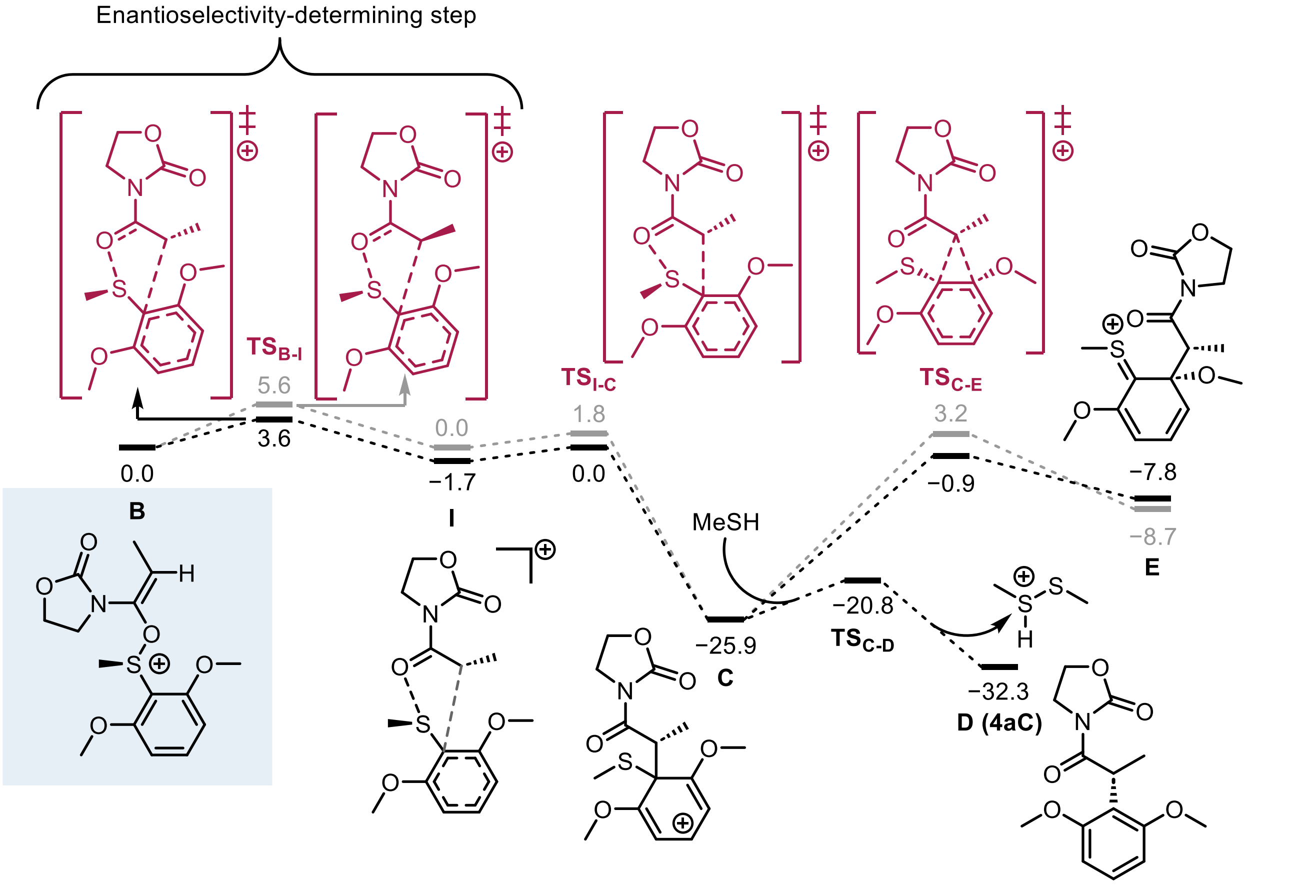

Fig. S 8: Computed energy profile of the ipso-rearrangement. The computed Gibbs free energy profile (RI-MP2-COSMO/def2-QZVP//RI-MP2/def2-SVP, ($\Delta G_{273,DCM}$) for the conversion of intermediate **B** (taken as a reference, 0.0 kcal mol^{-1}) into the [2,3]-rearrangement intermediate **C** and the formal [3,3]-rearrangement intermediate **E**. The black and the grey lines show diastereomeric pathways. The transition states are depicted with burgundy color.

Diastereomeric pathways are possible for the stepwise 2,3-rearrangement (as well as for the alkyl shift leading to the 3,3-product **E**), as shown in **Fig. S 8** with black and red lines. The energetic gap between the computed diastereomeric pathways reveals the mechanism of the experimentally observed chirality transfer. Indeed, $\Delta\Delta G^{‡}$ computed for the **TS$_{B-I_R}$** and **TS$_{B-I_S}$** is 2.0 kcal mol^{-1}, which corresponds to the 90% theoretical value of enantiomeric excess (*ee* = 100%(*er*-1/*er*+1), and *er* = e$^{\Delta\Delta G‡/RT}$).

Potential energy surface scan from intermediate B to the formal [3,3]-rearrangement intermediate E

Fig. S 9 presents two relaxed potential energy surface scans at the DFT level of theory (PBE0-D3(BJ), SMD(DCM)/def2-SVP) describing the path from the intermediate **B** to the formal [3,3]-rearrangement intermediate **E**, aimed at probing the feasibility of the [3,3]-sigmatropic rearrangement. Scan 1 (red line) corresponds to the systematic shortening of the C1-C2 bond of intermediate **B** in 0.02 Å increments. Scan 2 (blue line) attempts to block the *ipso*-rearrangement by, in addition to shortening the C1-C2 bond, constraining the dihedral angle C1-C3-C4-S.

The energy profile from Scan 1 (red) clearly supports an *ipso*-rearrangement mechanism. As the C1–C2 bond shortens, the energy first increases smoothly, passes through a shallow transition-state-like region ($\mathbf{TS_{B–C}}$), and then drops steeply to a well-defined intermediate **C** (resulting from [2,3]-rearrangement). This species can subsequently undergo an alkyl shift via transition state $\mathbf{TS_{C–E}}$, ultimately forming the formal [3,3]-rearrangement product **E**. Notably, the transient intermediate **I**, observed at the RI-MP2 level, was not located along this DFT-based scan. This absence likely reflects the limitations of DFT in capturing shallow or weakly stabilized intermediates within flat regions of the PES, a known issue for complex systems where dispersion and dynamic correlation are critical. These limitations justify our use of wave function-based RI-MP2 calculations, which were essential in revealing the full mechanistic landscape discussed in our study.

Scan 2 demonstrates that constraining the dihedral angle to prevent the *ipso*-rearrangement leads to a steep increase in the barrier, with apparent stabilization occurring only through a sudden jump to a structure resembling the transient intermediate **I**, which must form to allow energy relaxation. Attempts to relax the highest-energy structure, specifically to locate the [3,3]-path transition state, led to the same outcome as before: the transition state is not localizable.

Taken together, these results reinforce our conclusions that the reaction proceeds with high regioselectivity via the [2,3]-pathway, while the [3,3]-rearrangement is unfavorable.

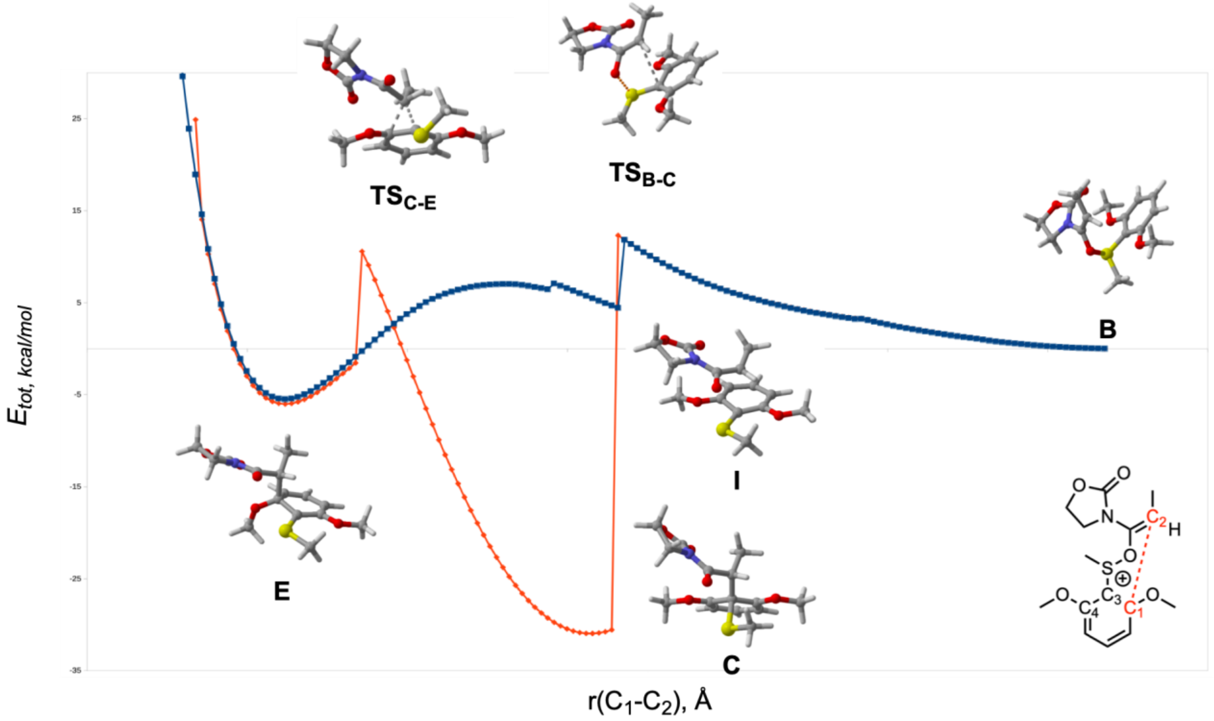

Fig. S 9: Relaxed potential energy surface scans along the C1–C2 bond in intermediate **B**. Calculations were performed at the PBE0-D3(BJ), SMD(DCM)/def2-SVP level of theory. The curves show the relative total energy difference, $E_{tot,}$ with respect to the intermediate **B**, taken as a reference, 0.0 kcal mol^{-1}. The red line corresponds to Scan1, involving only systematic shortening of the C1-C2 bond, while the blue line corresponds to Scan2, in which the C1-C2 was shortened while constraining the dihedral angle C1-C3-C4-S.

Comparison of HOMO orbitals in selected sulfoxides

The highest occupied molecular orbitals (HOMOs) of the sulfoxides can serve as a qualitative descriptor for predicting regioselectivity. As shown in **Table S 8**, the HOMOs of the 2,6-dimethoxyphenyl sulfoxide and of the 2,4-dimethoxy-substituted compound display negligible orbital amplitude at the C_6 position, consistent with the suppression of a [3,3]-sigmatropic rearrangement pathway. In contrast, the 2-isopropyl-4-methoxy analog shows increased HOMO density at C_6, particularly in conformation Conf2, which is only 0.6 kcal/mol higher in energy than the global minimum Conf1. This electronic distribution suggests that a [3,3]-rearrangement may be accessible in this system, in agreement with experimental observations.

Table S 8. DFT-optimized structures and HOMOs of three representative Sulfoxides

System	Conformation, relative energy (kcal/mol)[a]	Optimized structure[a]	HOMO[a]
2,6-dimethoxyphenyl sulfoxide	Conf1, 0.0	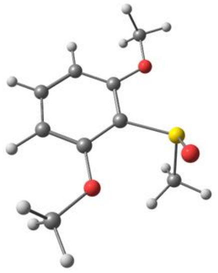	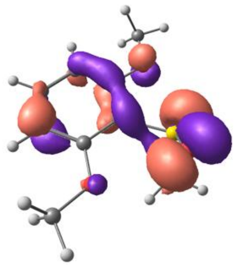
2,4-dimethoxyphenyl sulfoxide	Conf1, 0.0	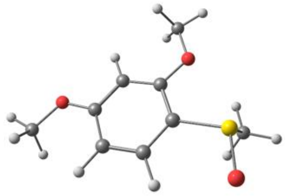	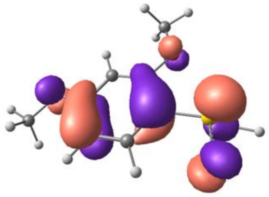
2-isopropyl-4-methoxyphenyl sulfoxide	Conf1, 0.0	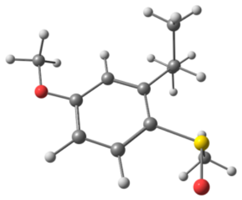	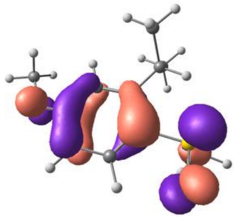
	Conf2, 0.6	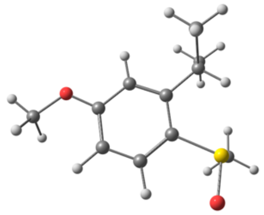	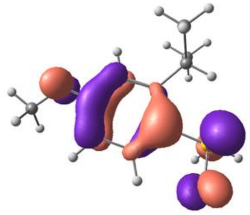

[a]PBE0-D3(BJ),CPCM(DCM)/def2-TZVP//PBE0-D3(BJ),SMD(DCM)/def2-SVP.

[2,3]- *vs* [3,3]-Rearrangement in the mono-methoxy-substituted system

The [3,3]-rearrangement reported in our previous work (*Chem. Sci.* **2018**, *9*, 4124) proceeded toward the carbon atom lacking a methoxy substituent, since that study focused on mono-methoxy-substituted sulfoxides. In the present system, however, this pathway is usually blocked because both carbon atoms that would potentially be available for the [3,3]-rearrangement bear methoxy substituents. As we demonstrate, the [3,3]-rearrangement can therefore occur only with the formation of the intermediate **E** (see **Fig. S 9**), but this pathway is energetically much less favorable than the [2,3]-pathway.

This raises the question of why, in the case of mono-methoxy-substituted sulfoxides, the analogous pathway ultimately leading to the *meta*-arylated product was observed. We believe the explanation lies in the relative energies of the *ipso*-intermediate **C** and the **E**-like intermediates (**E** in the present study and **I** in *Chem. Sci*. **2018**, *9*, 4124). To clarify this, we performed additional computations (PBE0-D3(BJ),SMD(DCM)/def2-SVP), including thorough conformational exploration, to compare the thermodynamic properties of these intermediates in both systems. The results, summarized in the table below, show that the likelihood of forming both intermediates is indeed much higher for the mono-methoxy case than for the di-methoxy case, consistent with the experimental observations.

Thus, both the *ipso*-pathway and the *meta*-arylation via the **E**-like intermediate (**I**) are feasible for the mono-methoxy system, whereas only the *ipso*-pathway is operative in the di-methoxy-substituted sulfoxides.

System	Δ*G* = *G*(**E**-like)-*G*(*ipso*, **C**), kcal/mol
Mono-methoxy-substituted sulfoxide (*Chem. Sci.* **2018**, *9*, 4124)	10.3
Di-methoxy-substituted sulfoxides (this study)	22.4

Cartesian coordinates of the most stable ($\Delta G_{273,DCM}$) conformations as computed at the RI-MP2-COSMO/def2-QZVP//RI-MP2/def2-SVP level of theory

B

C	2.1906287	-2.1274242	-1.4677046
C	1.0787640	-2.5744773	-0.7552506
C	0.5362978	-1.7596883	0.2447299
C	1.2151258	-0.5553286	0.5894651
C	2.3089446	-0.0819809	-0.1883815
C	2.8228881	-0.9100781	-1.1902044
H	3.6761803	-0.5991354	-1.7905580
O	2.7992775	1.1296183	0.1295559
C	3.9059363	1.6267917	-0.6216548
H	4.7845380	0.9772401	-0.5022887
H	4.1222860	2.6188254	-0.2137625
H	3.6455504	1.7138831	-1.6860766
S	0.4129711	0.3957527	1.7883235
O	-0.0140093	1.8495303	0.9916469
C	-0.6662130	1.6146393	-0.2154784
C	-0.1013878	2.0448032	-1.3606045
C	-0.7751377	2.0164350	-2.6908921
H	-1.8663481	1.9631640	-2.5826328
H	-0.5225136	2.9147261	-3.2698977
H	-0.4566573	1.1325013	-3.2643560
H	0.9072817	2.4522612	-1.2621548
N	-1.8552938	0.9049608	-0.0995545
C	-2.1052251	-0.2769817	-0.8028666
O	-1.3920279	-0.8087373	-1.6131664
O	-3.3016707	-0.7531082	-0.3920150
C	-3.9803320	0.2409555	0.3862152
C	-2.8622927	1.1337188	0.9263044
H	-2.5133768	0.8030997	1.9186085
H	-3.1530321	2.1913678	0.9802499
H	-4.6620467	0.7994592	-0.2720374
H	-4.5582207	-0.2640400	1.1680703
C	1.6545909	1.1376980	2.8387706
H	2.4173093	1.6209340	2.2250691
H	1.1136409	1.8534312	3.4707267
H	2.0646325	0.3232496	3.4504500
O	-0.5663481	-2.0421565	0.9509703
C	-1.3132266	-3.2107452	0.5990535
H	-1.6260605	-3.1577263	-0.4515840
H	-2.1934222	-3.2031631	1.2479220
H	-0.7203419	-4.1166634	0.7872852
H	0.6017263	-3.5134960	-1.0291692
H	2.5921242	-2.7567358	-2.2655757

TS_B-I_RS

C 1.8294955 2.6205359 0.7894309

C 2.6507214 1.4896706 0.8518459

C 2.3104102 0.3501635 0.1174097

C 1.1884270 0.4116496 -0.7648680

C 0.3178876 1.5482324 -0.7489846

C 0.6918002 2.6773685 -0.0155932

H 2.0970743 3.4969768 1.3846532

H 3.5183198 1.4918388 1.5095782

H 0.0631913 3.5655401 -0.0025001

S 0.5908314 -0.9033046 -1.6799642

C 1.9446914 -1.9702599 -2.1769407

H 2.7064689 -1.3502843 -2.6652199

H 1.5006242 -2.6682176 -2.8977381

H 2.3416912 -2.4866131 -1.3010414

O 0.0221594 -2.0791542 -0.3512883

C -0.5388668 -1.4350301 0.6905651

C 0.1788822 -1.2593342 1.8372027

N -1.8378783 -0.9756485 0.4852409

C -2.7776372 -1.6186409 -0.4230220

C -4.0102384 -0.7587365 -0.1558716

O -3.4741927 0.5077817 0.2496048

C -2.2341545 0.3347683 0.7583306

H -4.6417366 -0.6032082 -1.0374890

H -4.6181732 -1.1646332 0.6661772

H -2.4395856 -1.5435016 -1.4689289

H -2.9301353 -2.6756647 -0.1683886

O -1.5986138 1.1902844 1.3203111

O -0.7882761 1.4498119 -1.4940297

O 3.0117817 -0.7943371 0.1179956

C -1.6539556 2.5873286 -1.5815174

H -2.4853228 2.2744310 -2.2192681

H -1.1256638 3.4327517 -2.0438449

H -2.0294523 2.8553089 -0.5861093

C 4.1847137 -0.8697500 0.9279712

H 4.9227033 -0.1173259 0.6165299

H 3.9346959 -0.7340766 1.9899012

H 4.5917167 -1.8733235 0.7716220

C -0.3762325 -0.8025551 3.1396983

H -0.1722238 0.2697021 3.2873646

H -1.4651272 -0.9346851 3.1781714

H 0.0819097 -1.3609223 3.9674361

H 1.2227594 -1.5738775 1.7685575

TS_B-I_RR

C 1.6521819 2.6976920 1.2771632

C 2.1955604 1.6468627 0.5367273

C 1.3923978 0.9324490 -0.3582476

C	0.0567361	1.3846433	-0.5836814
C	-0.4926473	2.4379301	0.2197429
C	0.3292689	3.1206935	1.1181146
H	2.2903553	3.2192012	1.9946559
H	3.2234013	1.3396315	0.7185727
H	-0.0593077	3.9368462	1.7251930
S	-1.1032633	0.7017613	-1.6421285
C	-0.2947697	-0.4185952	-2.7847723
H	-1.1165600	-0.7908333	-3.4103134
H	0.2152565	-1.2219352	-2.2539228
H	0.4148974	0.1591976	-3.3903597
O	-1.9594547	-0.5693352	-0.5768652
C	-1.3181629	-0.8806126	0.5669694
C	-1.5202964	-0.1290658	1.6896085
N	-0.4705158	-1.9886153	0.5069549
C	-0.7641987	-3.1832619	-0.2703763
C	0.3645408	-4.0921269	0.2176900
O	1.3913676	-3.1881419	0.6457970
C	0.8390418	-1.9995860	0.9785157
H	0.0507359	-4.7017065	1.0778608
H	0.7765365	-4.7439575	-0.5605919
H	-1.7616119	-3.5801883	-0.0381621
H	-0.7071443	-2.9919555	-1.3537158
O	1.4160484	-1.1168936	1.5626562
O	-1.7886274	2.7042643	0.0005612
O	1.8205328	-0.1009029	-1.0911680
C	-2.3763331	3.8311543	0.6504969
H	-2.3715201	3.6967683	1.7416118
H	-3.4077506	3.8771724	0.2894068
H	-1.8448581	4.7545714	0.3813602
C	3.1787166	-0.5306823	-0.9394604
H	3.8683597	0.2697247	-1.2416463
H	3.3634524	-0.8399941	0.0964471
H	3.2909722	-1.3888244	-1.6089628
C	-1.0484602	-0.4665909	3.0587811
H	-1.7983063	-0.1667482	3.8032801
H	-0.8522864	-1.5416854	3.1614591
H	-0.1062964	0.0590009	3.2820098
H	-2.1473097	0.7520002	1.5340322

I_RS

O	-1.9041605	-0.0498604	1.2824555
C	0.9595526	0.5182210	1.3783219
C	1.1114894	1.9540938	1.4854841
C	1.5410207	-0.1520968	0.2345580
C	1.9621291	2.6217167	0.5997208
C	2.3782520	0.5435586	-0.6339535
C	2.5474775	1.9222907	-0.4605000

H	2.8294904	0.0425592	-1.4882653
C	-0.5146359	0.3775118	3.8353776
S	0.1505165	-0.5363161	2.4317057
C	-1.0193099	1.5423050	-0.1518554
C	-1.5340730	0.2459930	0.1009891
H	-1.1388311	2.1679760	0.7370616
C	-0.7006173	2.2073474	-1.4311327
H	0.2876041	0.8706418	4.3924304
H	-1.2702119	1.0871179	3.4901830
H	-0.9769936	-0.4083253	4.4466158
H	2.1208526	3.6953991	0.6870306
N	-1.6471015	-0.7588253	-0.8645600
C	-2.3652447	-1.9825727	-0.5199794
H	-1.8523542	-2.5136302	0.2962197
H	-3.3962344	-1.7695568	-0.2120426
C	-2.2657741	-2.7187351	-1.8479708
H	-3.0919173	-2.4597135	-2.5262434
C	-0.7564913	-1.0166775	-1.9135841
O	-1.0381446	-2.2342936	-2.4124466
H	-2.1961386	-3.8075596	-1.7509270
O	0.1468650	-0.3208928	-2.3056958
H	0.3818022	2.1095585	-1.6562415
H	-1.2256194	1.7573355	-2.2823417
H	-0.9325077	3.2796675	-1.3653791
H	3.1903006	2.4656639	-1.1580417
O	1.2720714	-1.4561827	0.1621192
C	2.0366403	-2.2664659	-0.7439770
H	3.1059473	-2.1861763	-0.5038217
H	1.6913524	-3.2912845	-0.5801212
H	1.8446875	-1.9664031	-1.7810198
O	0.5022754	2.5316177	2.5216723
C	0.6481846	3.9419207	2.7081485
H	0.0473816	4.1868620	3.5888949
H	1.6999738	4.2018124	2.8905282
H	0.2680642	4.4900199	1.8344379

I_RR

C	1.6072867	2.7391143	0.1066576
C	0.5625314	2.2913213	0.9275170
C	0.3839908	0.9264633	1.1474879
C	1.3612930	-0.0050329	0.6374358
C	2.3967658	0.4948551	-0.2526763
C	2.5454680	1.8690938	-0.4537398
H	1.7120643	3.8131421	-0.0682915
H	-0.1565541	3.0082594	1.3191815
H	3.3401110	2.2587364	-1.0883853
S	1.4555145	-1.6726689	0.9219683
C	0.2674753	-2.1473592	2.1940690

H	0.4764250	-3.2152528	2.3393649
H	-0.7578897	-2.0004370	1.8511024
H	0.4547141	-1.5958274	3.1209658
O	-0.0553156	-2.0599871	-0.9321007
C	-0.5577501	-0.9234600	-1.2026589
C	0.2518938	0.0546599	-1.8350535
N	-1.8981655	-0.7144678	-0.8513067
C	-2.7291466	-1.8544647	-0.4866215
C	-4.0796808	-1.1606030	-0.3727703
O	-3.7402413	0.1802939	0.0105053
C	-2.4943135	0.4707730	-0.4091876
H	-4.6077807	-1.1293486	-1.3370970
H	-4.7374454	-1.5840928	0.3939262
H	-2.6964766	-2.6369319	-1.2549702
H	-2.4056689	-2.2930512	0.4711521
O	-2.0036667	1.5711421	-0.3569220
O	3.2139208	-0.4427298	-0.7250504
O	-0.5883212	0.4039922	1.8961801
C	4.3140826	-0.0522051	-1.5514789
H	3.9549608	0.4531068	-2.4591799
H	4.8237728	-0.9814661	-1.8204907
H	4.9984349	0.6036530	-0.9960872
C	-1.4313029	1.2922341	2.6444890
H	-2.0087121	1.9333536	1.9681770
H	-2.1063094	0.6442717	3.2119697
H	-0.8226054	1.8952925	3.3329074
C	-0.1107818	1.3574964	-2.4251479
H	0.5246077	1.5611199	-3.2984194
H	-1.1695069	1.4133691	-2.7063495
H	0.0526398	2.1769261	-1.6941375
H	1.2139038	-0.3884455	-2.1077875

TS_I-C_RS

O	-2.2475757	0.4982810	0.5012145
C	-0.0320946	-1.2465390	-0.5780331
C	0.3038999	-2.4051964	0.2378522
C	1.0490918	-0.3485080	-0.9602483
C	1.6269567	-2.6301449	0.6181680
C	2.3594566	-0.6080949	-0.5691877
C	2.6251600	-1.7353592	0.2156970
H	3.1666565	0.0683475	-0.8426635
C	-2.7295158	-2.1477025	-0.9678397
S	-1.4354935	-1.0210586	-1.5320843
C	-0.3269059	-0.2518691	1.5600643
C	-1.1158145	0.7911439	0.9306070
H	-0.9869544	-1.1041705	1.7468608
C	0.8477994	-0.1102879	2.4528691

H	-2.4808170	-3.1917879	-1.1755499
H	-2.9440479	-1.9790843	0.0906546
H	-3.5954554	-1.8400954	-1.5688542
H	1.8925114	-3.4938588	1.2253468
N	-0.6679249	2.1029225	0.7543736
C	-1.5970520	3.0696949	0.1766007
H	-1.8662411	2.7726284	-0.8486311
H	-2.5146282	3.1472889	0.7722182
C	-0.7318300	4.3207694	0.2222167
H	-0.8356857	4.8613867	1.1741584
C	0.6487374	2.5431362	0.5678733
O	0.6080698	3.8119578	0.1286356
H	-0.8984660	5.0099084	-0.6126046
O	1.6671530	1.9153446	0.7195193
H	1.8045100	-0.2076099	1.9060921
H	0.8788542	0.8862832	2.9144199
H	0.8196676	-0.8833830	3.2325617
H	3.6567306	-1.9297995	0.5215629
O	0.6686856	0.6629025	-1.7322977
C	1.6777067	1.4643264	-2.3677118
H	2.3147998	0.8283134	-2.9976889
H	1.1310154	2.1793229	-2.9890654
H	2.2745034	1.9947605	-1.6162536
O	-0.7128291	-3.1999000	0.5516832
C	-0.4712076	-4.3448064	1.3757853
H	-1.4470510	-4.8192582	1.5131187
H	0.2156964	-5.0416746	0.8766202
H	-0.0635847	-4.0402698	2.3498823

TS_I-C_RR

O	2.0838417	-0.6342438	0.5619618
C	0.2066492	0.6842471	-1.4185876
C	-0.9187350	1.1760776	-0.6469084
C	-0.0732769	-0.2047759	-2.5438313
C	-2.2172849	0.8955409	-1.0639140
C	-1.3876505	-0.4703951	-2.9288364
C	-2.4338802	0.0610198	-2.1705014
H	-1.6024212	-1.1136437	-3.7808395
C	1.9866452	2.5216295	-0.1146181
S	1.8203145	1.2199849	-1.3583944
C	0.1299484	-1.5198533	-0.3213854
C	0.8810105	-0.9027537	0.7532514
H	0.8380123	-1.8037328	-1.1054390
C	-1.1555199	-2.2457580	-0.2630457
H	1.3456295	3.3781816	-0.3425061
H	1.8015197	2.1405870	0.8913048
H	3.0415778	2.8097796	-0.2148705
H	-3.0687348	1.2650781	-0.4958471

N	0.3277825	-0.5978790	2.0030943
C	1.2150628	-0.1281893	3.0599190
H	2.0534934	-0.8180862	3.2149017
H	1.6223812	0.8659604	2.8139624
C	0.2330409	-0.0801658	4.2216883
H	0.4238091	0.7273933	4.9366020
C	-0.9979914	-0.2754035	2.3170001
O	-1.0312142	0.1655529	3.5867503
H	0.1782187	-1.0405193	4.7547851
O	-1.9635531	-0.3284404	1.5968535
H	-1.3647077	-2.6264104	0.7456252
H	-2.0133382	-1.5878145	-0.5100722
H	-1.1486368	-3.0723907	-0.9865481
H	-3.4635440	-0.1578718	-2.4658250
O	1.0114461	-0.6241333	-3.1822335
C	0.8637164	-1.4566442	-4.3372854
H	0.3127405	-0.9248704	-5.1248181
H	1.8811325	-1.6730715	-4.6740663
H	0.3490689	-2.3916301	-4.0752834
O	-0.6113725	1.9475054	0.3937337
C	-1.6621757	2.6829298	1.0387506
H	-1.1680591	3.2782645	1.8126161
H	-2.1550905	3.3447220	0.3126504
H	-2.3859490	1.9990692	1.4973074

C

C	-2.8032531	0.0831652	-2.1806946
C	-2.0788062	-1.0924501	-1.9806915
C	-1.2223353	-1.1666908	-0.8863590
O	-0.3928531	-2.1579900	-0.6537148
C	-0.3611970	-3.2857382	-1.5439897
H	0.3964012	-3.9586693	-1.1337388
H	-1.3411393	-3.7804842	-1.5624001
H	-0.0739615	-2.9593081	-2.5520140
C	-1.2077490	-0.1223174	0.1890630
C	-1.8797157	1.1501444	-0.2350082
C	-2.7379383	1.1890480	-1.3280856
H	-3.2920844	2.0916998	-1.5777844
O	-1.6891892	2.1362852	0.5997540
C	-2.3640080	3.3869311	0.3942921
H	-3.4510530	3.2393457	0.4378179
H	-2.0675847	3.8195780	-0.5701674
H	-2.0389774	4.0313989	1.2150991
C	0.2038131	0.0363514	0.7810832
C	0.3568934	0.8897514	2.0470079
H	0.4001366	1.9606375	1.8211924
H	-0.4921783	0.7182706	2.7212694

H	1.2781228	0.5911319	2.5621604
H	0.5634945	-0.9707142	1.0217608
C	1.0537991	0.5681059	-0.3581825
O	0.5428049	1.0577879	-1.3603329
N	2.4253462	0.5421979	-0.2189429
C	3.1762110	-0.3087924	0.6311521
O	2.7522227	-1.0800723	1.4490298
O	4.4720342	-0.1238044	0.3419037
C	4.6372573	0.9876912	-0.5503675
C	3.2861085	1.1138388	-1.2465620
H	3.2280679	0.5237148	-2.1742941
H	3.0042555	2.1496469	-1.4705162
H	4.8783399	1.8811104	0.0436684
H	5.4671175	0.7627194	-1.2286685
S	-2.4534670	-0.7770485	1.4325207
C	-1.5780744	-2.1867022	2.1452782
H	-2.2762061	-2.5697989	2.9037119
H	-0.6501292	-1.8825484	2.6453662
H	-1.3787657	-2.9794279	1.4159162
H	-2.1407526	-1.8974171	-2.7102084
H	-3.4514280	0.1478121	-3.0595343

TS_C-D

C	-2.0802032	-3.0671712	1.1535356
C	-2.1394676	-2.9555003	-0.2415636
C	-1.3397095	-2.0111260	-0.9008607
C	-0.5668711	-1.0749570	-0.1481077
C	-0.4830459	-1.2749797	1.2695824
C	-1.2862927	-2.2174547	1.9256237
H	-2.6829624	-3.8320413	1.6499704
H	-2.7487822	-3.6613228	-0.8041268
H	-1.2377739	-2.3518623	3.0052682
S	-2.0361628	0.8651250	0.2584828
C	-3.6392669	0.1169623	0.6188678
H	-3.9995395	0.3871673	1.6192686
H	-4.3829865	0.3610532	-0.1498544
H	-3.4456164	-0.9657948	0.5978161
O	2.0917039	-1.8966898	-0.3660927
C	1.8506498	-0.7073388	-0.4279541
C	0.4760712	-0.1371244	-0.7162443
H	0.4104345	0.7816066	-0.1230922
C	0.3897487	0.2720442	-2.1906810
H	-0.6278614	0.5948555	-2.4411994
H	1.0763413	1.1142779	-2.3565005
H	0.6611064	-0.5522124	-2.8590112
N	2.8922737	0.2210611	-0.3010478
C	4.2451505	-0.2623750	-0.0553368
C	5.0197797	1.0448276	-0.1660035

O	4.0560961	2.0553520	0.1671184
C	2.8105921	1.5973663	-0.0414072
H	5.8594457	1.1264660	0.5326004
H	5.3751146	1.2272927	-1.1906832
H	4.3155627	-0.7193938	0.9440093
H	4.5399737	-1.0104627	-0.8006627
O	1.8270678	2.2981803	0.0046725
O	0.4017727	-0.4725368	1.8842830
O	-1.2954493	-1.8834621	-2.2351888
C	0.5164849	-0.5451699	3.2990948
H	1.2714020	0.1969079	3.5776977
H	-0.4394644	-0.2972358	3.7840353
H	0.8468063	-1.5443604	3.6177276
C	-2.0354828	-2.7962305	-3.0335427
H	-1.7125496	-3.8319867	-2.8534654
H	-3.1157938	-2.7063619	-2.8439922
H	-1.8246537	-2.5237447	-4.0723704
S	-2.7362779	3.0565072	0.3179970
H	-2.3115334	3.3604314	1.5600854
C	-1.3047402	3.6845994	-0.5996592
H	-1.2702650	4.7740002	-0.4784110
H	-0.3759803	3.2143841	-0.2481536
H	-1.4885092	3.4302846	-1.6499508

D

C	-2.2203832	1.5368740	-1.8396138
C	-2.7730750	0.4180127	-1.2128098
C	-2.1639245	-0.0930915	-0.0548080
C	-0.9635337	0.4604954	0.4249671
C	-0.4484006	1.6092475	-0.2048178
C	-1.0509395	2.1353489	-1.3571578
H	-0.6489617	3.0171610	-1.8551353
O	0.6885686	2.1075389	0.3608202
C	1.3207029	3.1994365	-0.2660275
H	0.6687485	4.0871342	-0.2905287
H	1.6271035	2.9539496	-1.2964510
H	2.2096146	3.4234793	0.3347601
C	-0.2772449	-0.1421771	1.6244220
H	-0.6553406	-1.1734844	1.7014408
C	-0.5849670	0.6113482	2.9159345
H	-0.2219861	1.6450224	2.8458373
H	-1.6711473	0.6194429	3.0793341
H	-0.0959166	0.1317106	3.7746502
C	1.2191174	-0.2403651	1.4061557
O	2.0614551	0.1254694	2.2019269
N	1.6402451	-0.7760294	0.1741065
C	0.9634396	-1.6790833	-0.6502655
O	-0.1287003	-2.1600212	-0.5045456

O 1.7889461 -1.9813947 -1.6884622

C 2.9361202 -1.1301639 -1.6543672

C 3.0402865 -0.7015082 -0.1963050

H 3.4273947 0.3163590 -0.0628081

H 3.6523419 -1.3902348 0.4083943

H 3.8018110 -1.6976524 -2.0158297

H 2.7609347 -0.2668921 -2.3157749

O -2.6572929 -1.1410503 0.6525607

C -3.4410689 -2.0834405 -0.0503672

H -4.4322375 -1.6805436 -0.3137487

H -2.9220691 -2.4193310 -0.9604879

H -3.5782273 -2.9351264 0.6257202

H -3.6919935 -0.0218689 -1.5997489

H -2.7012902 1.9448183 -2.7321399

MeSH

S 0.6583875 -0.0876402 -0.0002239

H 0.8874025 1.2319720 0.0001509

C -1.1512880 0.0234667 -0.0000865

H -1.5288081 -1.0066127 -0.0004016

H -1.5307966 0.5282180 0.8971469

H -1.5308973 0.5288062 -0.8969459

MeSHMe

S -0.8596288 0.6188969 0.3238677

C -2.0484695 -0.7351256 0.1225905

H -2.9692306 -0.3278212 0.5651174

H -2.2307866 -0.9581682 -0.9346015

H -1.7495613 -1.6272143 0.6844251

S 0.8123192 -0.3018516 -0.5253246

H 1.2038343 0.6675708 -1.3808404

C 2.0982713 -0.1116474 0.7336420

H 3.0429926 -0.4385356 0.2790479

H 2.1505648 0.9291971 1.0713366

H 1.8176146 -0.7805009 1.5556092

TS_C-E_S

O -0.6881009 -1.7907496 -1.1484105

C 1.6662143 0.1804064 0.3104450

C 1.2842030 -1.1413640 0.7421898

C 0.7842569 1.2921619 0.6475645

C 0.2569192 -1.2833762 1.6692943

C -0.2567207 1.1082437 1.6017600

C -0.5030808 -0.1629957 2.0804203

H -0.8692491 1.9518825 1.9116386

C 4.1631559 -0.7938457 -0.7239906

S 3.1943288 0.6754697 -0.2957450

C -0.0616824 0.5074423 -1.2537158

C	-0.9957550	-0.6161768	-0.9684438
H	-0.5103239	1.5014743	-1.2101632
C	0.8842095	0.2930257	-2.3759928
H	4.3788253	-1.4034903	0.1575050
H	3.6759708	-1.3901230	-1.5002044
H	5.0941225	-0.3622949	-1.1153694
H	0.0076605	-2.2697712	2.0593867
N	-2.2721241	-0.3086107	-0.5165488
C	-3.2154767	-1.3864132	-0.2449112
H	-3.2111091	-2.1254265	-1.0552435
H	-2.9505203	-1.8963802	0.6941796
C	-4.5199936	-0.5990113	-0.1485495
H	-5.1900276	-0.9455453	0.6453609
C	-2.8399793	0.9446317	-0.2060158
O	-4.1091725	0.7414129	0.1637204
H	-5.0625791	-0.5798404	-1.1042591
O	-2.2998899	2.0221315	-0.2281352
H	0.3361221	0.4907851	-3.3151607
H	1.7109530	1.0210328	-2.3419817
H	1.2712410	-0.7335343	-2.3991577
H	-1.2940840	-0.3075203	2.8212919
O	1.2335252	2.4780284	0.2633806
C	0.5055076	3.6632418	0.6312413
H	1.0182170	4.4833883	0.1213101
H	-0.5364833	3.5931209	0.2960163
H	0.5563444	3.8120073	1.7180534
O	2.0004458	-2.1419709	0.2352022
C	1.6026781	-3.4800203	0.5388435
H	2.2681702	-4.1254211	-0.0419300
H	1.7290798	-3.6907195	1.6106460
H	0.5610590	-3.6387800	0.2299893

TS_C-E_R

O	0.339916	1.260917	2.089196
C	1.008676	0.243154	-0.767888
C	2.020180	-0.608141	-0.142864
C	-0.005827	-0.404406	-1.562216
C	2.191717	-1.943228	-0.622585
C	0.166794	-1.724120	-1.967202
C	1.256950	-2.482751	-1.481979
H	-0.563059	-2.192546	-2.627000
C	2.321673	2.645943	0.110571
S	0.852182	1.931723	-0.667821
C	0.575704	-0.963846	1.425099
C	-0.226677	0.268374	1.624136
H	1.485282	-0.806066	2.013196
C	0.058184	-2.357223	1.408193
H	3.226555	2.388184	-0.446331

H	2.386120	2.345475	1.158673
H	2.130959	3.724593	0.033690
H	3.009922	-2.555698	-0.245269
N	-1.577581	0.384891	1.313080
C	-2.229691	1.661769	1.600888
H	-1.843255	2.442217	0.928972
H	-2.052620	1.971292	2.637602
C	-3.680091	1.298212	1.314871
H	-4.203269	0.940289	2.213355
C	-2.406404	-0.406146	0.494895
O	-3.589377	0.214160	0.379116
H	-4.256876	2.106218	0.852291
O	-2.135822	-1.444993	-0.049291
H	0.836376	-3.052970	1.746468
H	-0.299096	-2.665418	0.414517
H	-0.818901	-2.434639	2.070497
H	1.373775	-3.514999	-1.820842
O	-0.993869	0.405158	-1.943076
C	-1.965064	-0.091736	-2.870813
H	-1.479508	-0.363520	-3.818856
H	-2.663597	0.733879	-3.033636
H	-2.493381	-0.951779	-2.439998
O	3.000220	0.053194	0.484243
C	4.081265	-0.686366	1.054361
H	4.680149	0.045390	1.605035
H	4.694489	-1.151479	0.270522
H	3.711259	-1.454592	1.749544

E_S

C	1.9901359	-2.5171110	0.6377110
C	0.7664545	-2.0139479	0.9542394
C	0.4293387	-0.6072347	0.6087478
O	-0.4618173	0.0027969	1.5077724
C	-0.0529211	-0.0489459	2.8722440
H	0.9381410	0.4104815	3.0150595
H	-0.7971769	0.5232483	3.4367335
H	-0.0276489	-1.0849571	3.2415320
C	1.6131310	0.2904102	0.3156236
C	2.8221545	-0.3149518	-0.1571200
C	3.0058004	-1.6757804	0.0800075
H	3.9666293	-2.1286281	-0.1720136
O	3.7426472	0.4993982	-0.6799507
C	5.0056774	-0.0482397	-1.0598002
H	5.5777127	0.7863201	-1.4753200
H	4.8779589	-0.8264262	-1.8258857
H	5.5315036	-0.4606270	-0.1864419
S	1.2482302	1.9177165	0.5035300
C	2.6375701	2.9281092	-0.0681315

H	2.2892008	3.9526621	0.1149389
H	2.8195658	2.7738255	-1.1352417
H	3.5398676	2.7252542	0.5159769
C	-0.3834723	-0.6381935	-0.7698315
C	0.1782311	-1.5071800	-1.8859669
H	-0.4278622	-1.3456527	-2.7891626
H	0.1278348	-2.5716187	-1.6279823
H	1.2134526	-1.2370559	-2.1351680
H	-0.4401688	0.4097690	-1.0832166
C	-1.7740888	-1.1339239	-0.3826650
O	-1.9763633	-2.3007563	-0.0956417
N	-2.8206473	-0.2298798	-0.4251661
C	-2.7670951	1.1847068	-0.4522358
O	-1.8034486	1.8906547	-0.5973160
O	-4.0167434	1.6398330	-0.2791497
C	-4.9637101	0.5661759	-0.3653460
C	-4.1512880	-0.6780974	-0.0303395
H	-4.1667837	-0.9233924	1.0429373
H	-4.4554830	-1.5661454	-0.5968401
H	-5.3631020	0.5340821	-1.3894153
H	-5.7777219	0.7677871	0.3390903
H	-0.0467800	-2.6410566	1.3239930
H	2.2224345	-3.5691388	0.8151514

E_R

O	0.339916	1.260917	2.089196
C	1.008676	0.243154	-0.767888
C	2.020180	-0.608141	-0.142864
C	-0.005827	-0.404406	-1.562216
C	2.191717	-1.943228	-0.622585
C	0.166794	-1.724120	-1.967202
C	1.256950	-2.482751	-1.481979
H	-0.563059	-2.192546	-2.627000
C	2.321673	2.645943	0.110571
S	0.852182	1.931723	-0.667821
C	0.575704	-0.963846	1.425099
C	-0.226677	0.268374	1.624136
H	1.485282	-0.806066	2.013196
C	0.058184	-2.357223	1.408193
H	3.226555	2.388184	-0.446331
H	2.386120	2.345475	1.158673
H	2.130959	3.724593	0.033690
H	3.009922	-2.555698	-0.245269
N	-1.577581	0.384891	1.313080
C	-2.229691	1.661769	1.600888
H	-1.843255	2.442217	0.928972
H	-2.052620	1.971292	2.637602
C	-3.680091	1.298212	1.314871

H	-4.203269	0.940289	2.213355
C	-2.406404	-0.406146	0.494895
O	-3.589377	0.214160	0.379116
H	-4.256876	2.106218	0.852291
O	-2.135822	-1.444993	-0.049291
H	0.836376	-3.052970	1.746468
H	-0.299096	-2.665418	0.414517
H	-0.818901	-2.434639	2.070497
H	1.373775	-3.514999	-1.820842
O	-0.993869	0.405158	-1.943076
C	-1.965064	-0.091736	-2.870813
H	-1.479508	-0.363520	-3.818856
H	-2.663597	0.733879	-3.033636
H	-2.493381	-0.951779	-2.439998
O	3.000220	0.053194	0.484243
C	4.081265	-0.686366	1.054361
H	4.680149	0.045390	1.605035
H	4.694489	-1.151479	0.270522
H	3.711259	-1.454592	1.749544

9. NMR Spectra

pre-1c – (2-Chloro-6-methoxyphenyl)(methyl)sulfane

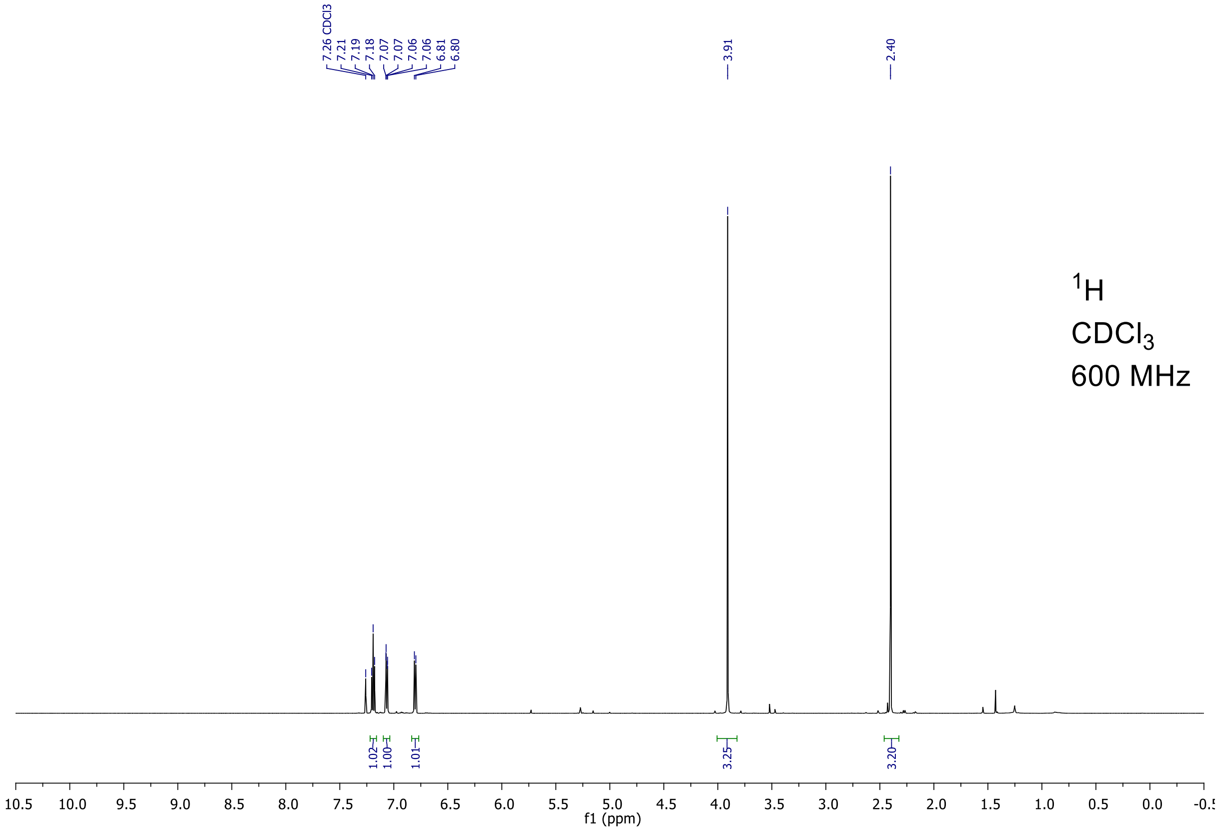

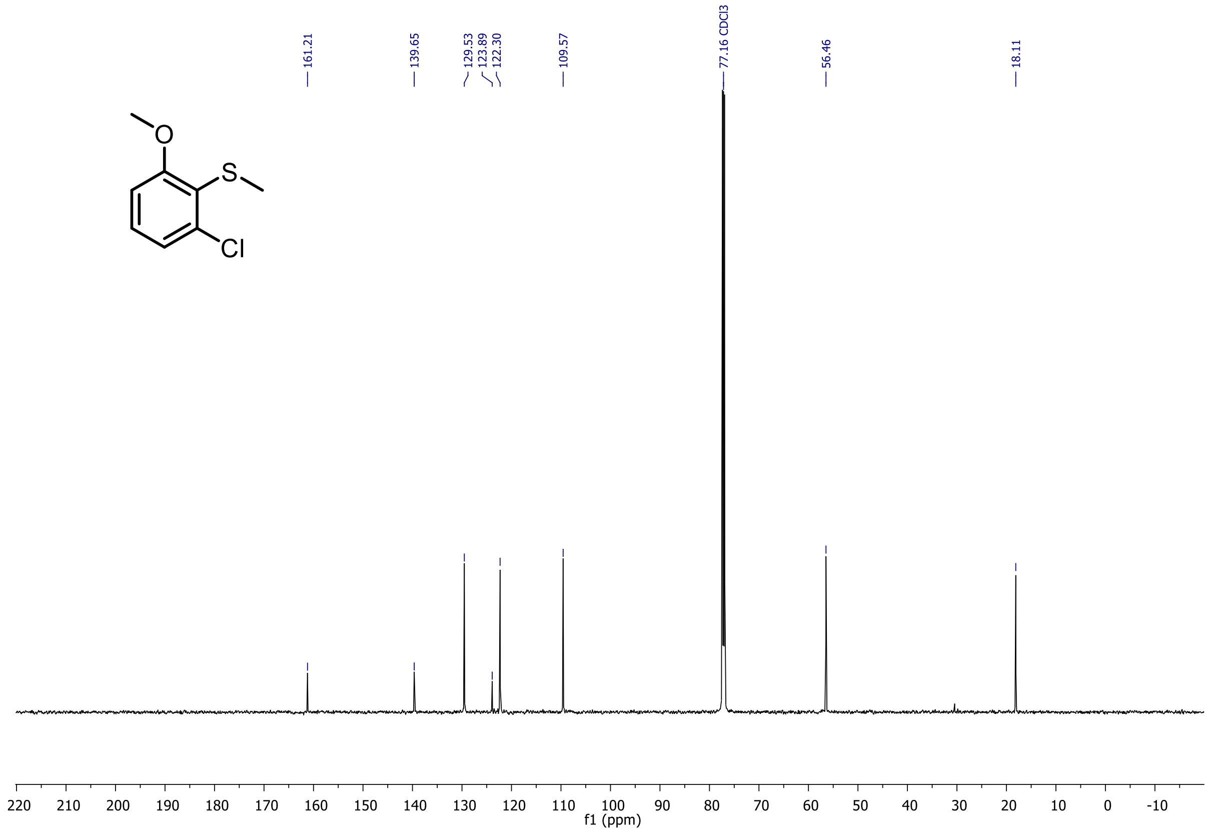

pre3-1d – (1,5-Dichloro-3-methoxyphenyl)(methyl)sulfane

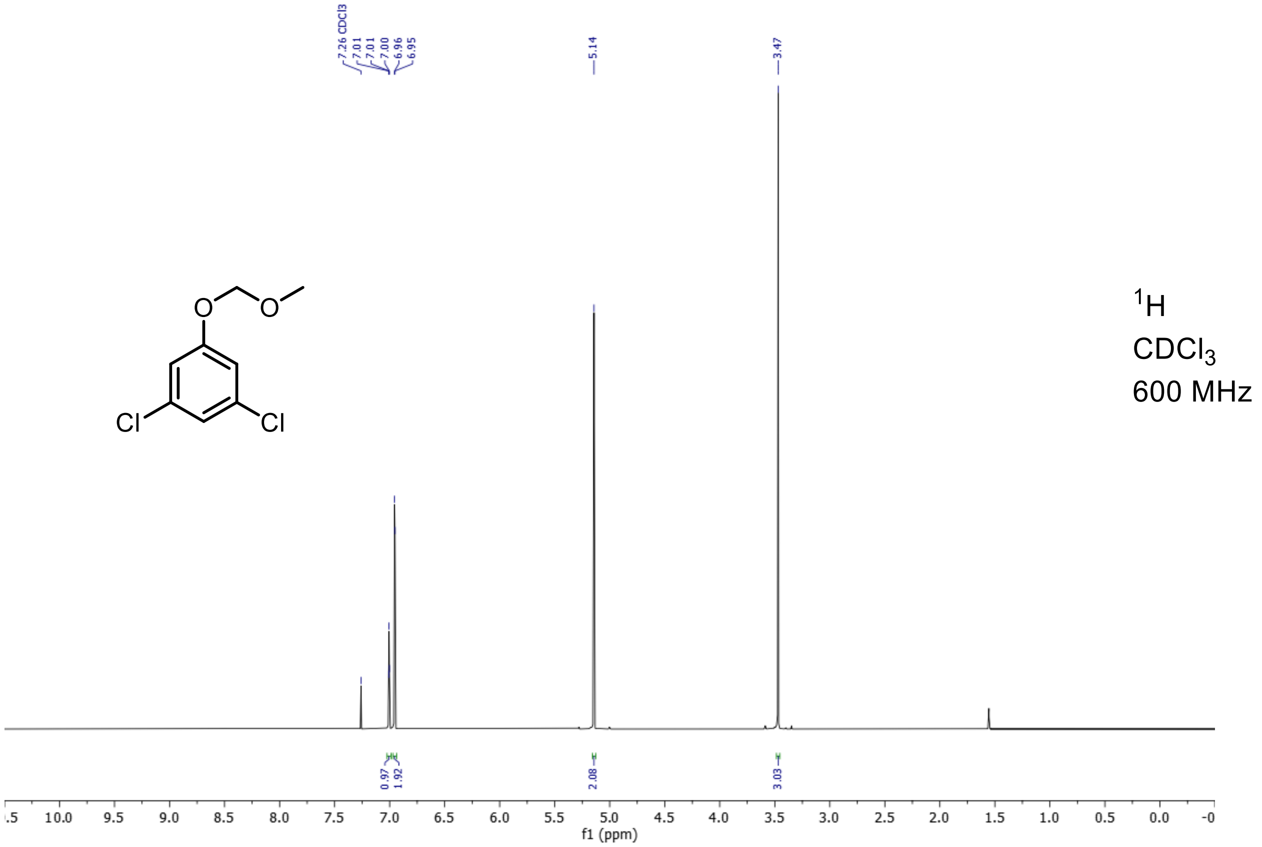

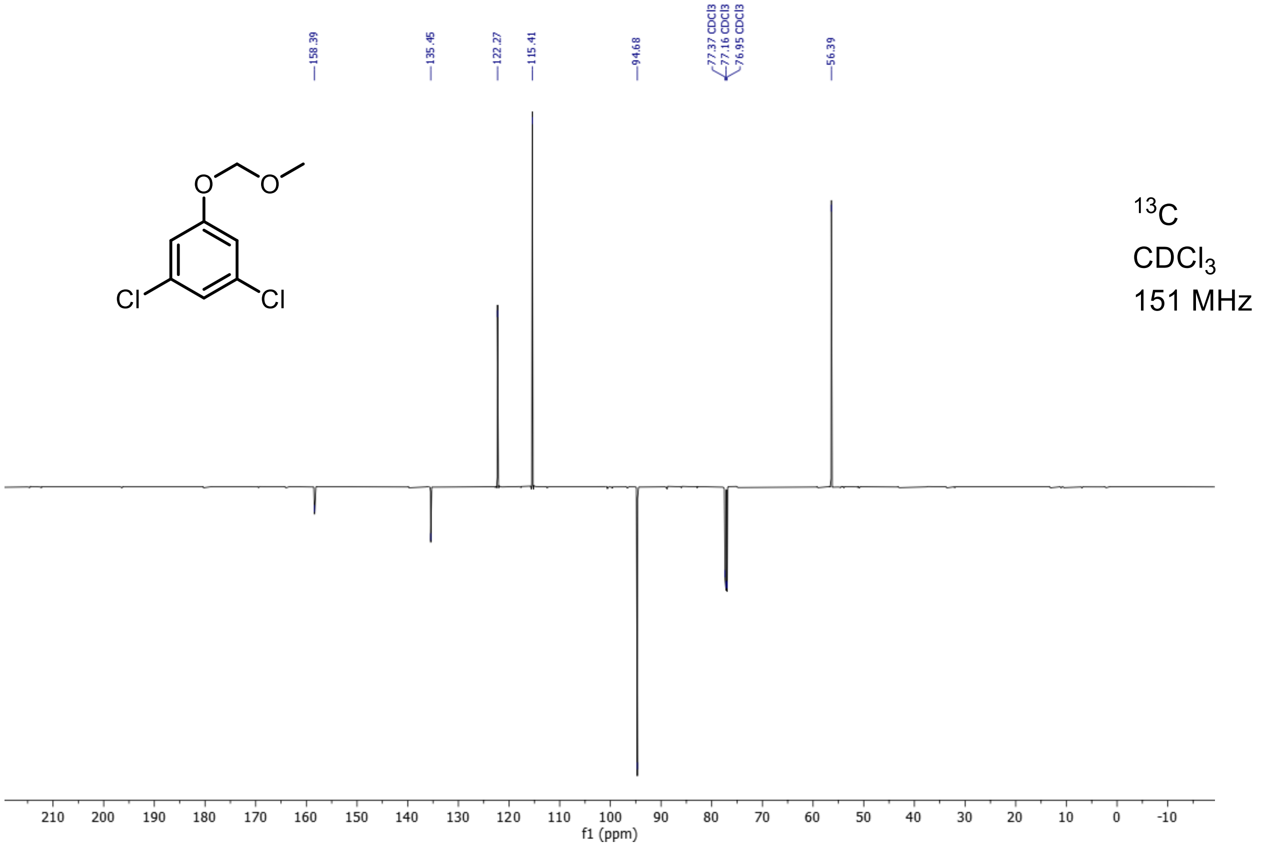

pre2-1d – (1,5-Dichloro-3-methoxyphenyl)(methyl)sulfane

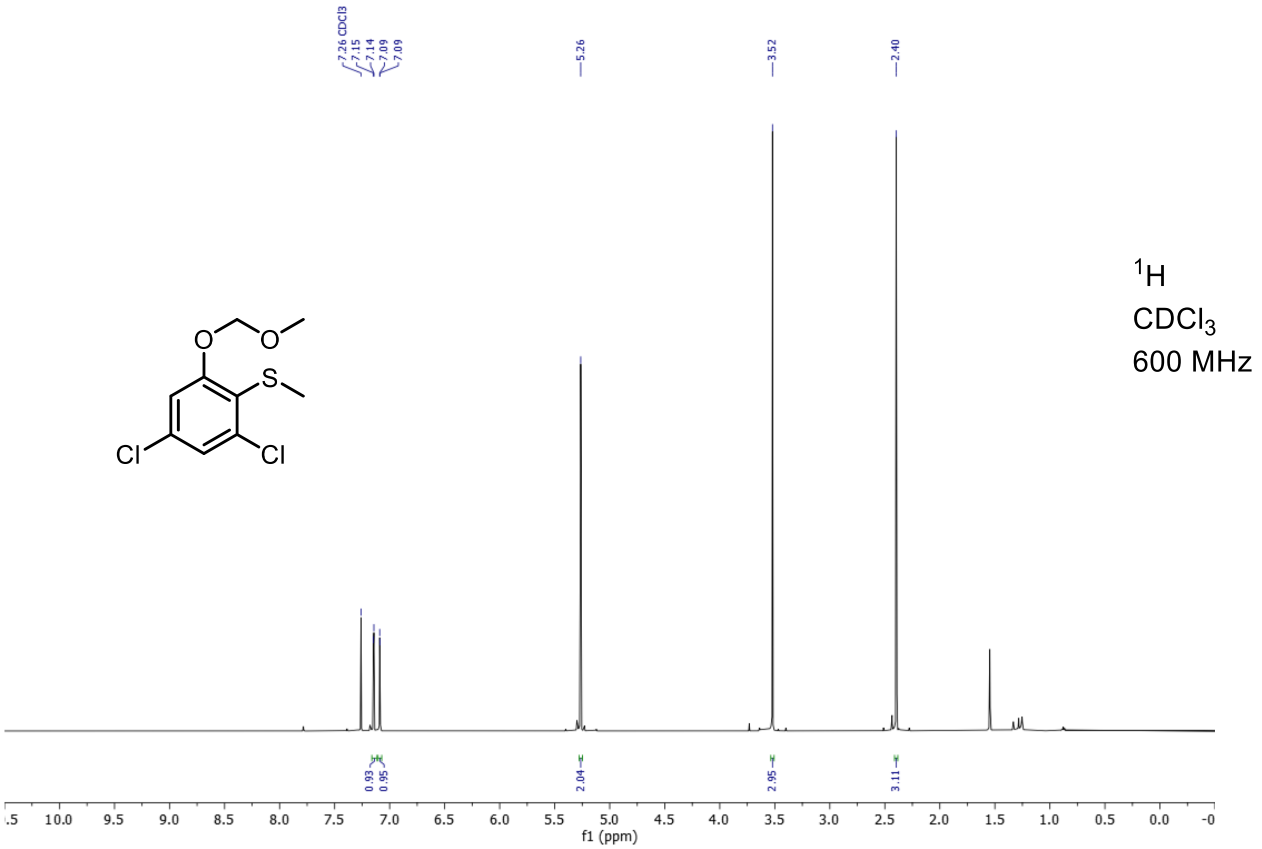

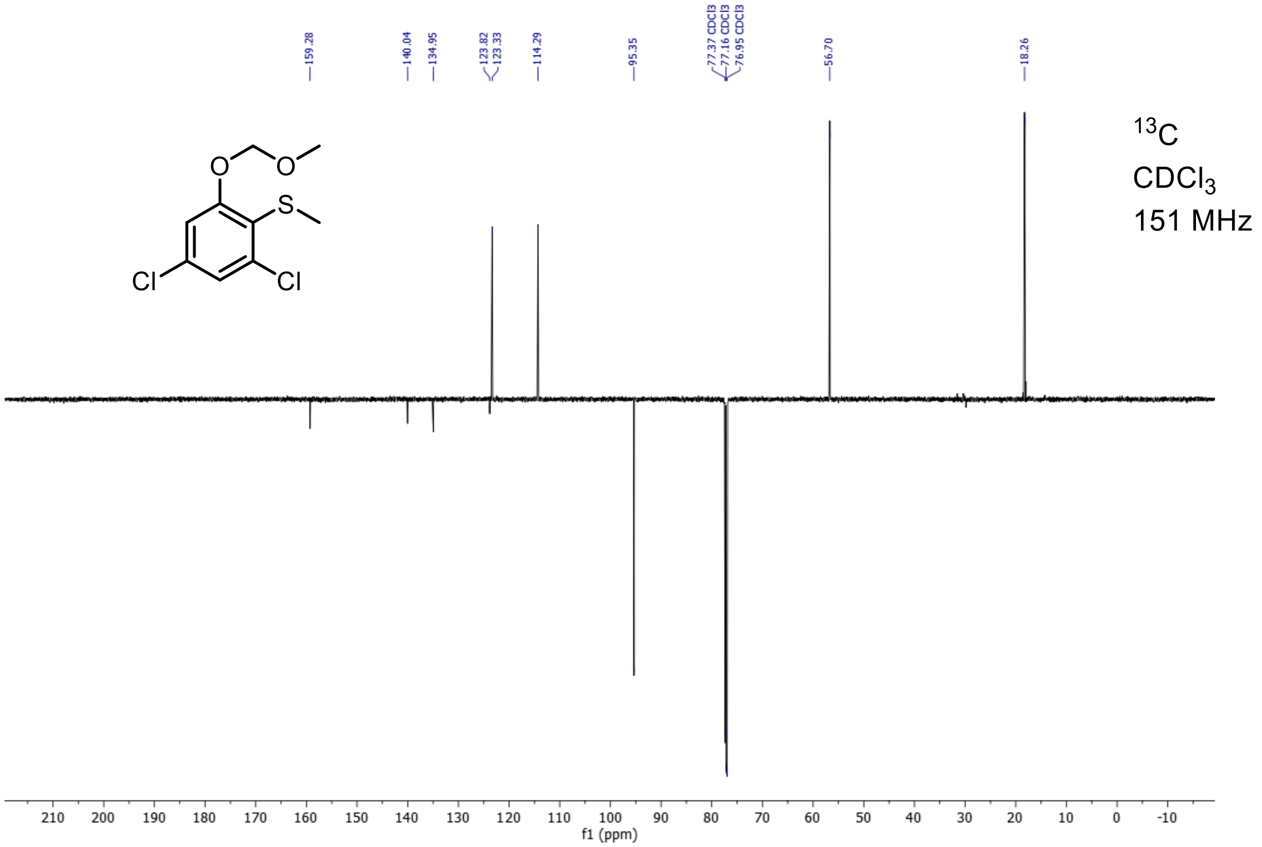

pre1-1d – (1,5-Dichloro-3-methoxyphenyl)(methyl)sulfane

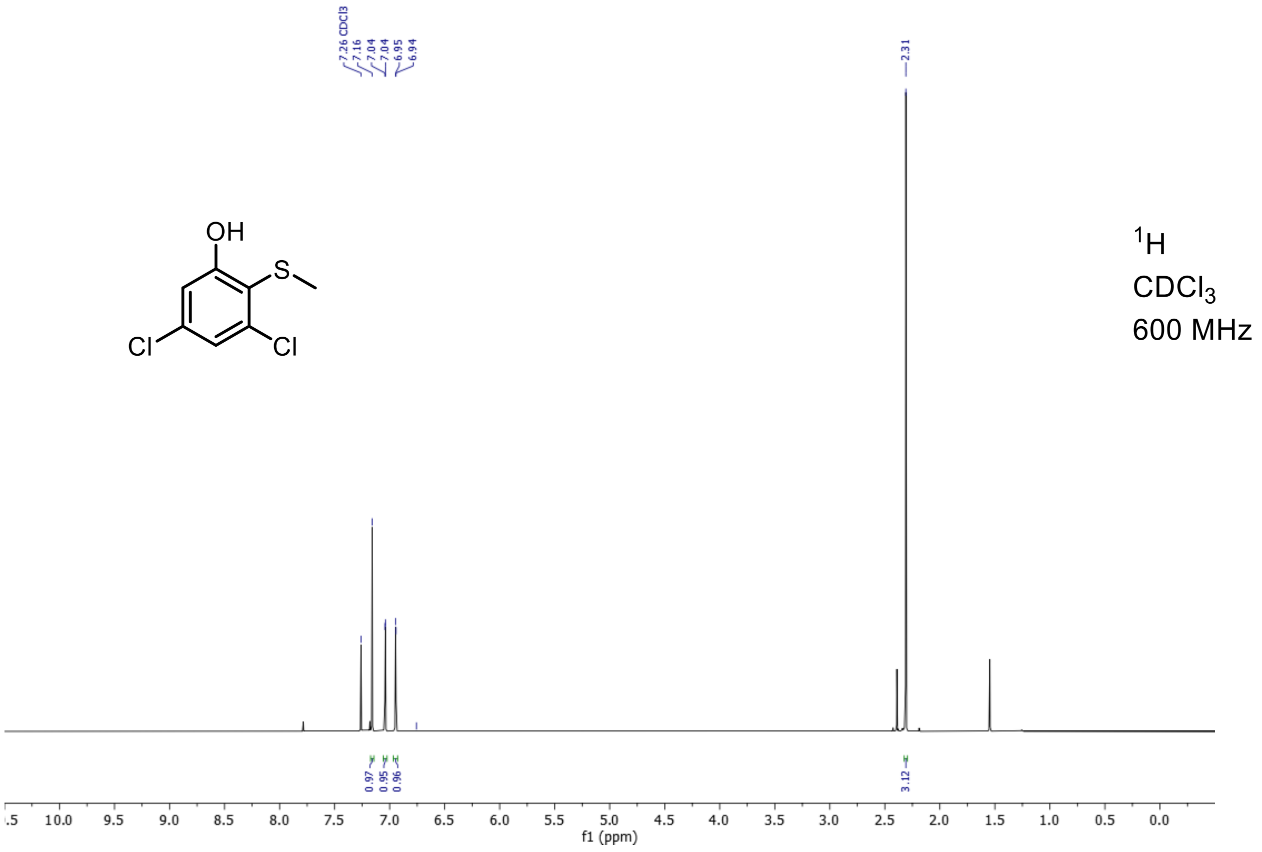

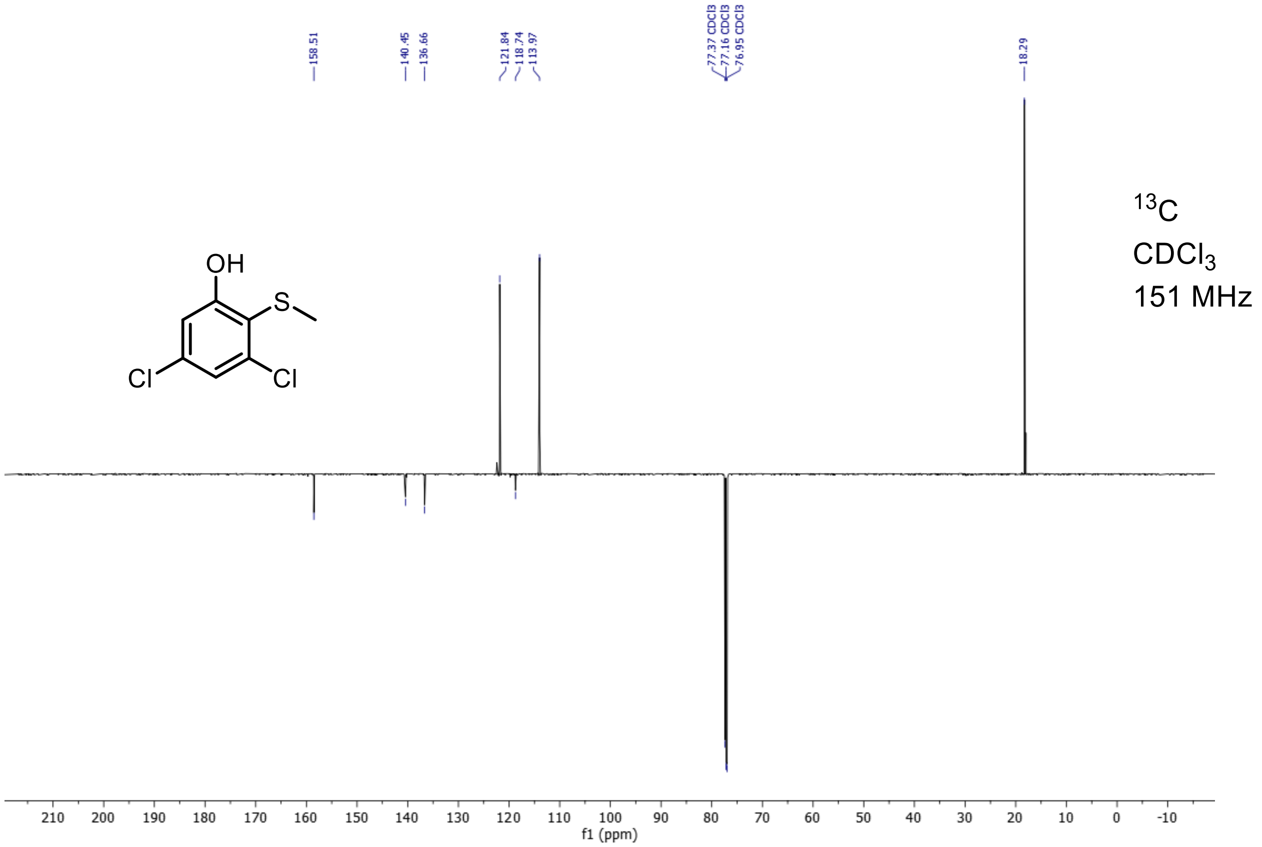

pre-1d – (1,5-Dichloro-3-methoxyphenyl)(methyl)sulfane

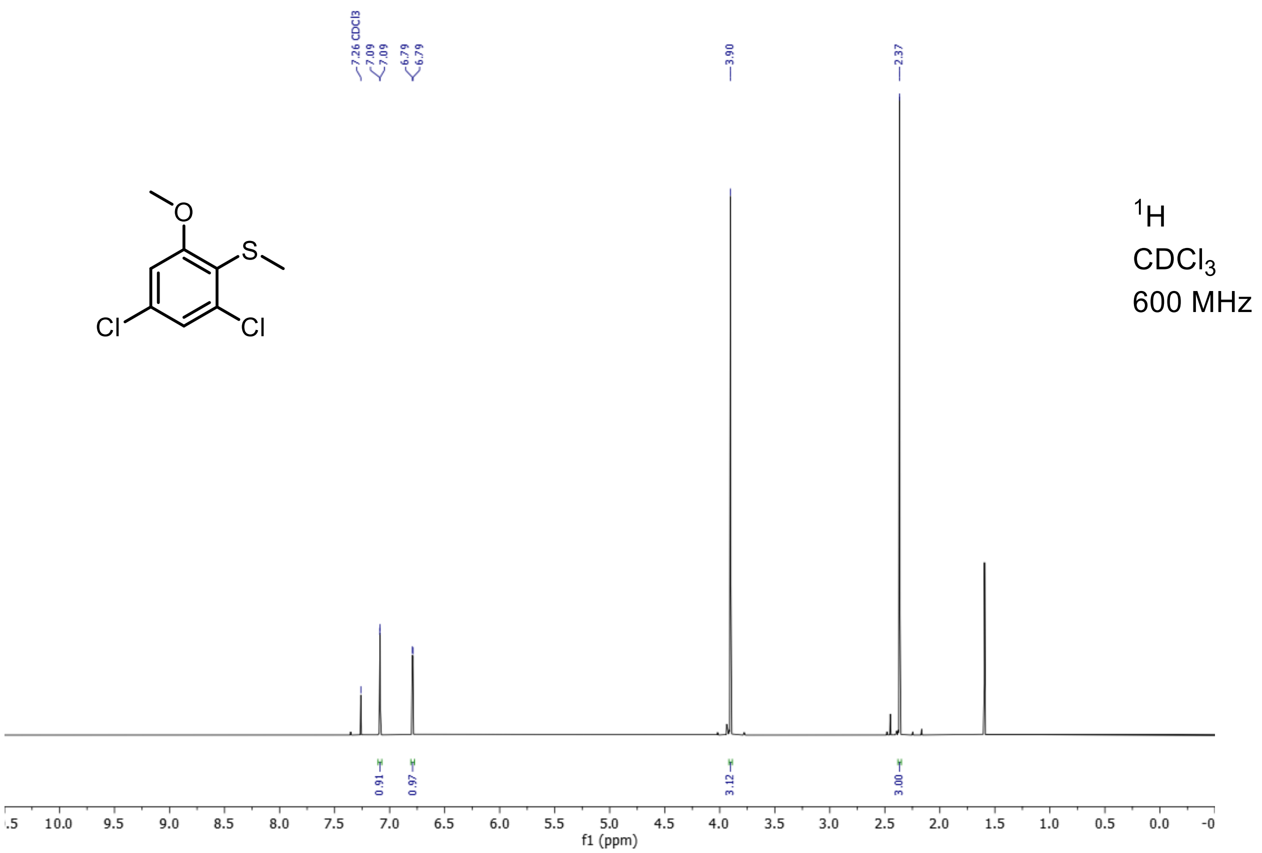

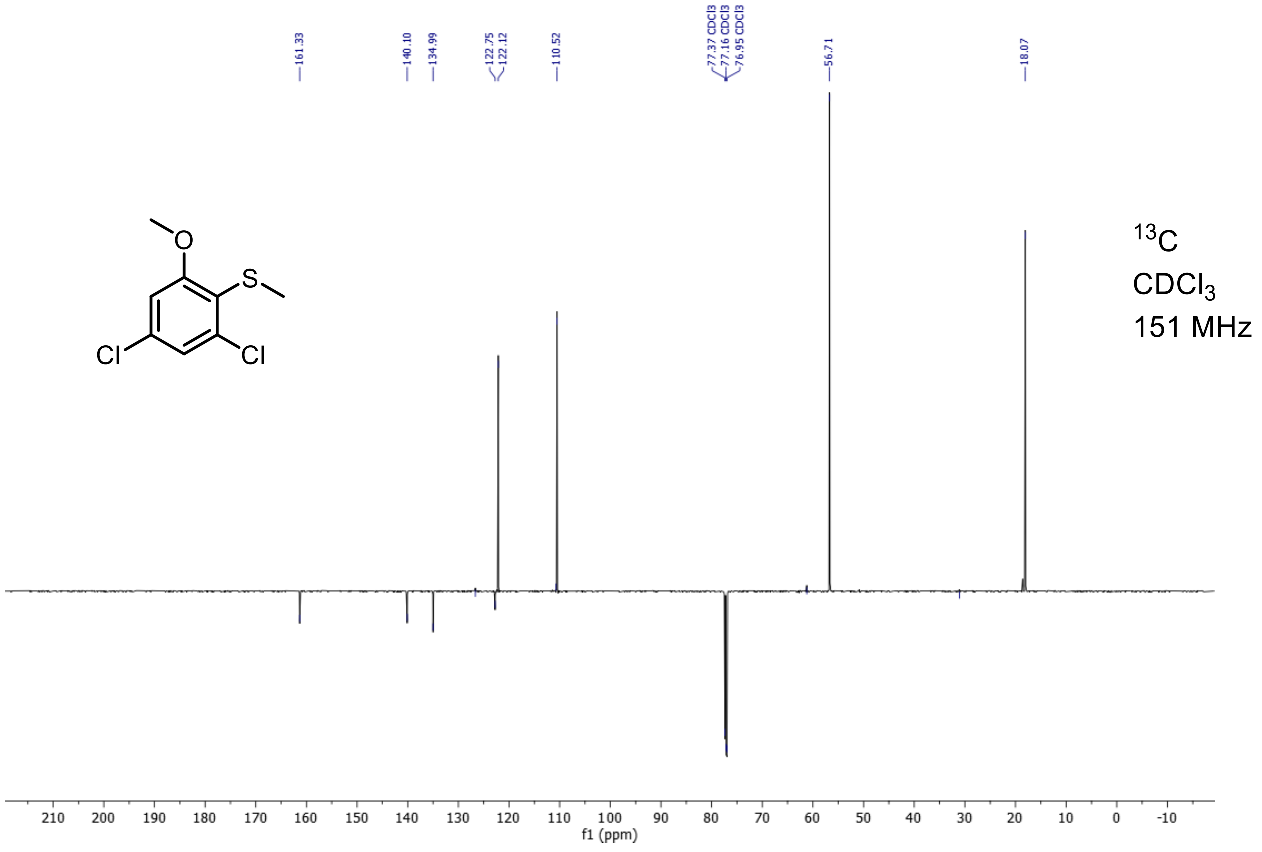

pre-1e – Ethyl 2-bromo-4,6-dimethoxybenzenesulfinate

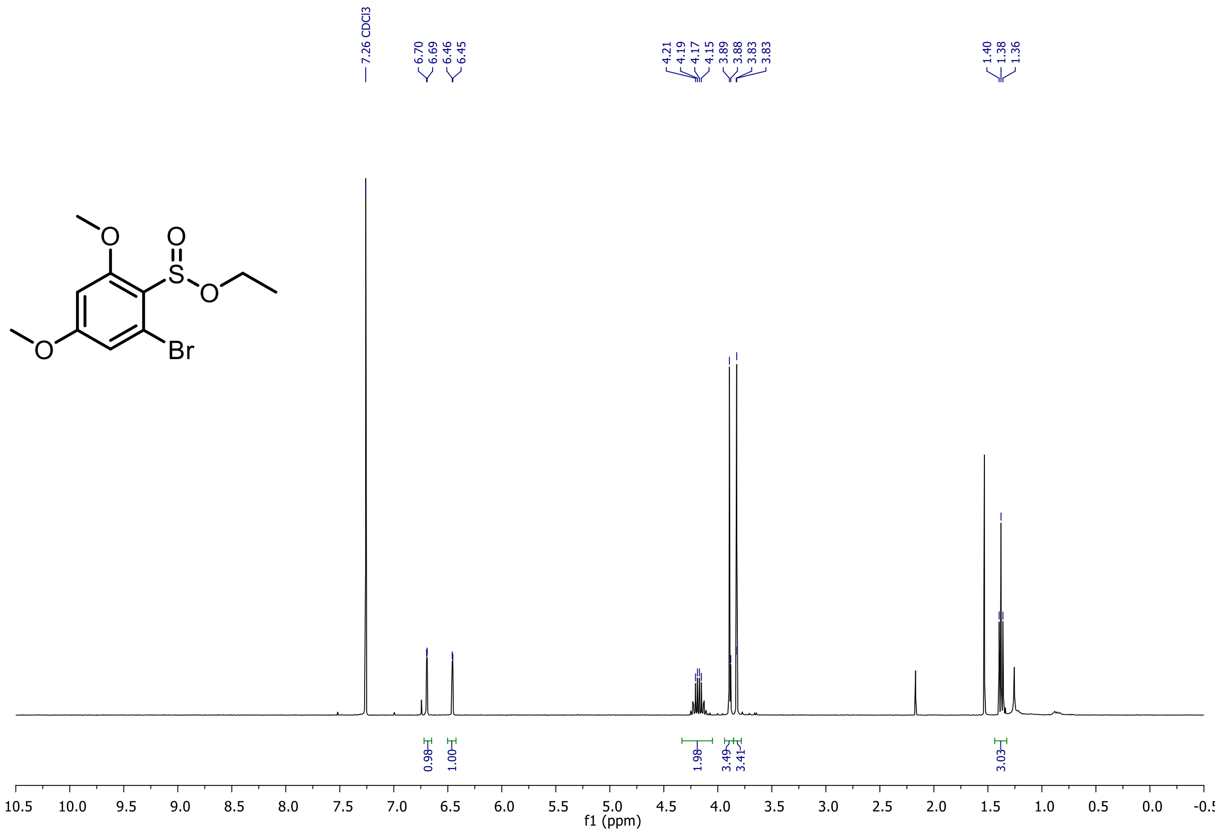

pre-1h – 3,5-Diisopropyl-4-(methylsulfinyl)phenol

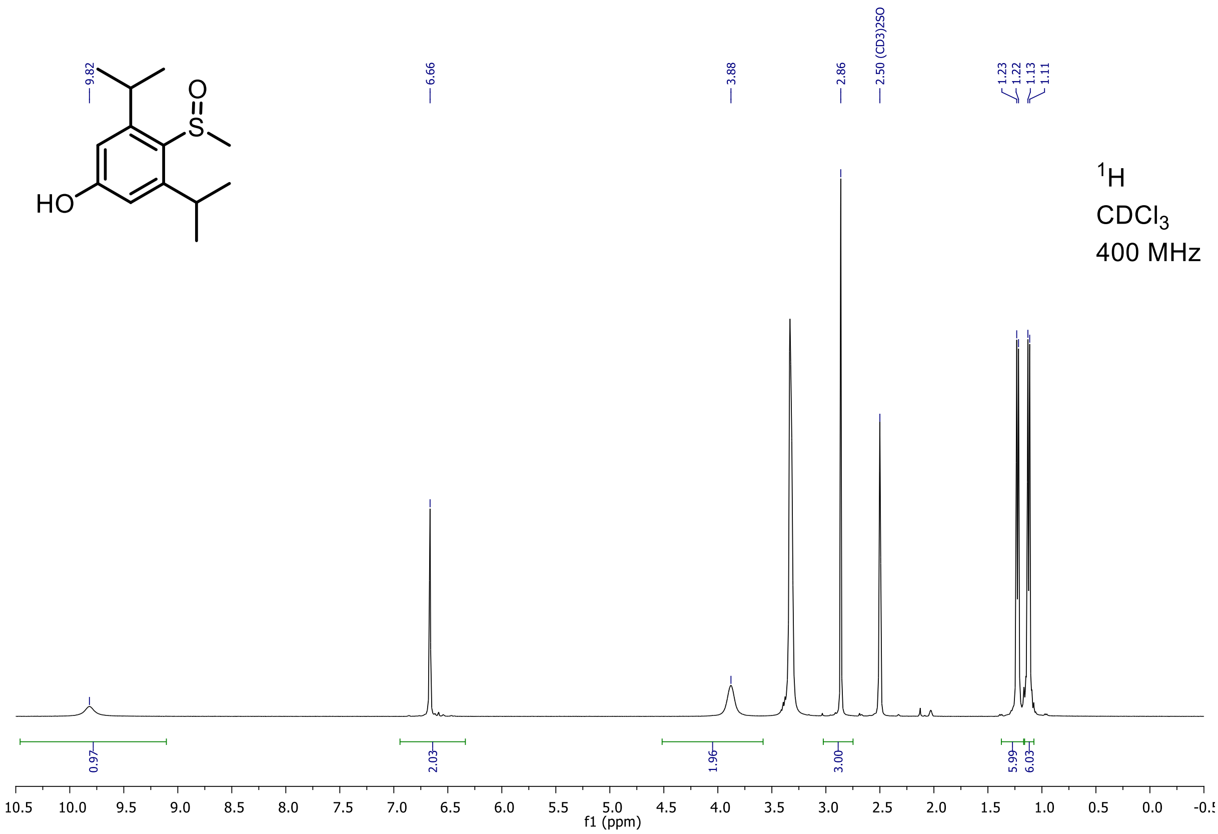

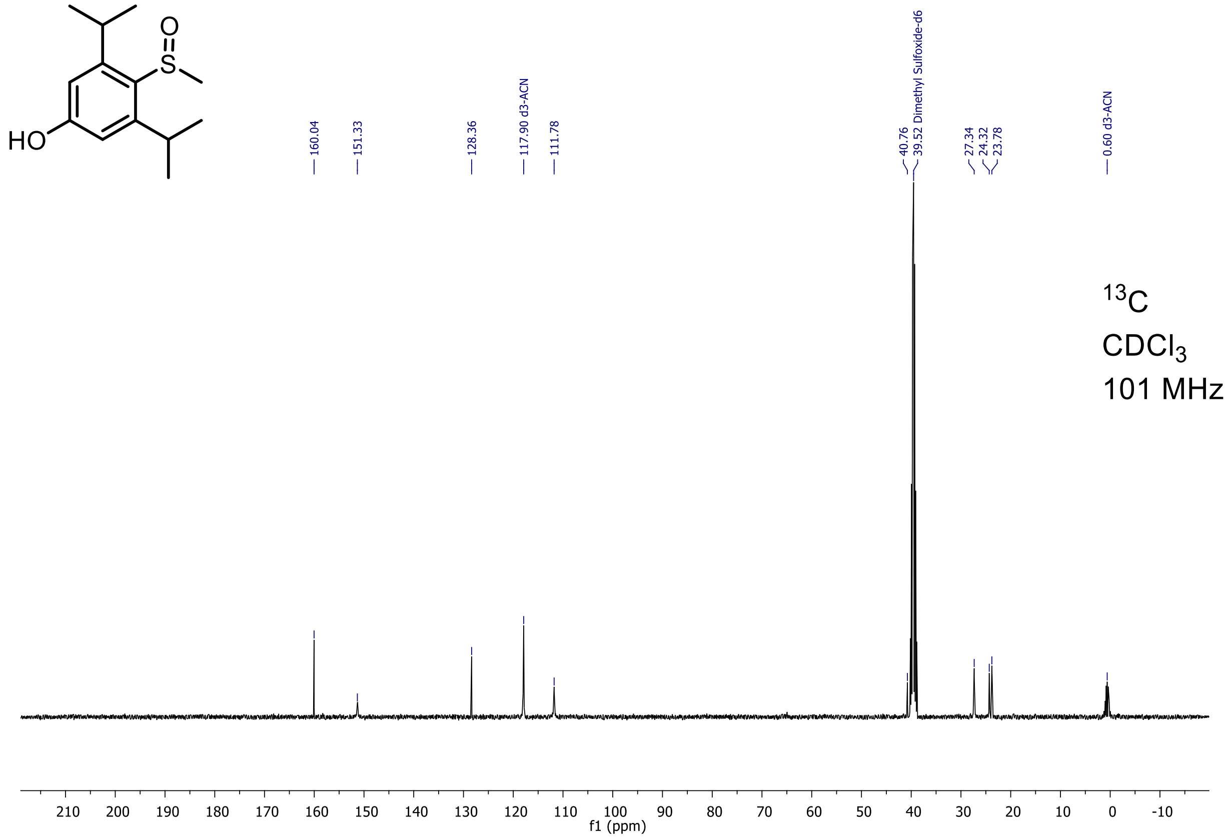

pre-1i – Ethyl 2-(chloromethyl)-4,6-dimethoxybenzenesulfinate

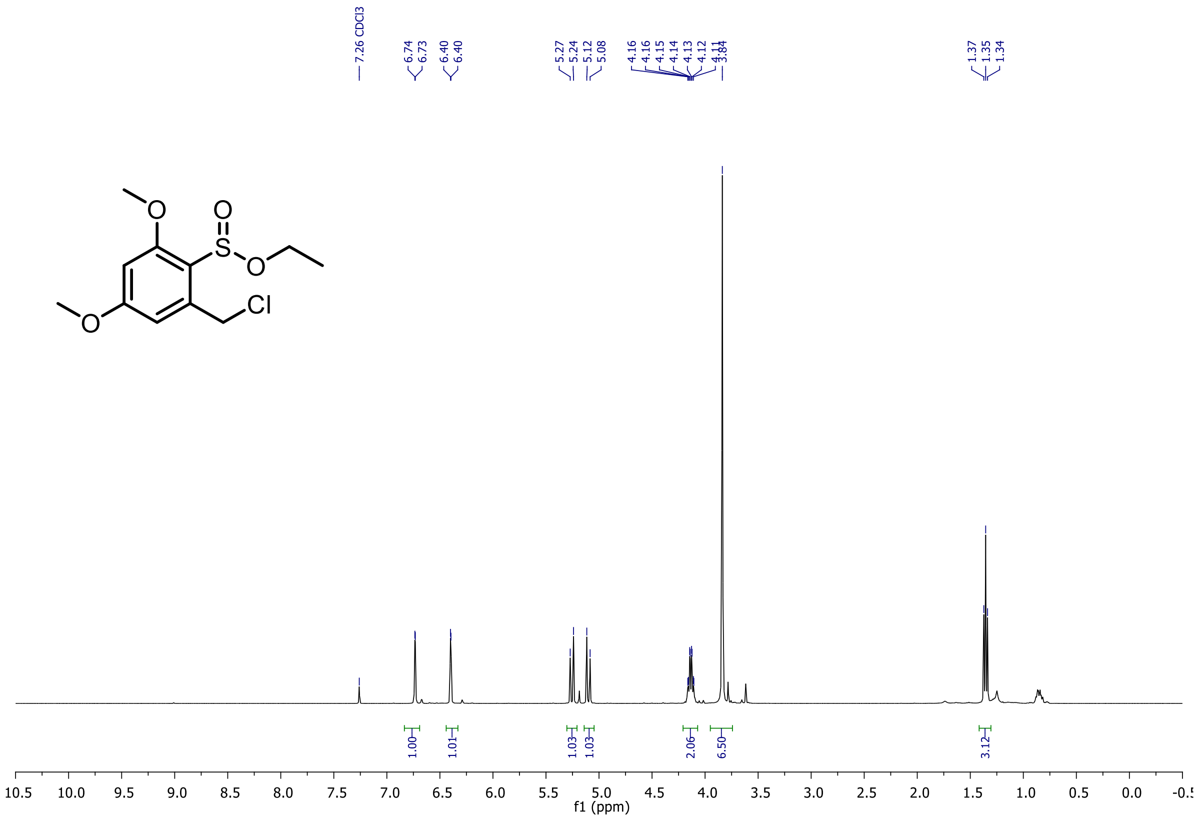

pre-1m – Ethyl 2,4-bis(allyloxy)benzenesulfinate

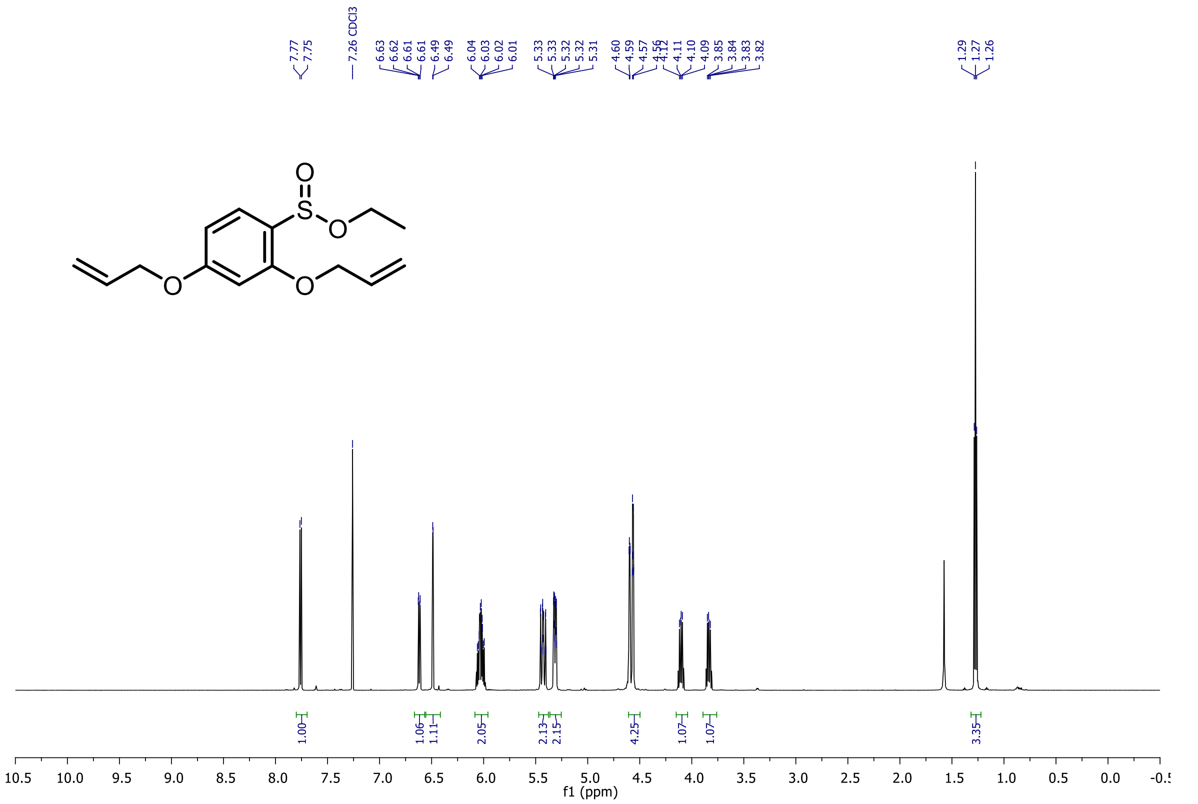

1a – 1,3-Dimethoxy-2-(methylsulfinyl)benzene

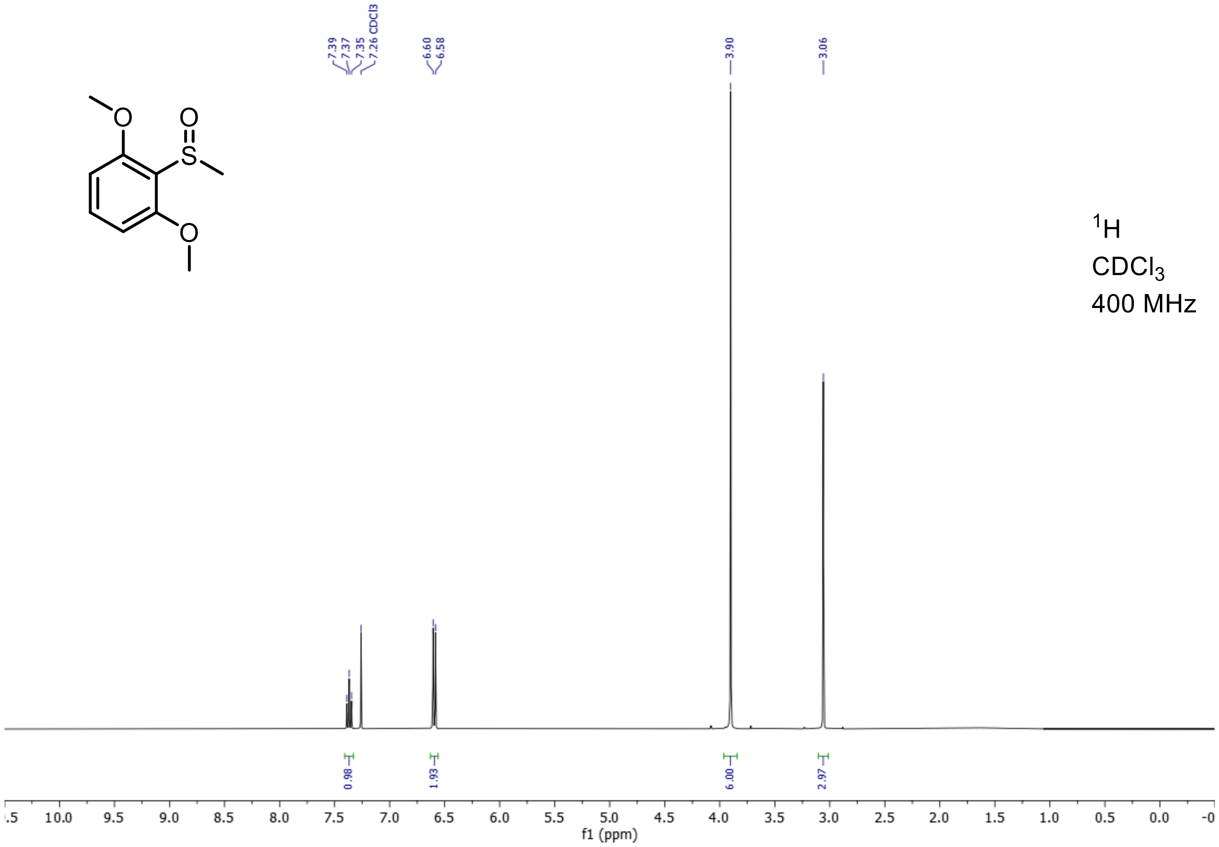

1c – 1-Chloro-3-methoxy-2-(methylsulfinyl)benzene

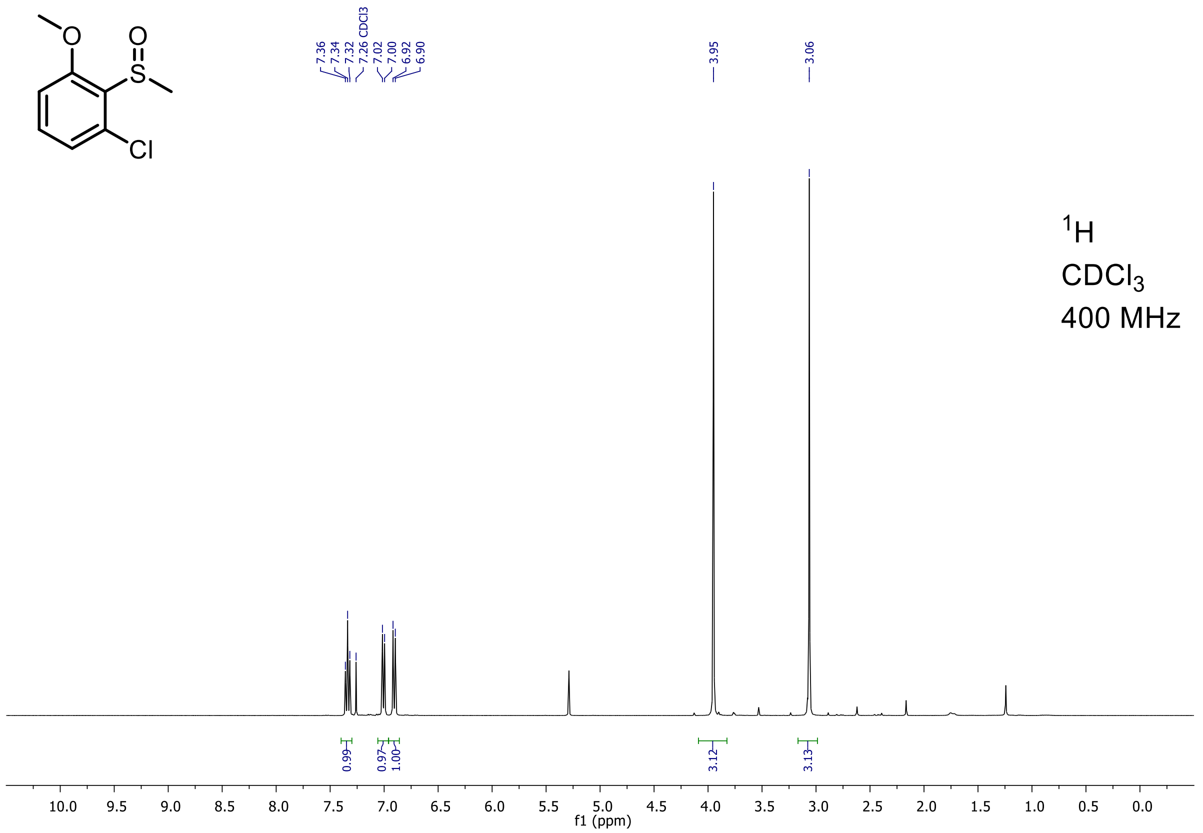

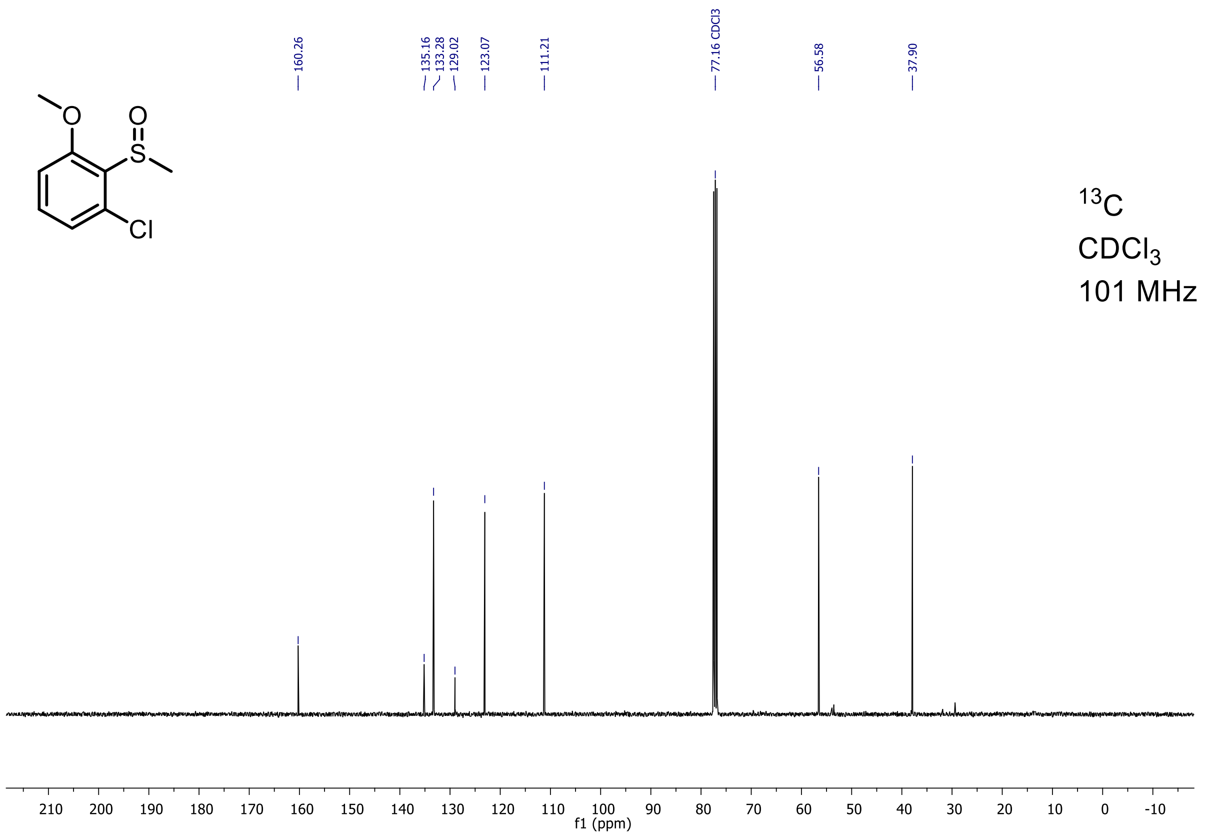

1d – 1,3-Dichloro-5-methoxy-2-(methylsulfinyl)benzene

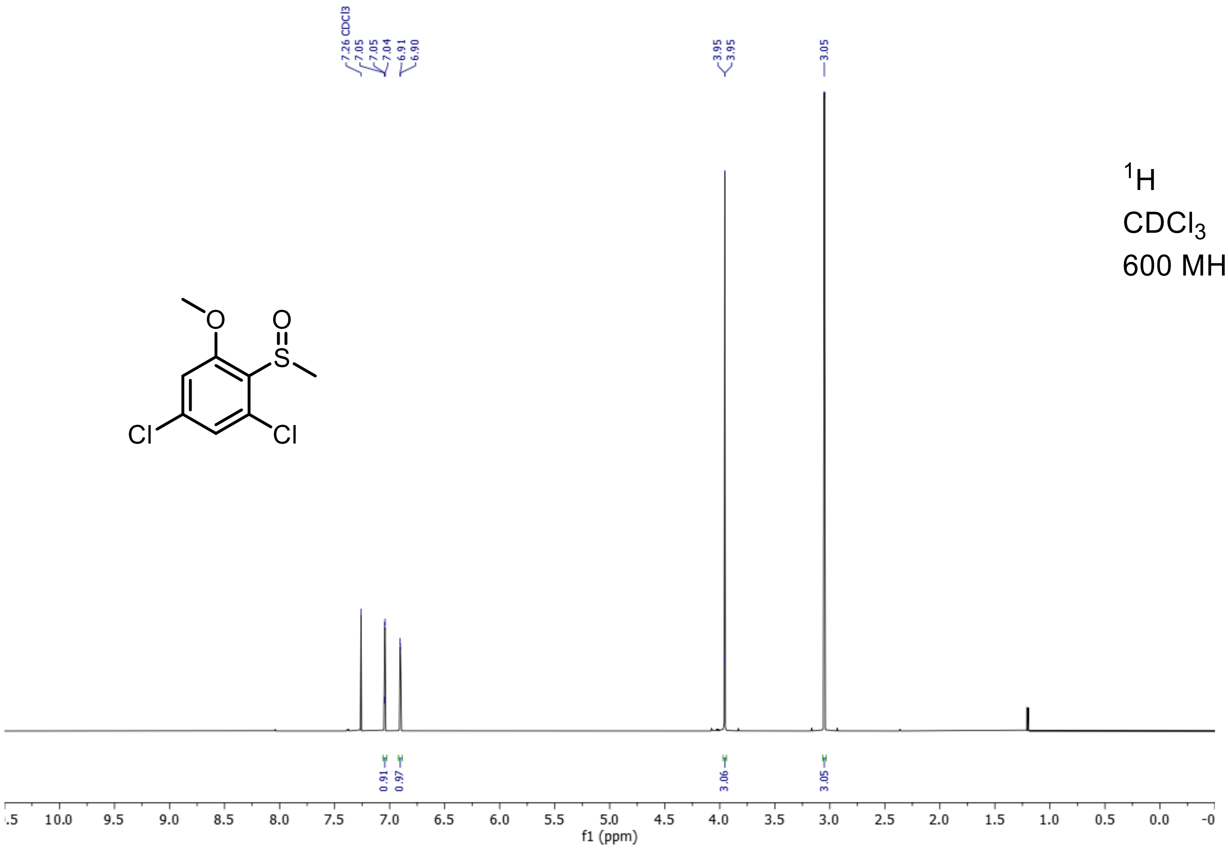

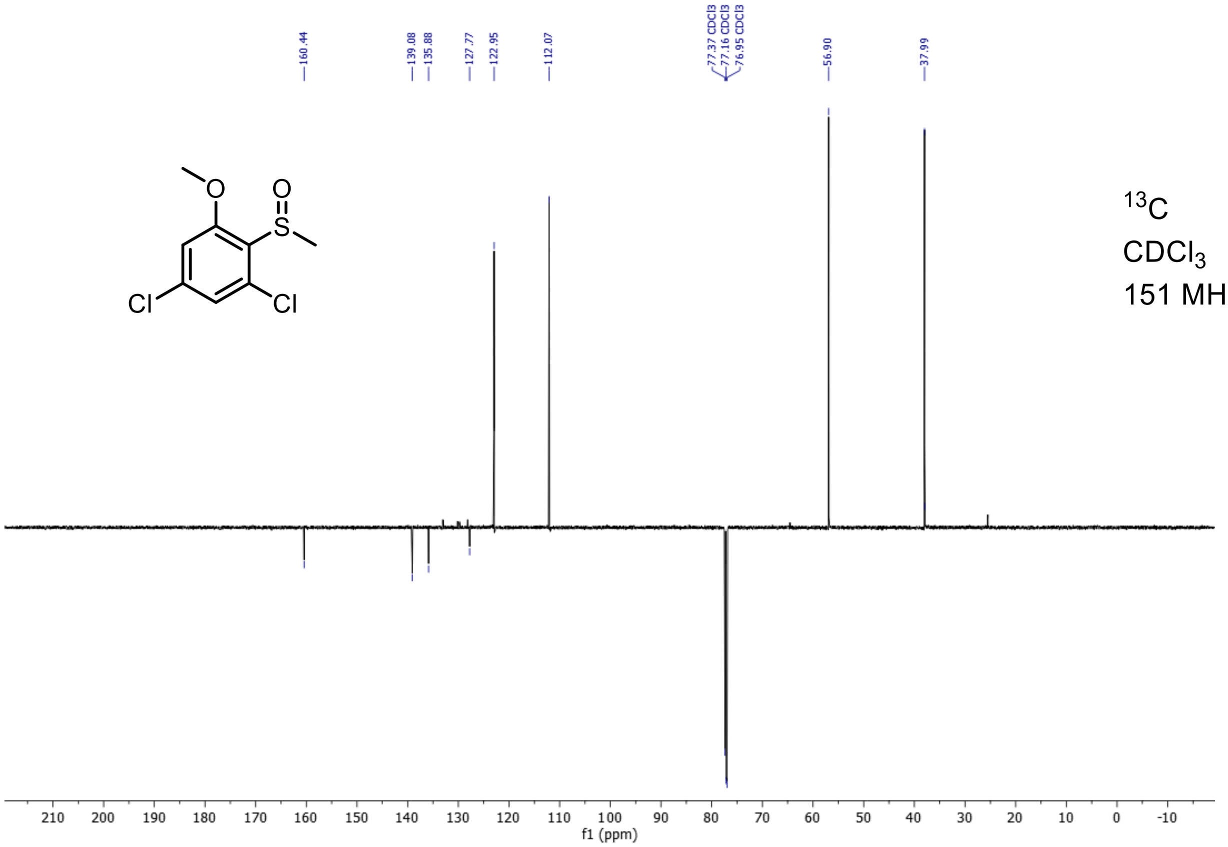

1e – 1-Bromo-3,5-dimethoxy-2-(methylsulfinyl)benzene

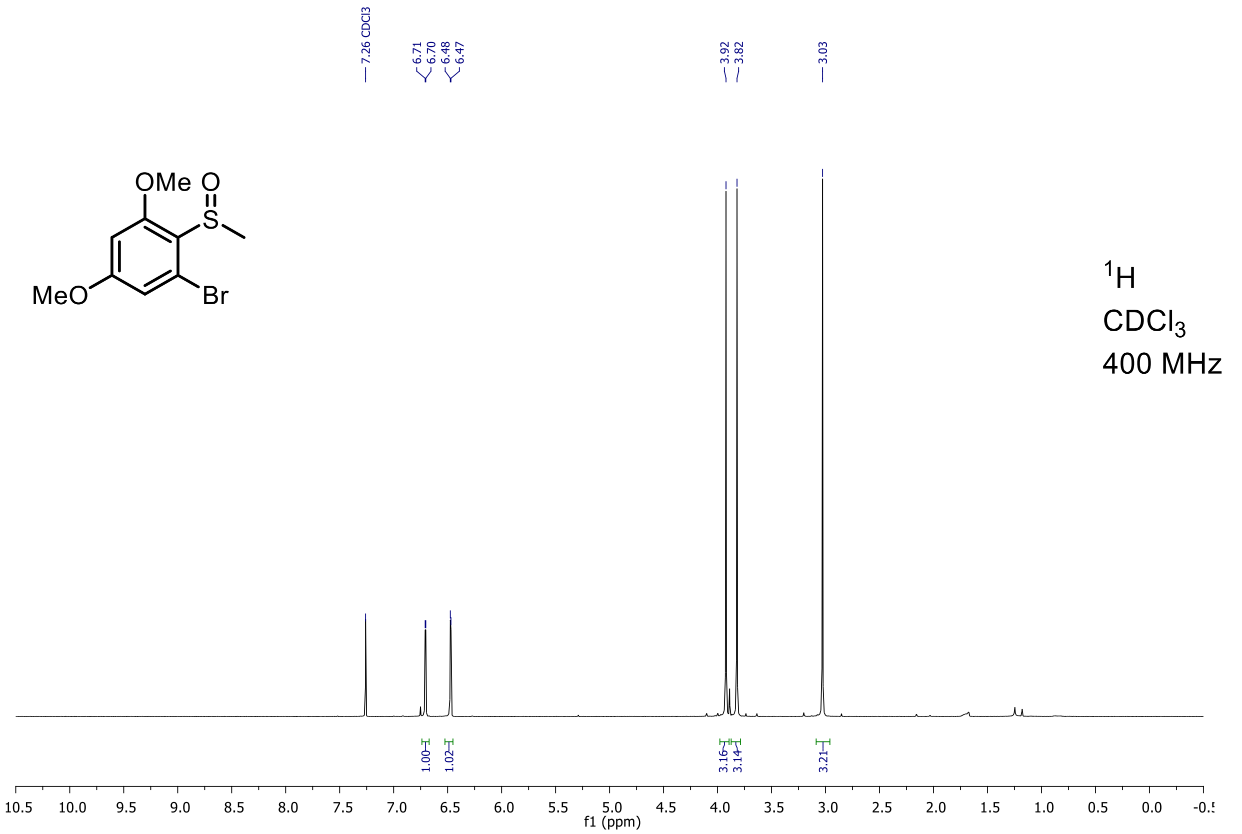

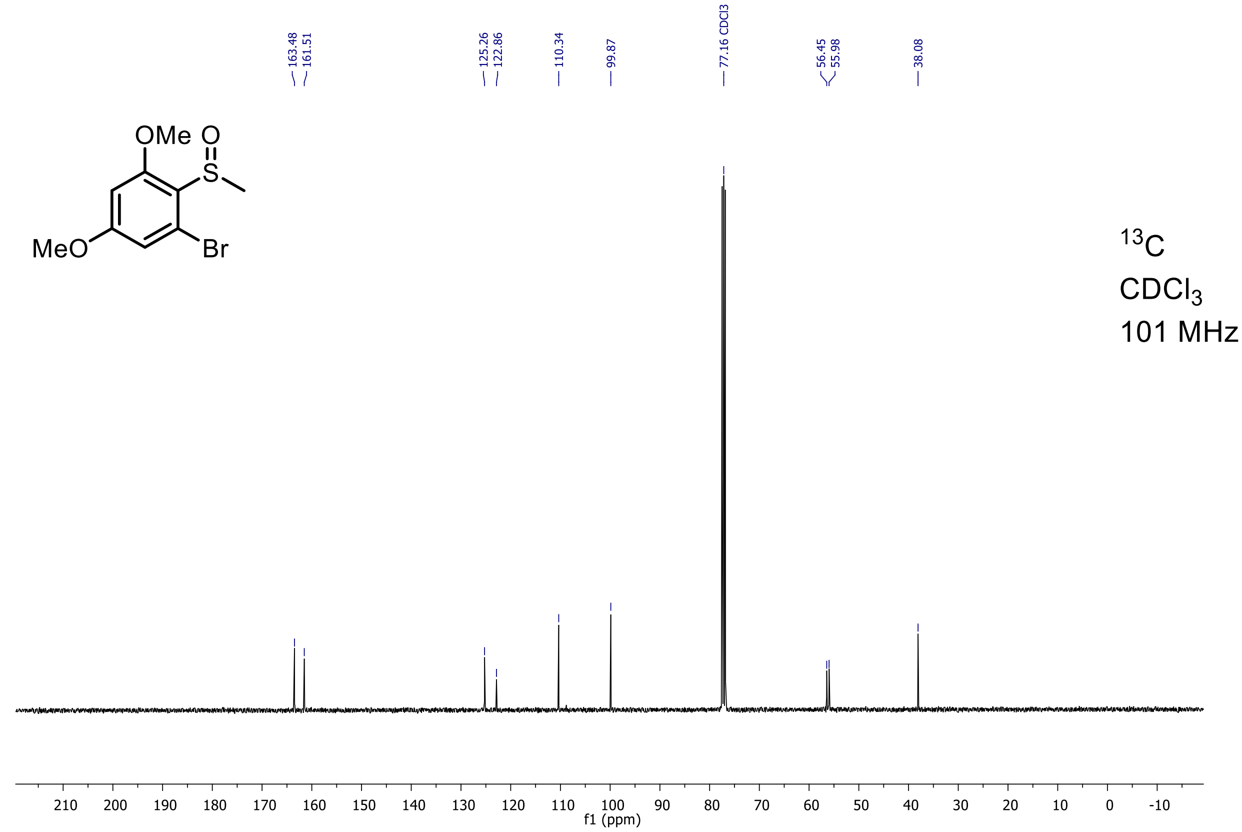

1g – 1,5-Di-*tert*-butyl-3-methoxy-2-(methylsulfinyl)benzene

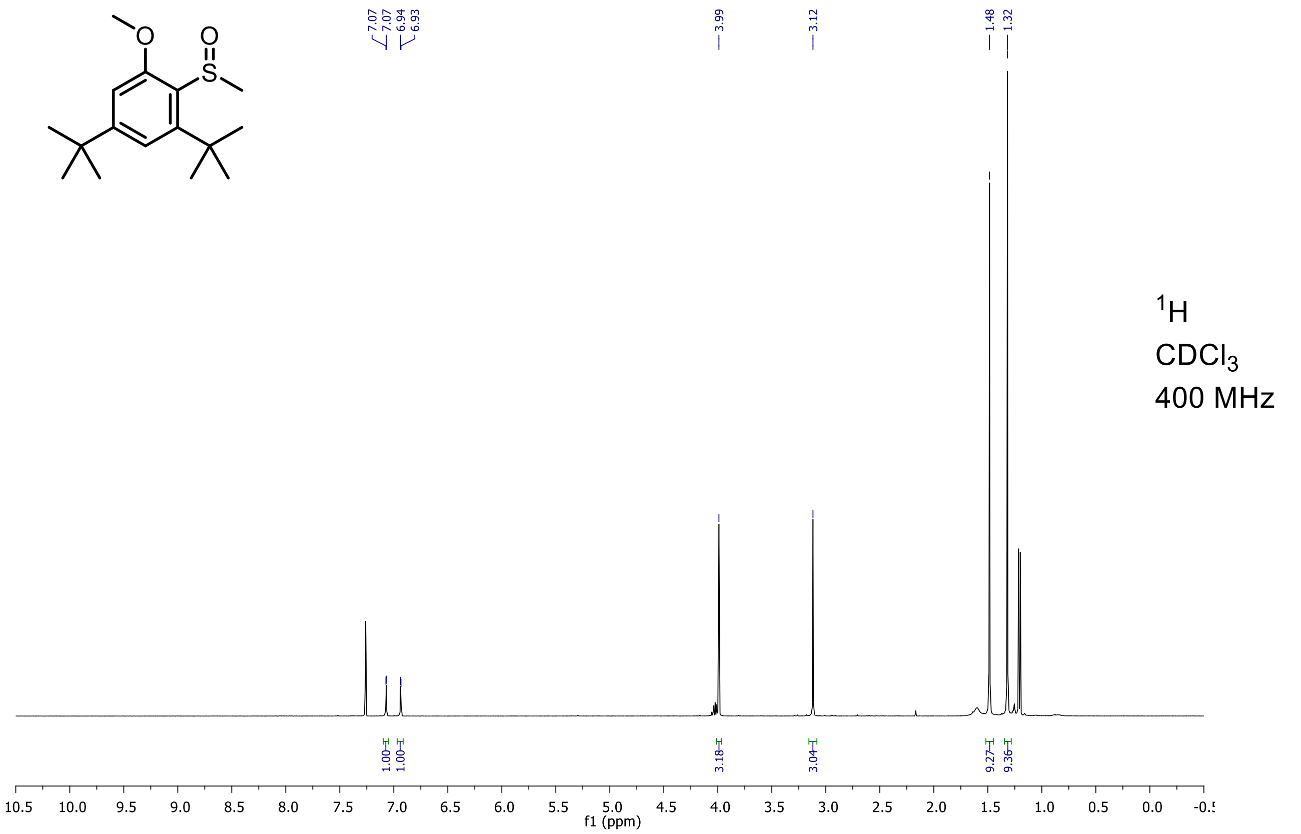

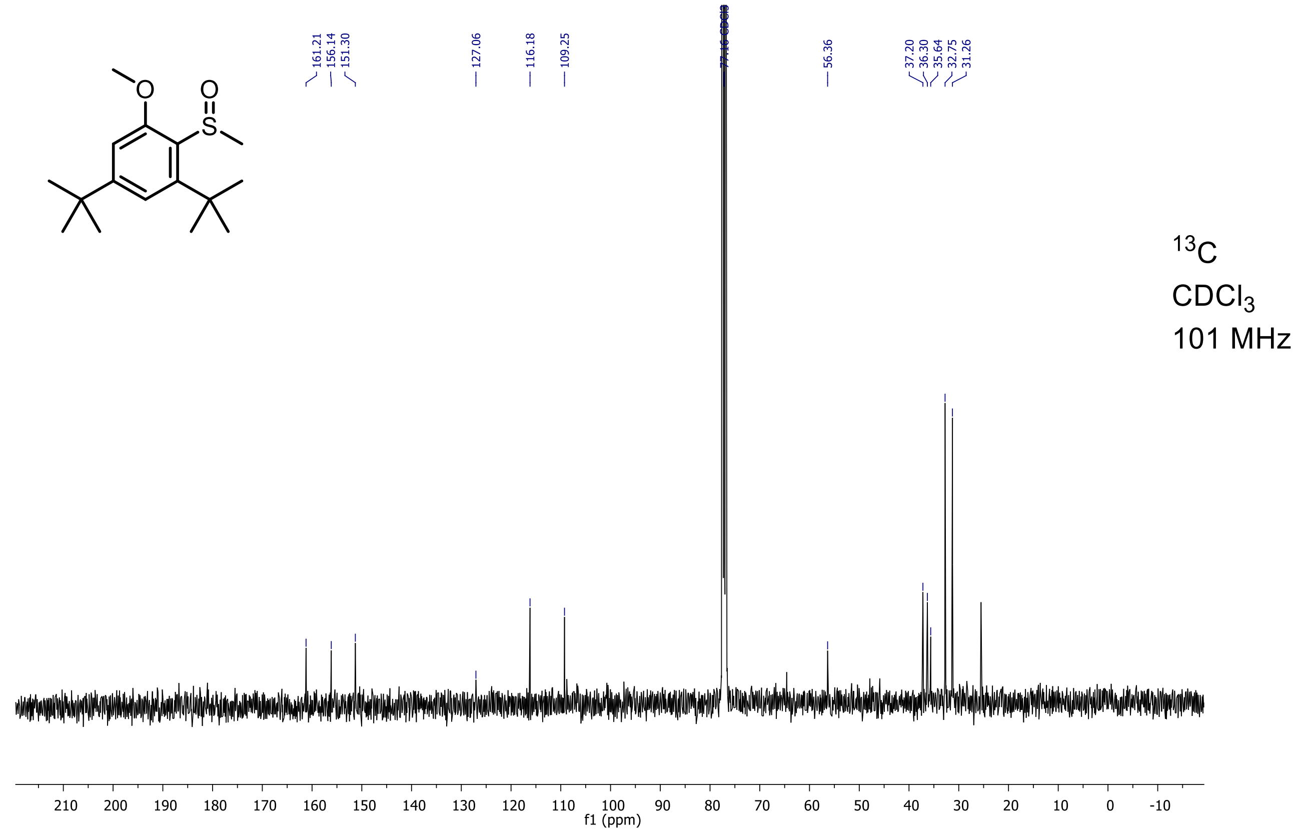

1h – 1,3-Diisopropyl-5-methoxy-2-(methylsulfinyl)benzene

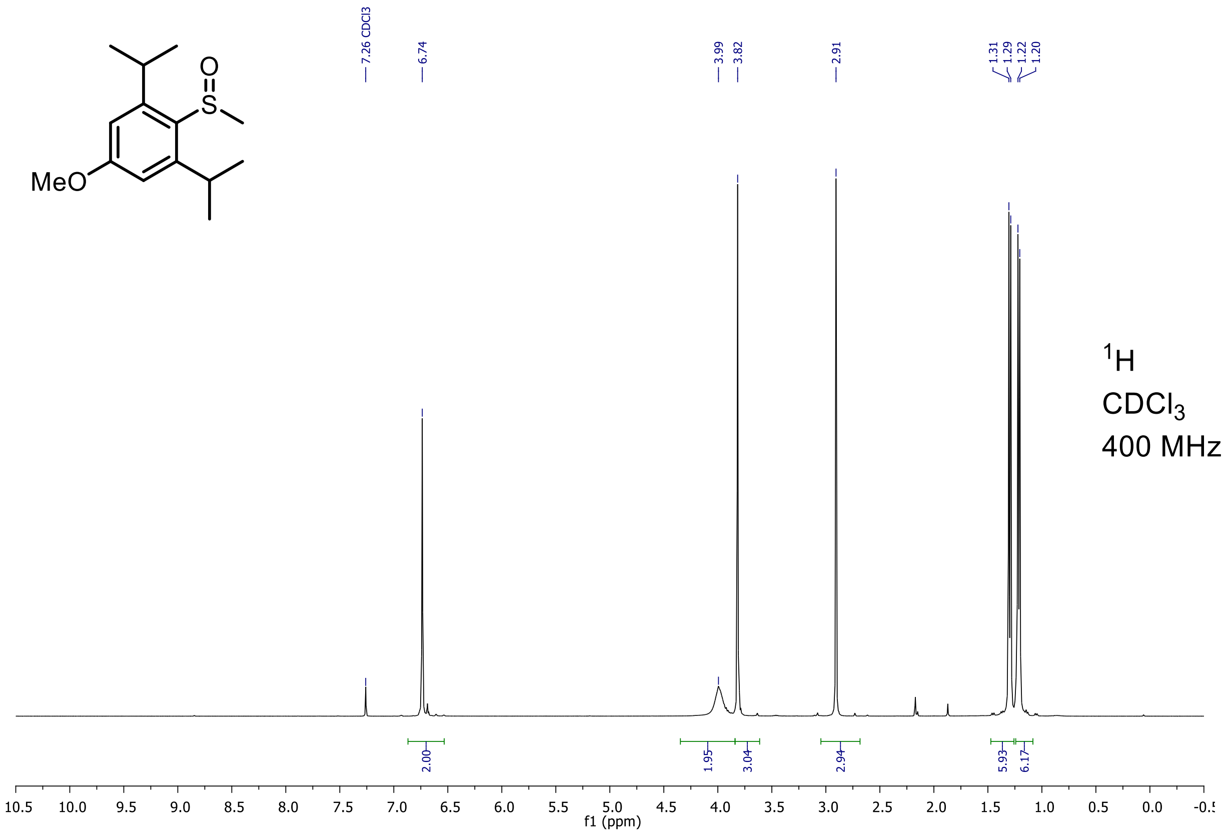

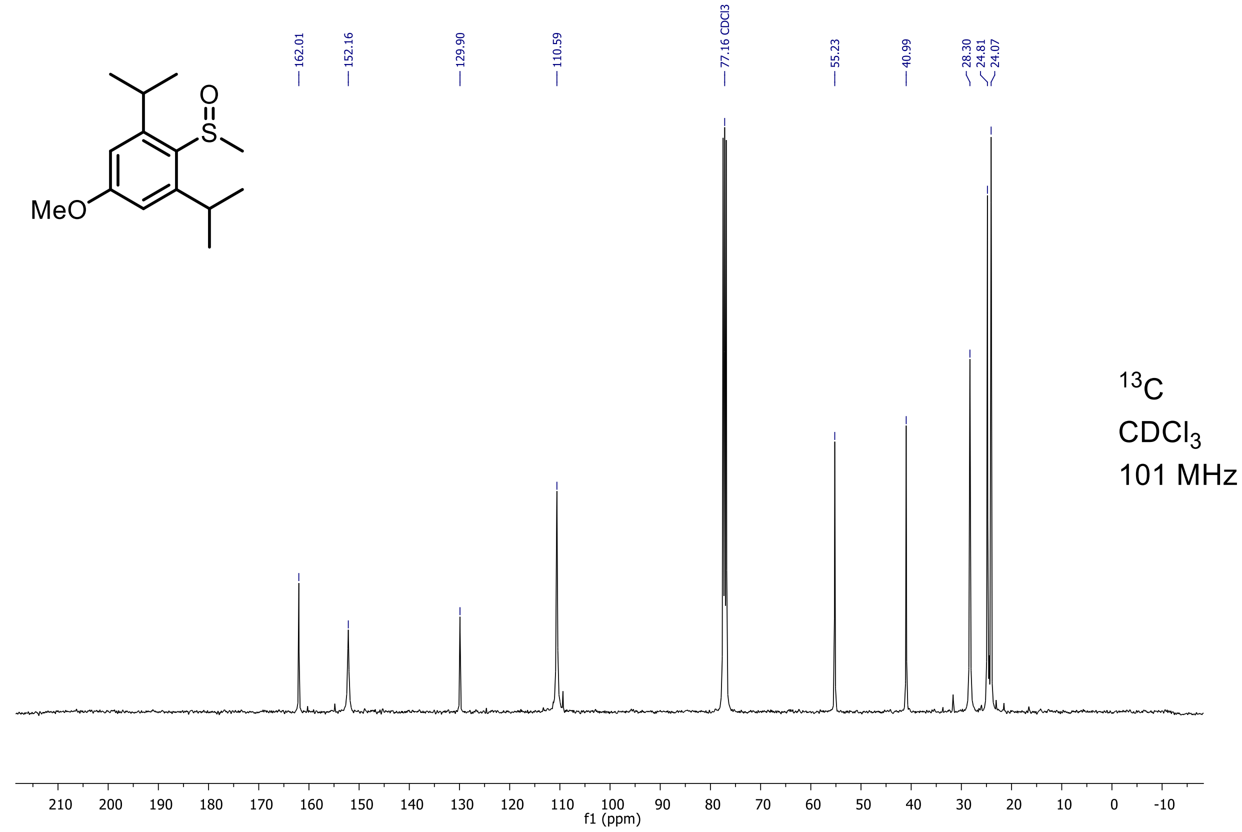

1i – 1-(Chloromethyl)-3,5-dimethoxy-2-(methylsulfinyl)benzene

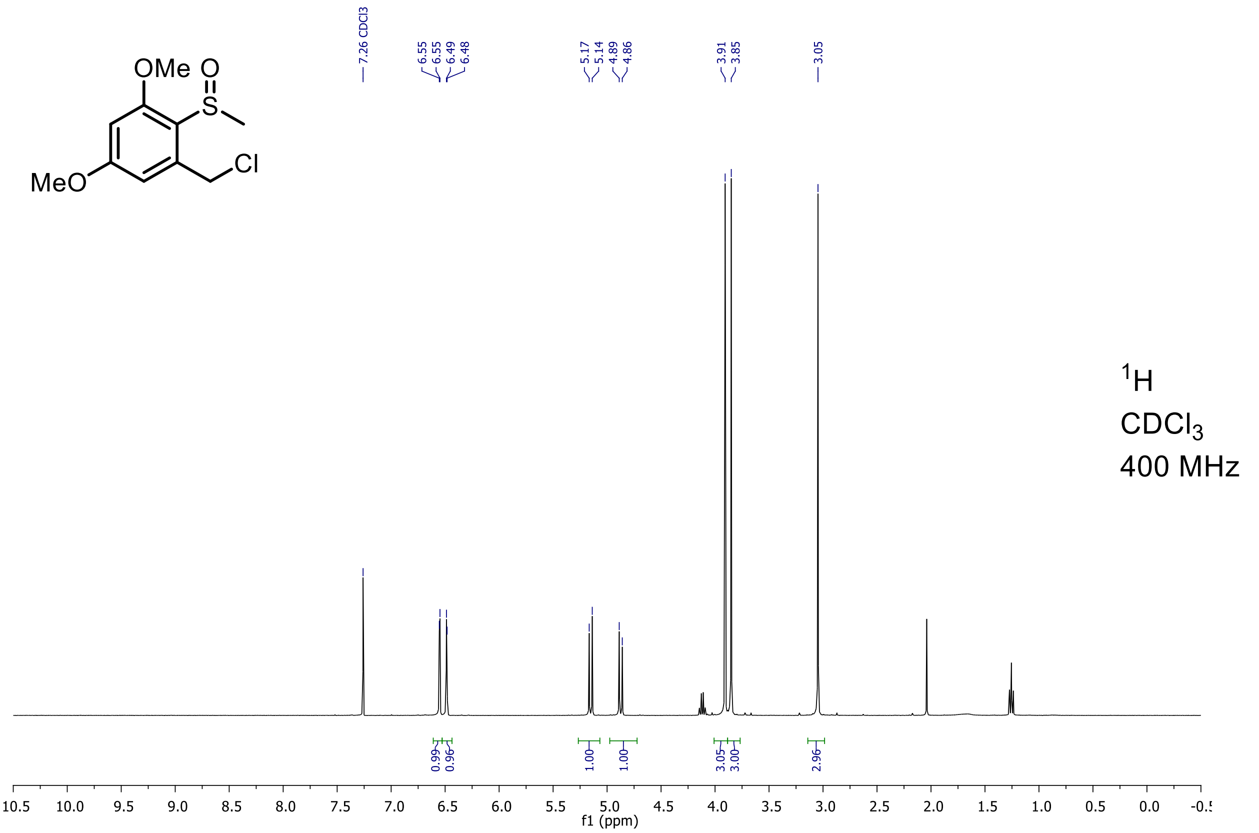

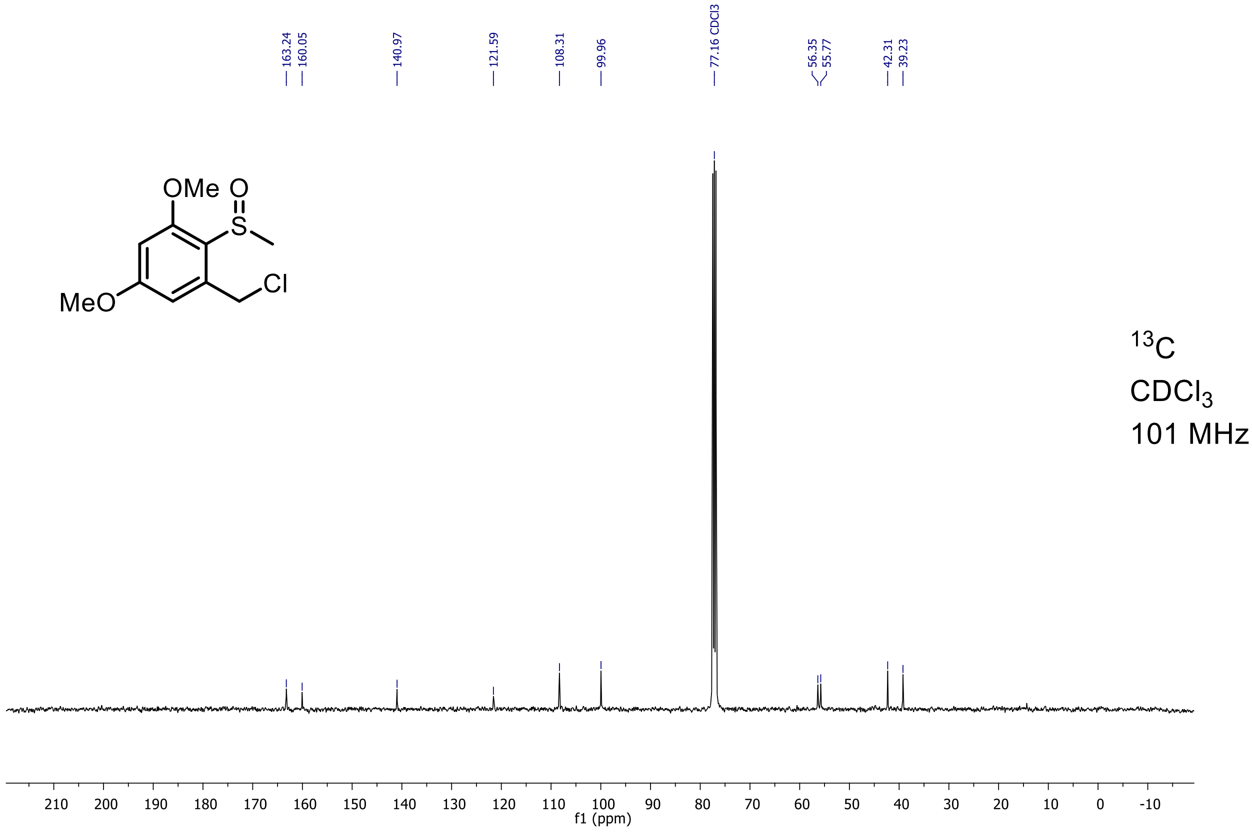

1m – 2,4-Bis(allyloxy)-1-(methylsulfinyl)benzene

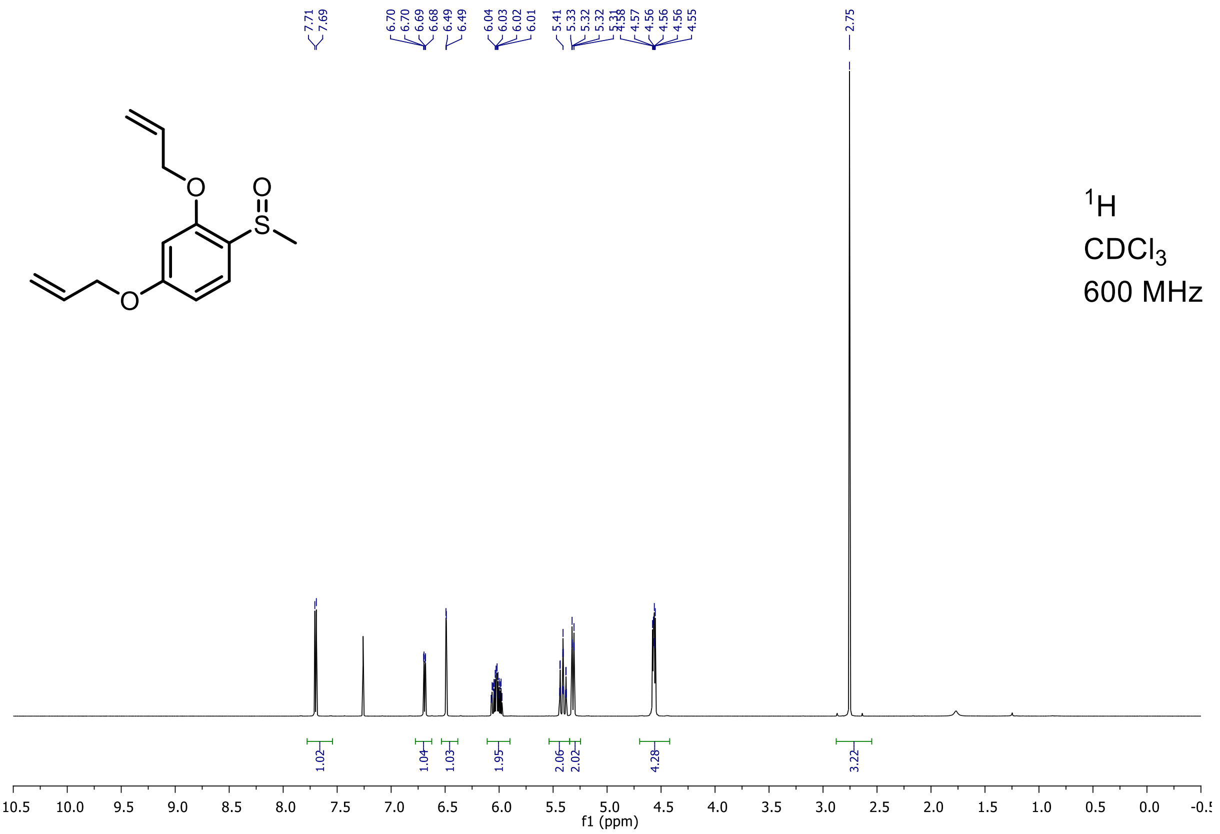

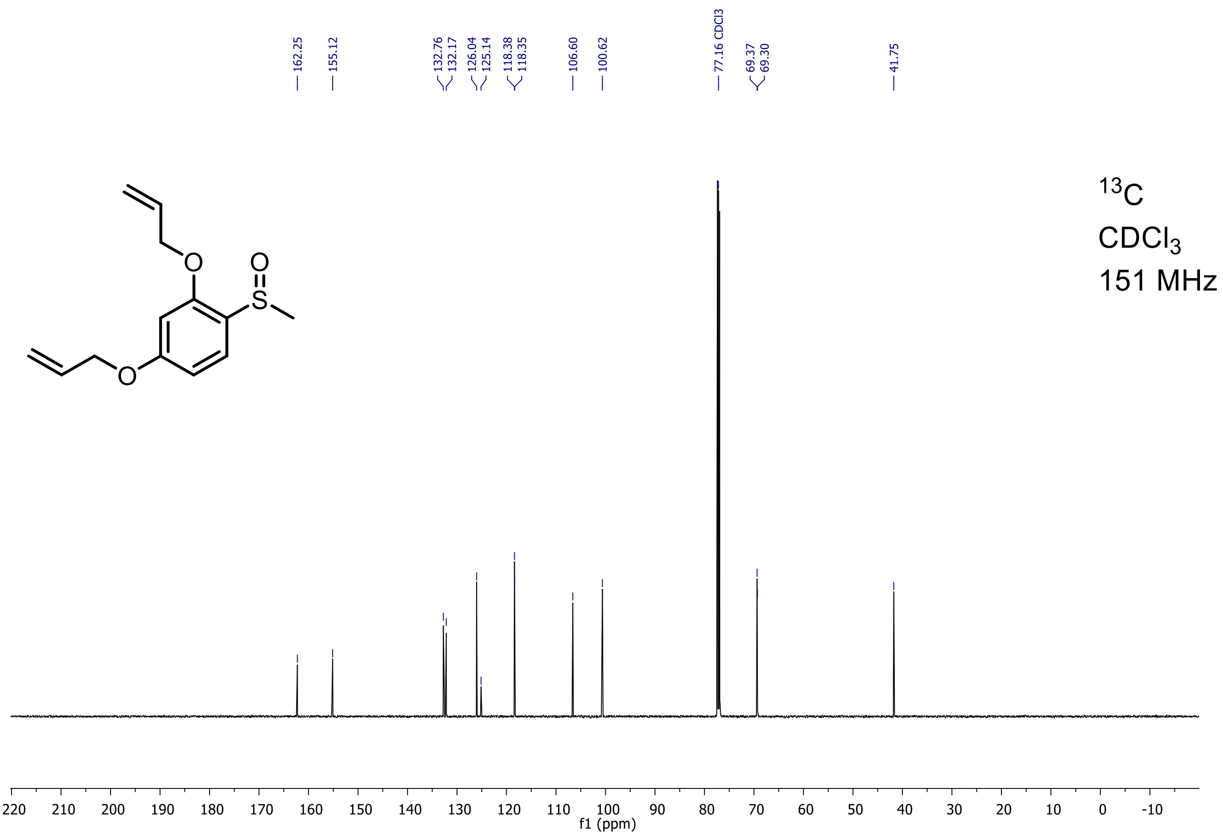

2l – *N*-Methoxy-*N*-methyl-11-(2-oxooxazolidin-3-yl)undec-10-ynamide

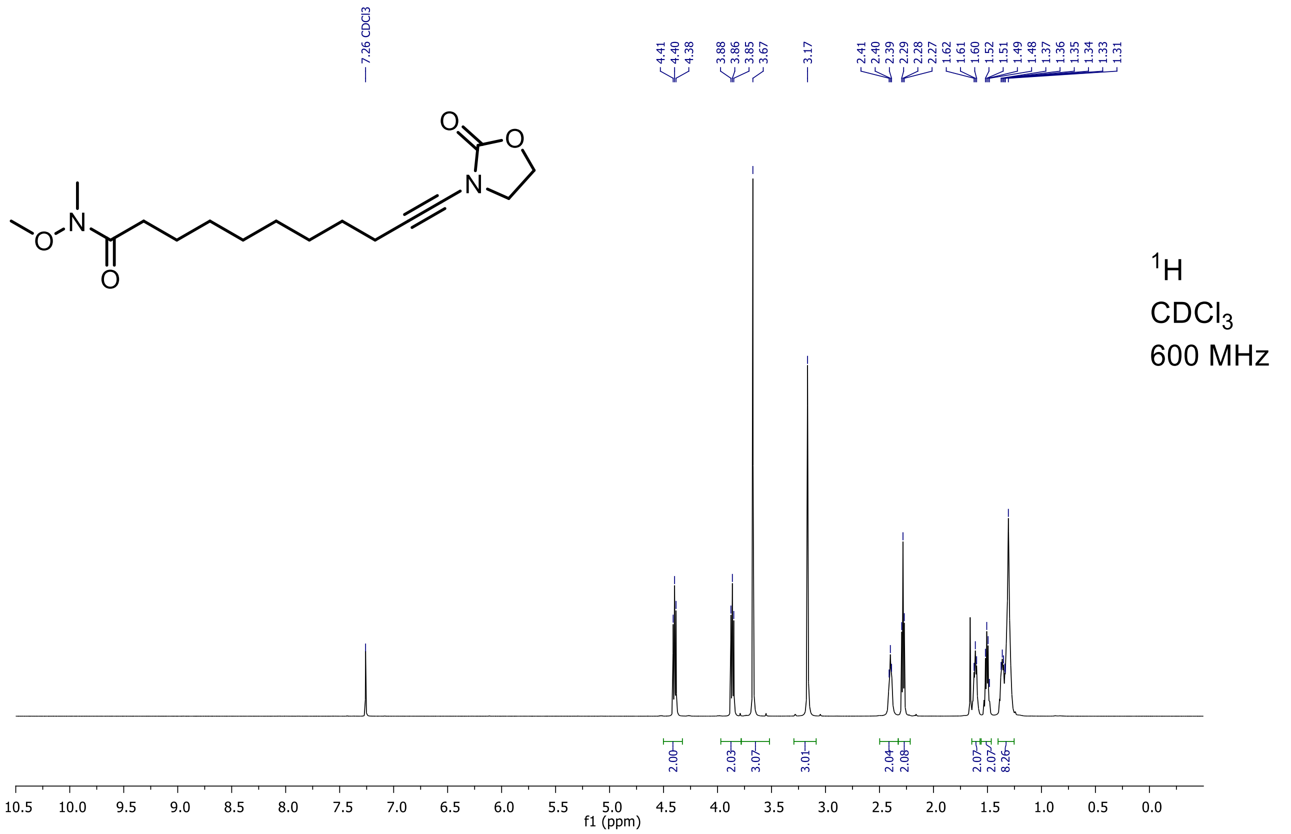

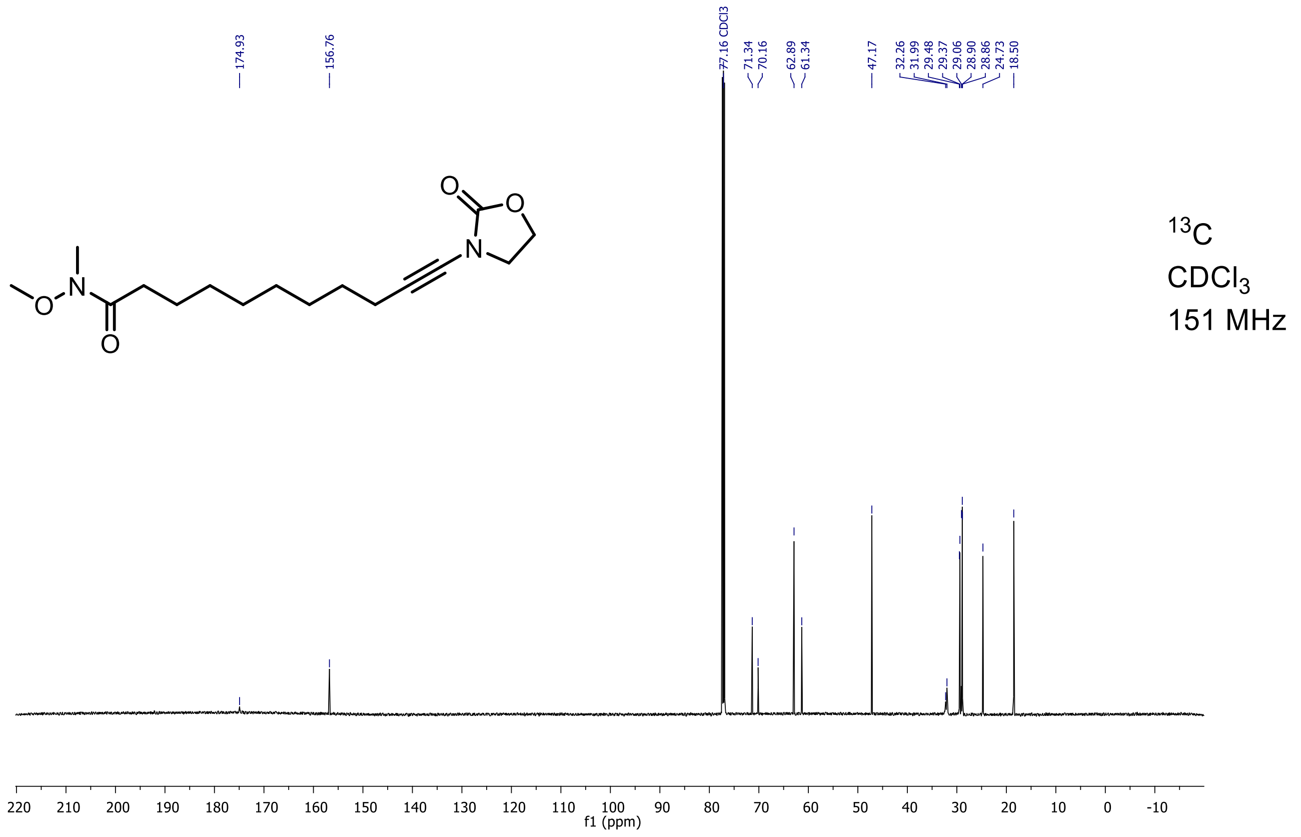

2J – Benzyl (*S*)-2-((*tert*-butoxycarbonyl)amino)-6-(2-oxooxazolidin-3-yl)hex-5-ynoate

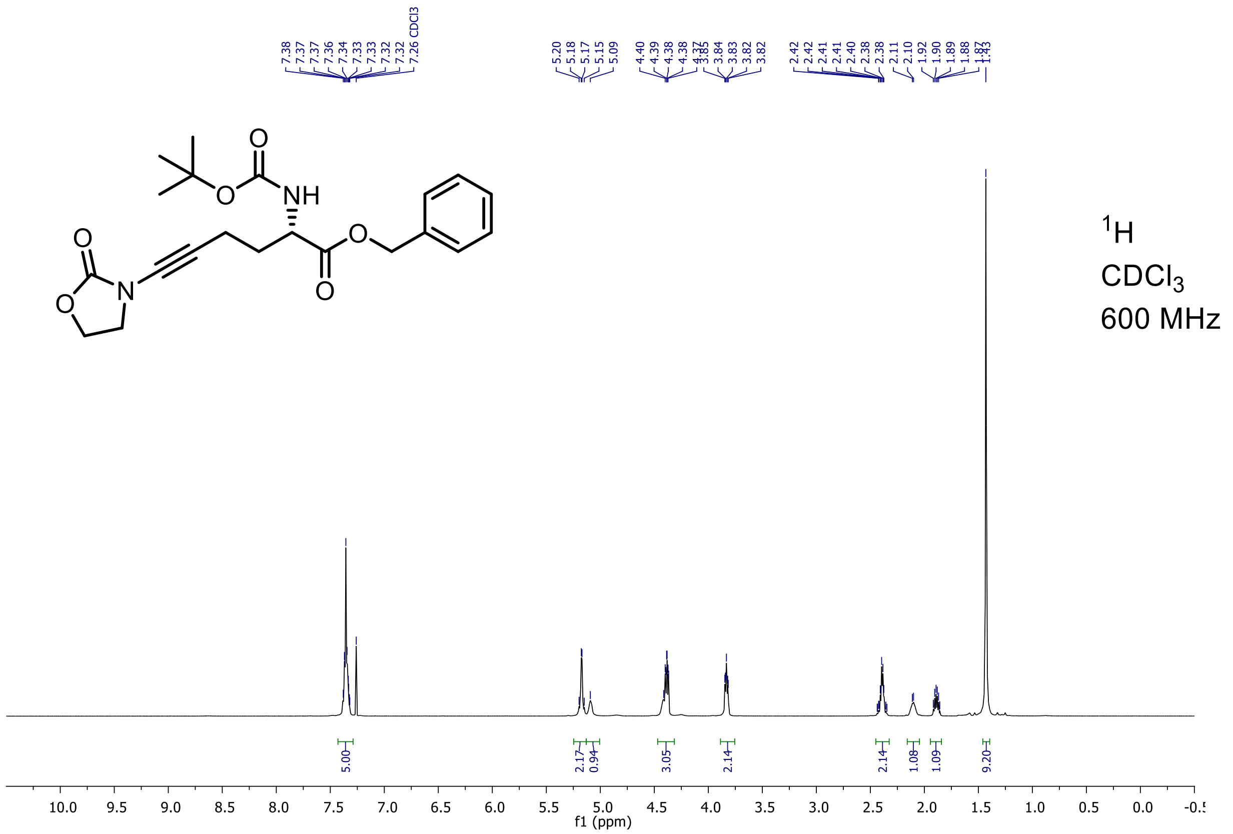

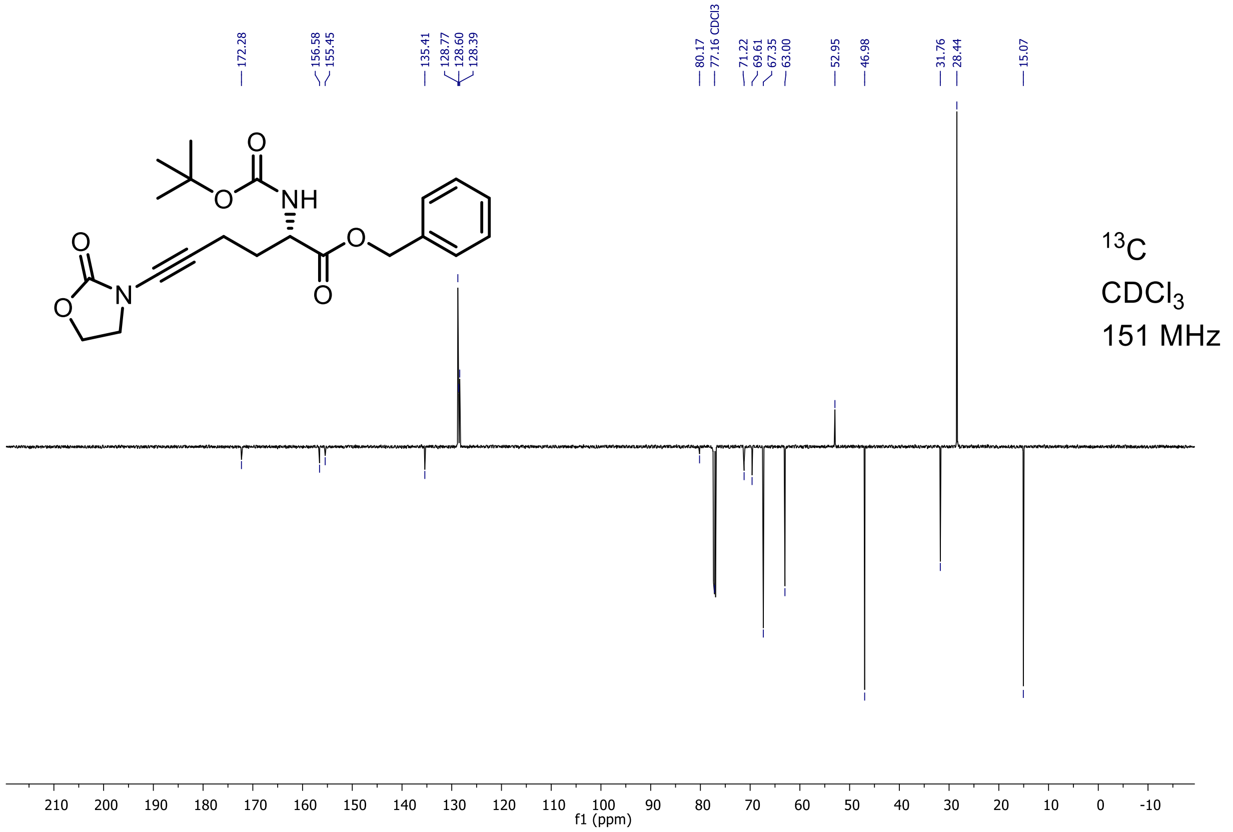

2K – *N*,4-Dimethyl-*N*-(5-phenylpent-1-yn-1-yl)benzenesulfonamide

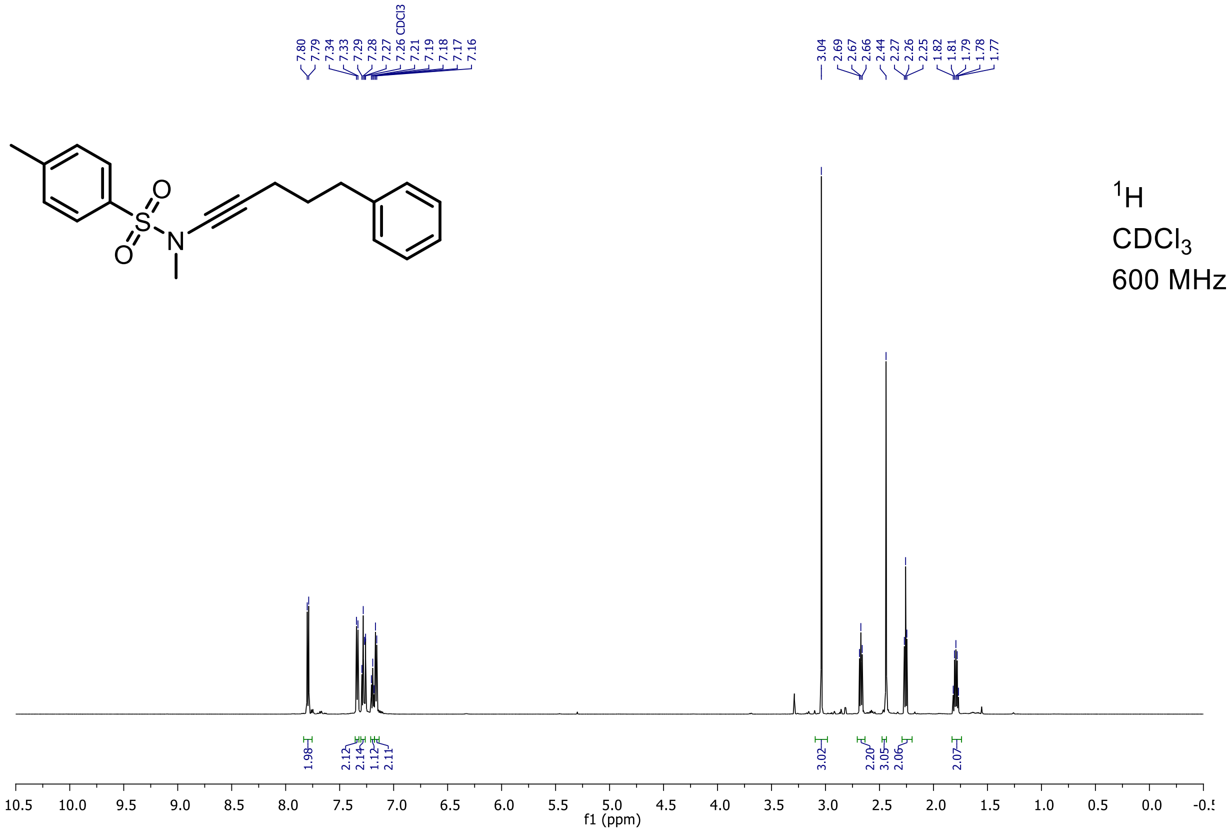

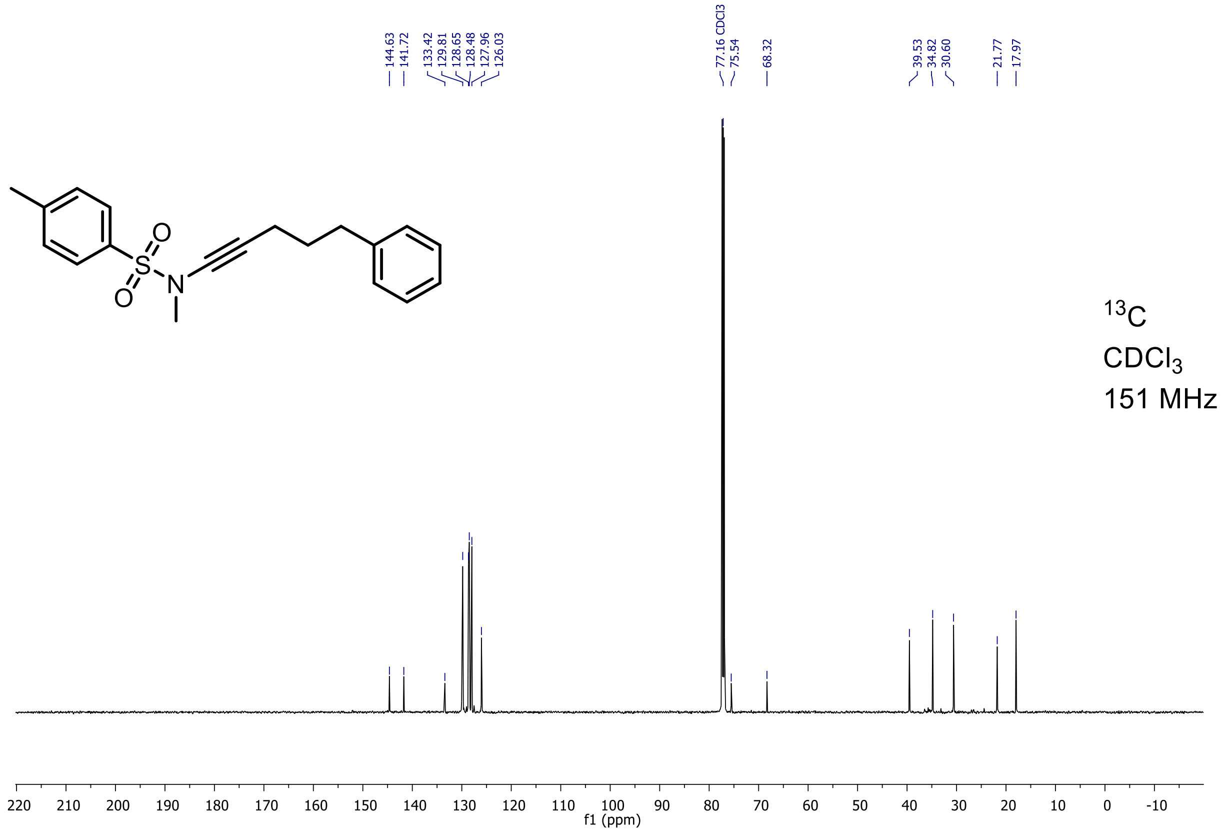

3G – (Cyclopentylethynyl)(phenyl)sulfane

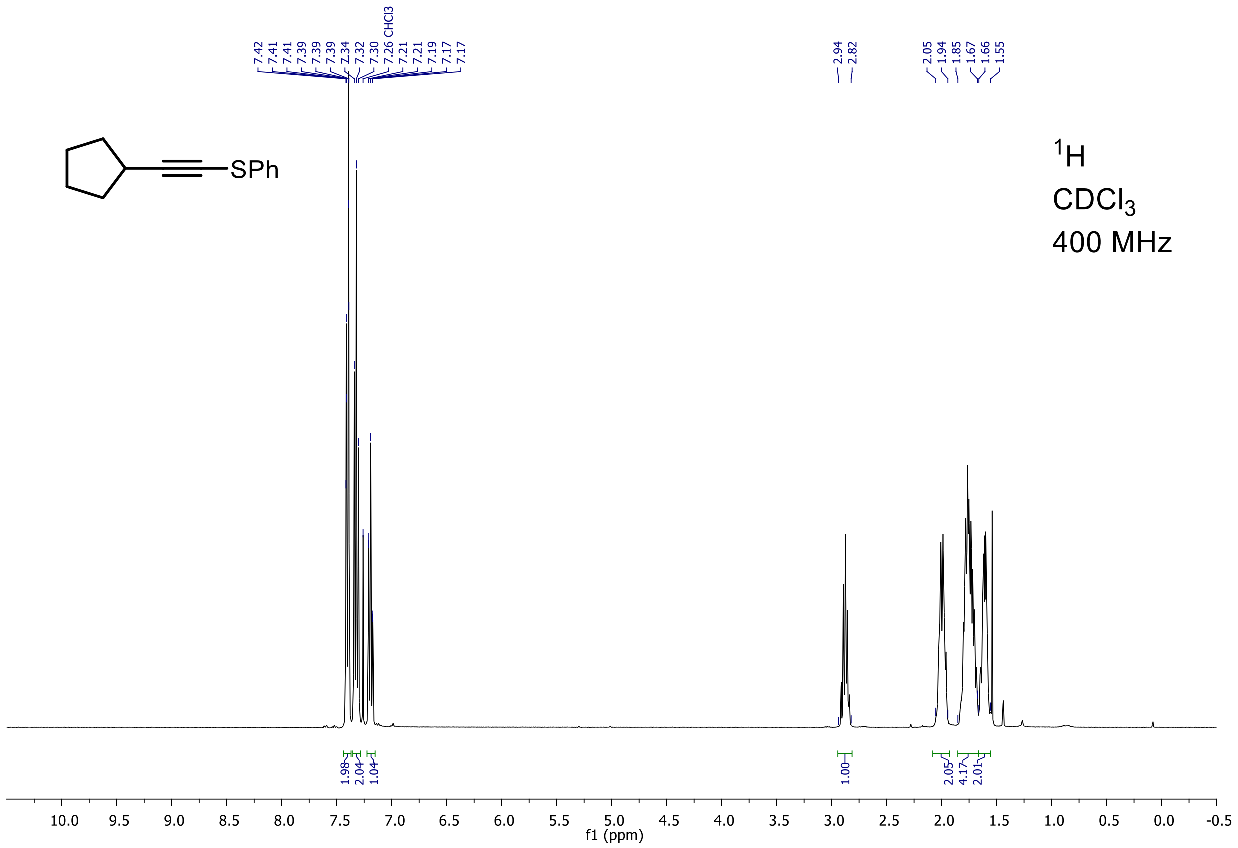

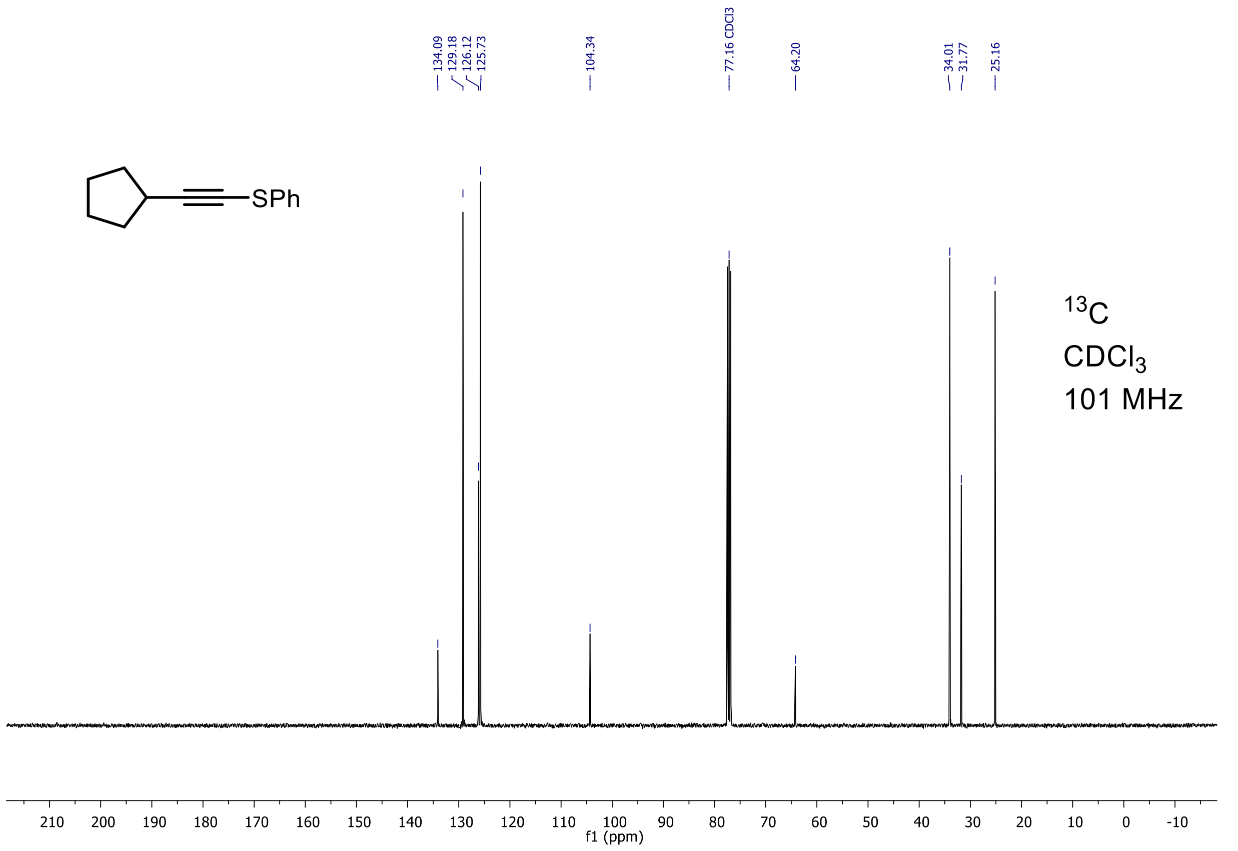

3H – Phenyl(5-phenylpent-1-yn-1-yl)sulfane

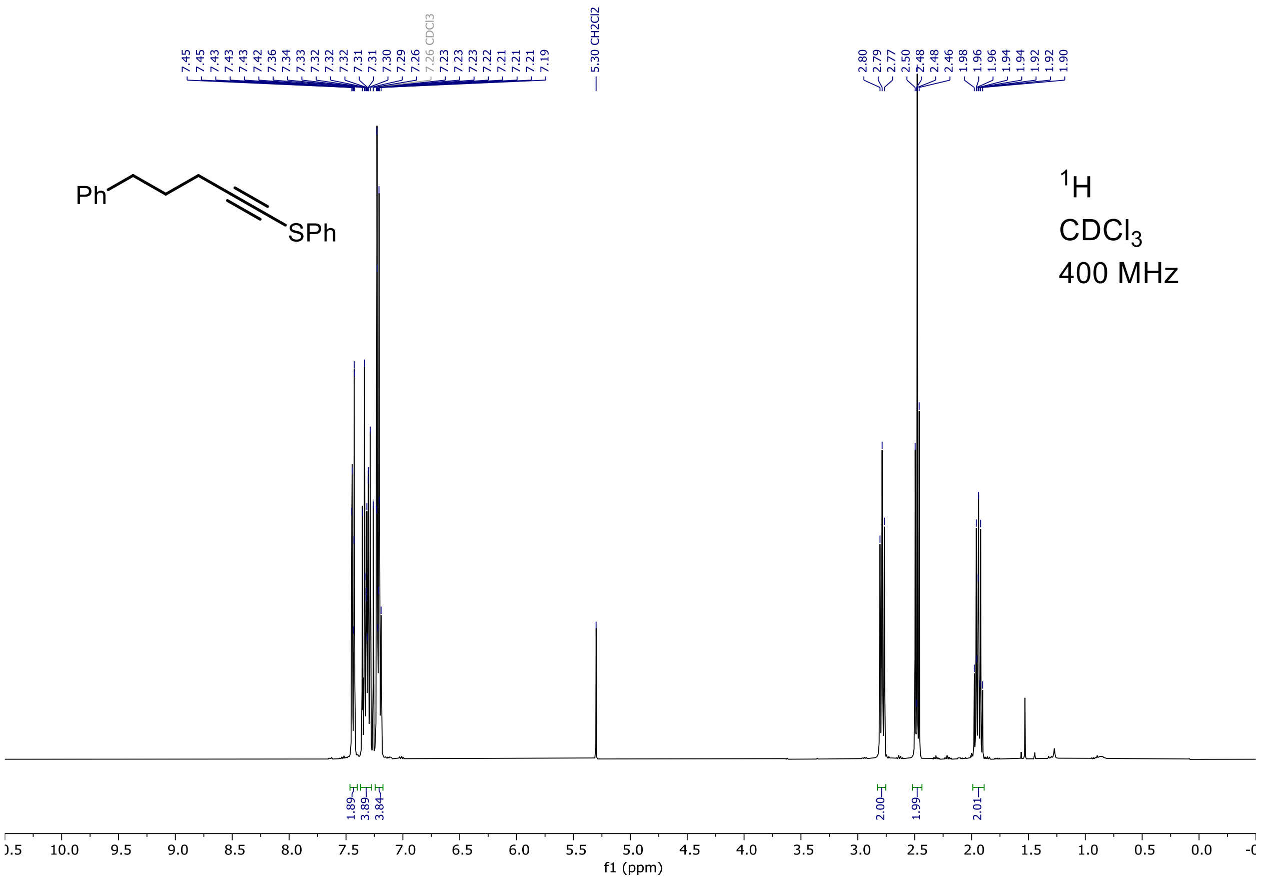

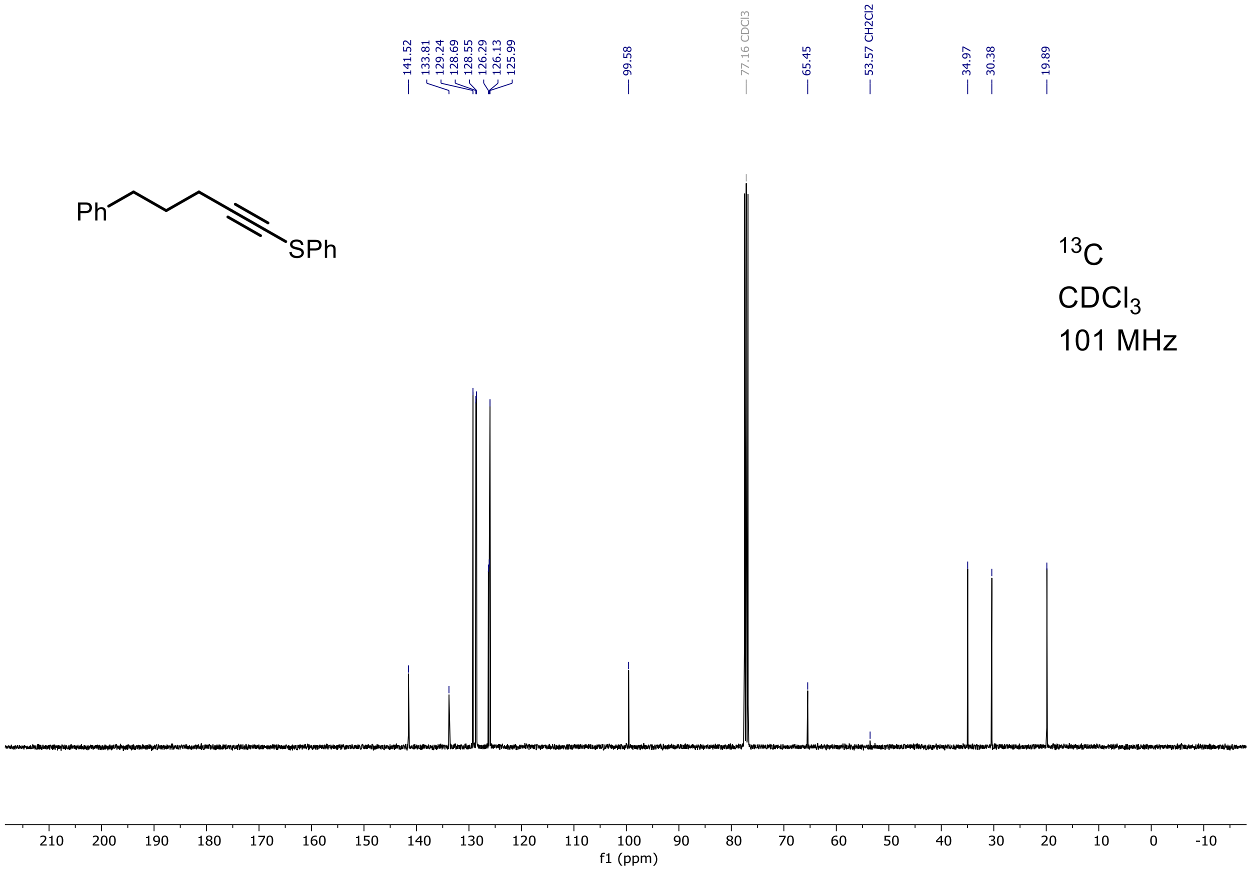

4aA – 3-(2-(2,6-Dimethoxyphenyl)hexanoyl)oxazolidin-2-one

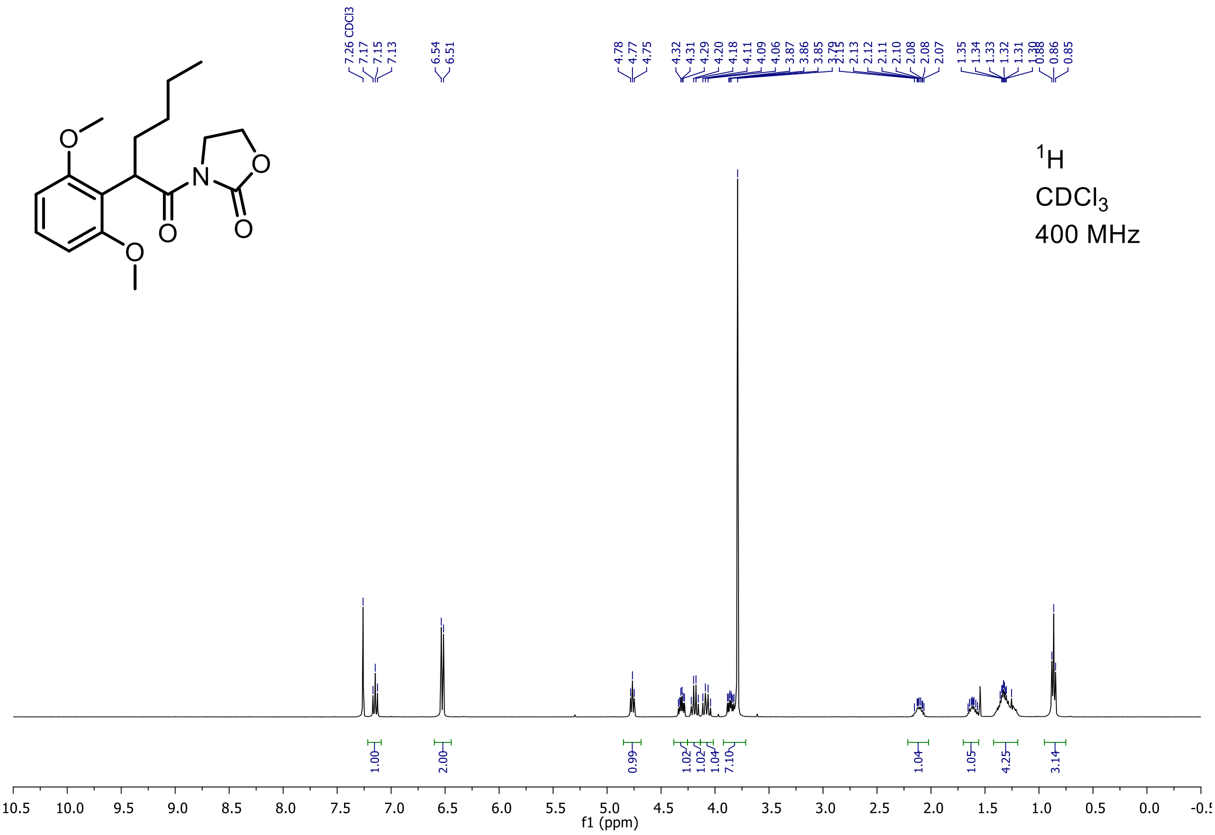

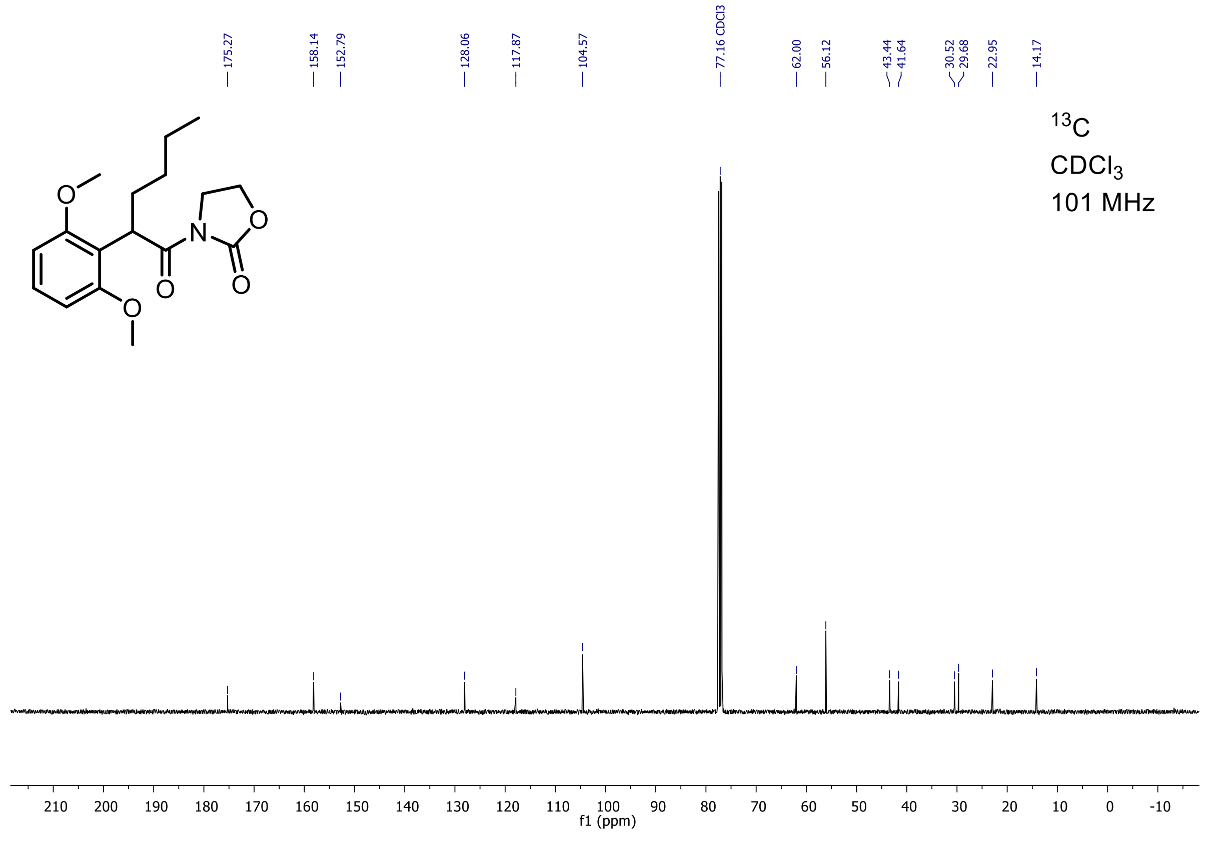

4bA – 3-(2-(2,4,6-Trimethoxyphenyl)hexanoyl)oxazolidin-2-one

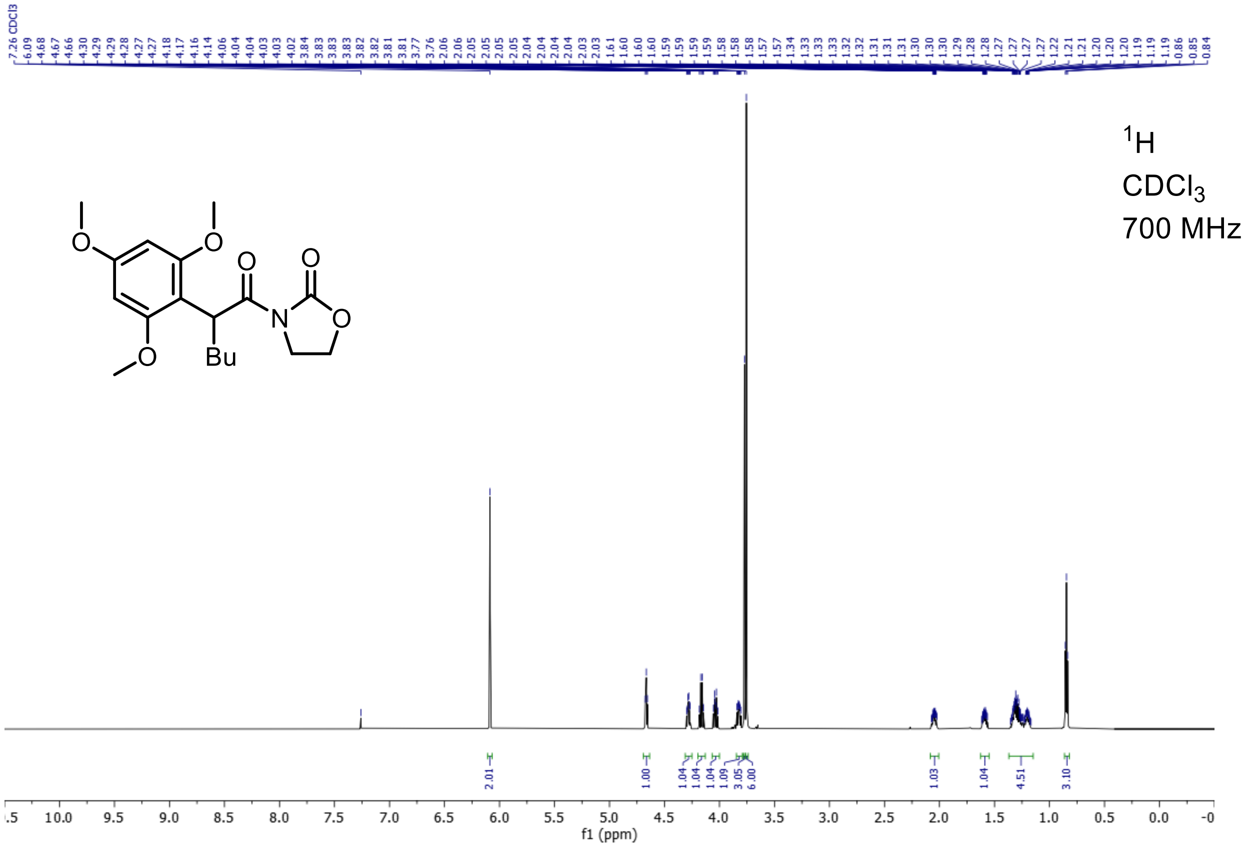

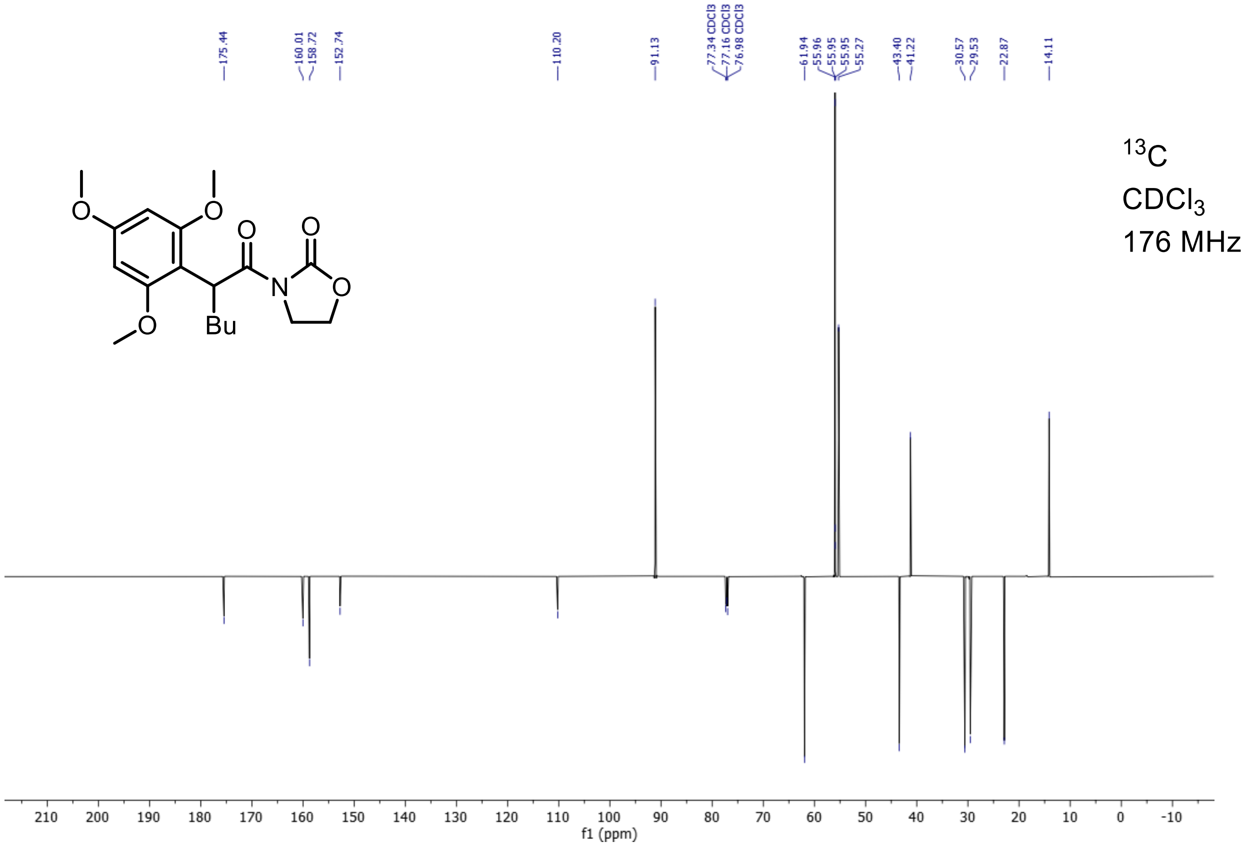

4cA – 3-(2-(2-Chloro-6-methoxyphenyl)hexanoyl)oxazolidin-2-one

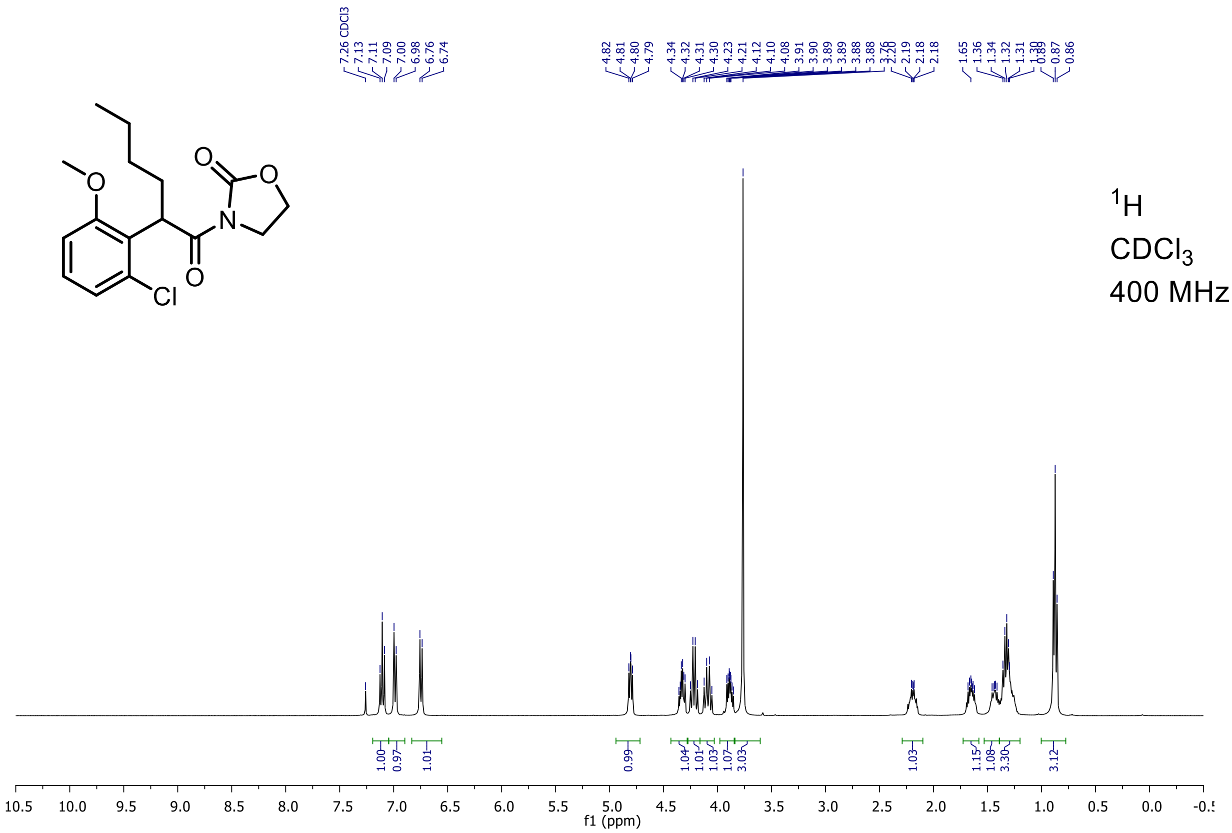

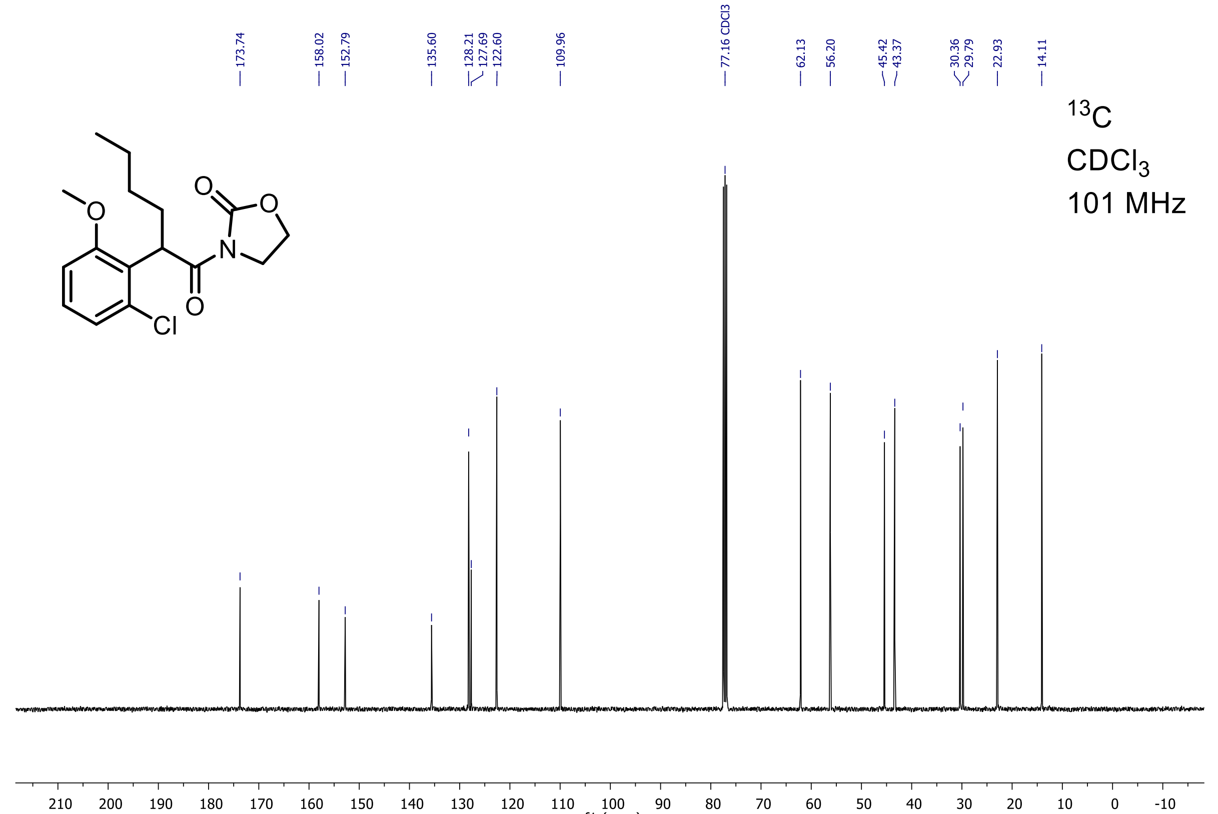

4dA – 3-(2-(2,4-Dichloro-6-methoxyphenyl)hexanoyl)oxazolidin-2-one

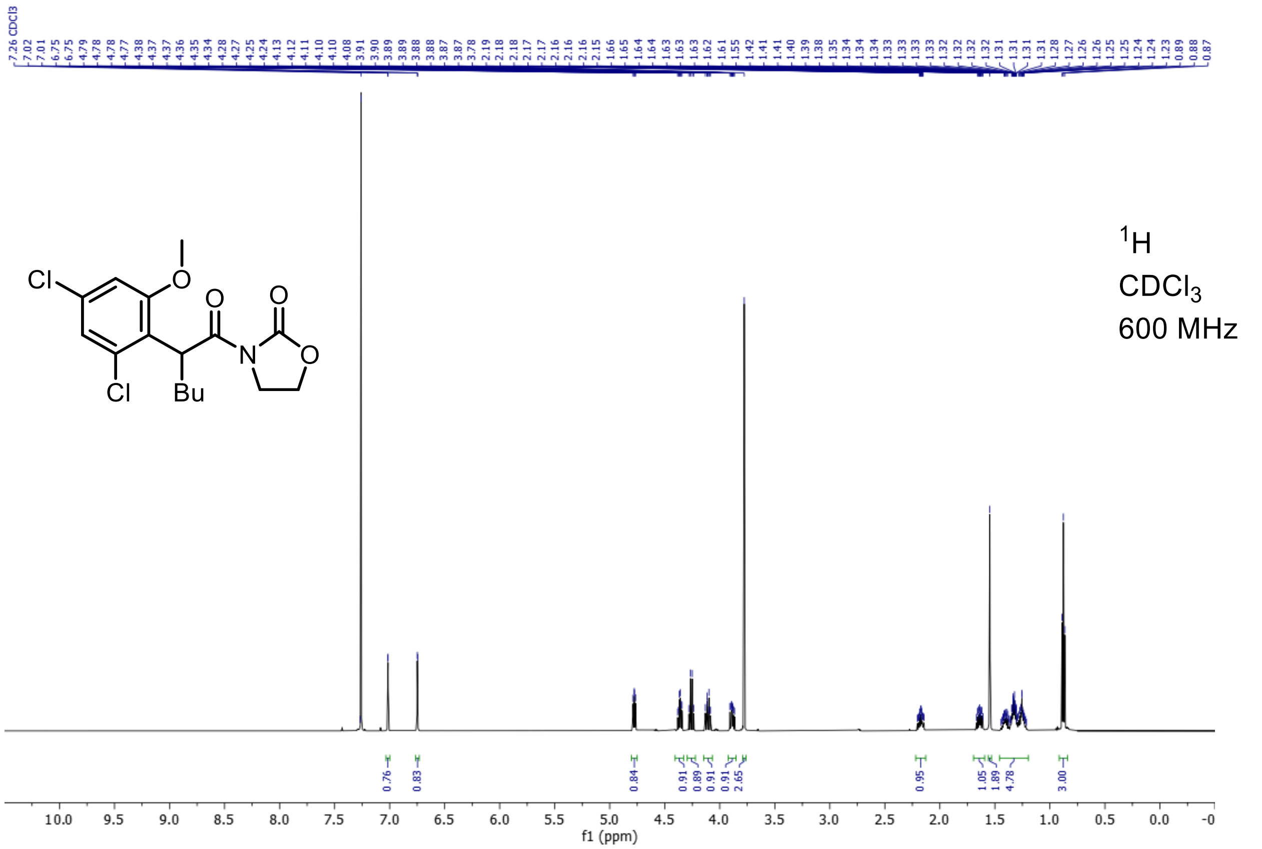

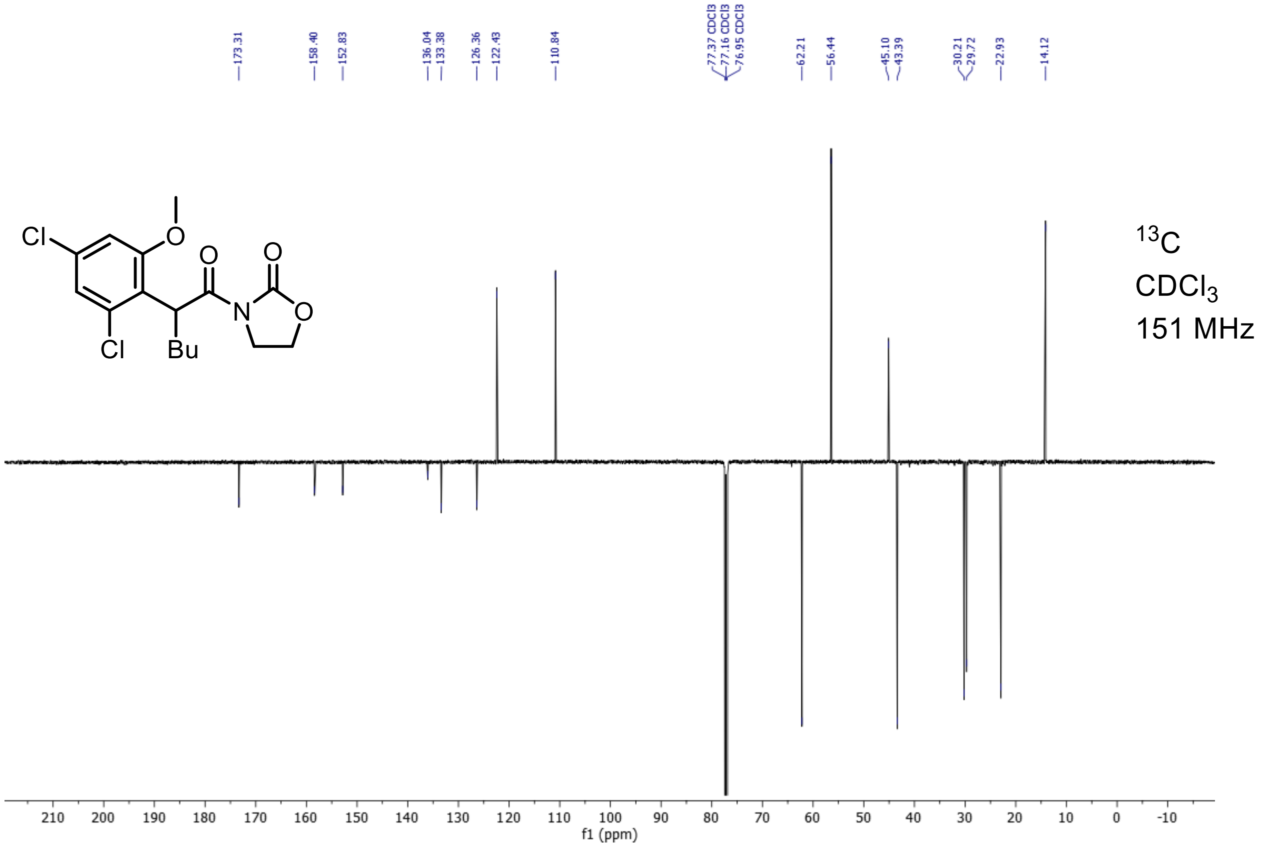

4eA – 3-(2-(2-Bromo-4,6-dimethoxyphenyl)hexanoyl)oxazolidin-2-one

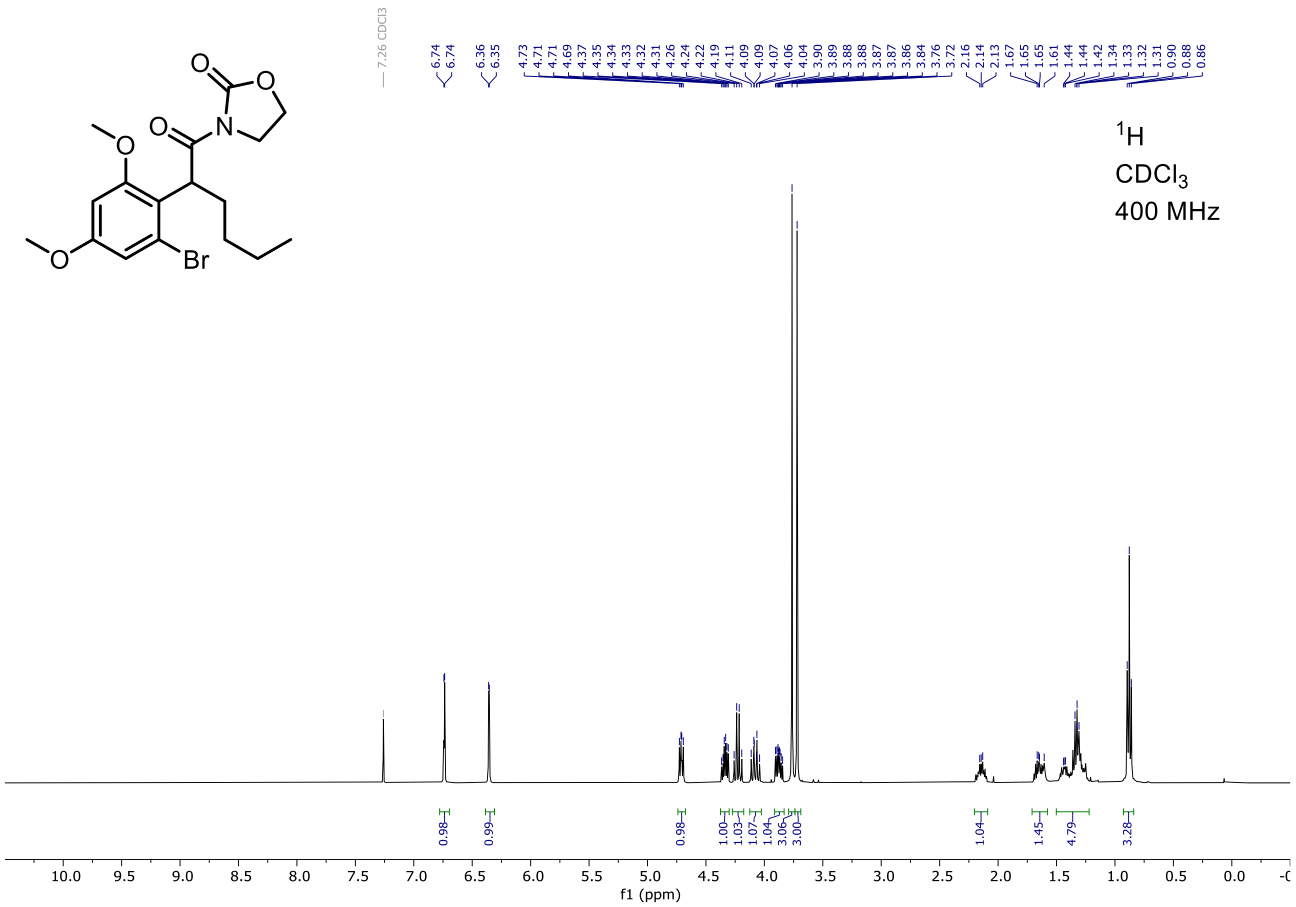

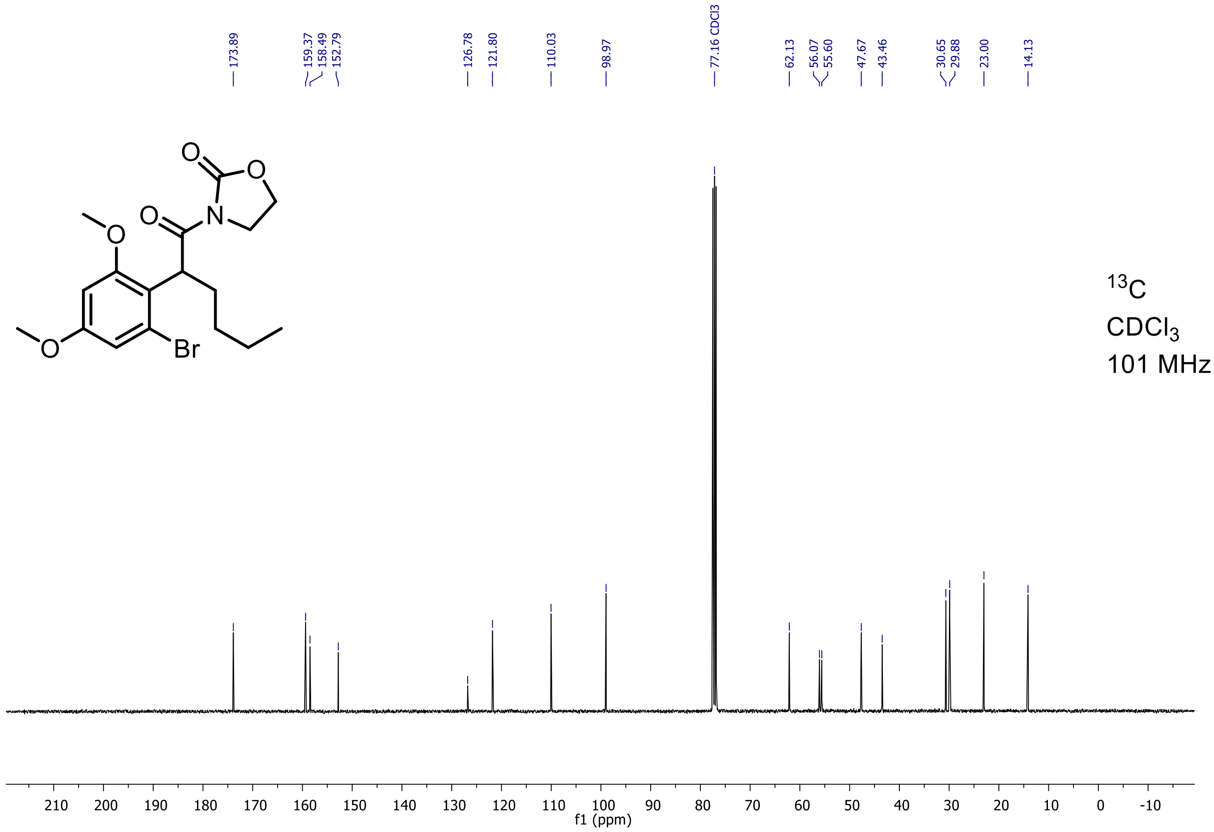

4fA – 3-(2-(4-Methoxy-2,6-dimethylphenyl)hexanoyl)oxazolidin-2-one

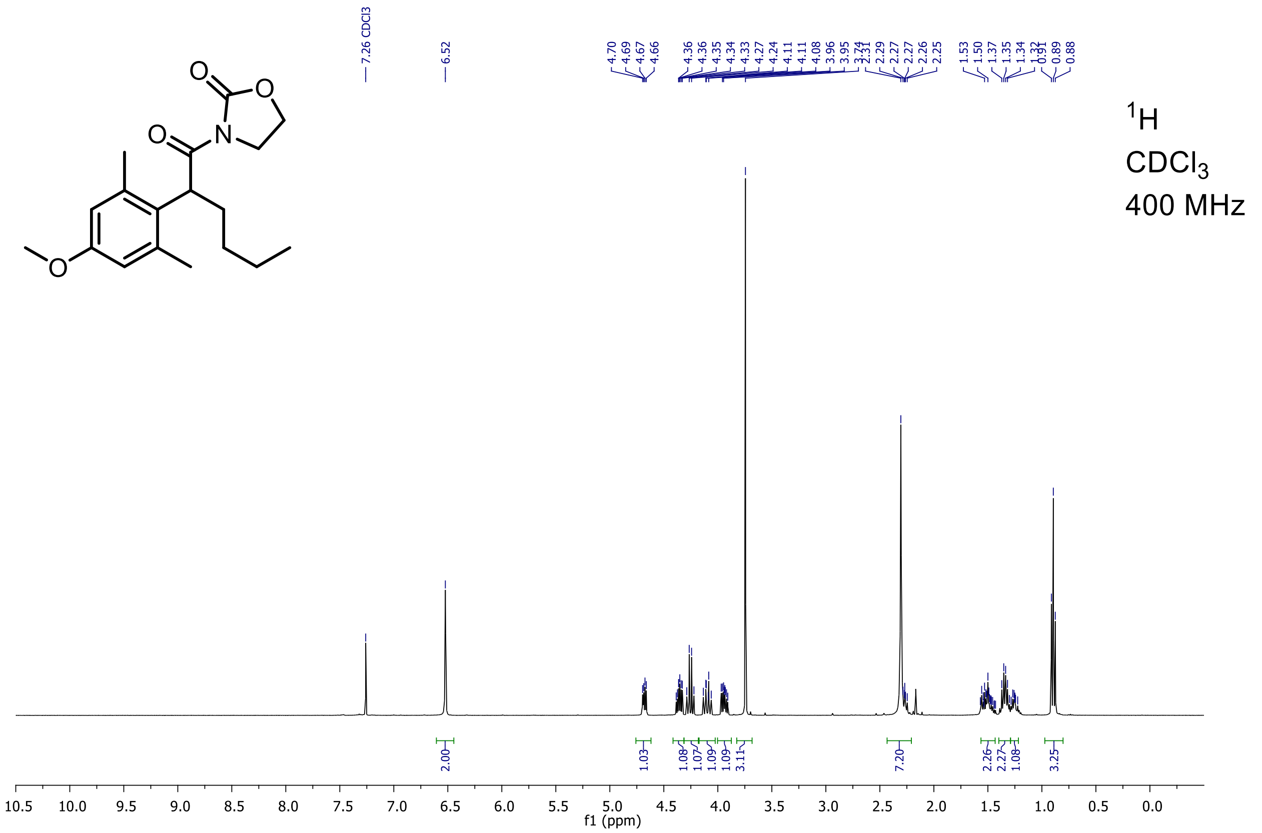

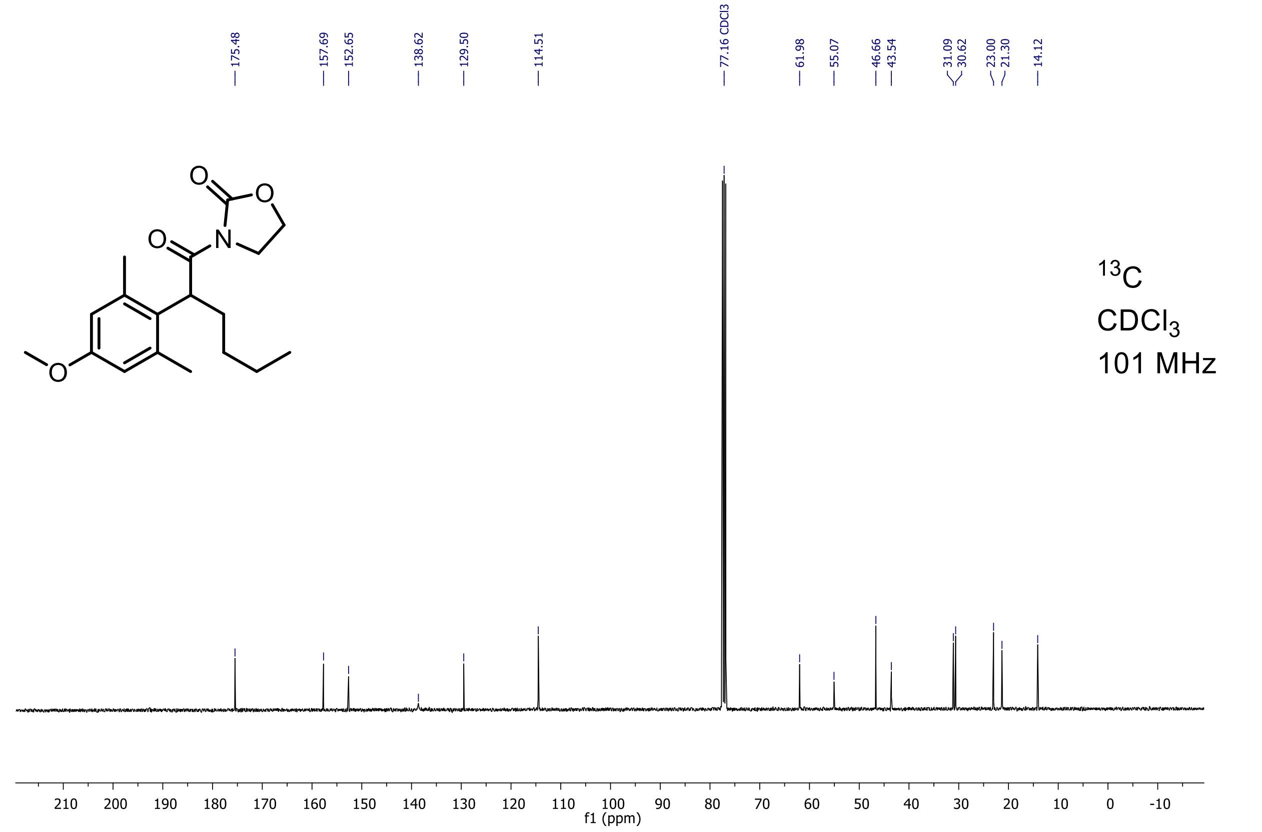

4gA – 3-(2-(2,4-Di-*tert*-butyl-6-methoxyphenyl)hexanoyl)oxazolidin-2-one

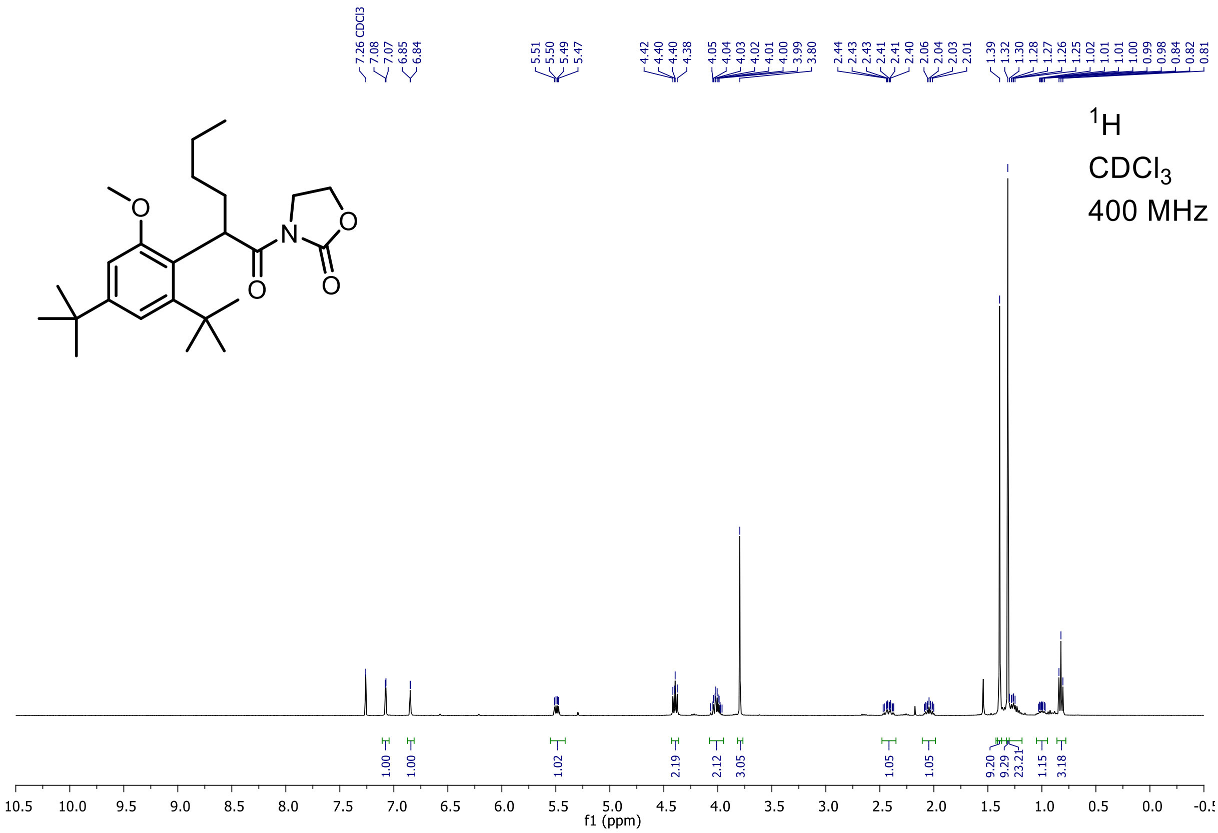

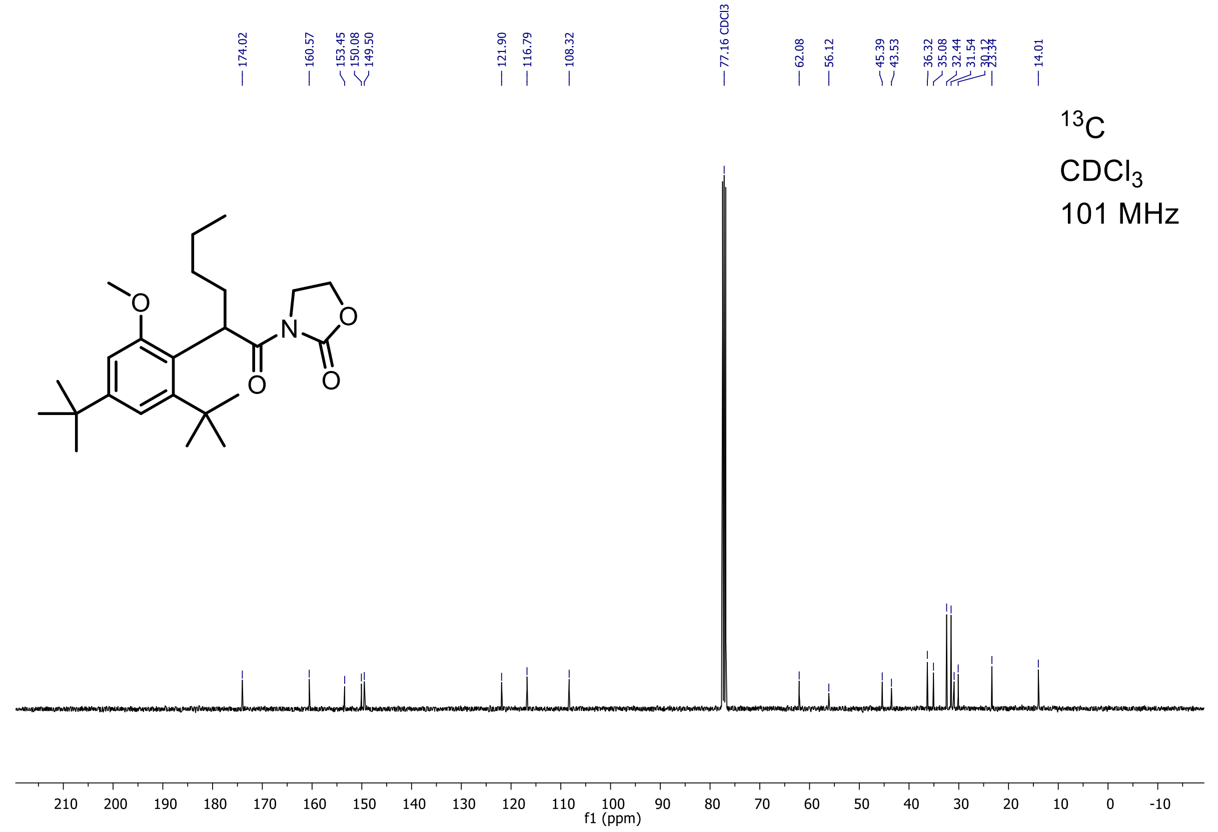

4hA – 3-(2-(2,6-Diisopropyl-4-methoxyphenyl)hexanoyl)oxazolidin-2-one

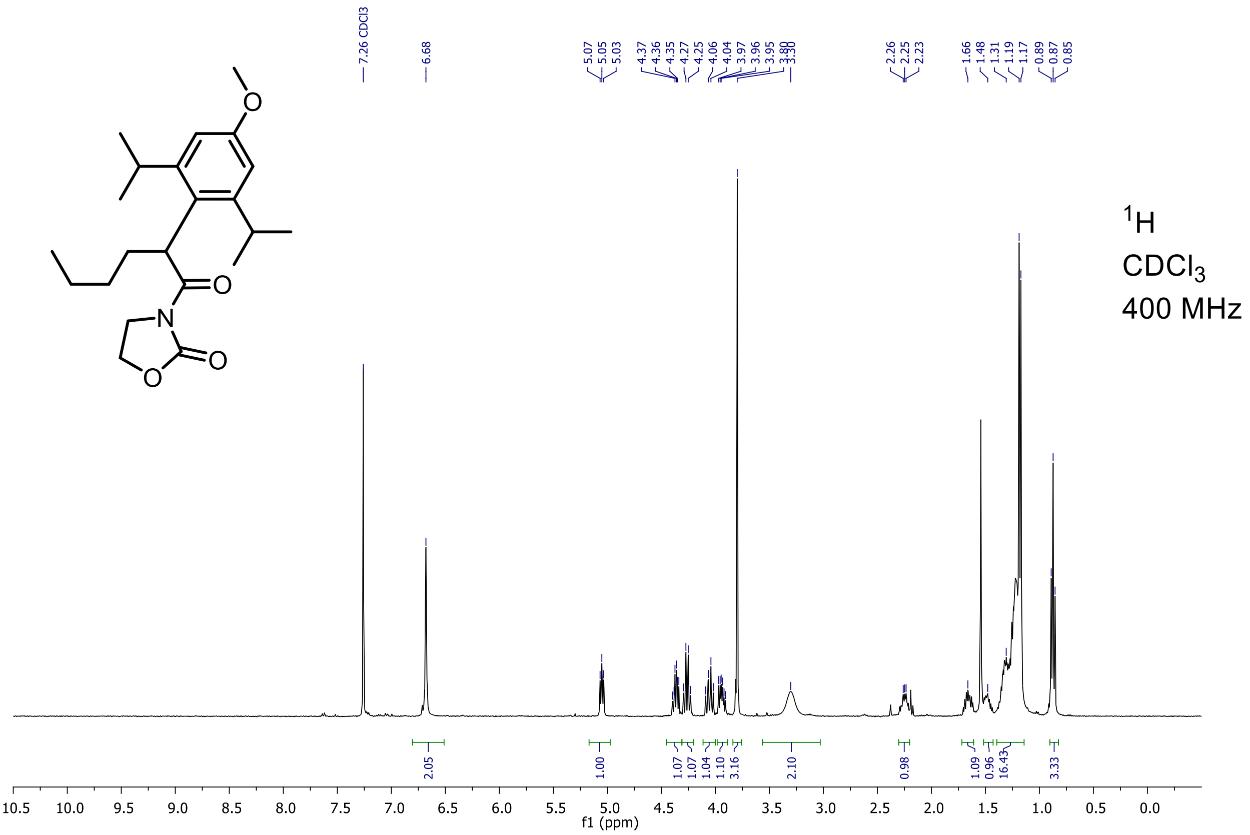

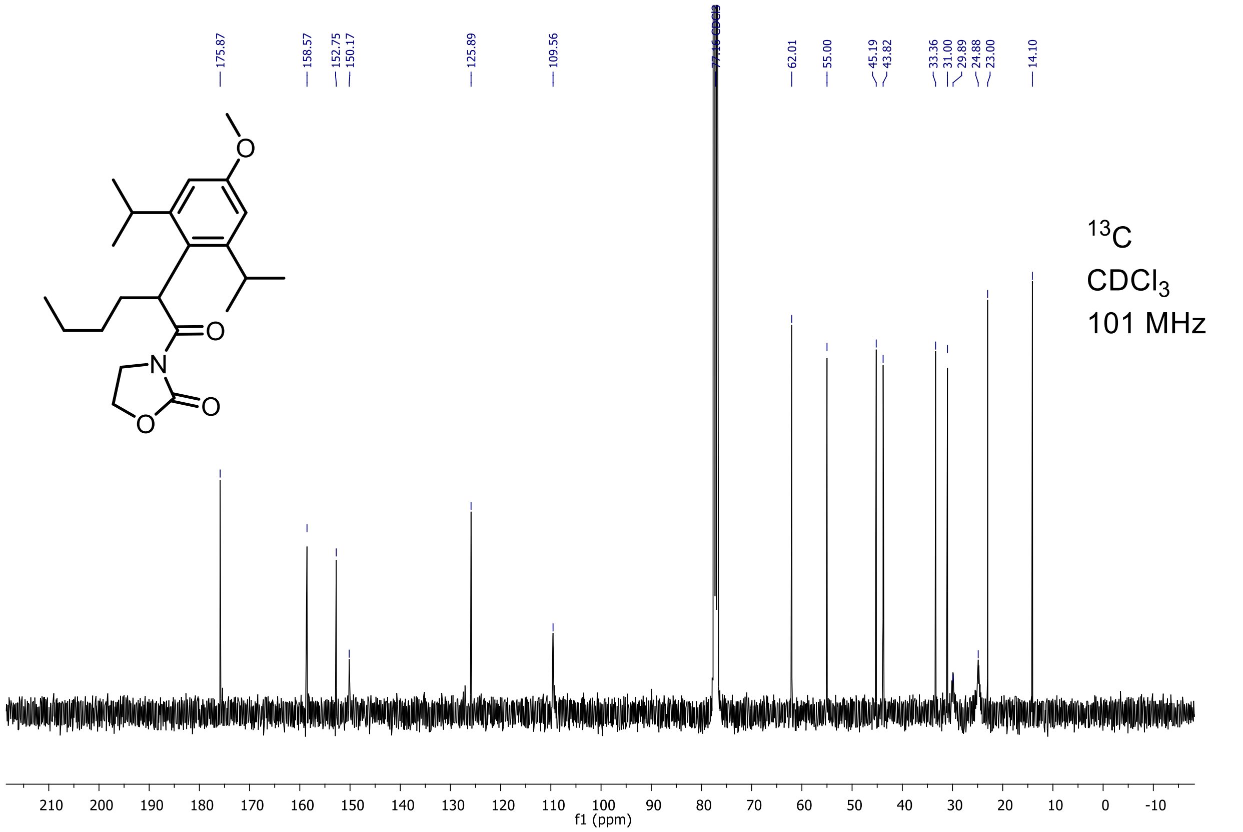

4iA – 3-(2-(2-(Chloromethyl)-4,6-dimethoxyphenyl)hexanoyl)oxazolidin-2-one

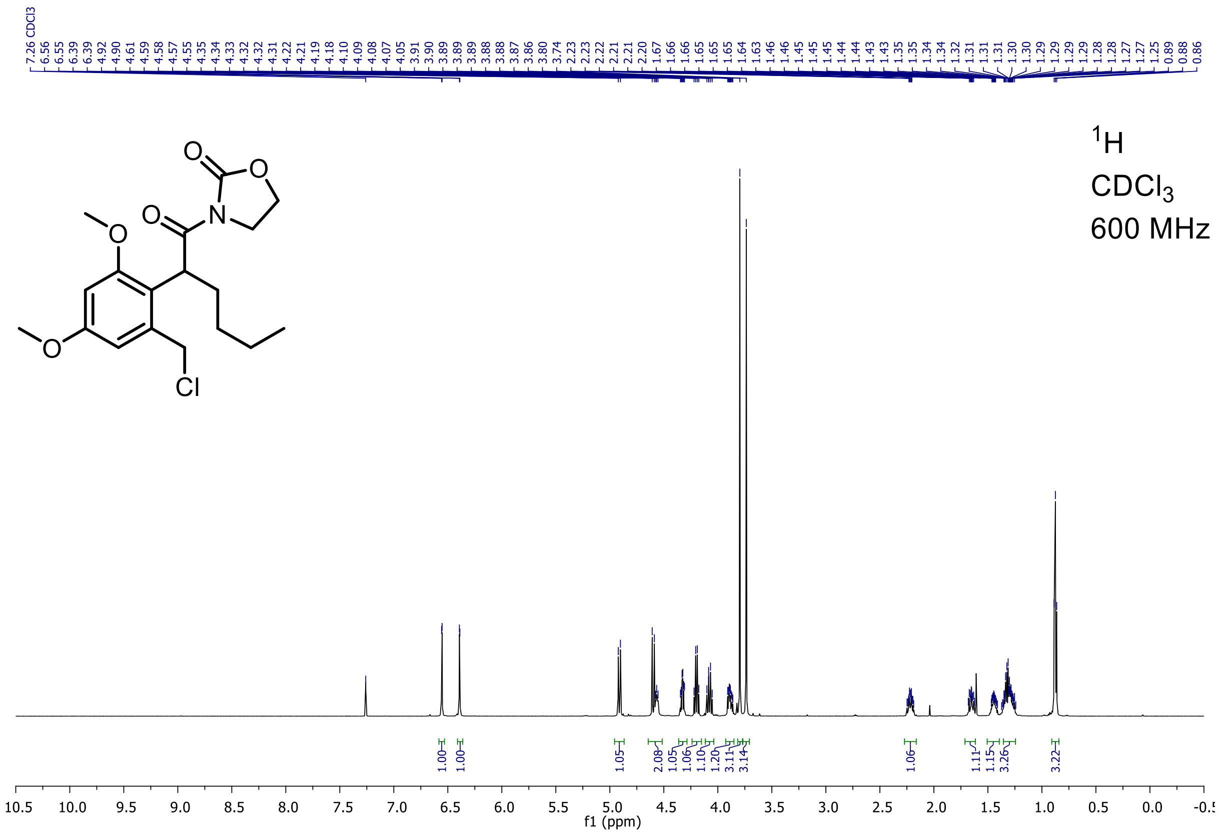

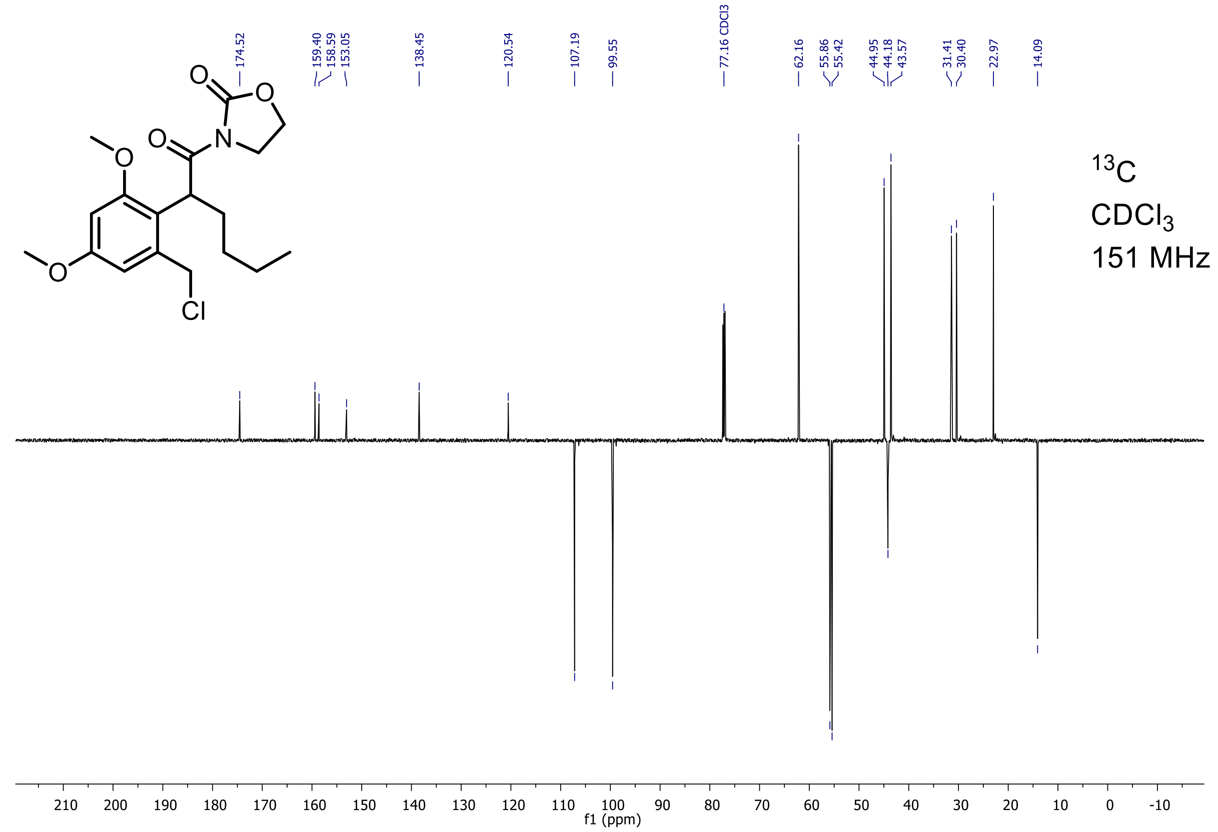

4jA – 3-(2-(2-Methoxynaphthalen-1-yl)hexanoyl)oxazolidin-2-one

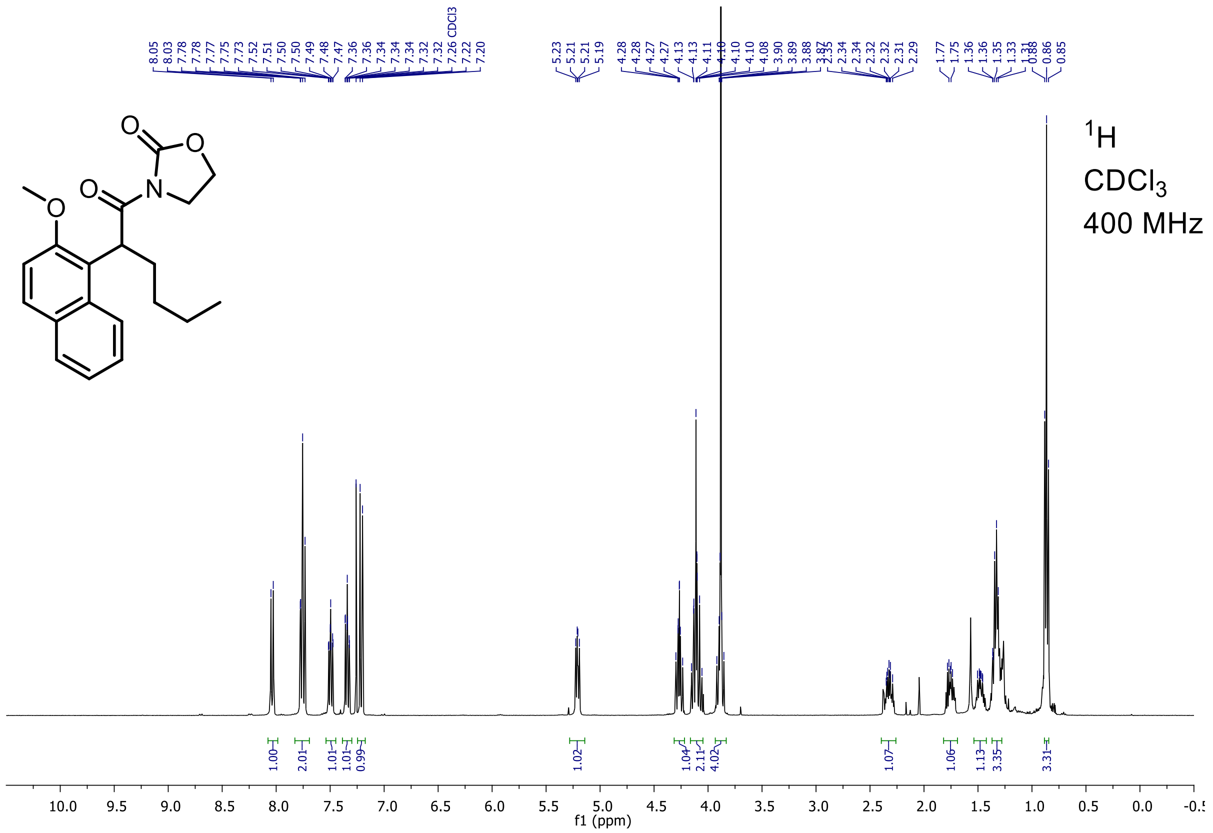

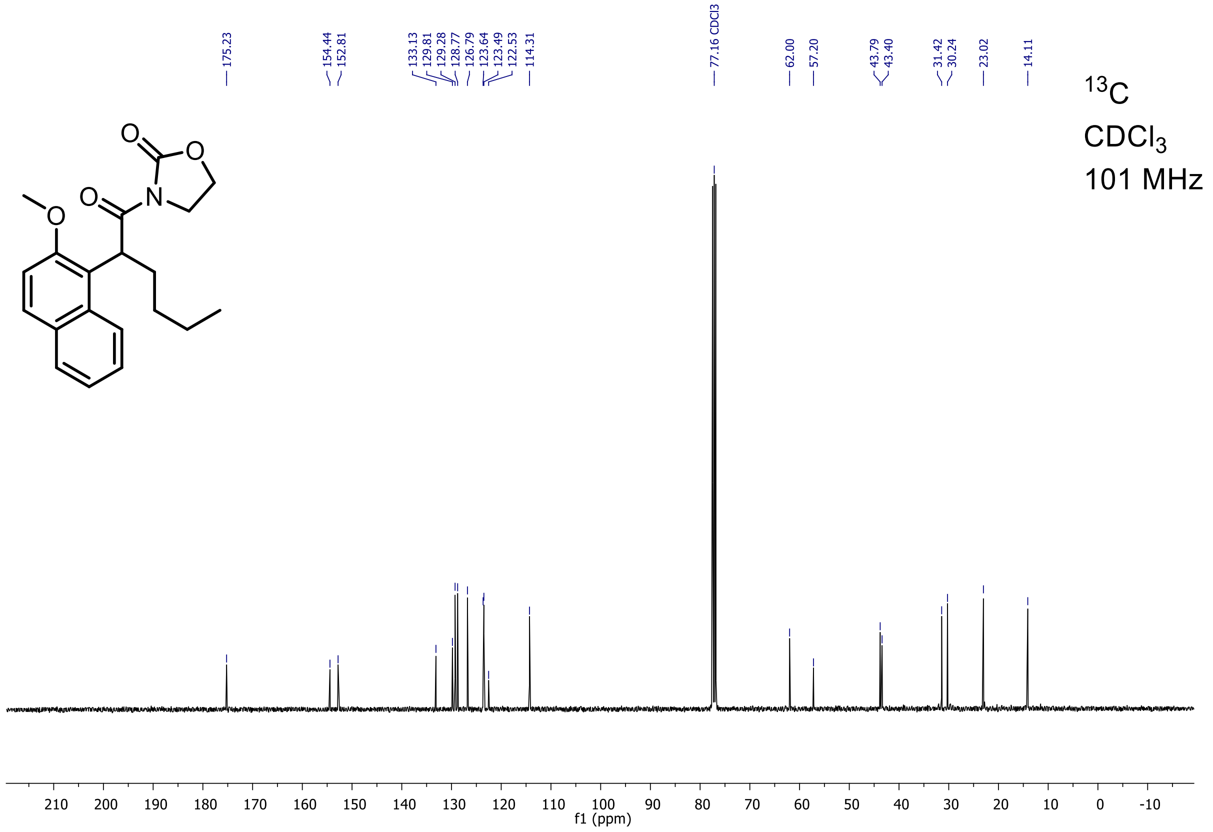

4kA – Methyl 3-methoxy-4-(1-oxo-1-(2-oxooxazolidin-3-yl)hexan-2-yl)-2-naphthoate

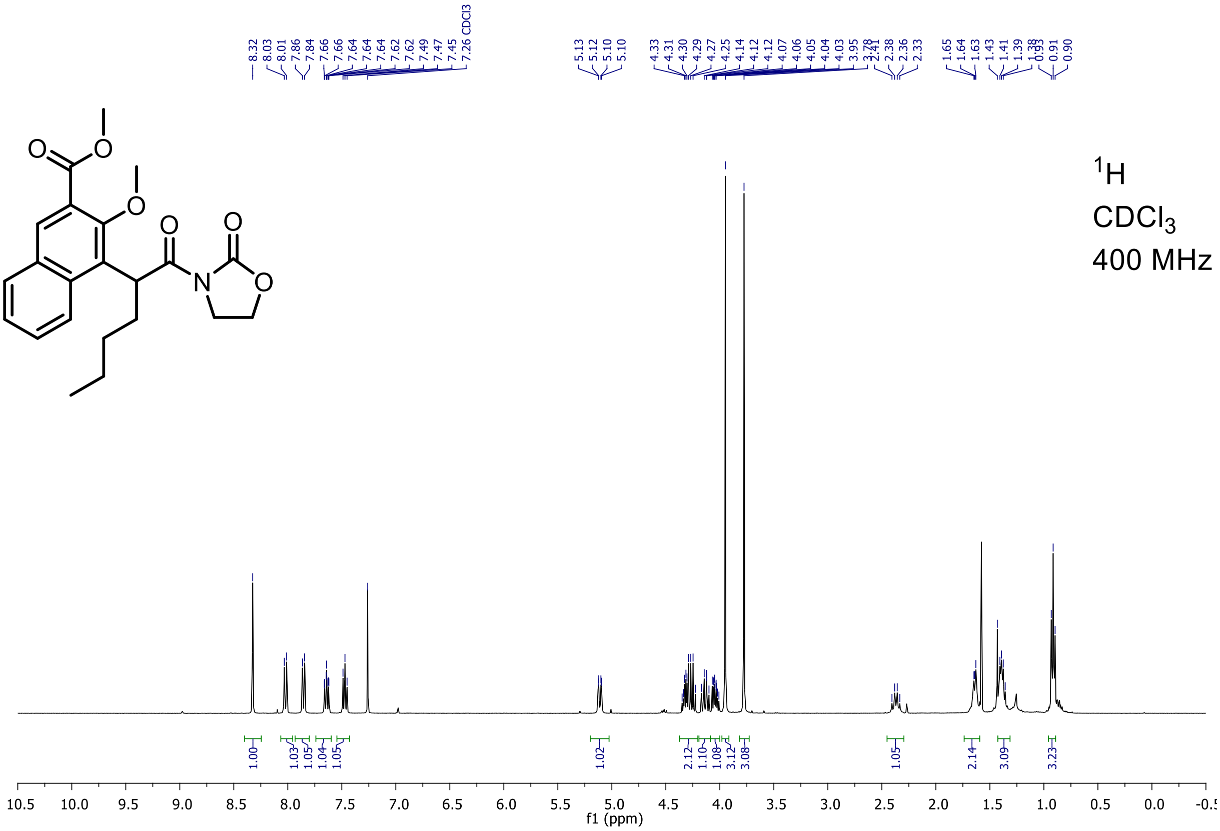

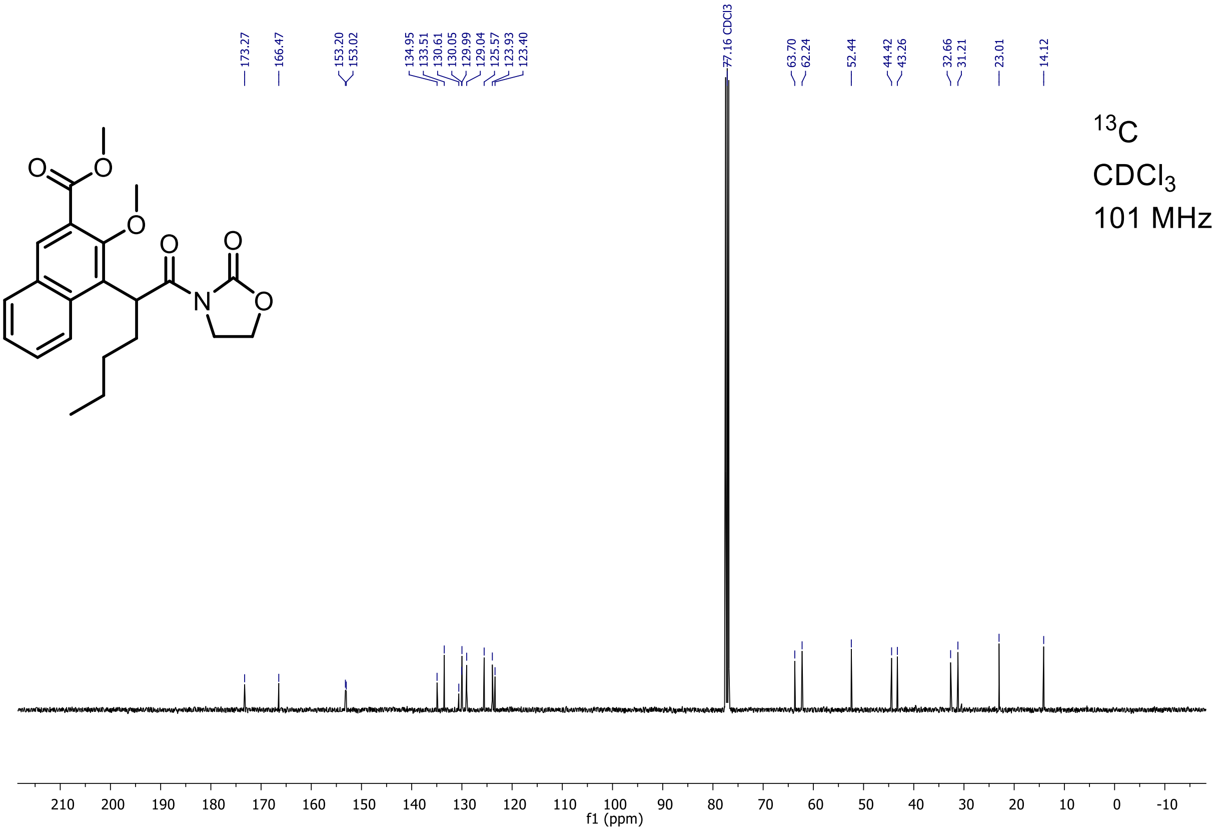

4lA – 3-(2-(2-methyl-1H-indol-3-yl)hexanoyl)oxazolidin-2-one

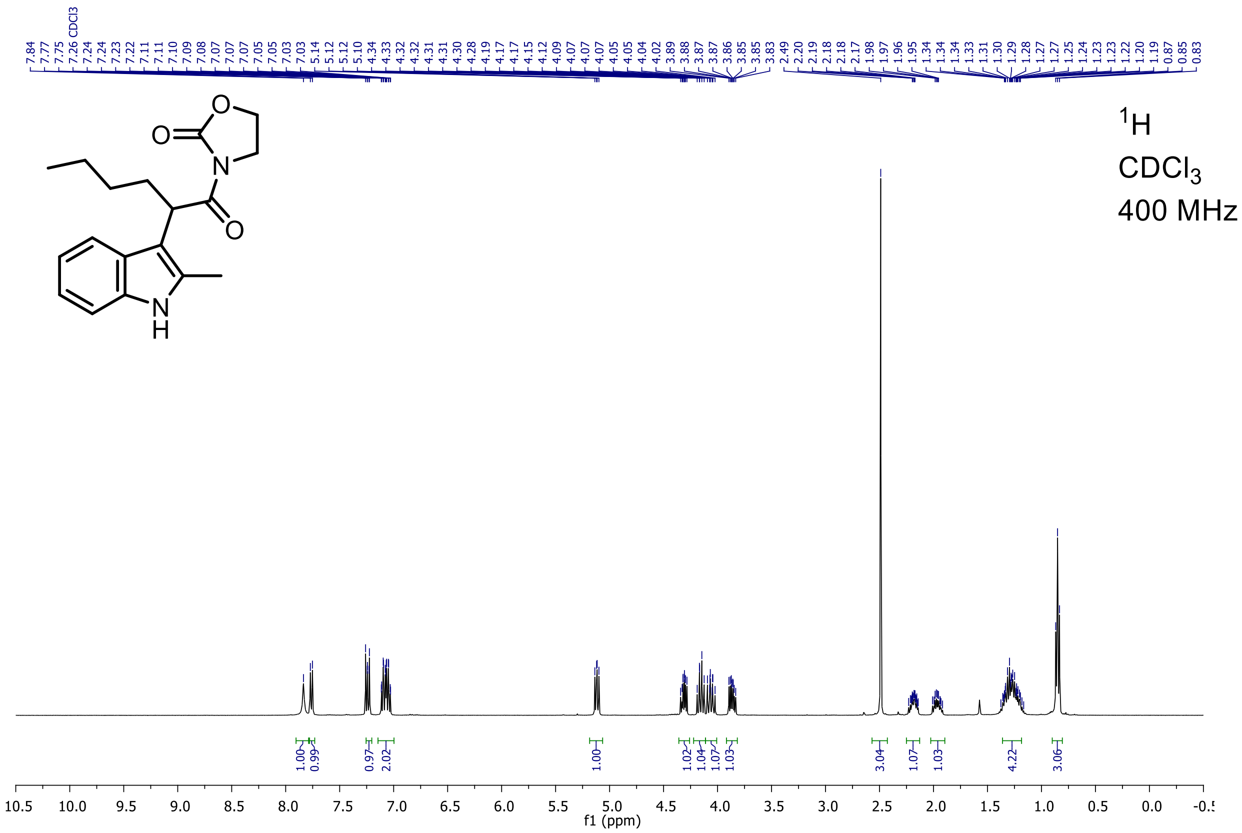

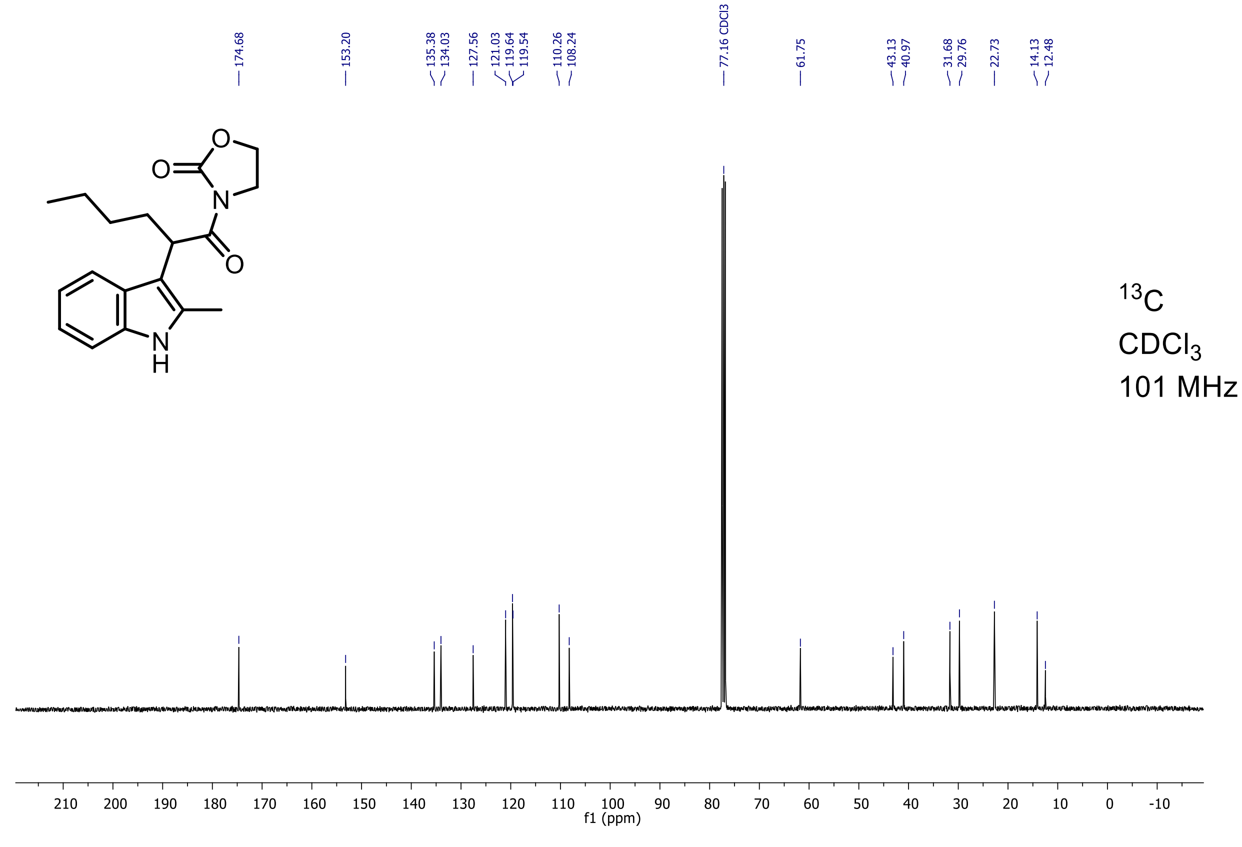

4aB – 3-(2-(2,6-Dimethoxyphenyl)acetyl)oxazolidin-2-one

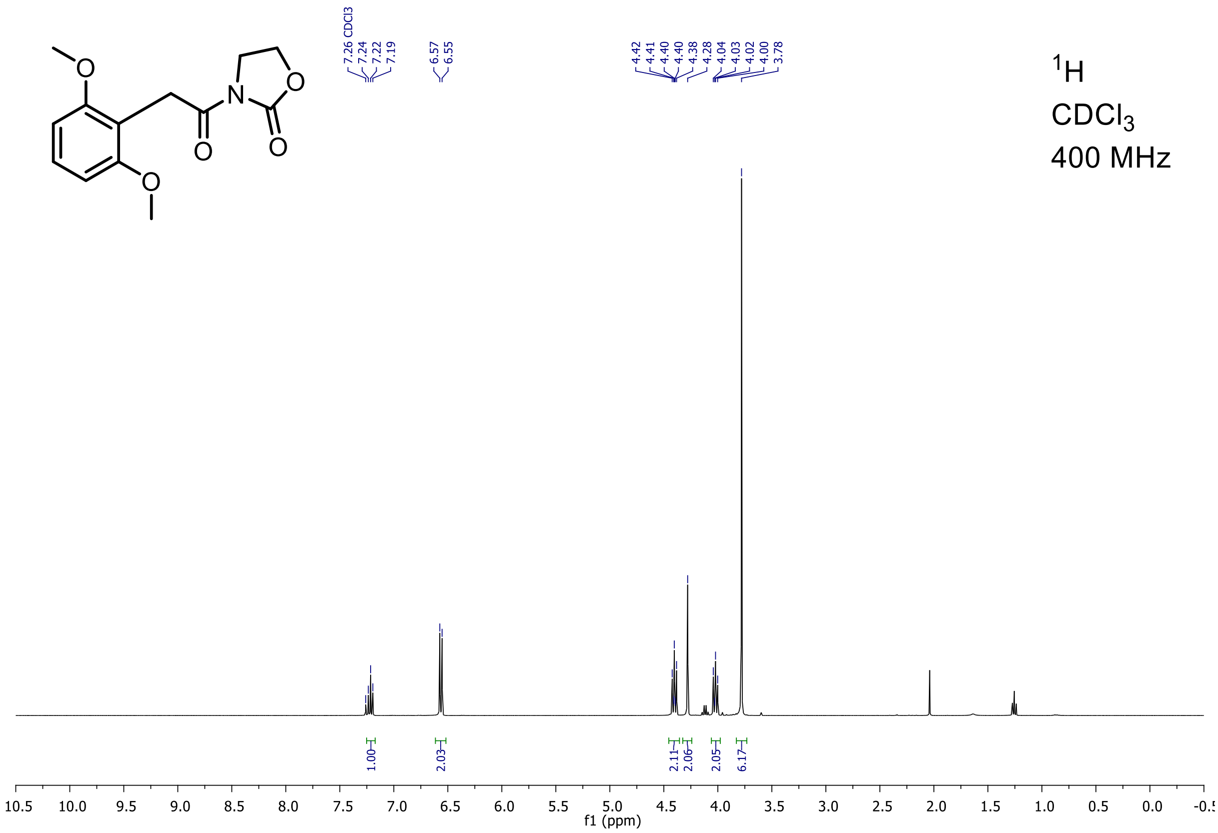

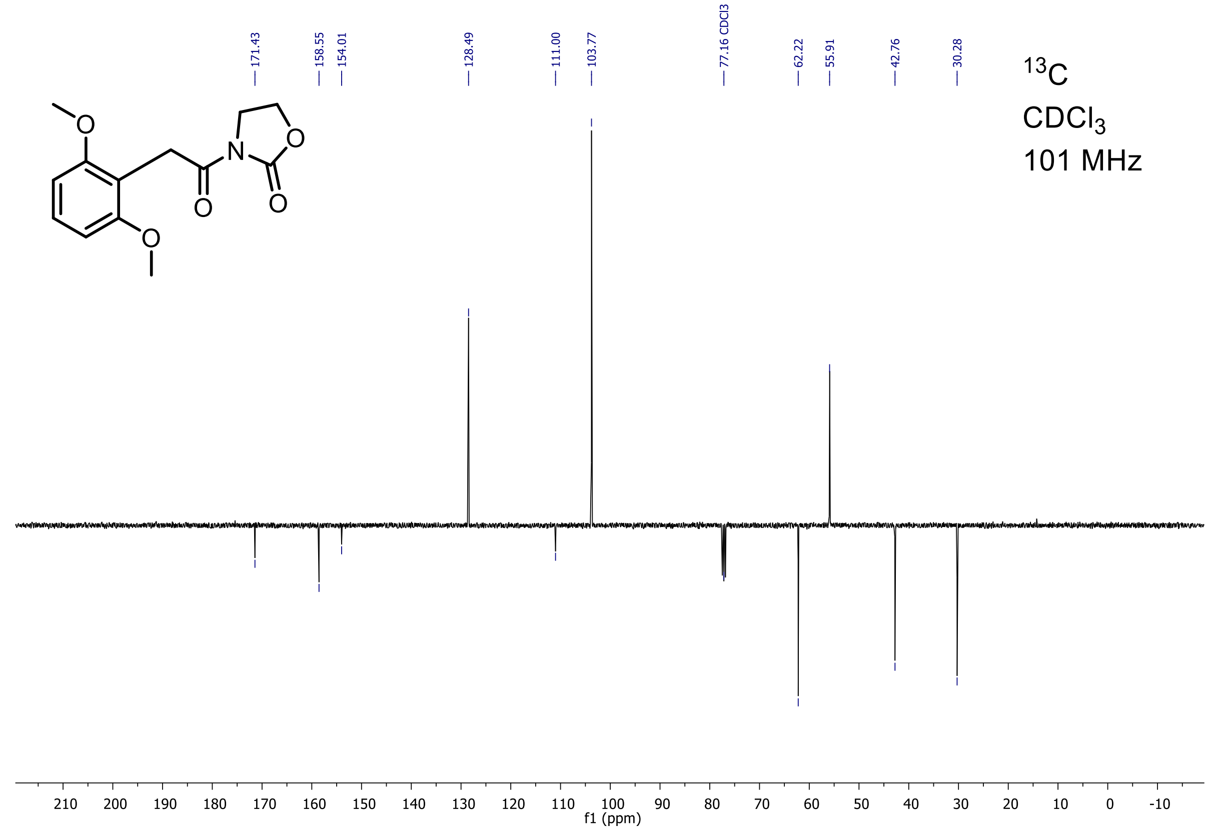

4aC – 3-(2-(2,6-Dimethoxyphenyl)propanoyl)oxazolidin-2-one

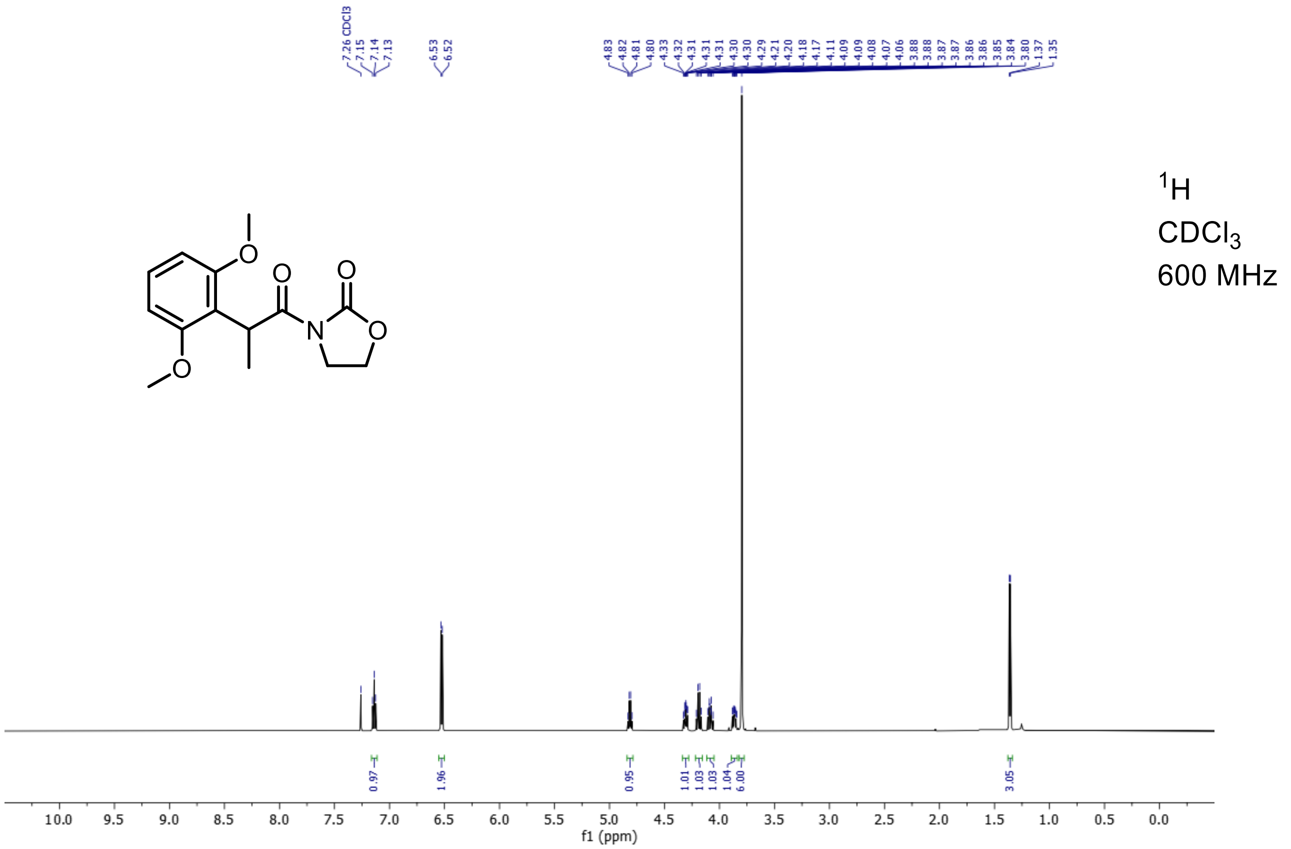

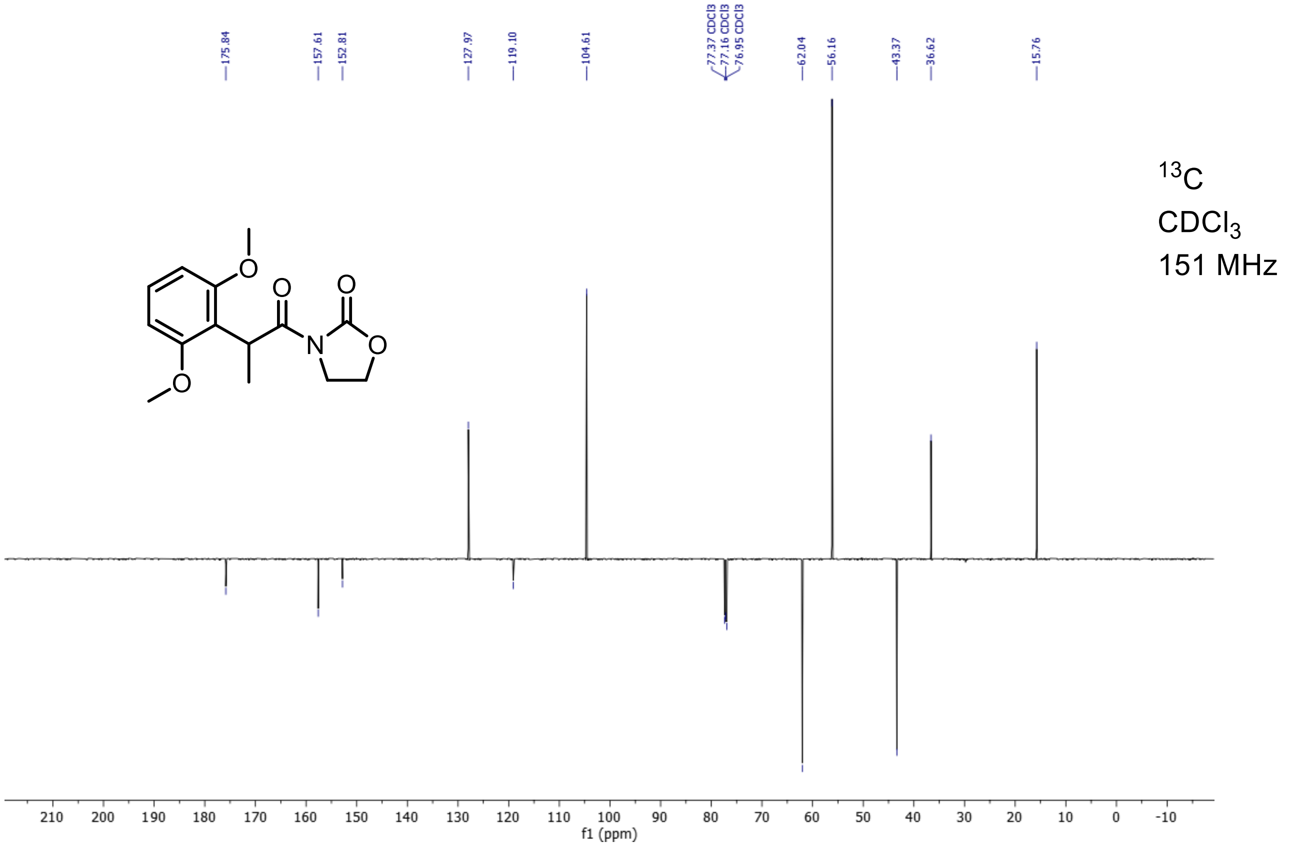

4aD – 3-(2-Cyclohexyl-2-(2,6-dimethoxyphenyl)acetyl)oxazolidin-2-one

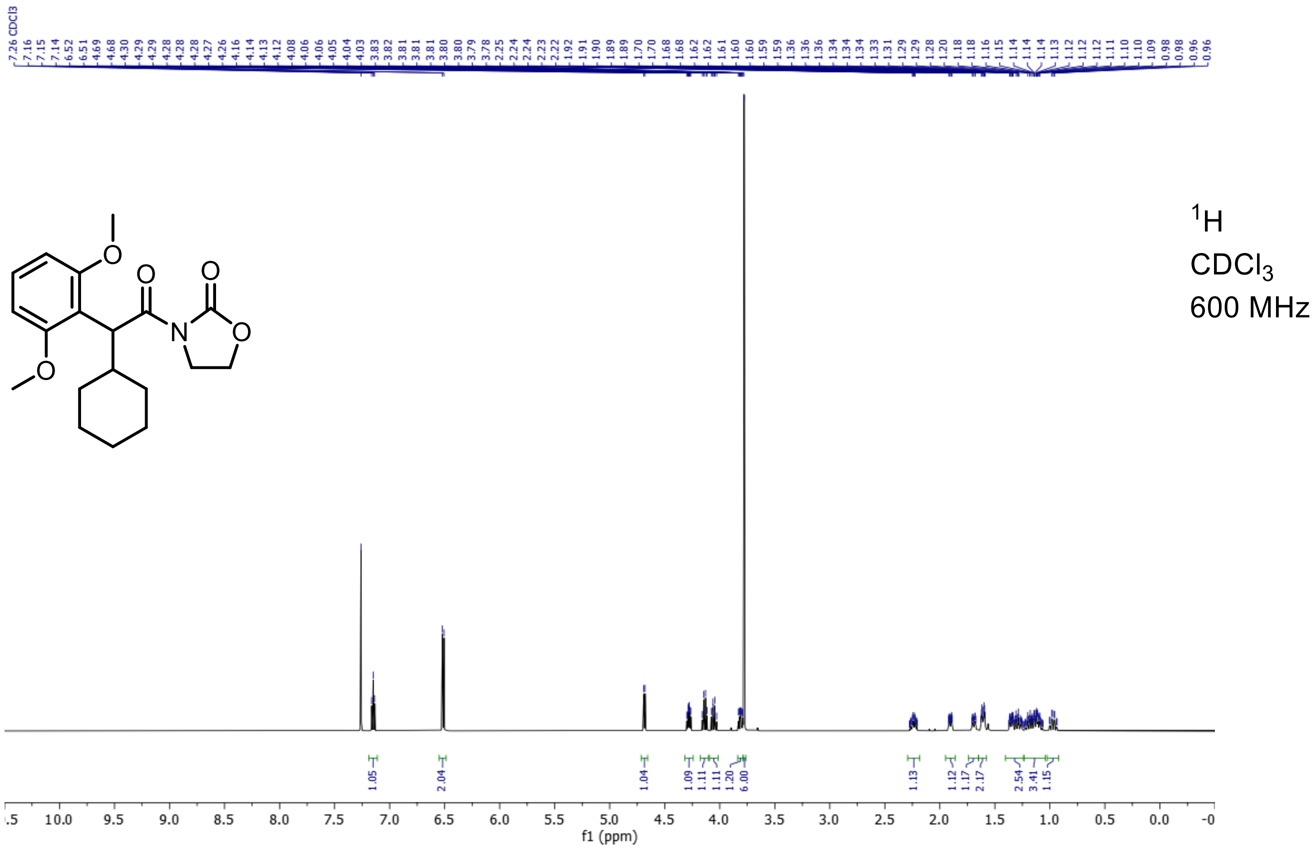

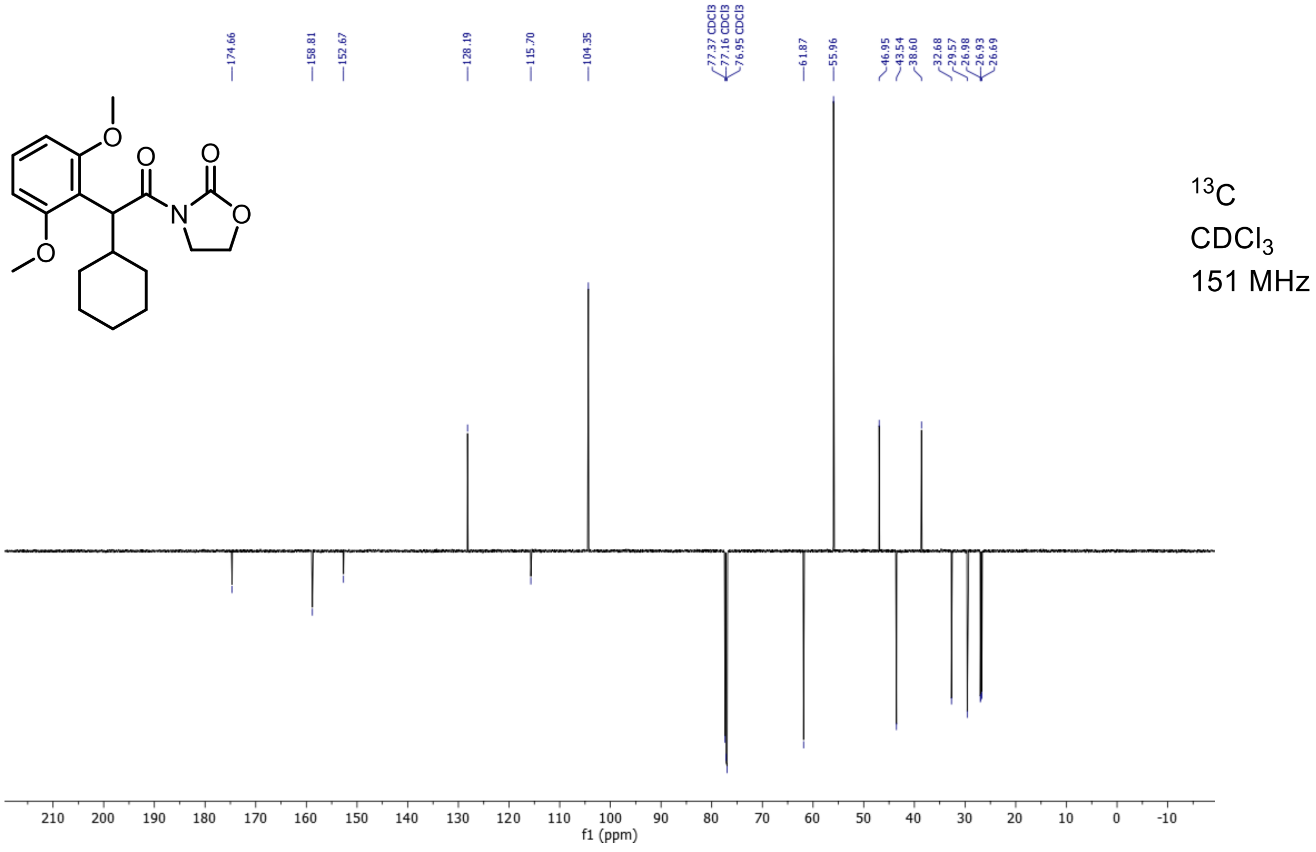

4aE – 3-(3-Cyclohexyl-2-(2,6-dimethoxyphenyl)propanoyl)oxazolidin-2-one

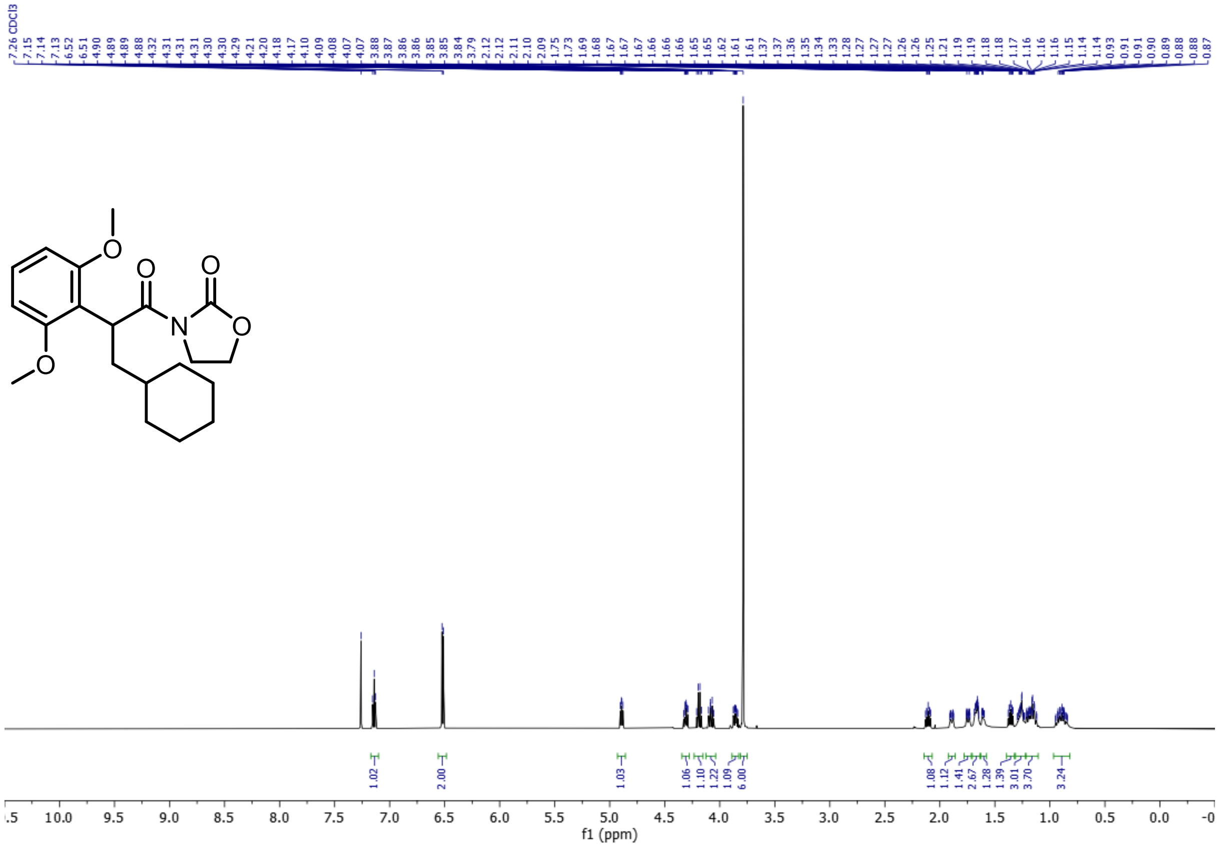

^{1}H
$CDCl_3$
600 MHz

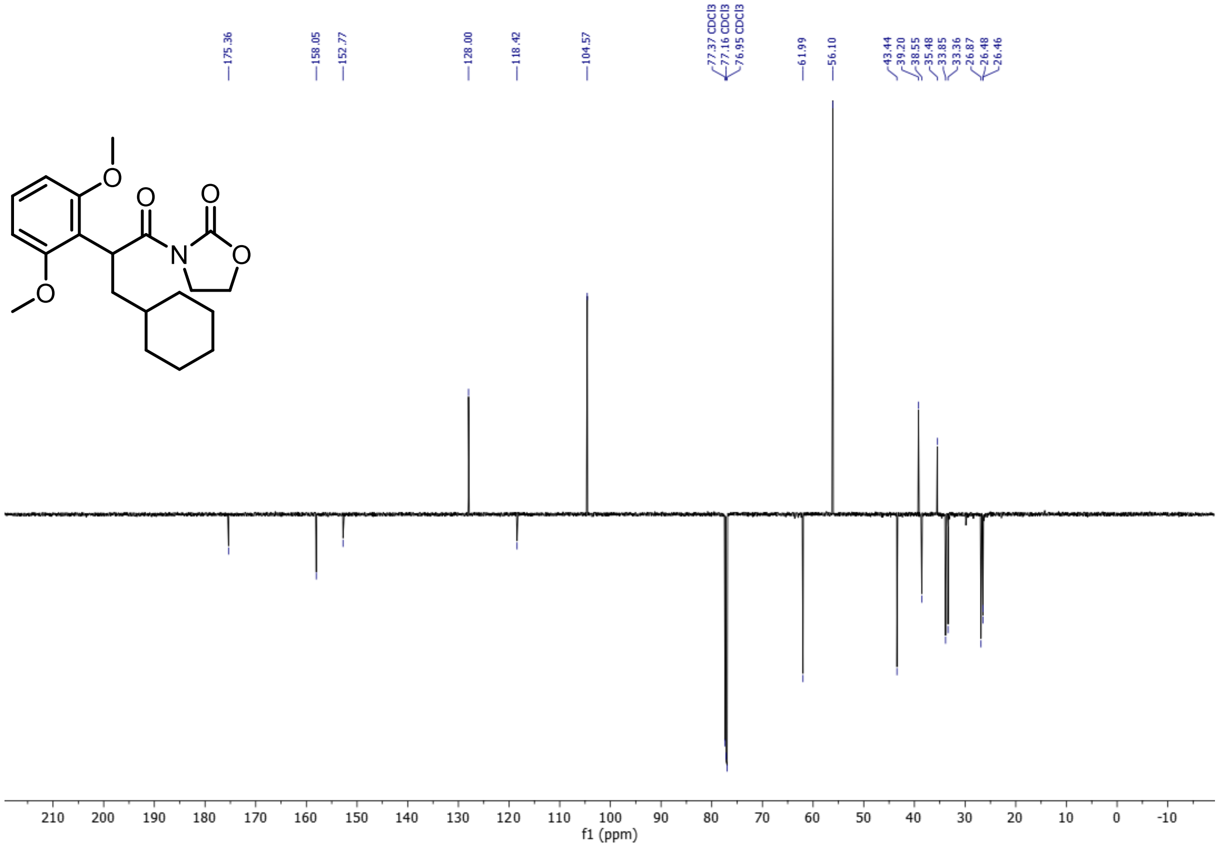

^{13}C
$CDCl_3$
151 MHz

4aF – 3-(2-(2,6-Dimethoxyphenyl)-5-phenylpentanoyl)oxazolidin-2-one

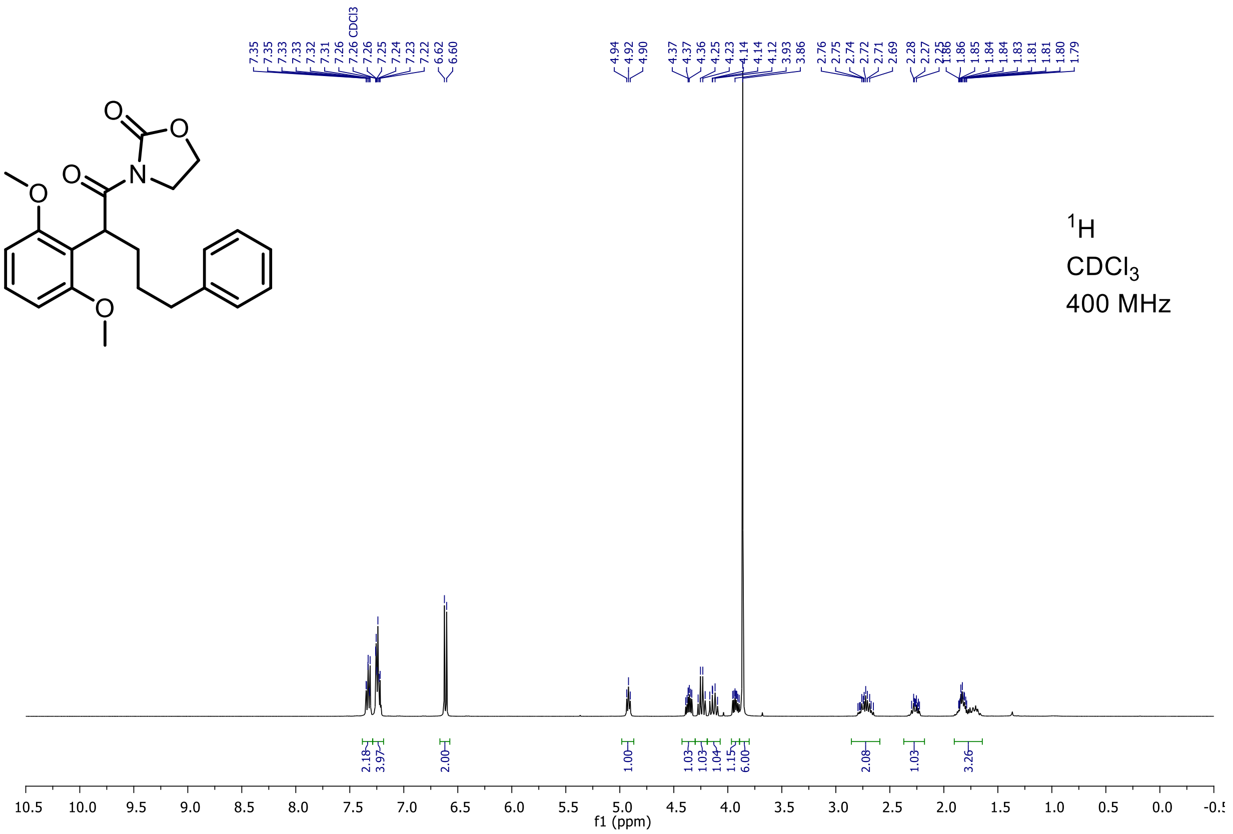

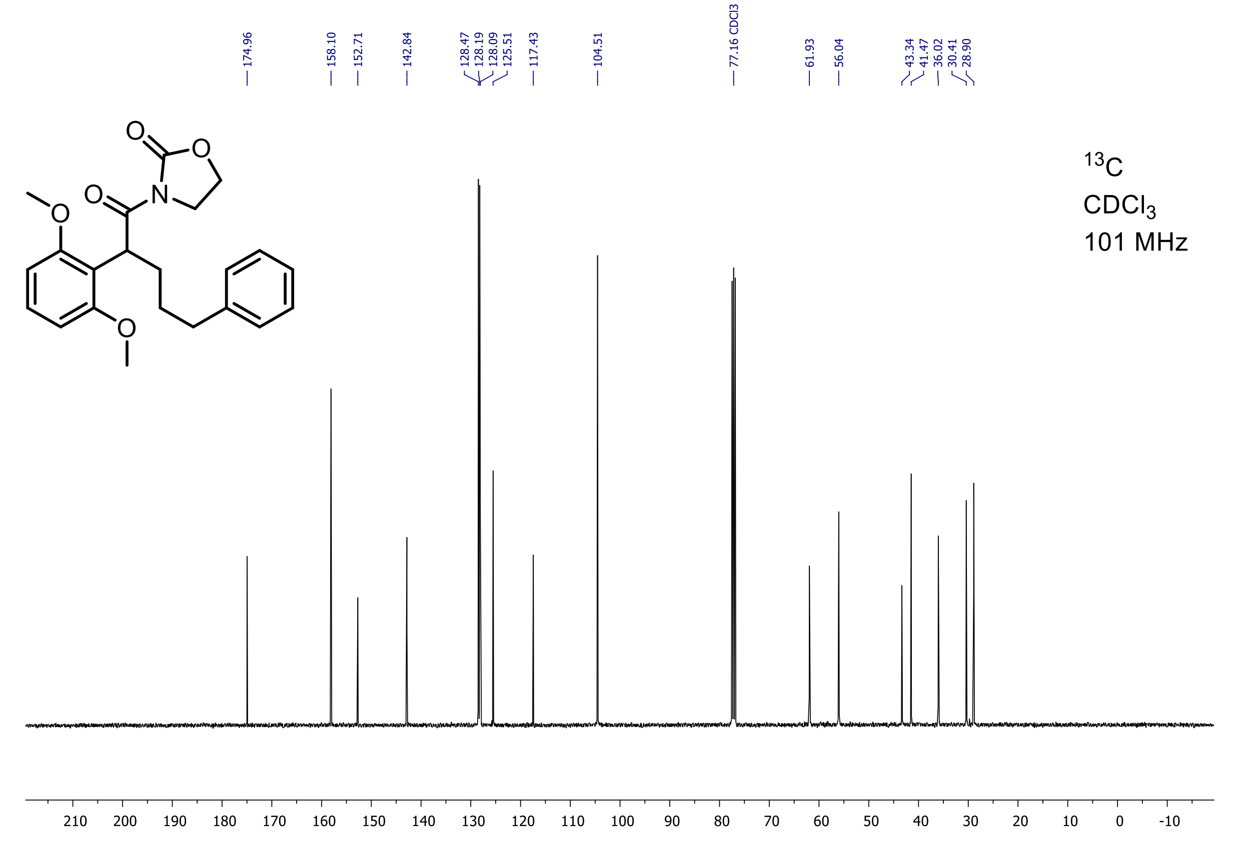

4aG – 2-(2,6-Dimethoxyphenyl)-1-(2-oxooxazolidin-3-yl)-11-phenylundecane-1,11-dione

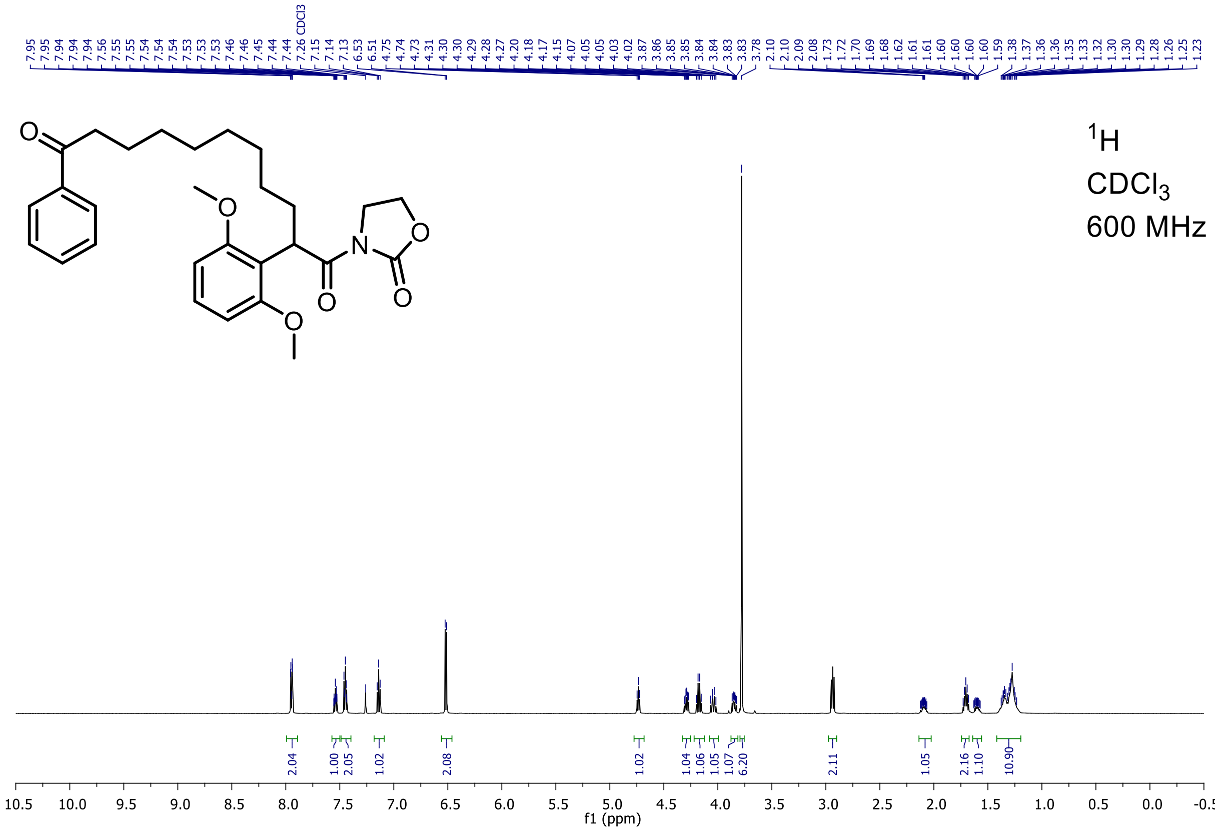

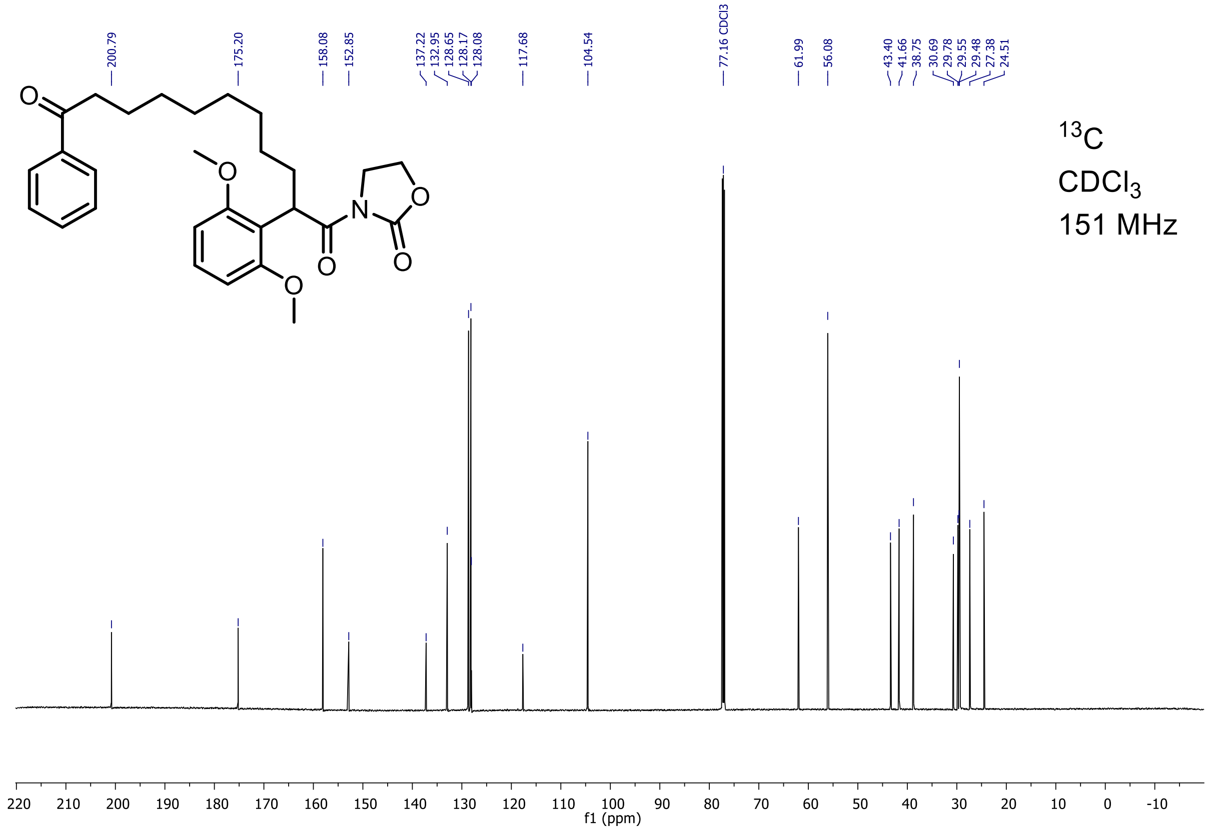

4aH – Methyl 10-(2,6-dimethoxyphenyl)-11-oxo-11-(2-oxooxazolidin-3-yl)undecanoate

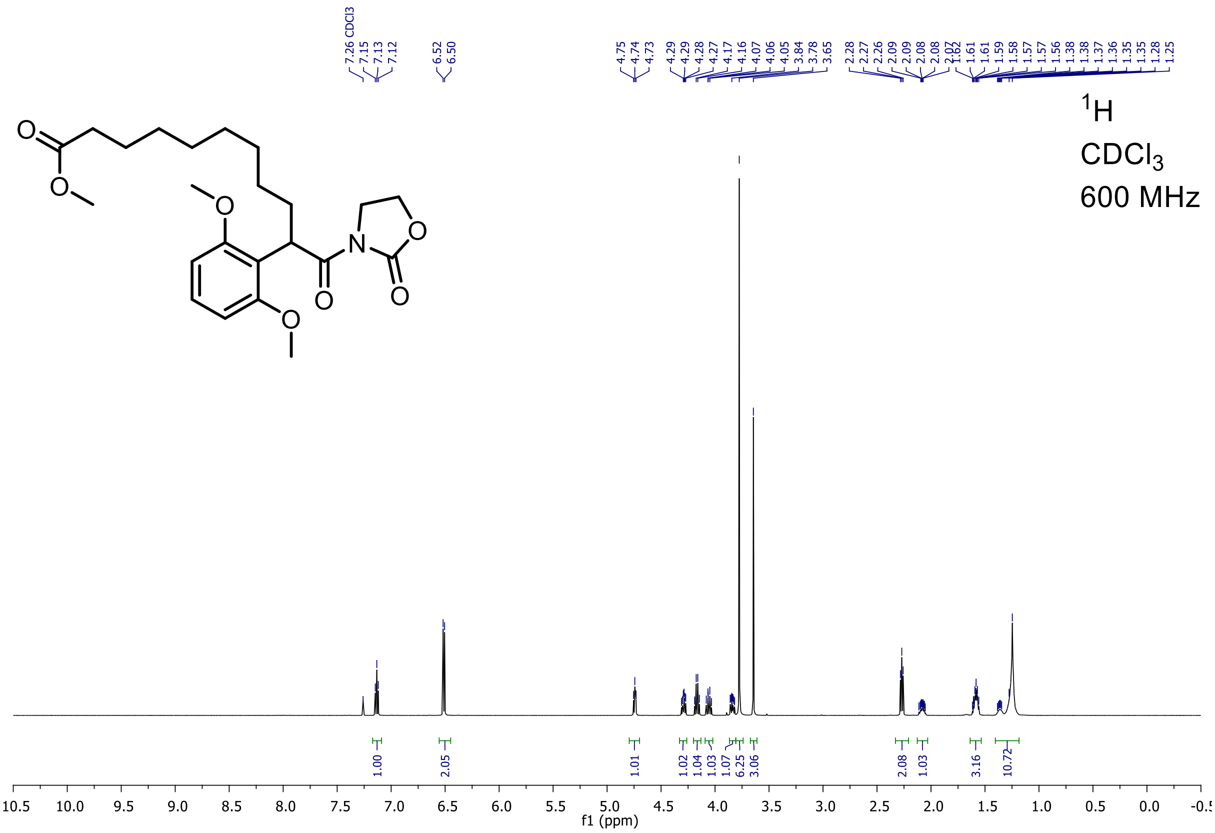

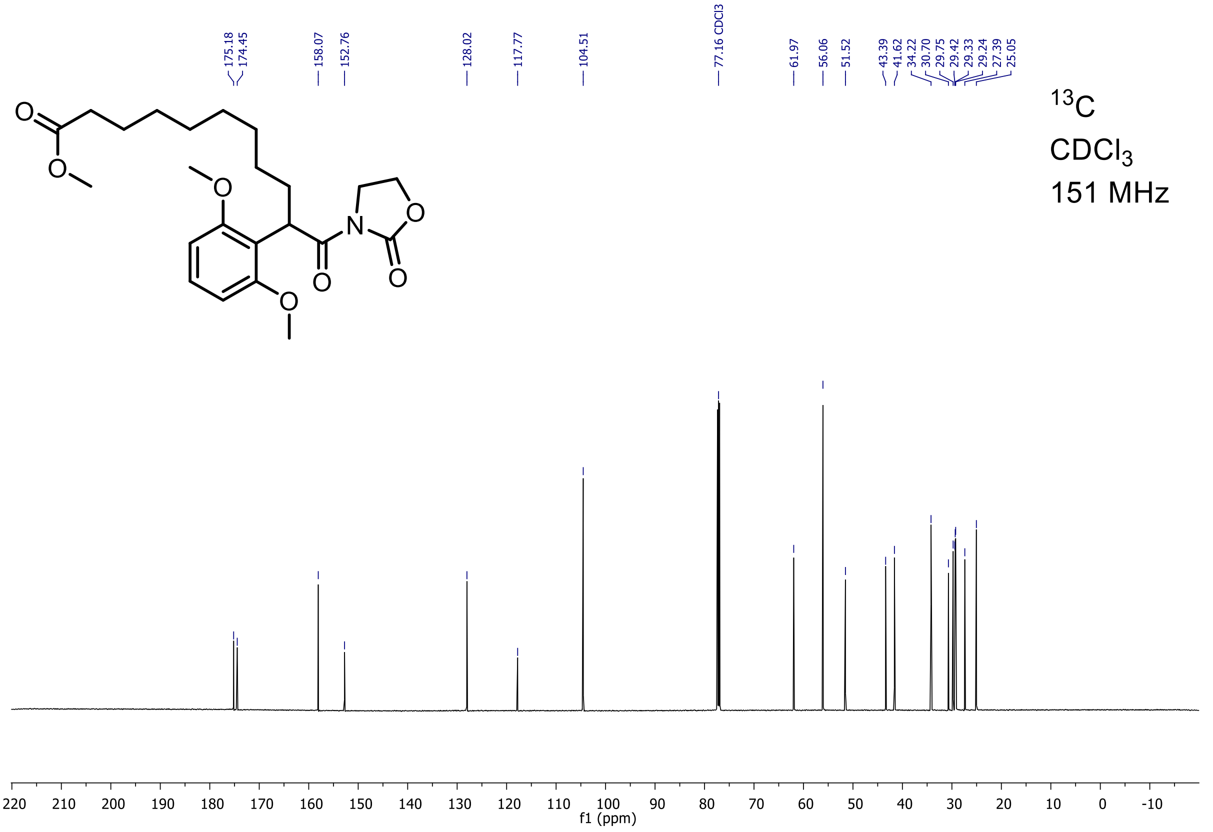

4al – 10-(2,6-Dimethoxyphenyl)-*N*-methoxy-*N*-methyl-11-oxo-11-(2-oxooxazolidin-3-yl)un…

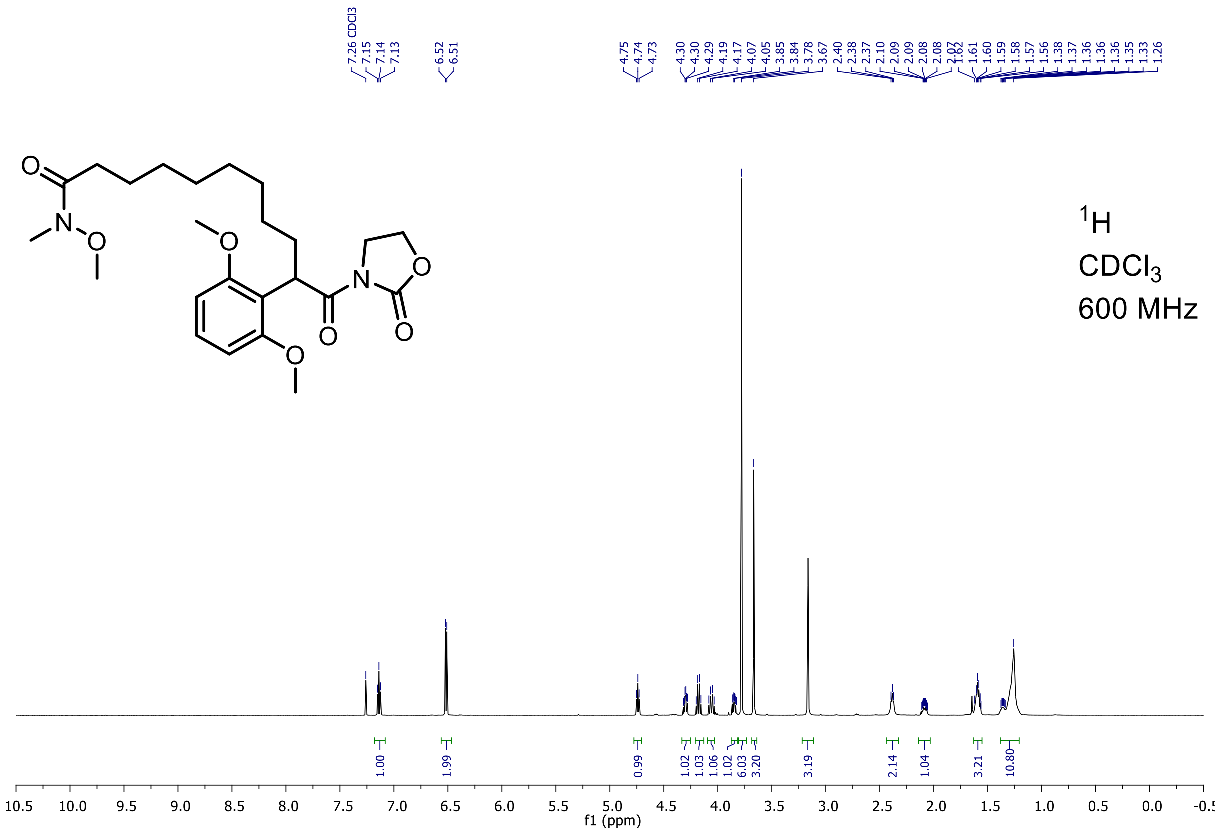

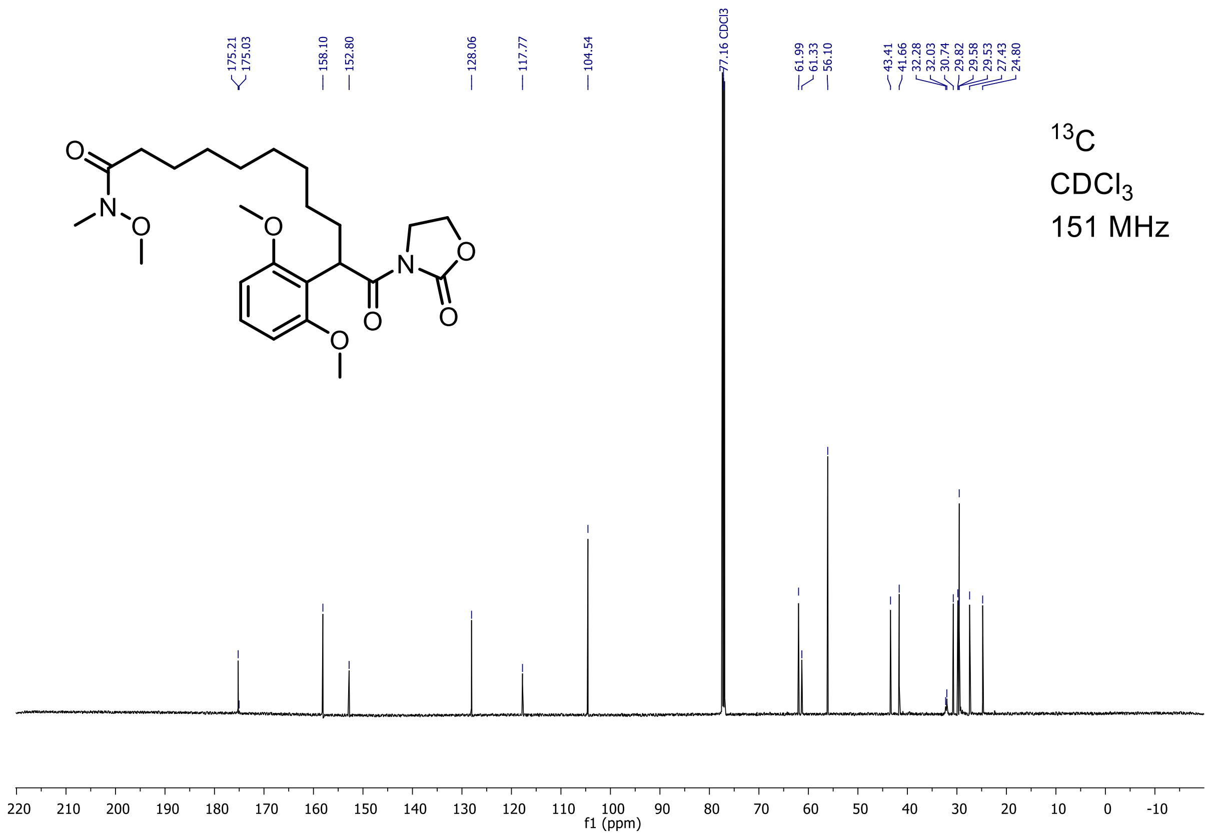

4aJ – Benzyl (*S*)-2-((*tert*-butoxycarbonyl)amino)-5-(2,6-dimethoxyphenyl)-6-oxo-6-(2-oxo…

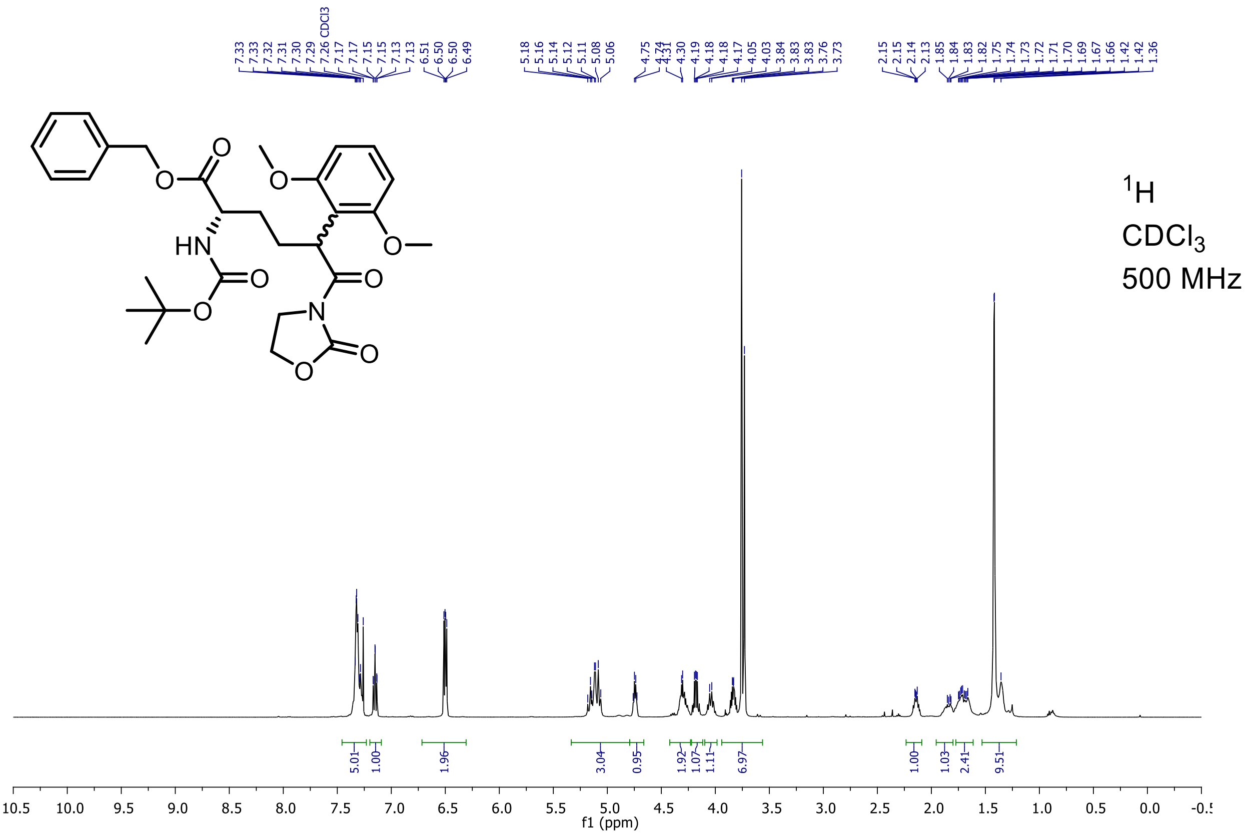

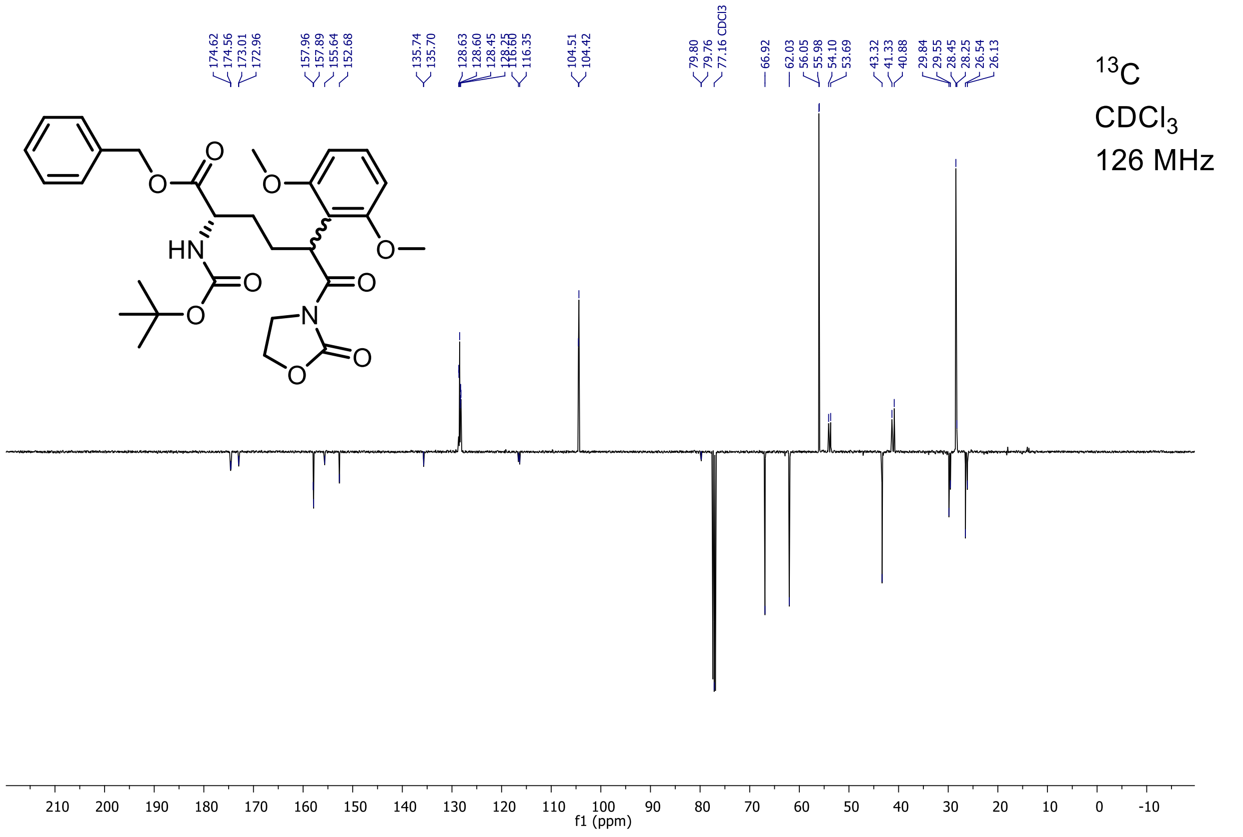

5aA – *S*-Methyl 2-(2,6-dimethoxyphenyl)hexanethioate

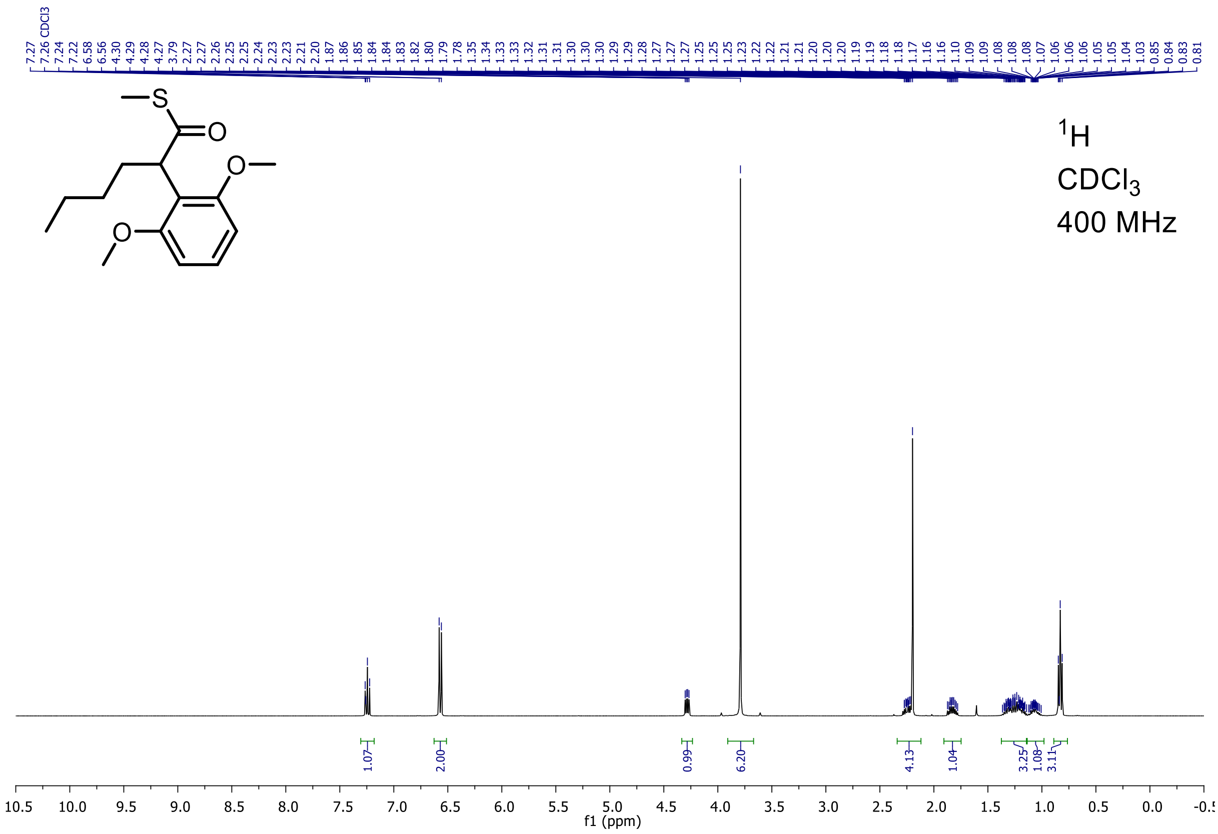

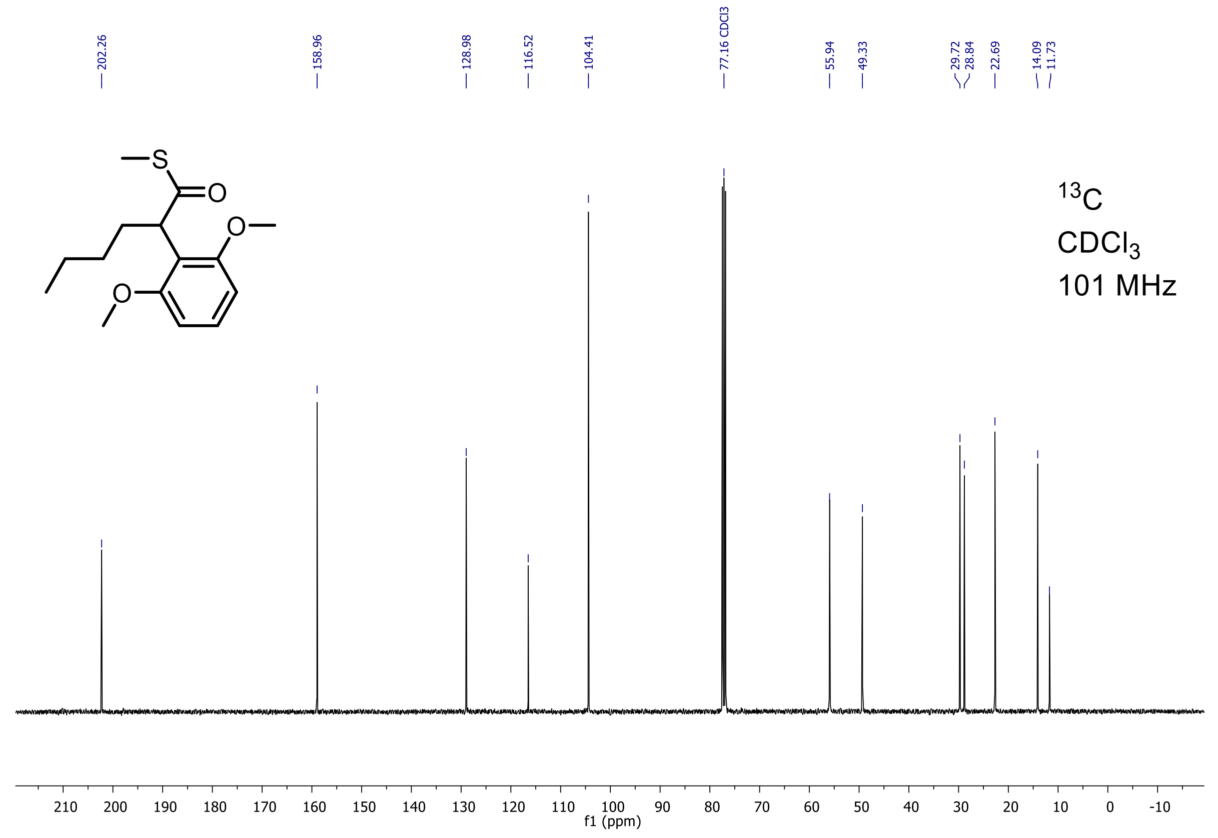

5aB – *S*-Methyl 2-cyclopentyl-2-(2,6-dimethoxyphenyl)ethanethioate

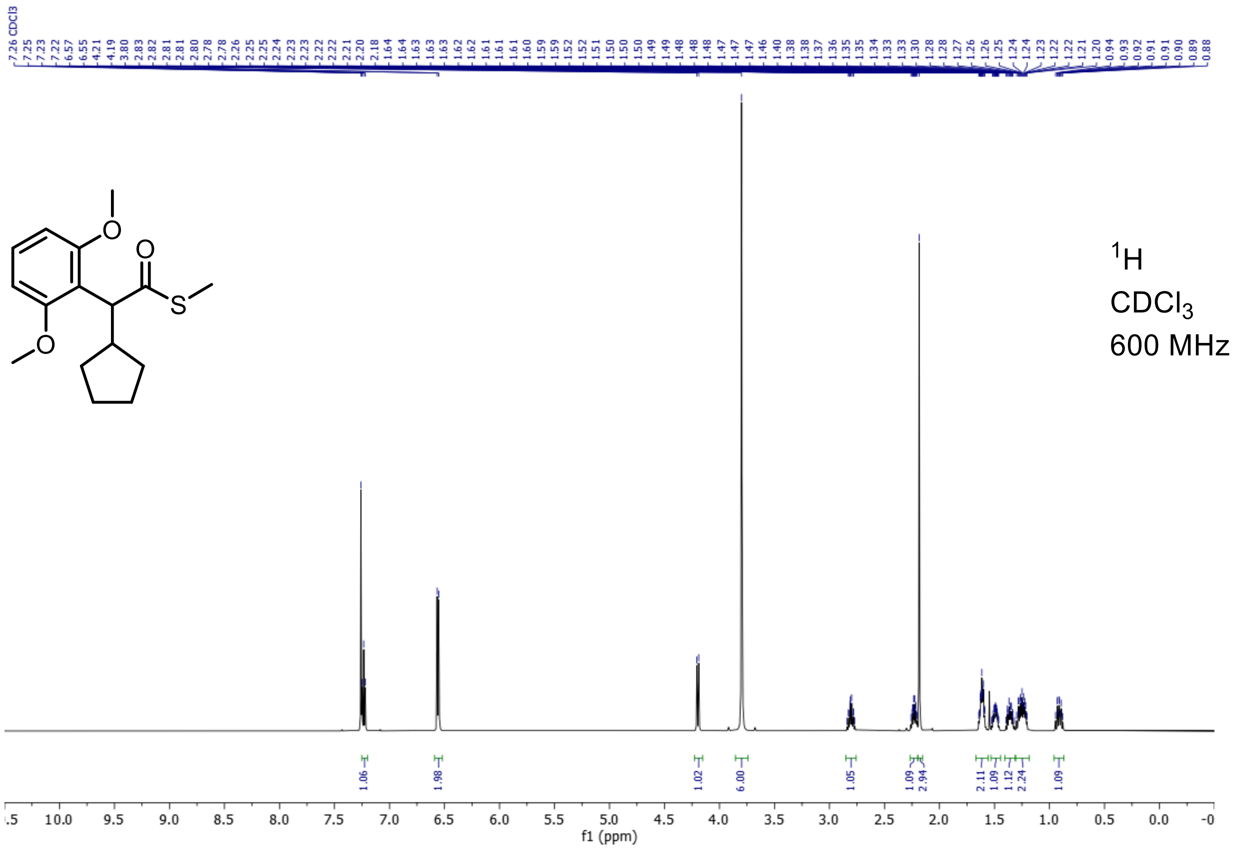

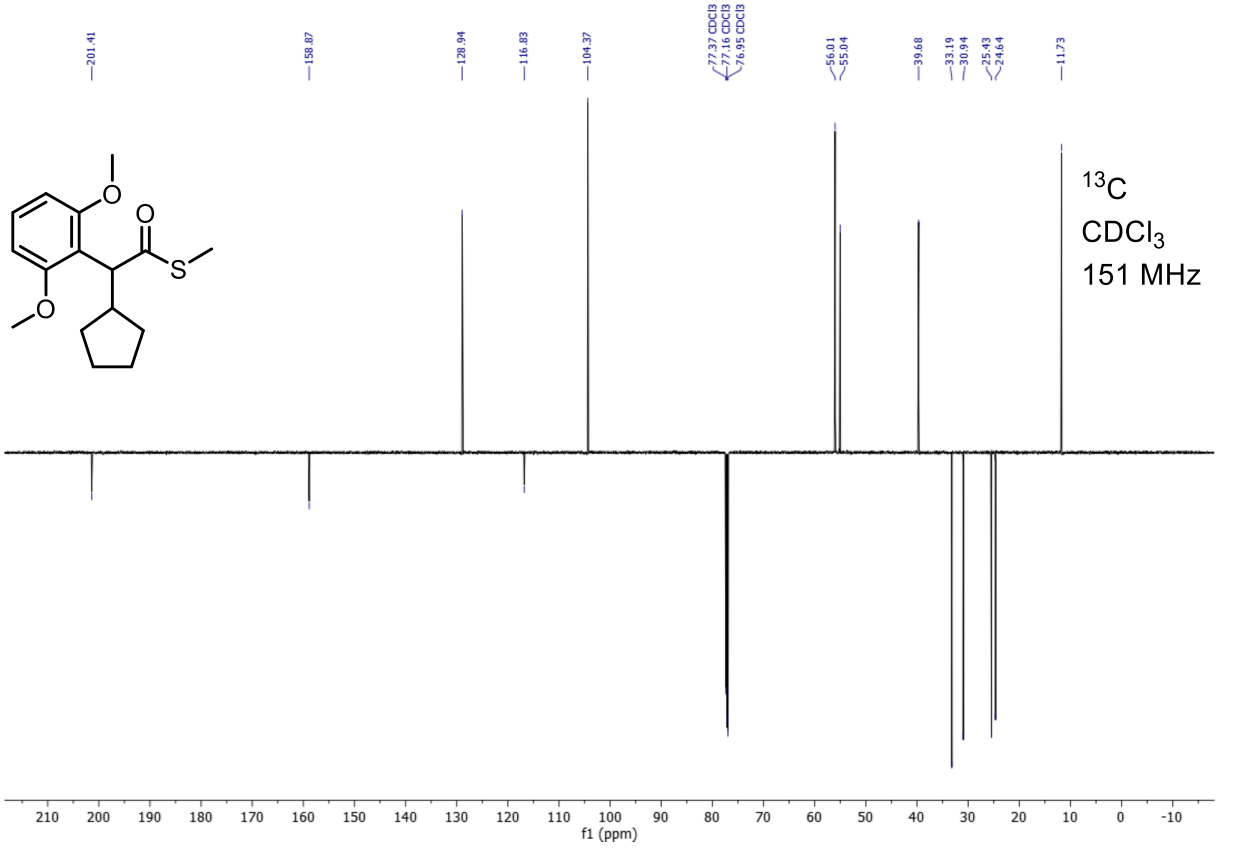

5aC – *S*-Phenyl 2-(2,6-dimethoxyphenyl)-3,3-dimethylbutanethioate

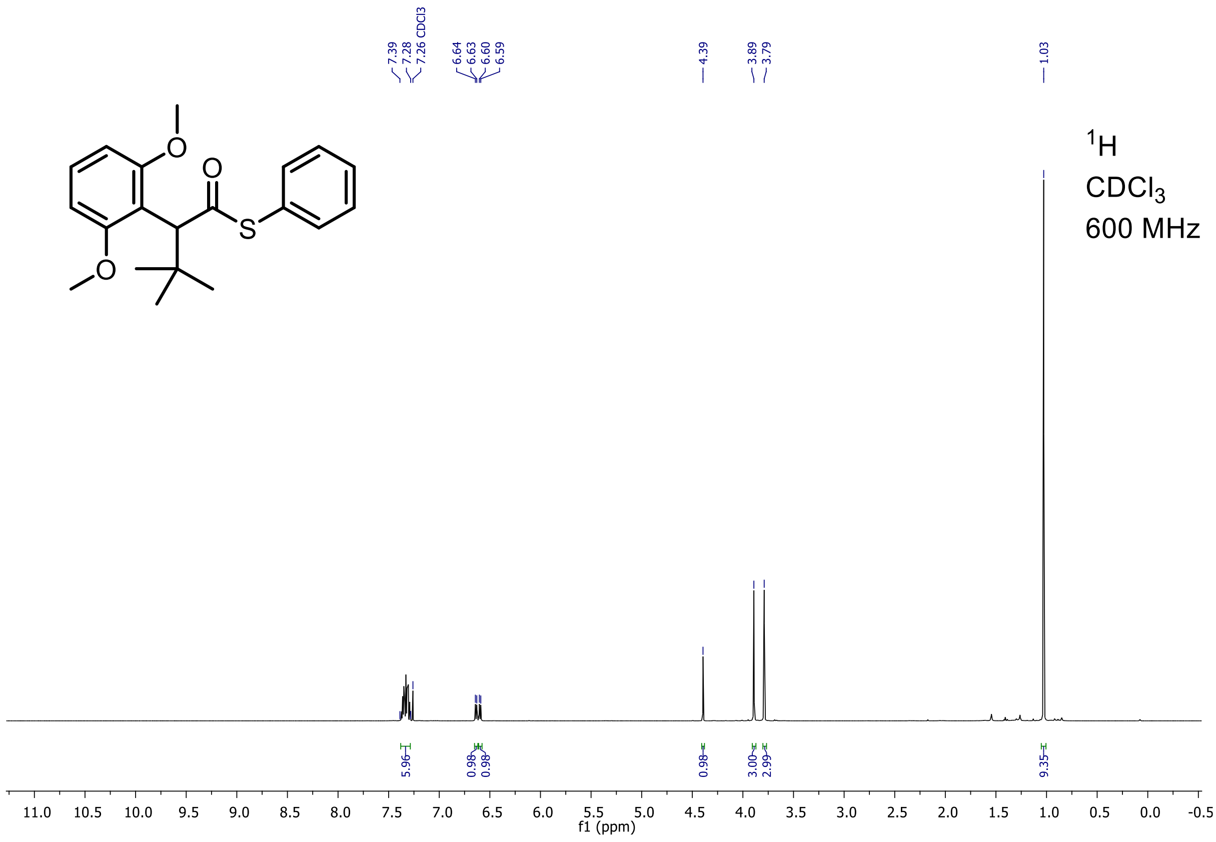

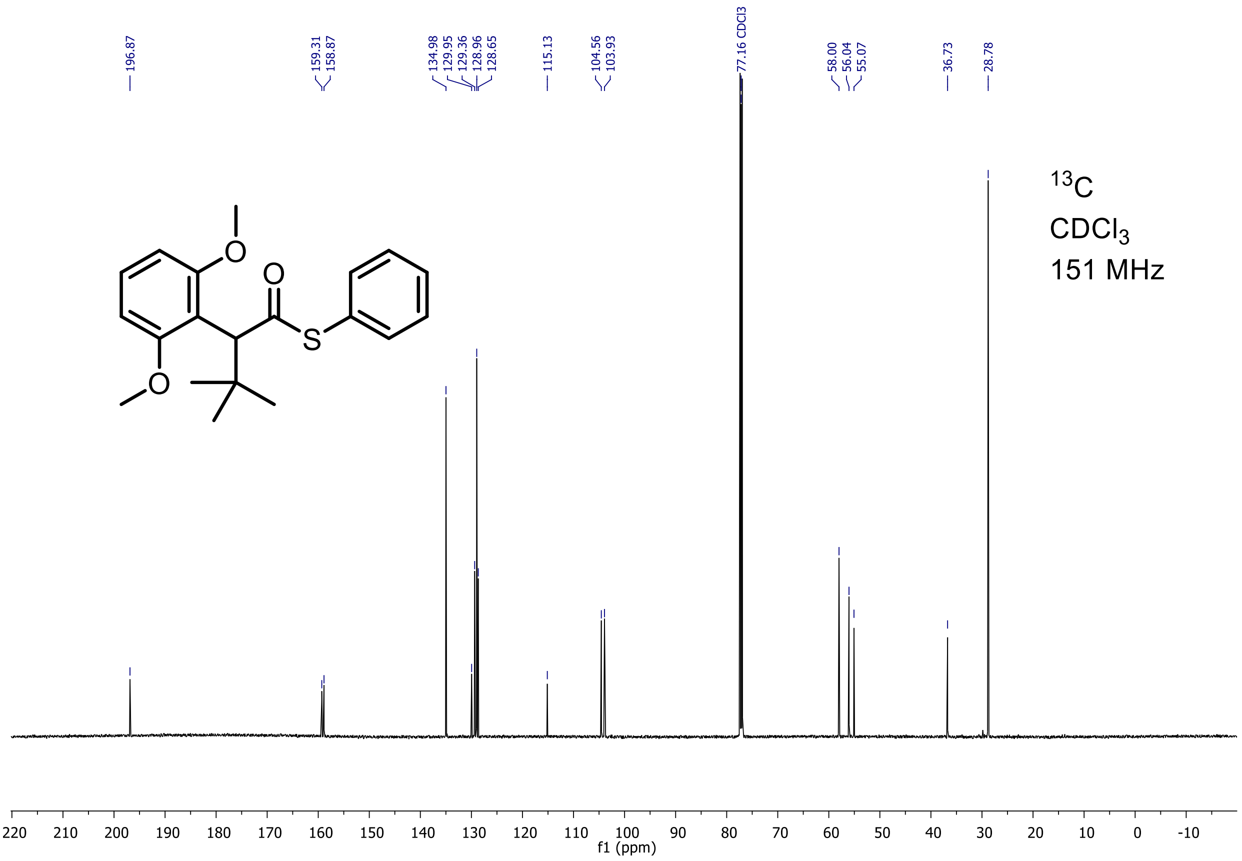

5hA – *S*-Methyl 2-(2,6-diisopropyl-4-methoxyphenyl)hexanethioate

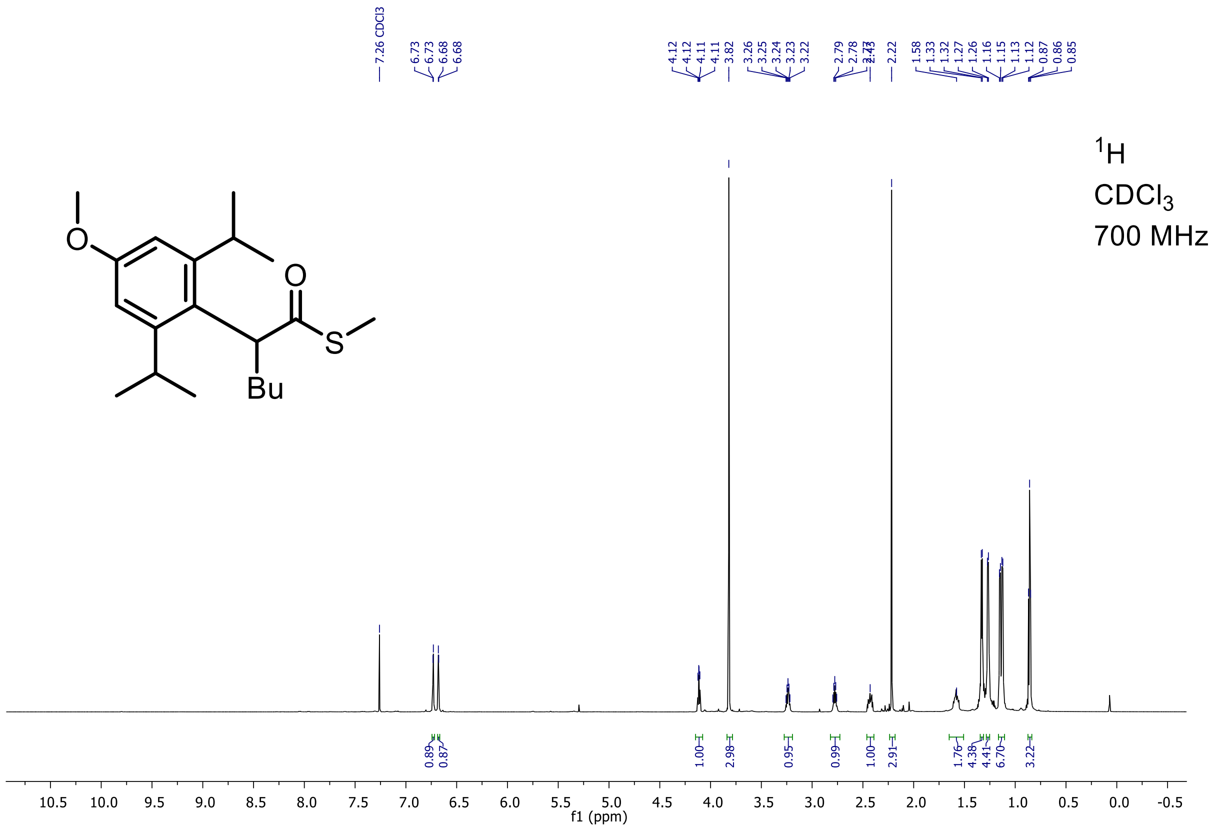

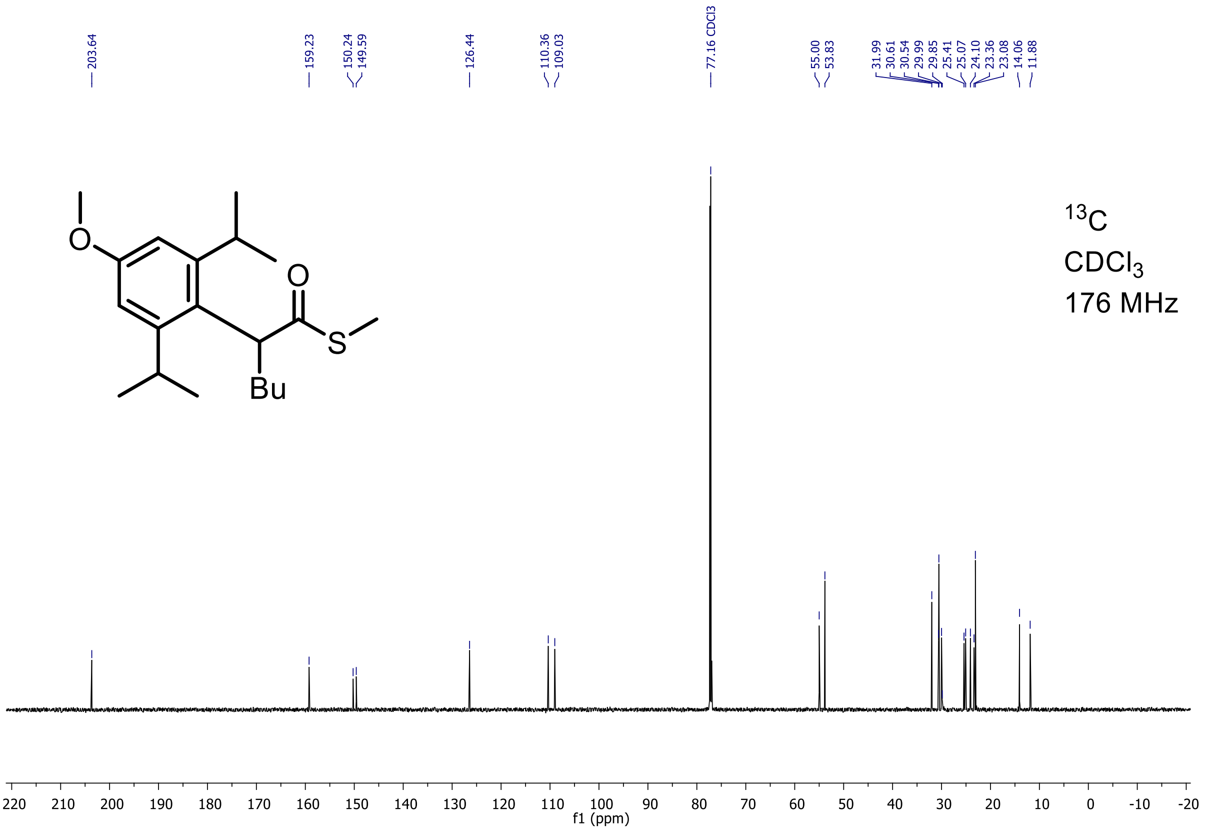

5hD – *S*-Methyl 5-chloro-2-(2,6-diisopropyl-4-methoxyphenyl)pentanethioate

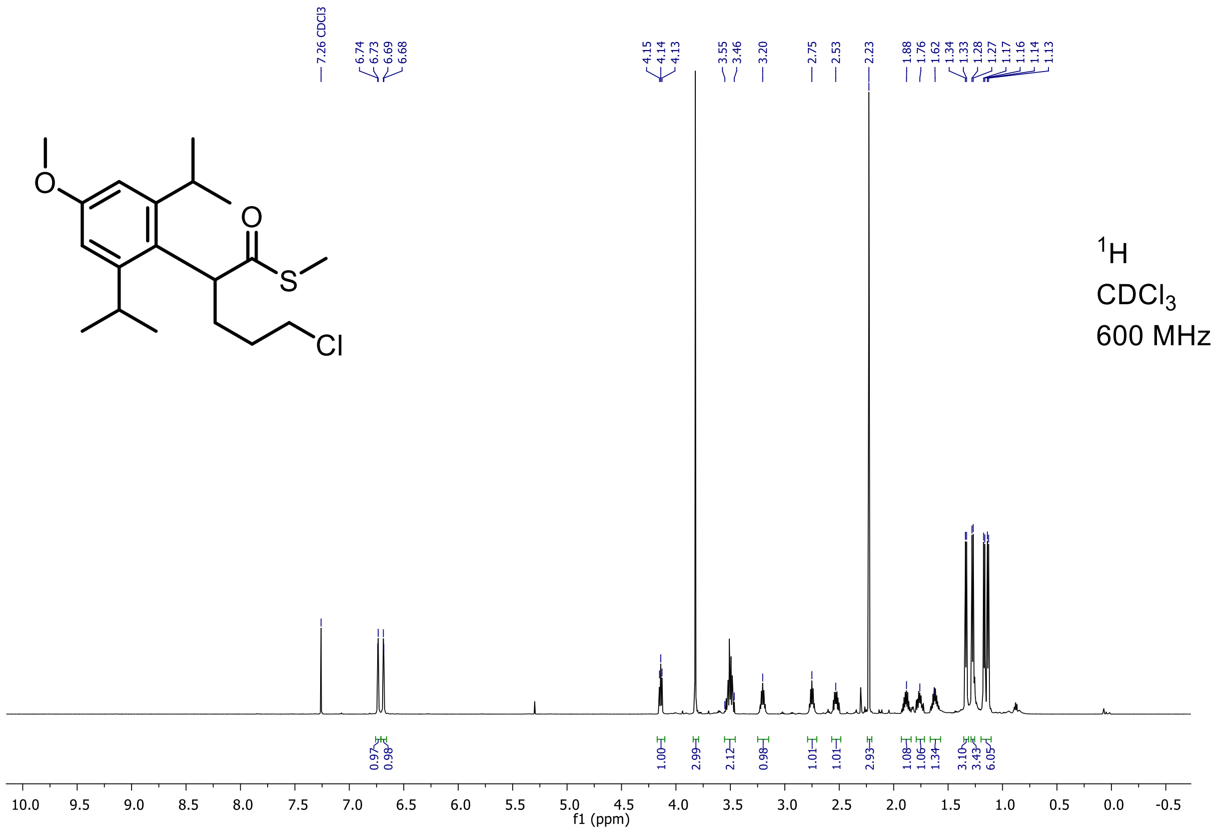

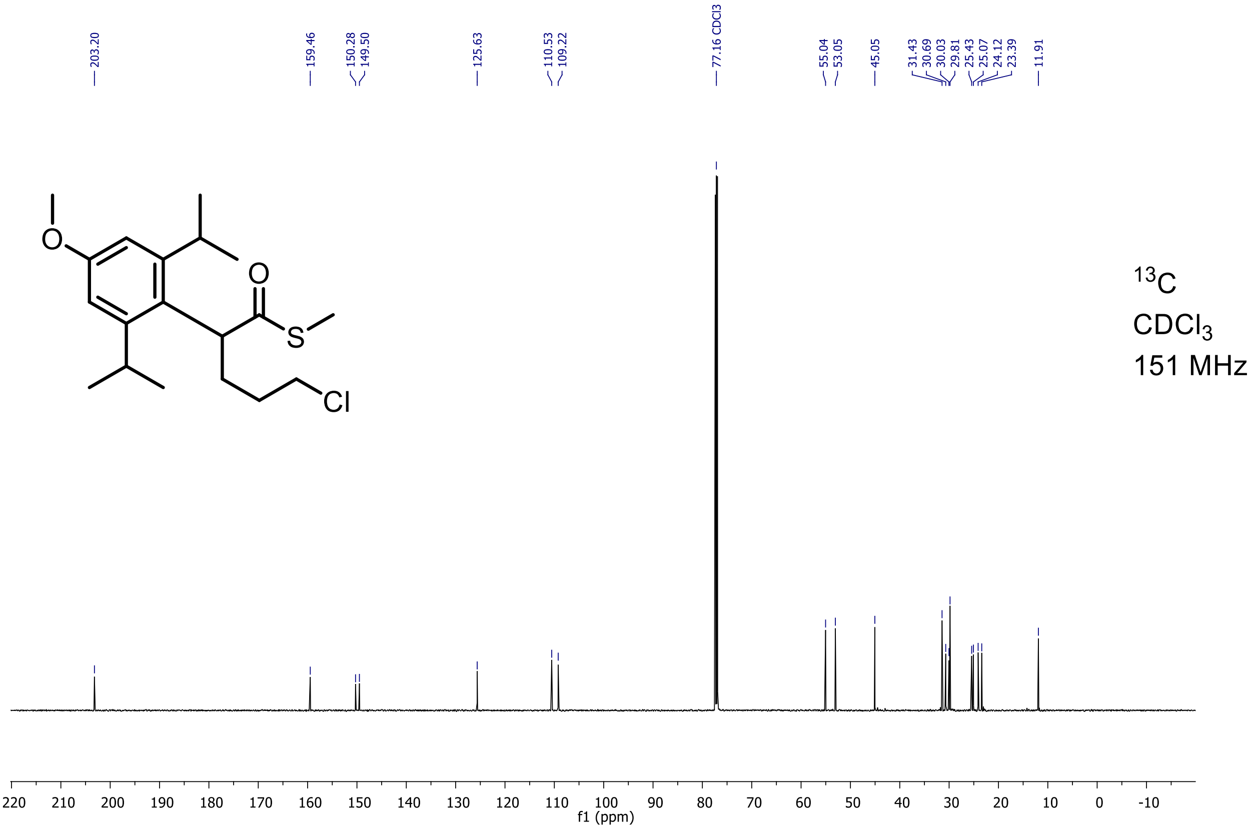

5hE – 4-(2,6-Diisopropyl-4-methoxyphenyl)-5-(methylthio)-5-oxopentyl acetate

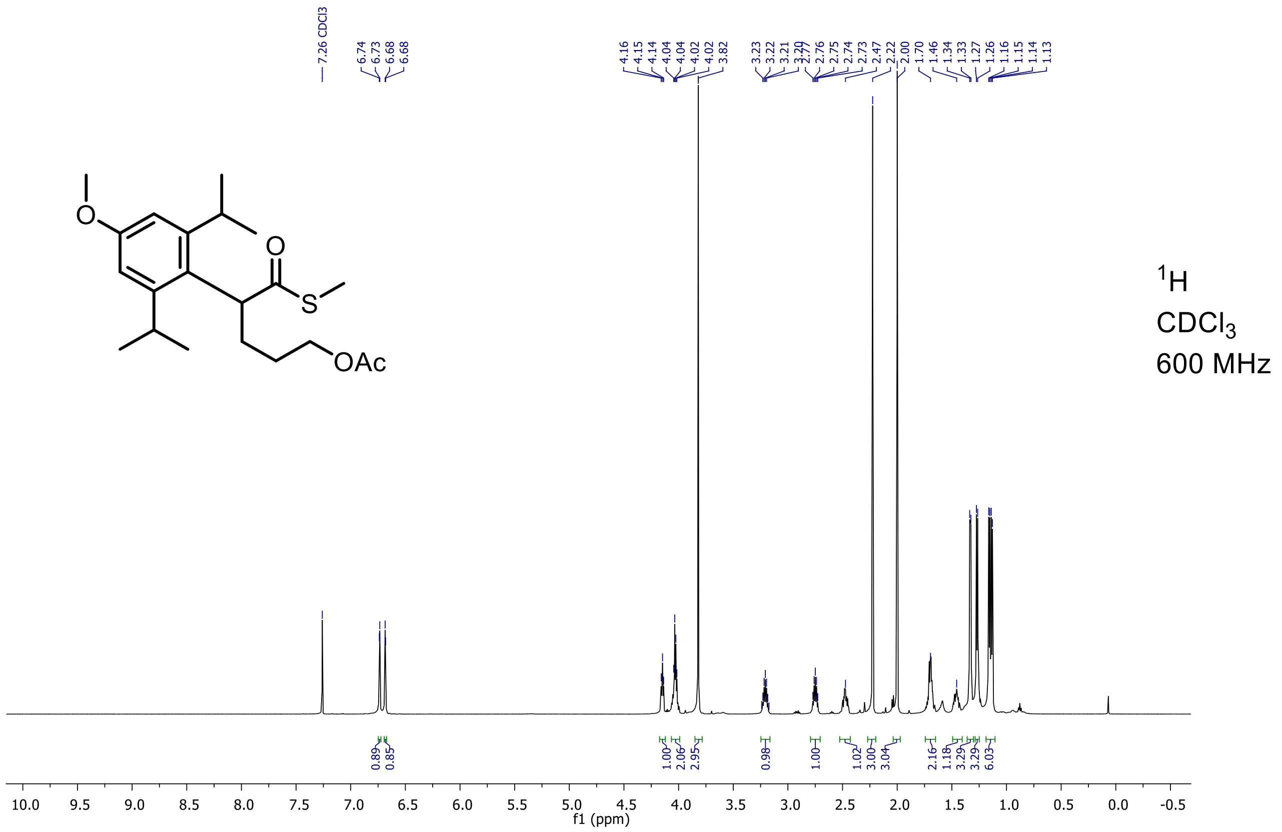

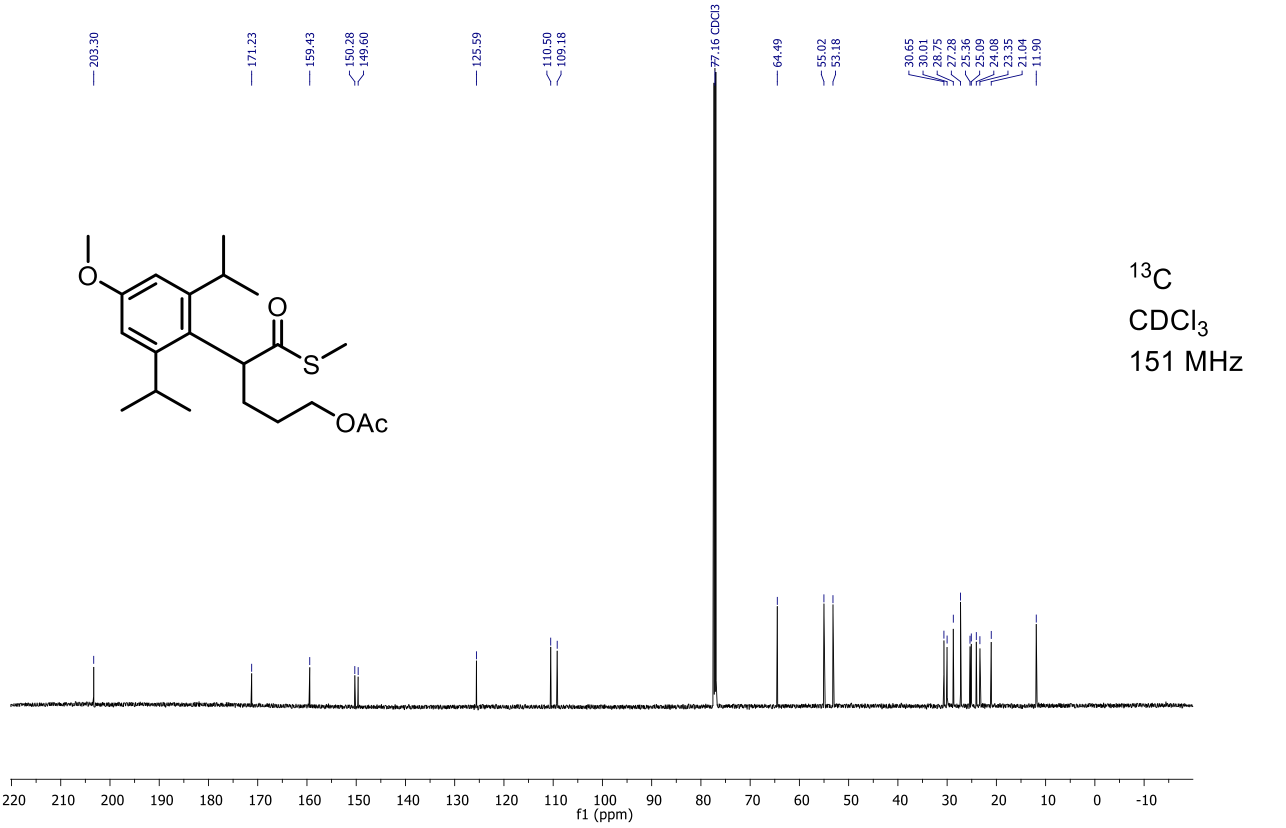

5hF – *S*-Phenyl 2-(2,6-diisopropyl-4-methoxyphenyl)hexanethioate

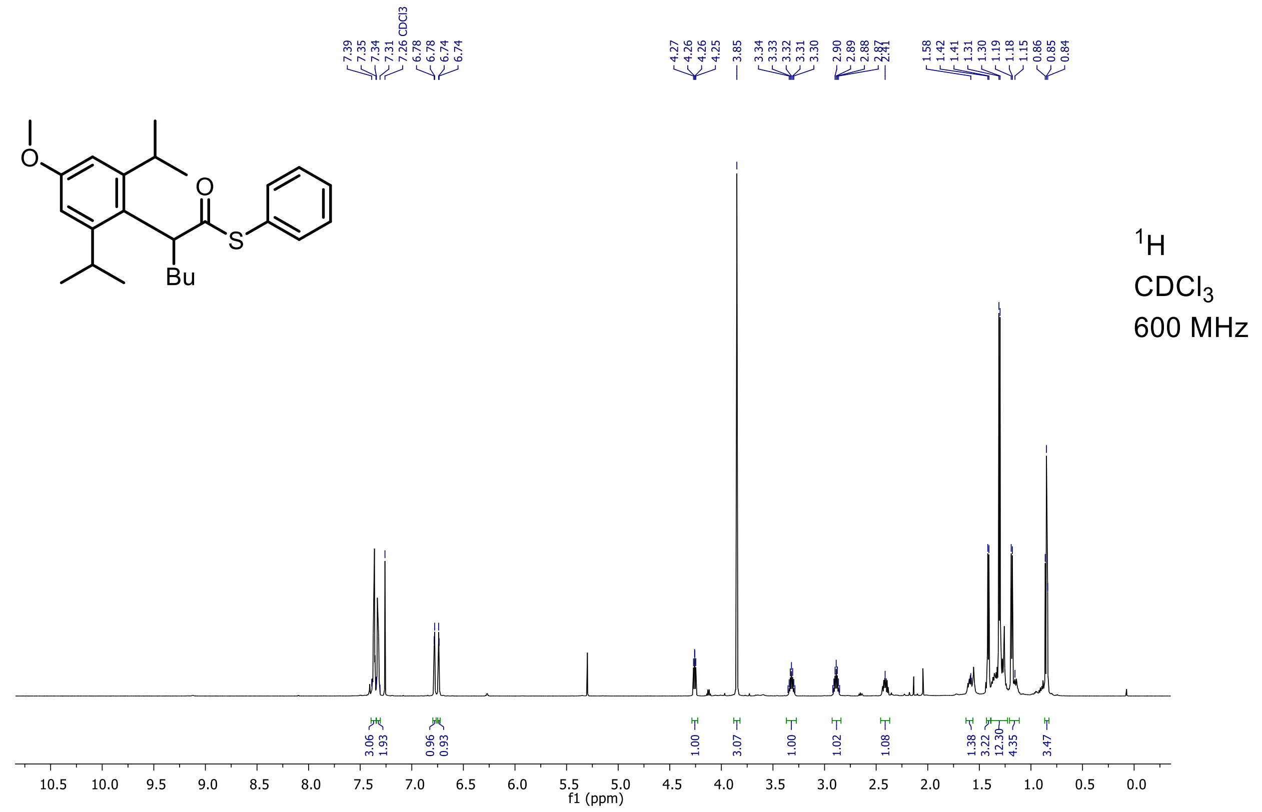

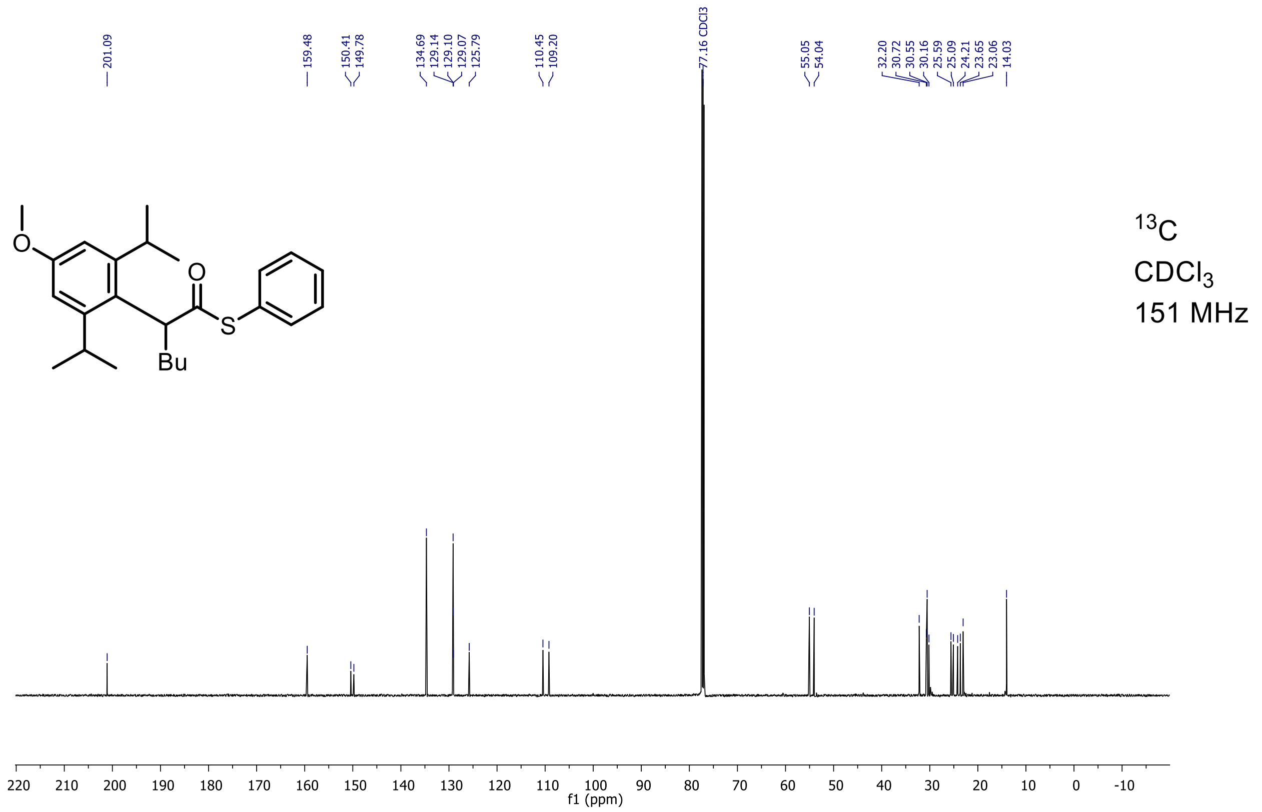

5hG – *S*-Phenyl 2-cyclopentyl-2-(2,6-diisopropyl-4-methoxyphenyl)ethanethioate

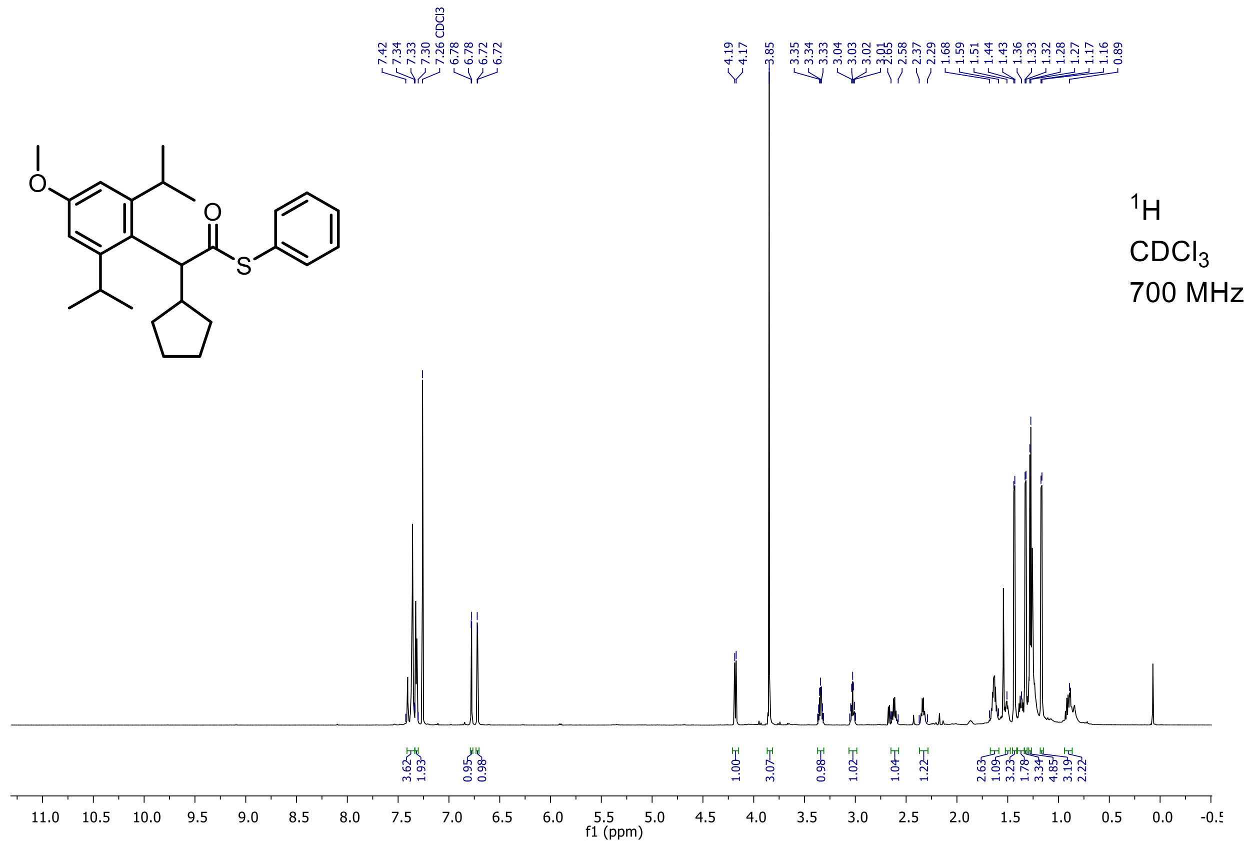

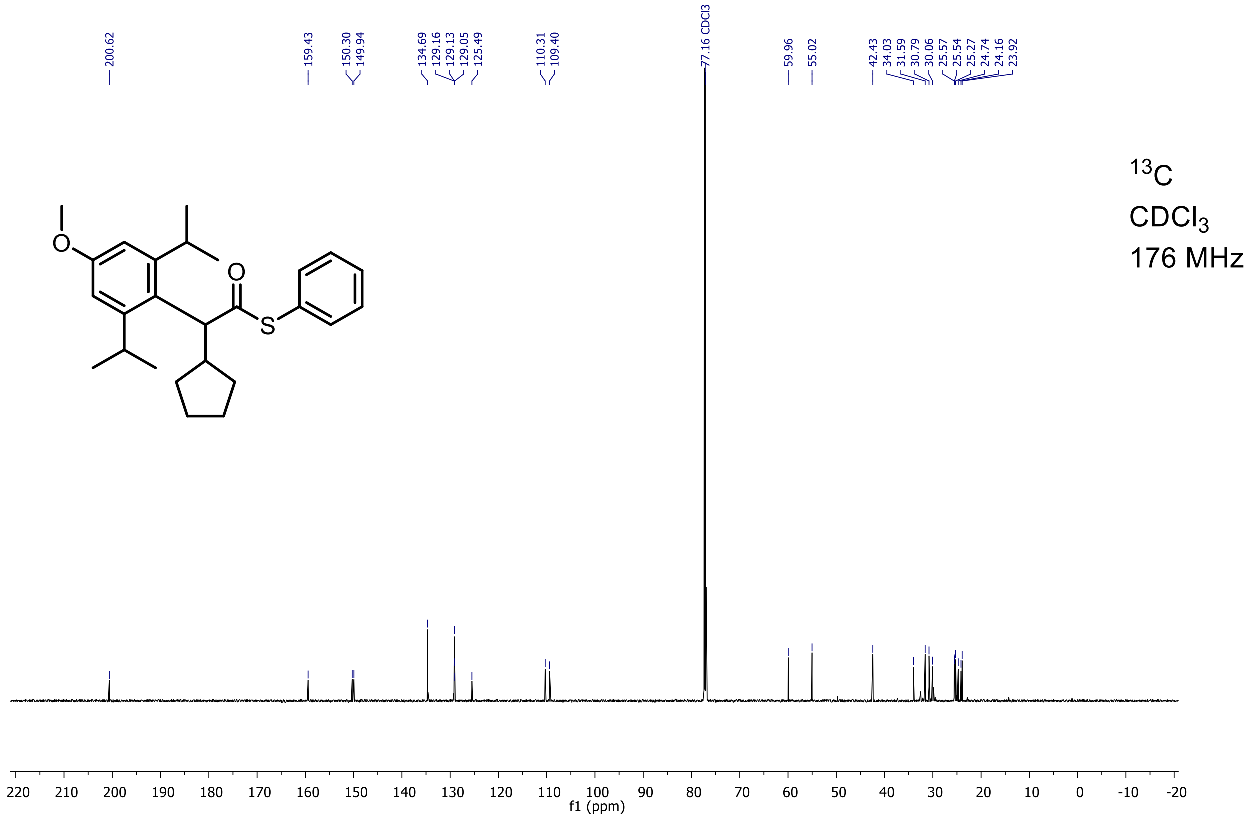

5hH – *S*-Phenyl 2-(2,6-diisopropyl-4-methoxyphenyl)-5-phenylpentanethioate

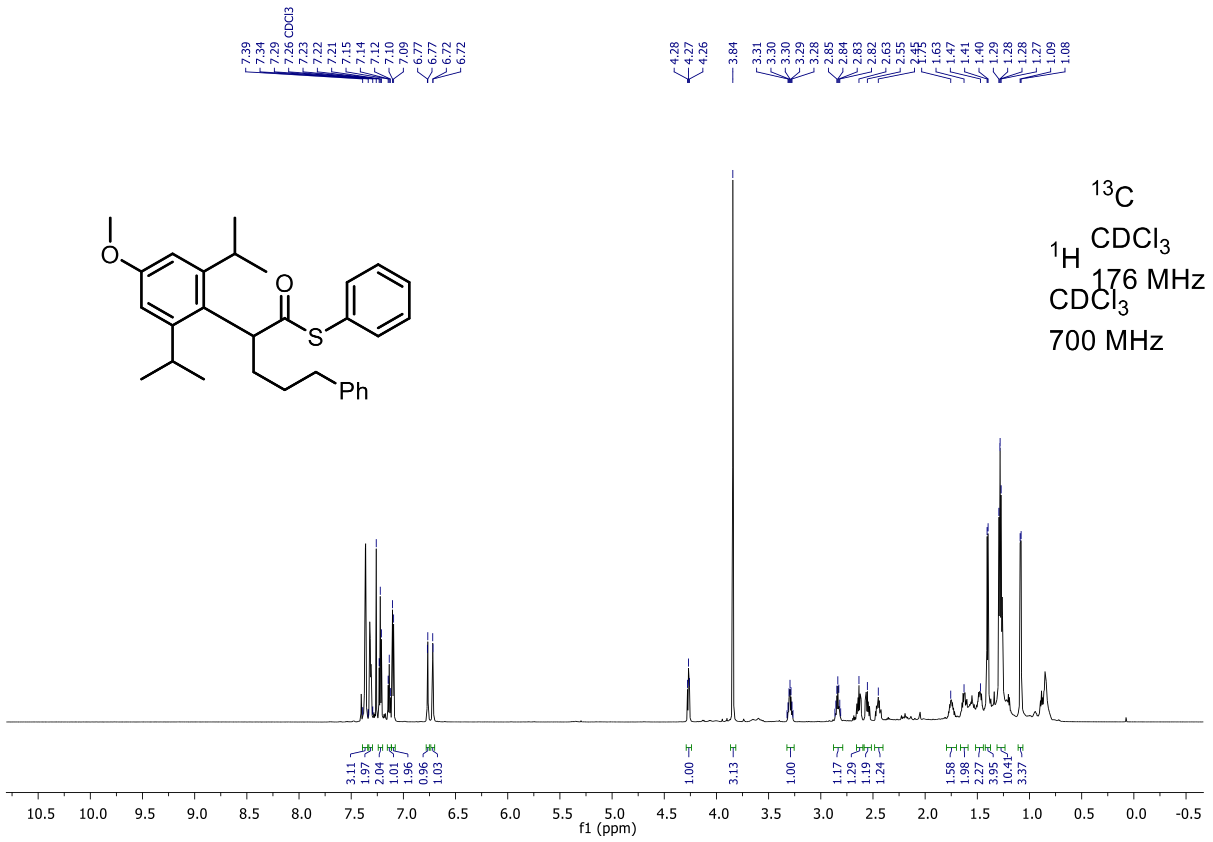

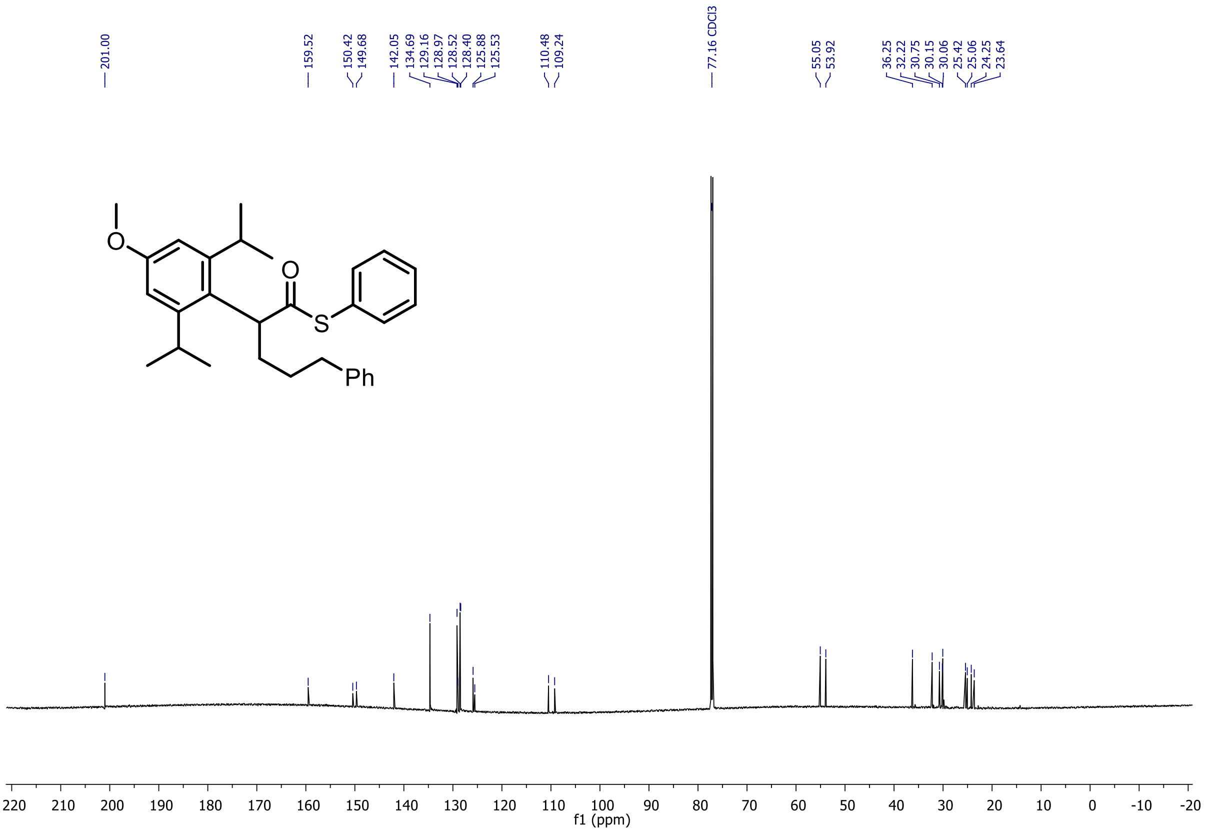

4aK – 2-(2,6-Dimethoxyphenyl)-*N*-methyl-5-phenyl-*N*-tosylpentanamide

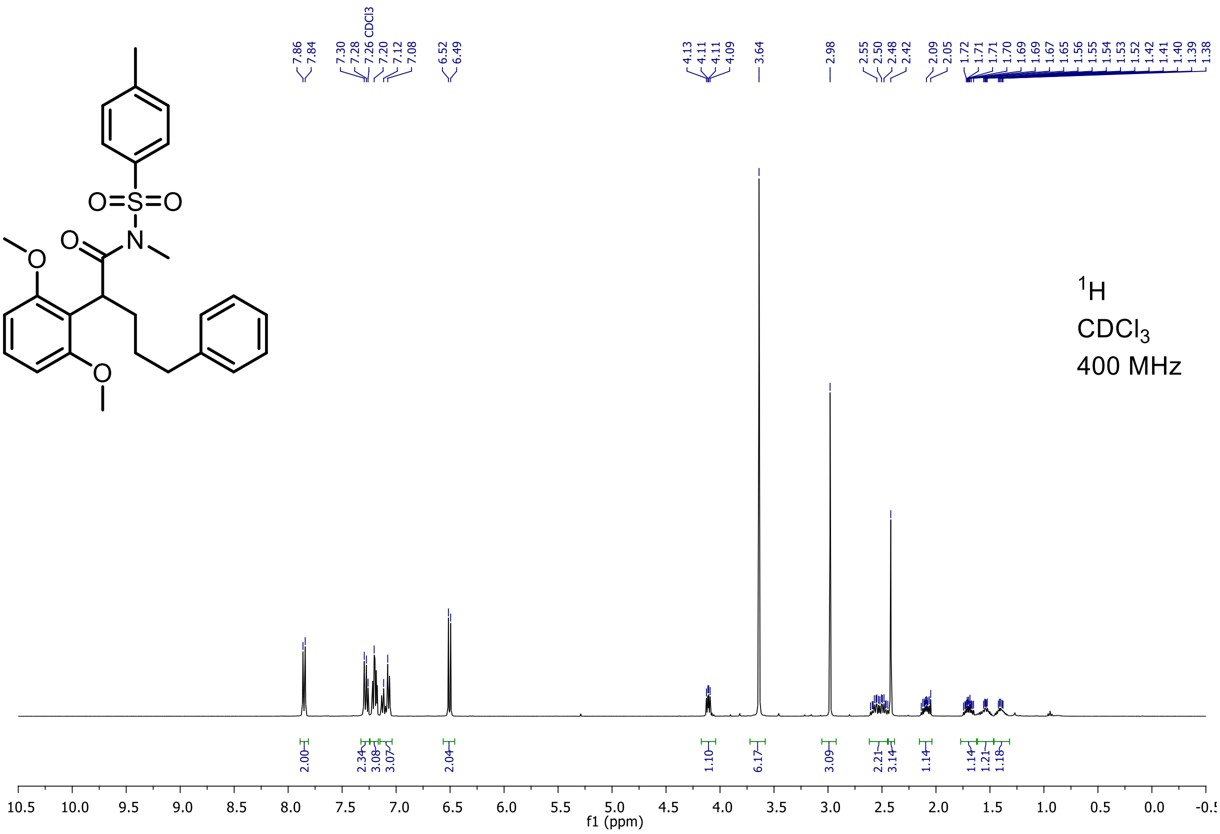

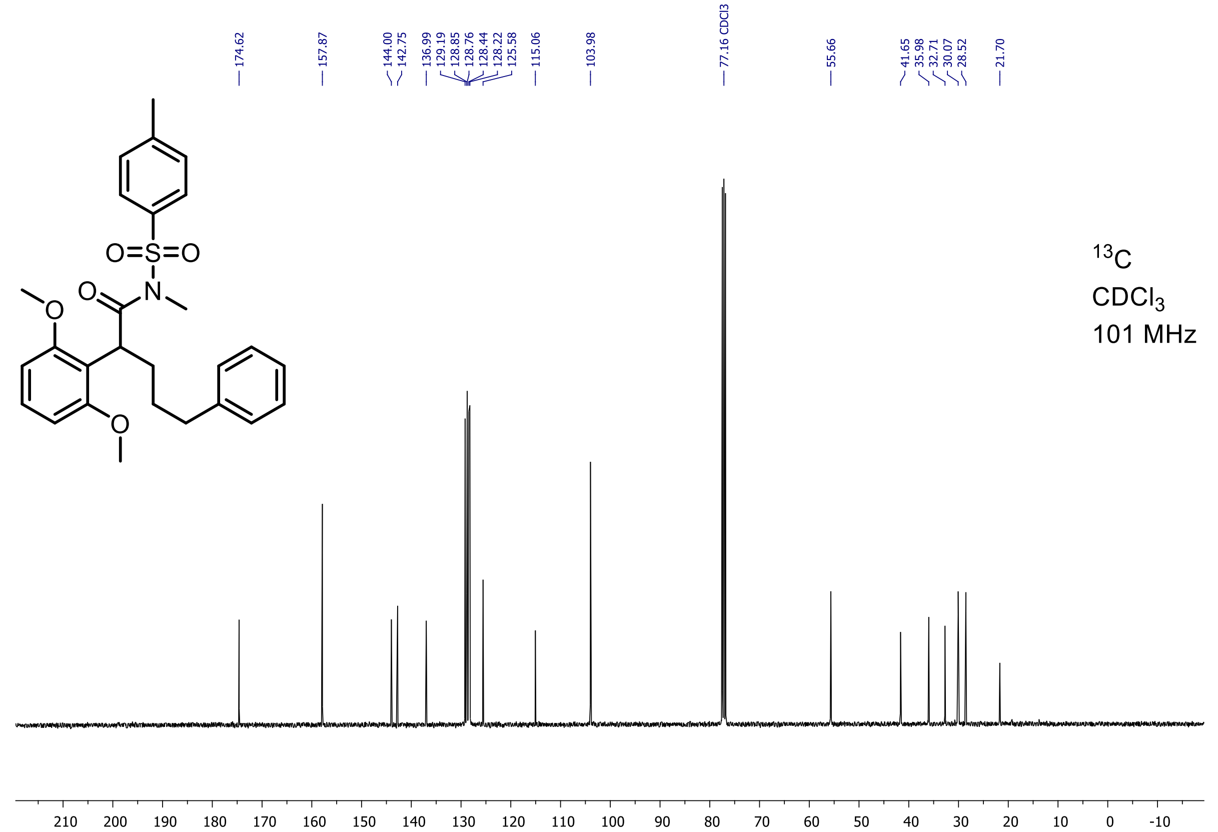

4aL – 2-Chloro-2-(2,6-dimethoxyphenyl)-*N*-phenyl-*N*-tosylacetamide

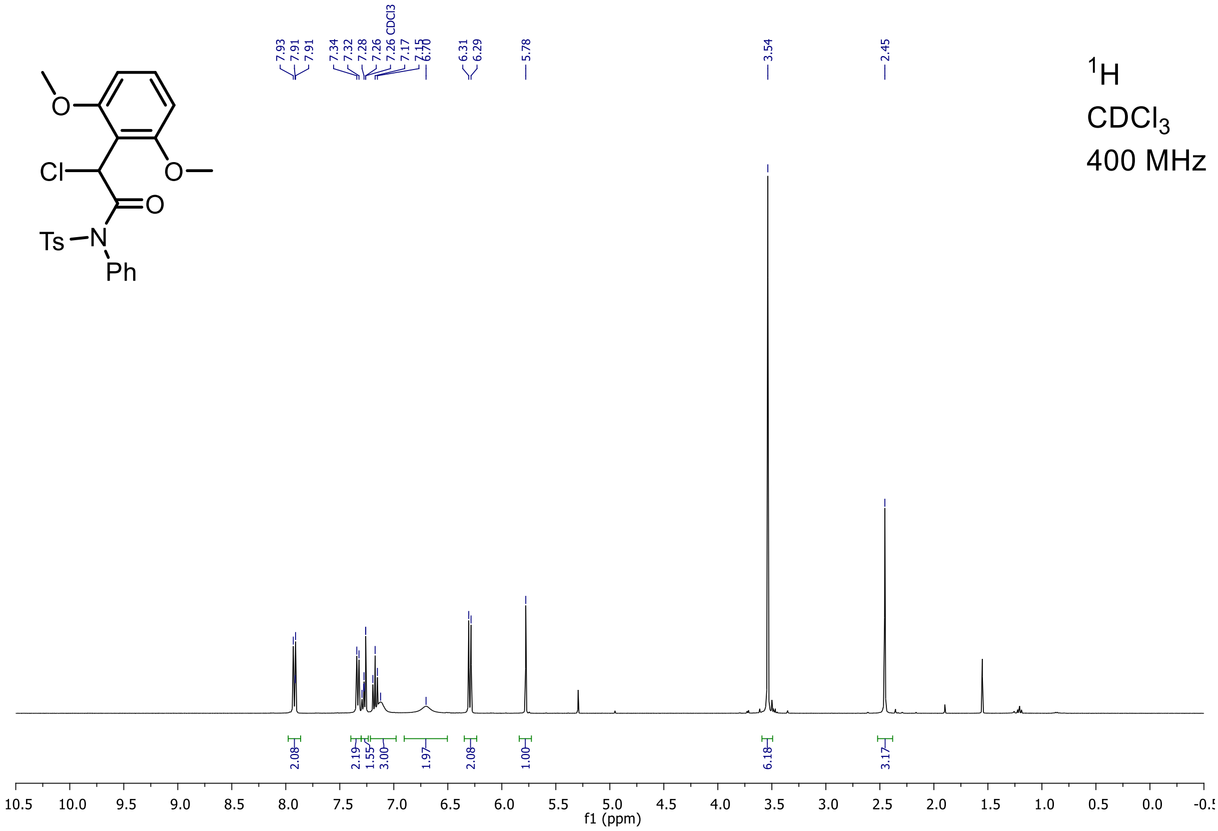

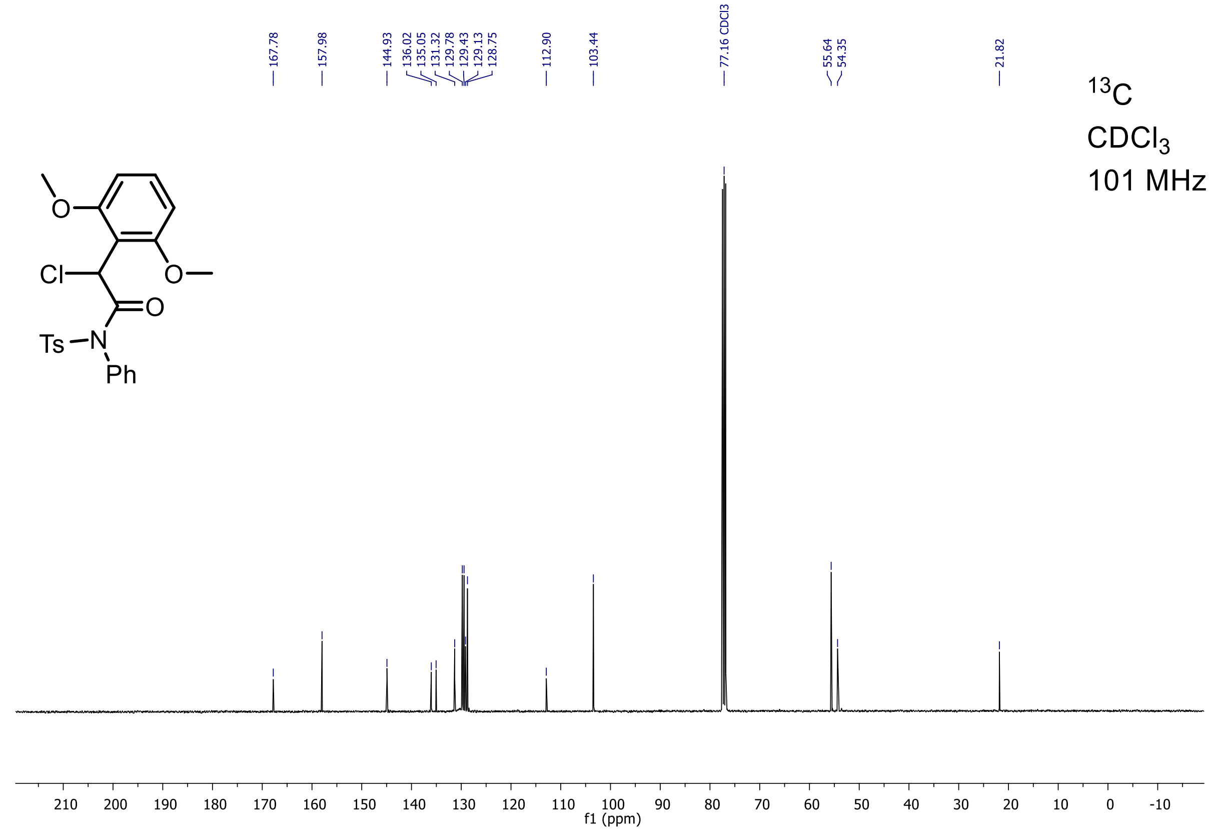

4aM – 2-(2,6-Dimethoxyphenyl)-*N*-phenyl-2-(pyridin-2-yl)-*N*-tosylacetamide

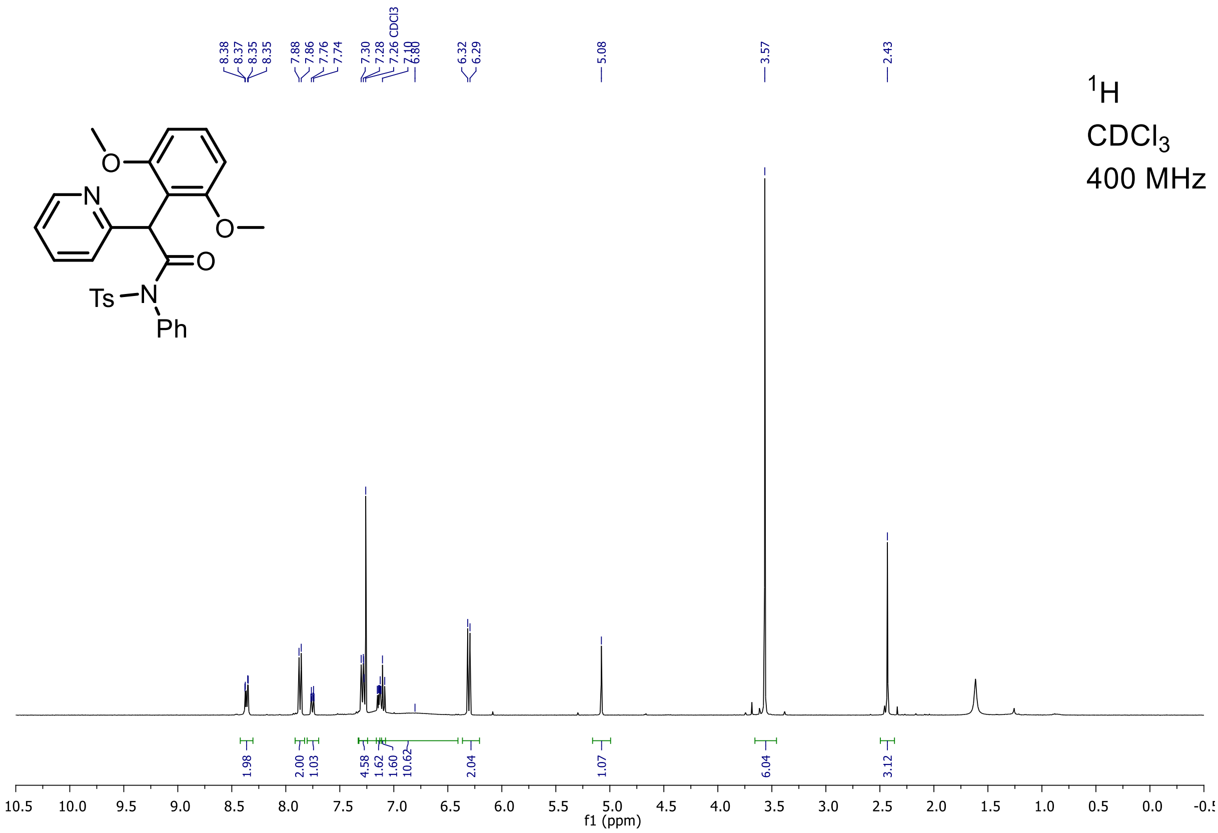

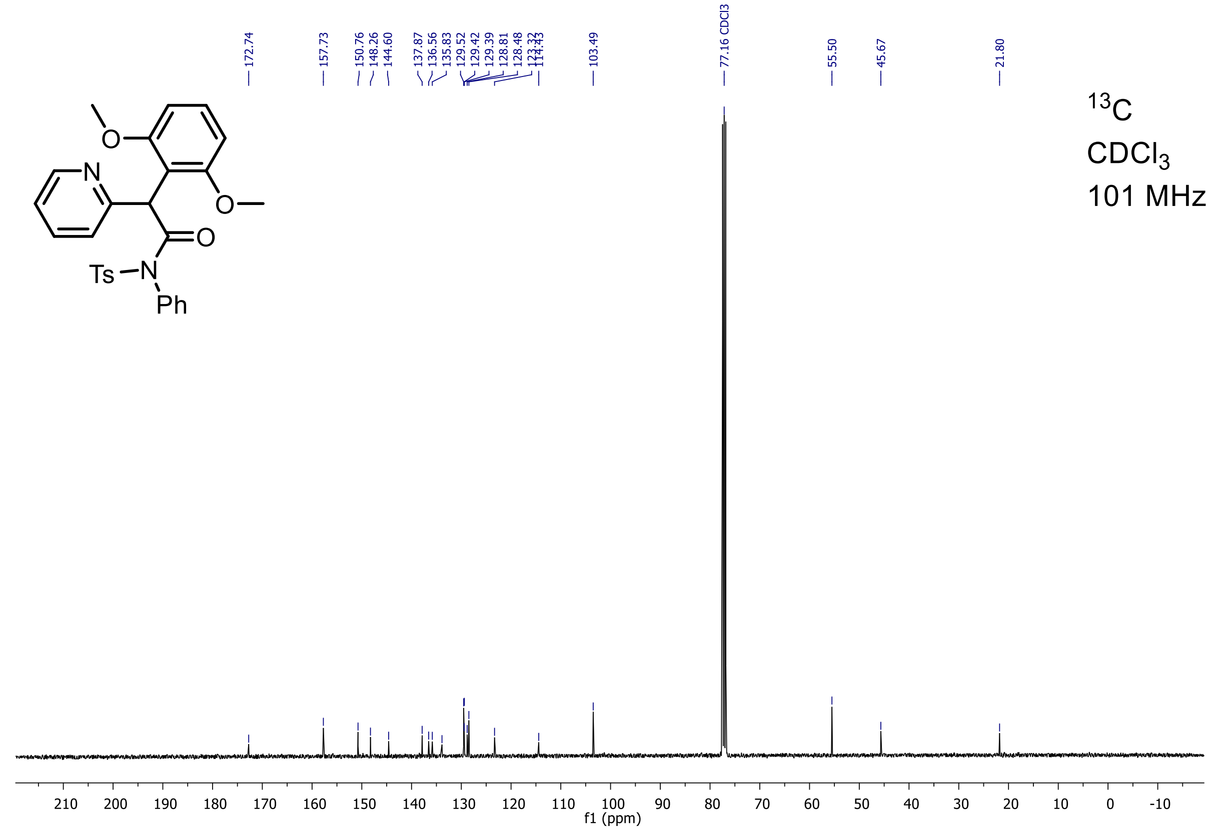

9aA – Ethyl 2-(2,6-dimethoxyphenyl)acetate

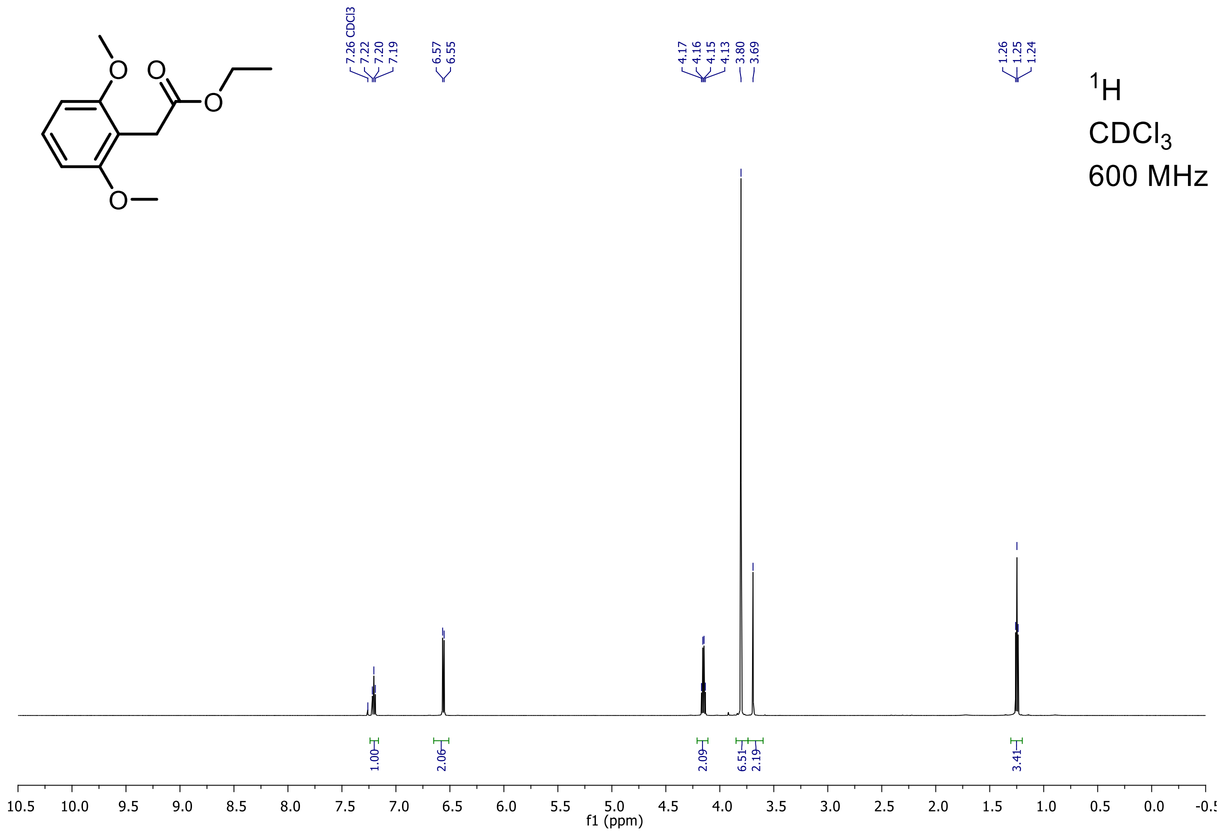

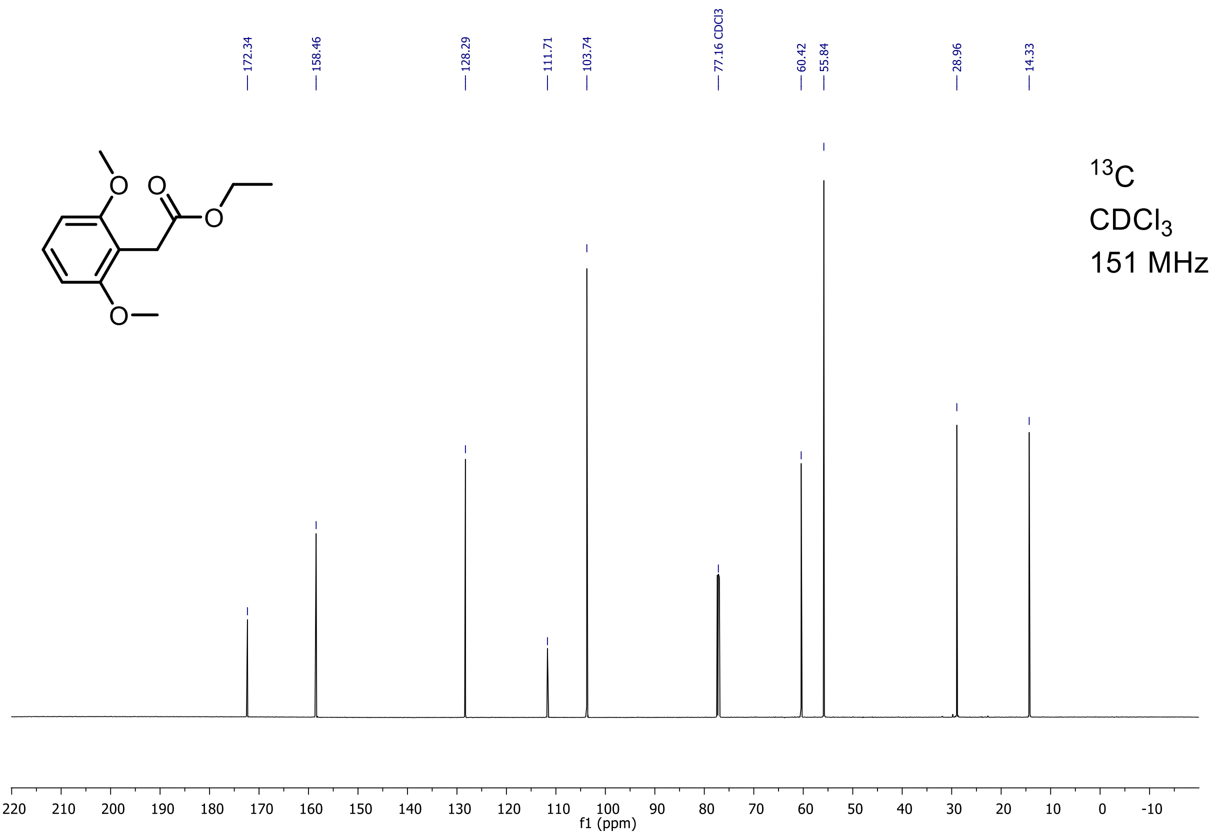

10aA – 2-(2,6-Dimethoxyphenyl)-1-(4-methoxyphenyl)ethan-1-one

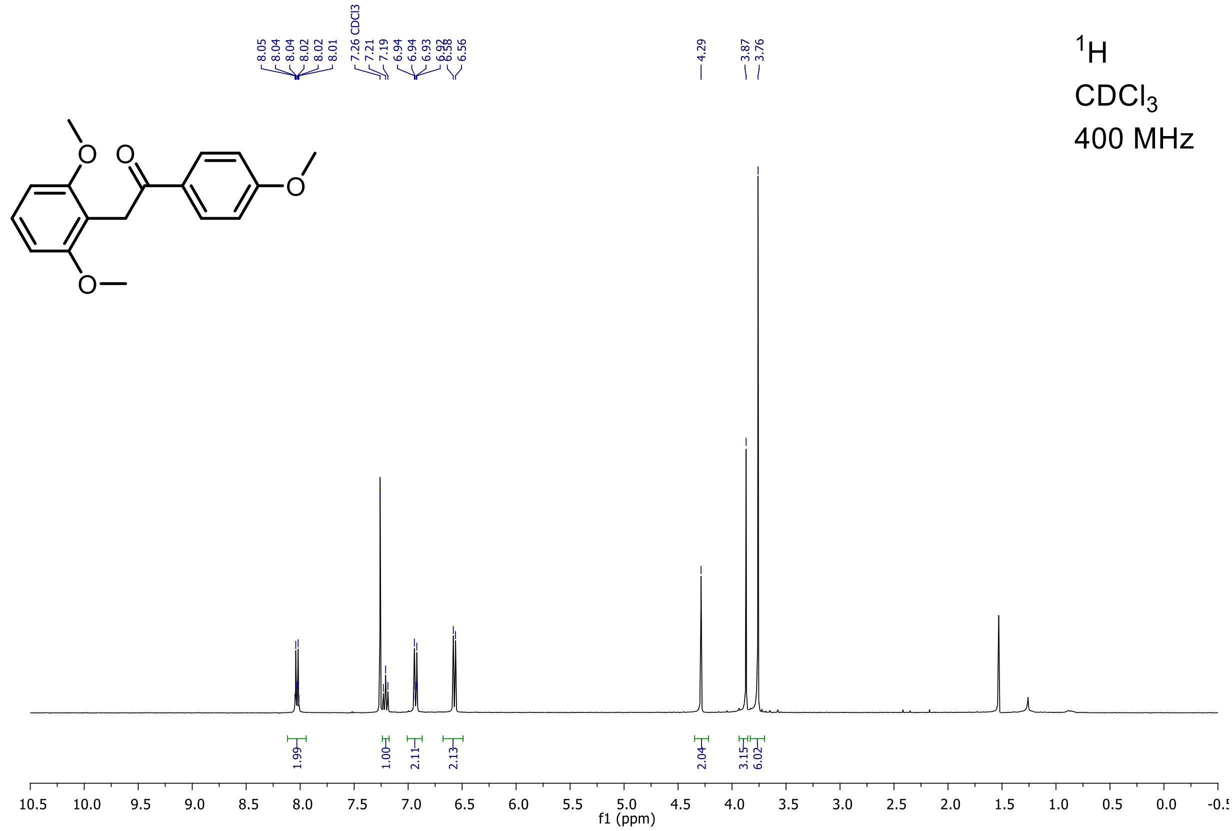

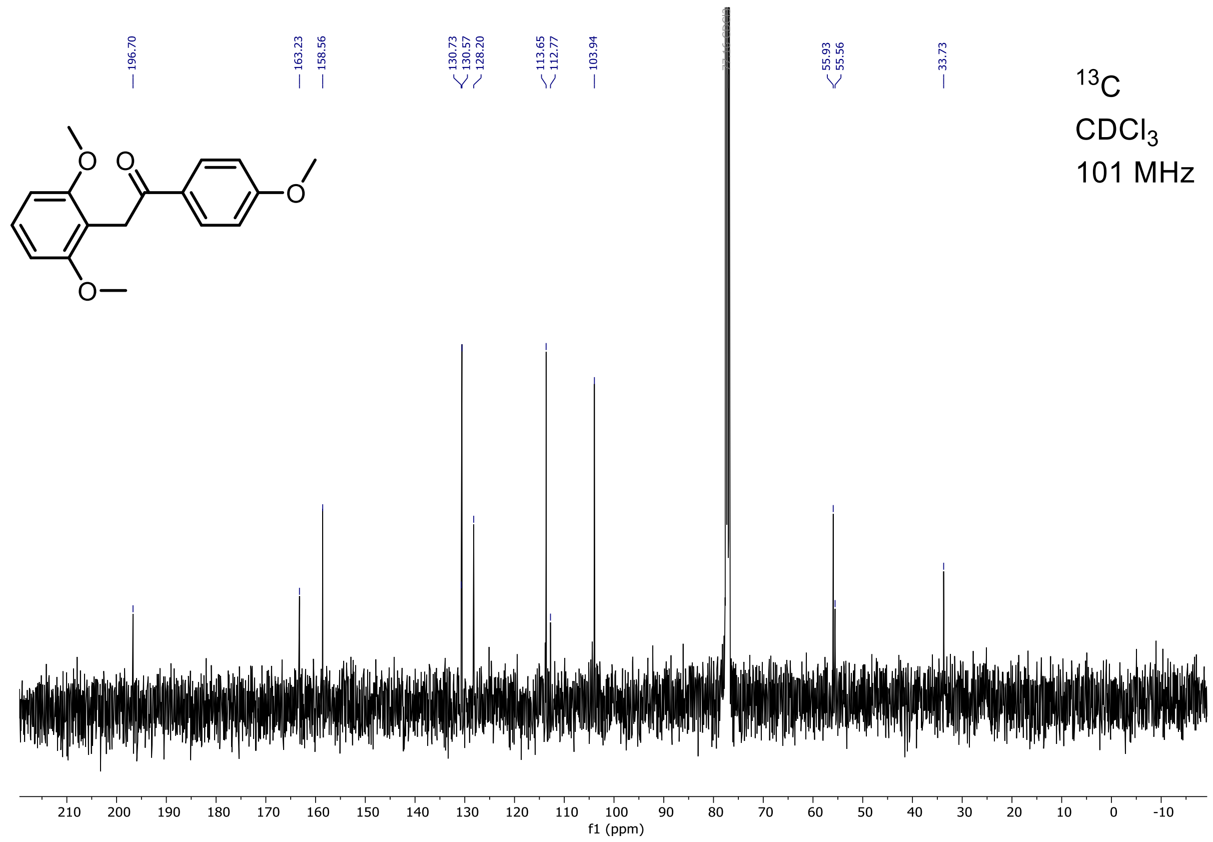

11aA – 2-(2,6-Dimethoxyphenyl)-*N*,*N*-dimethyl-4-phenylbutanamide

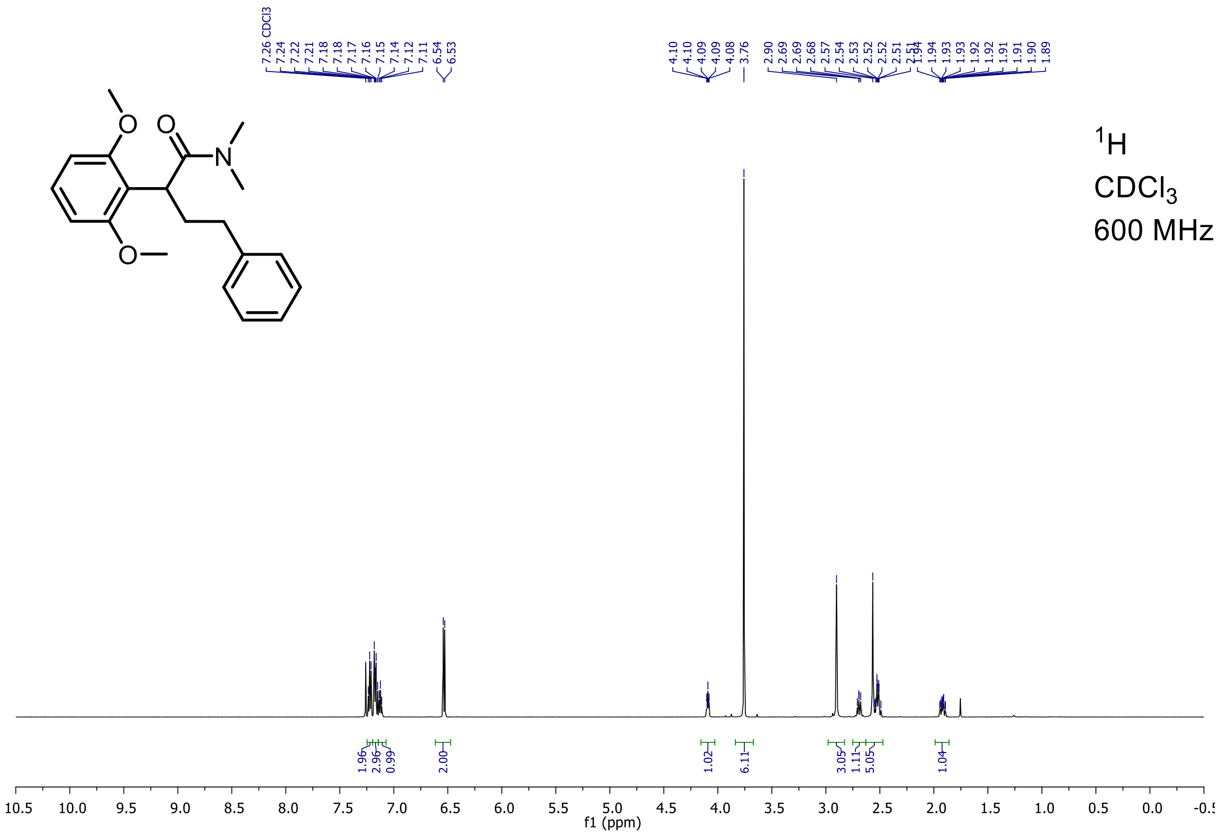

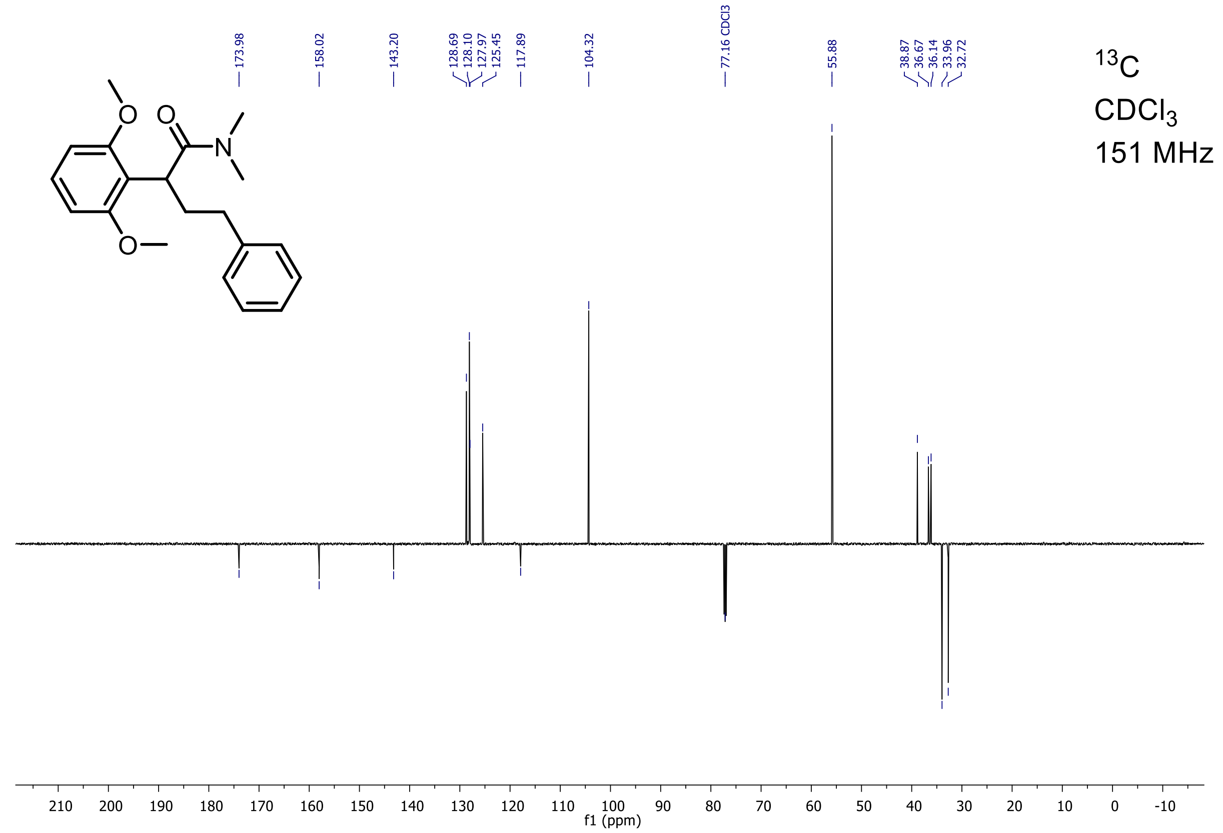

4mA – 3-(2-(2,4-Dimethoxyphenyl)hexanoyl)oxazolidin-2-one

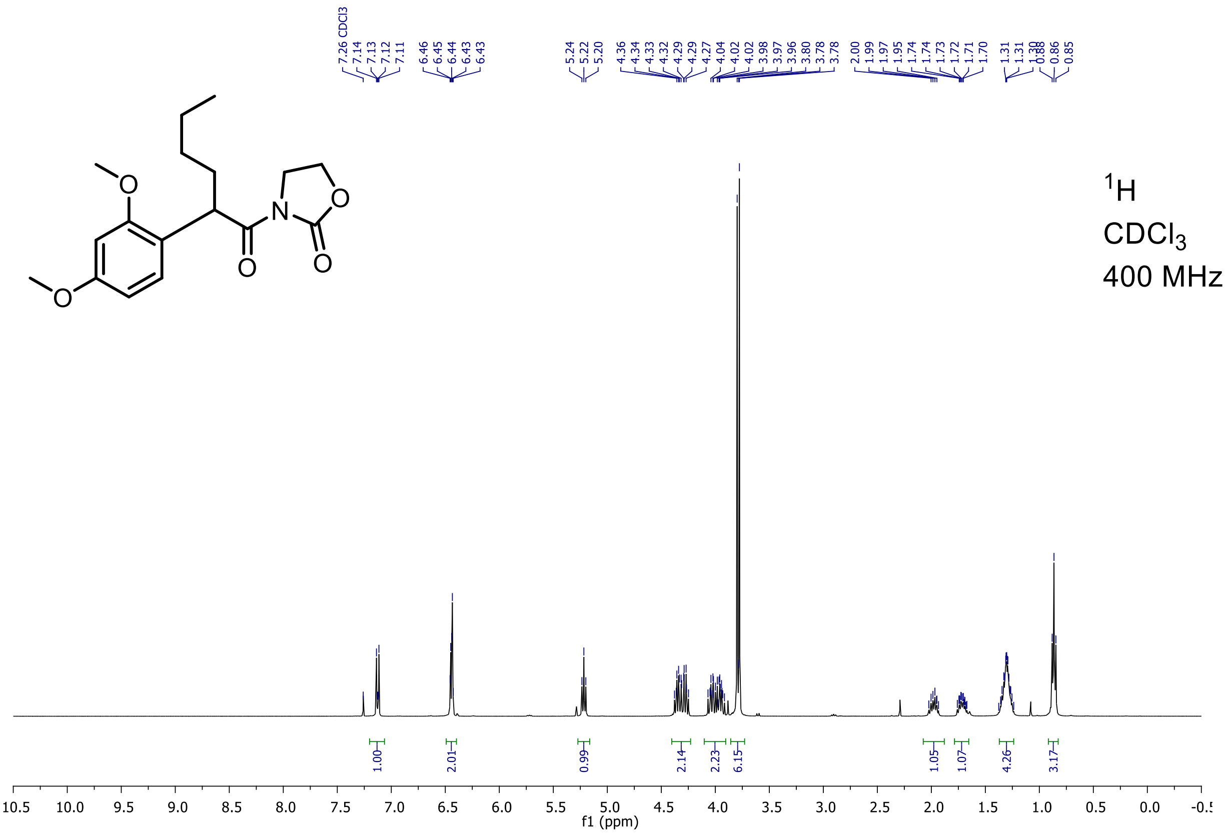

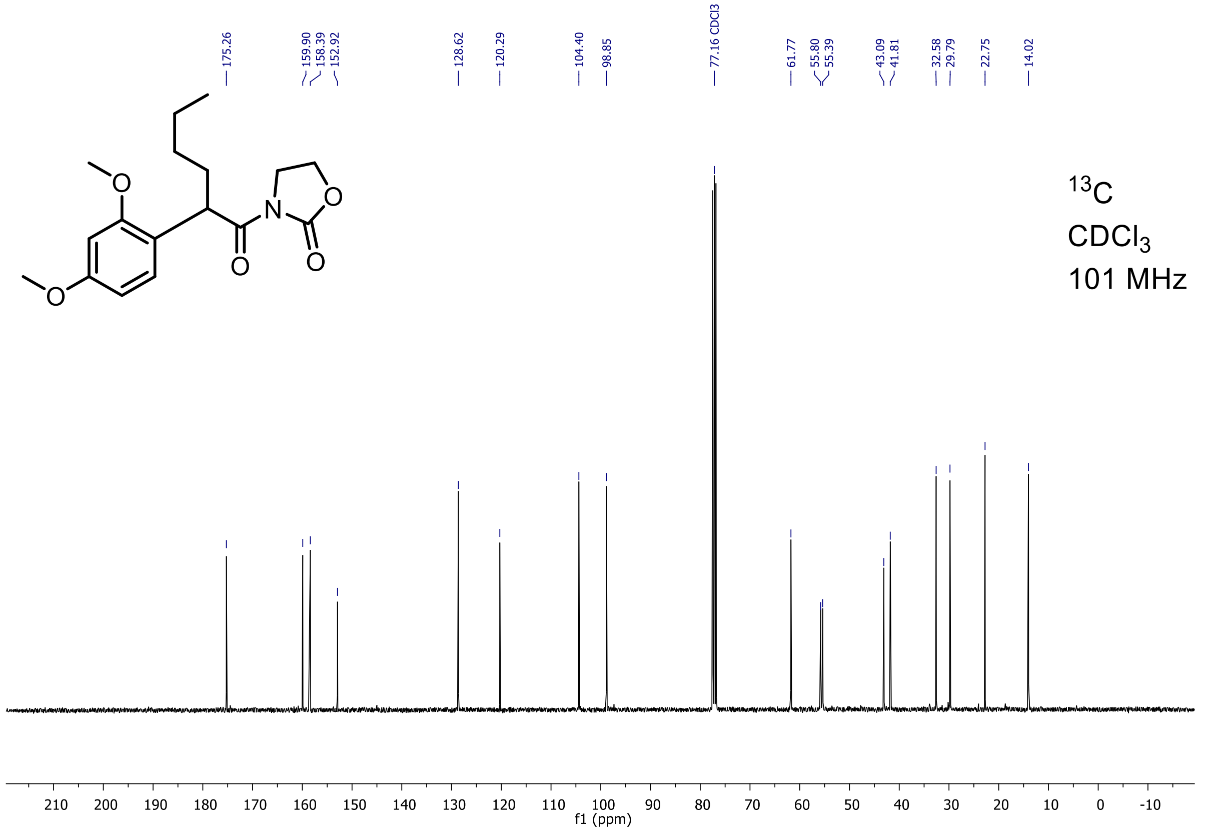

4nA – 3-(2-(2,4-Bis(allyloxy)phenyl)hexanoyl)oxazolidin-2-one

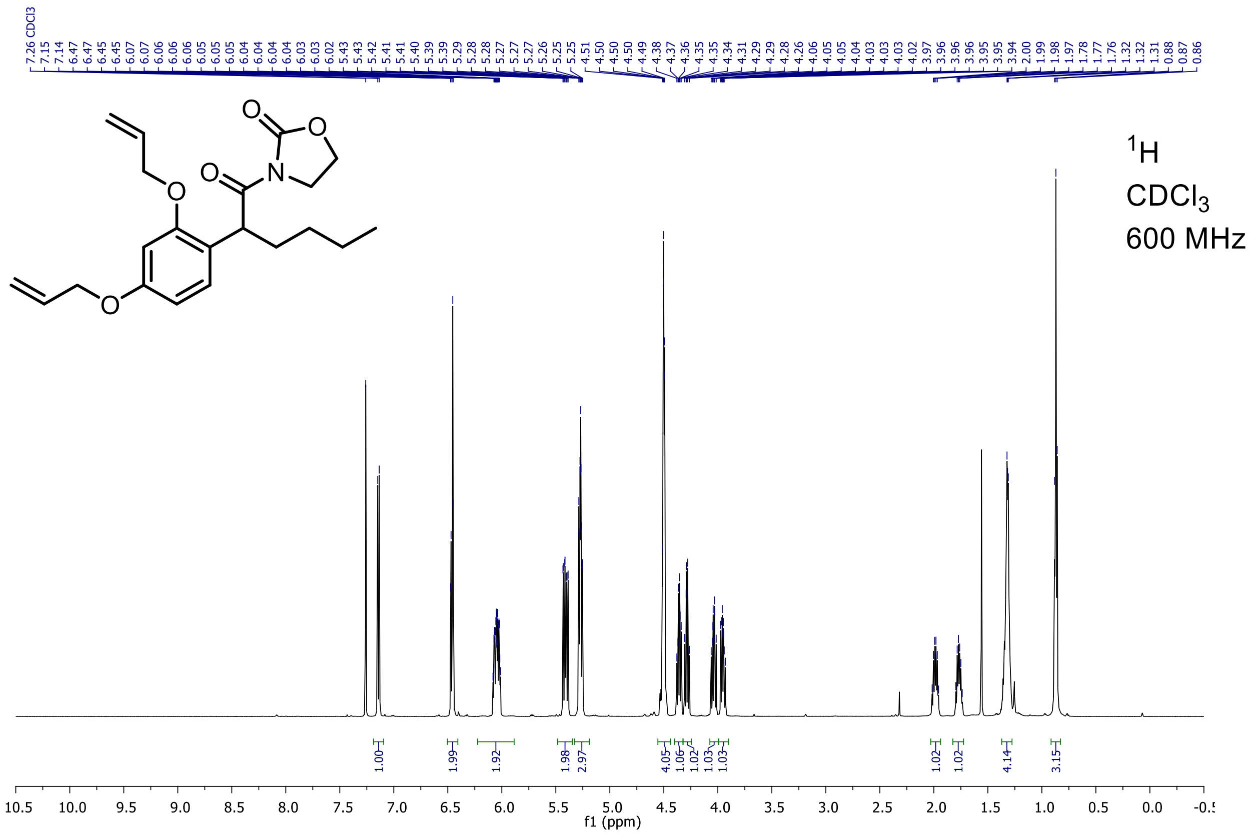

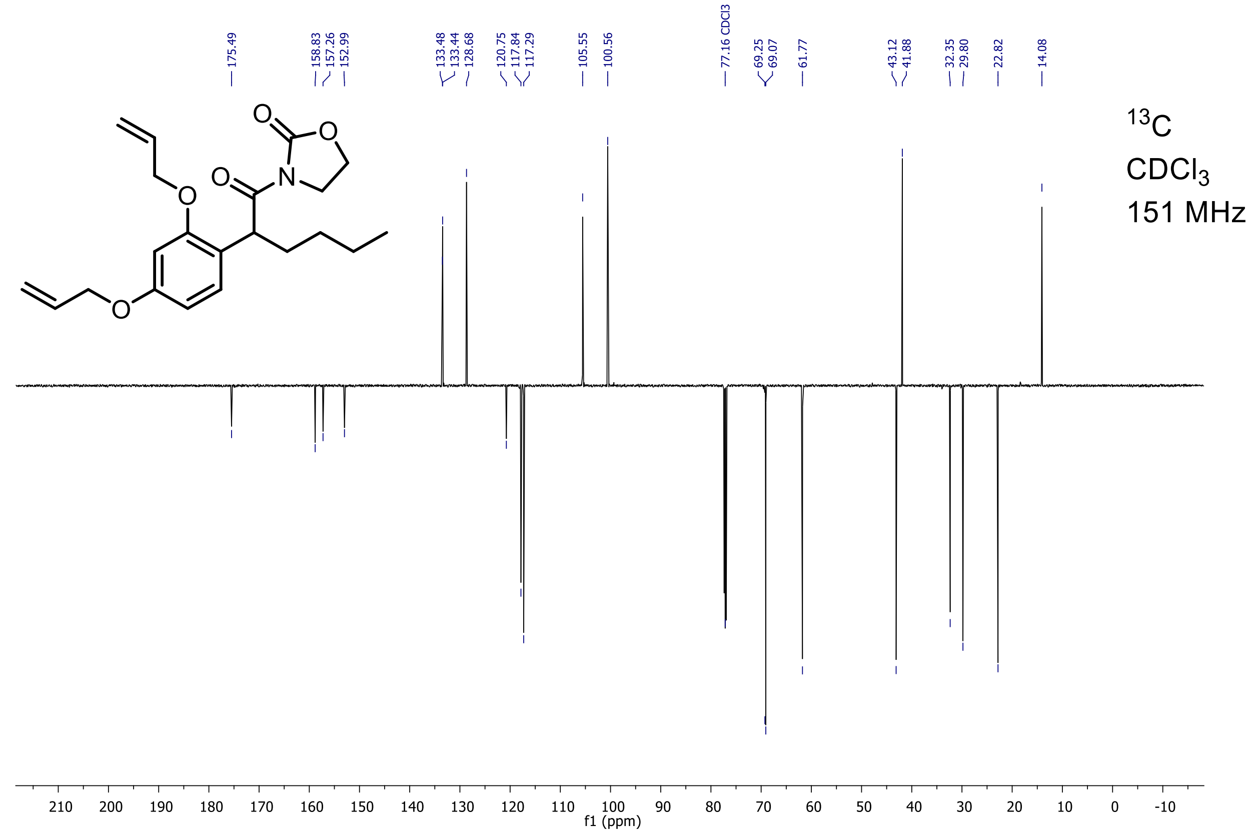

4oA – 3-[2-(4-hydroxy-2-methylphenyl)hexanoyl]oxazolidin-2-one

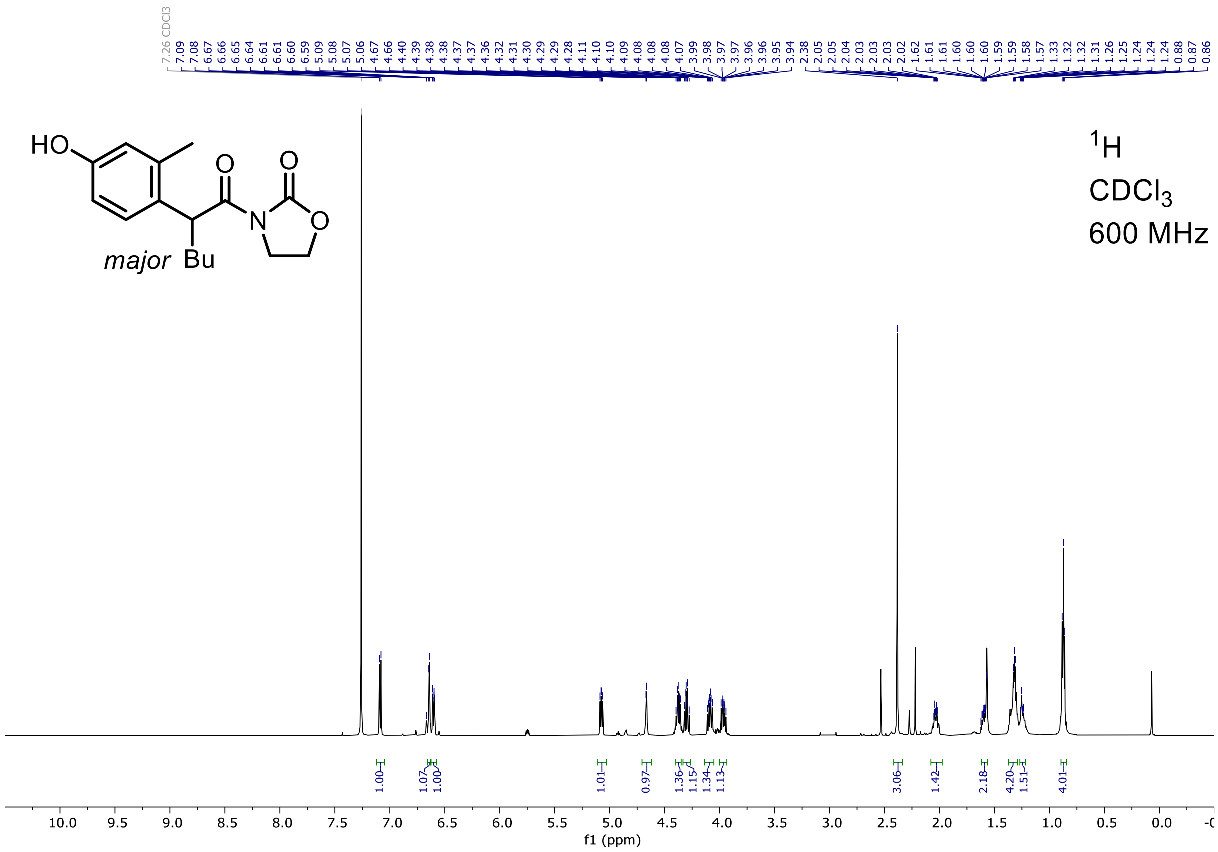

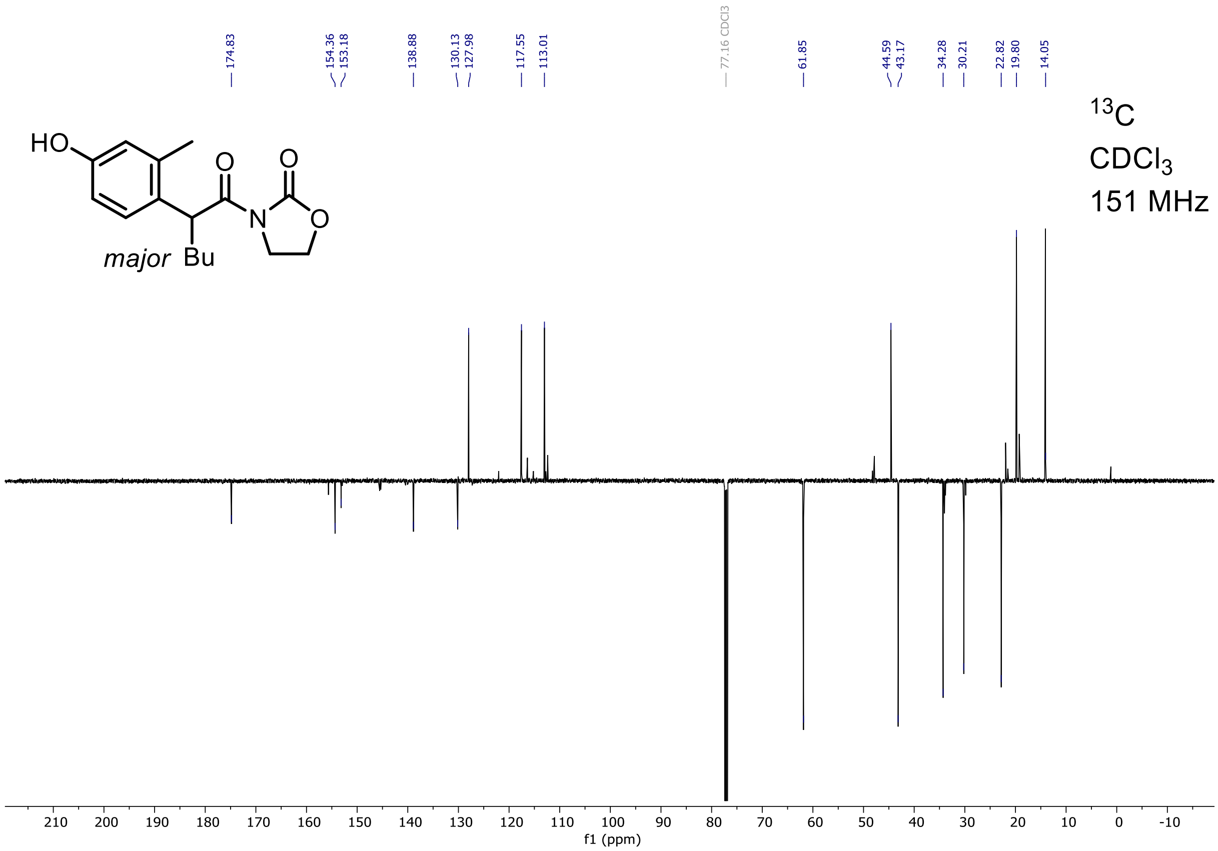

4oA’ – 3-{2-[5-hydroxy-3-methyl-2-(methylthio)phenyl]hexanoyl}oxazolidin-2-one

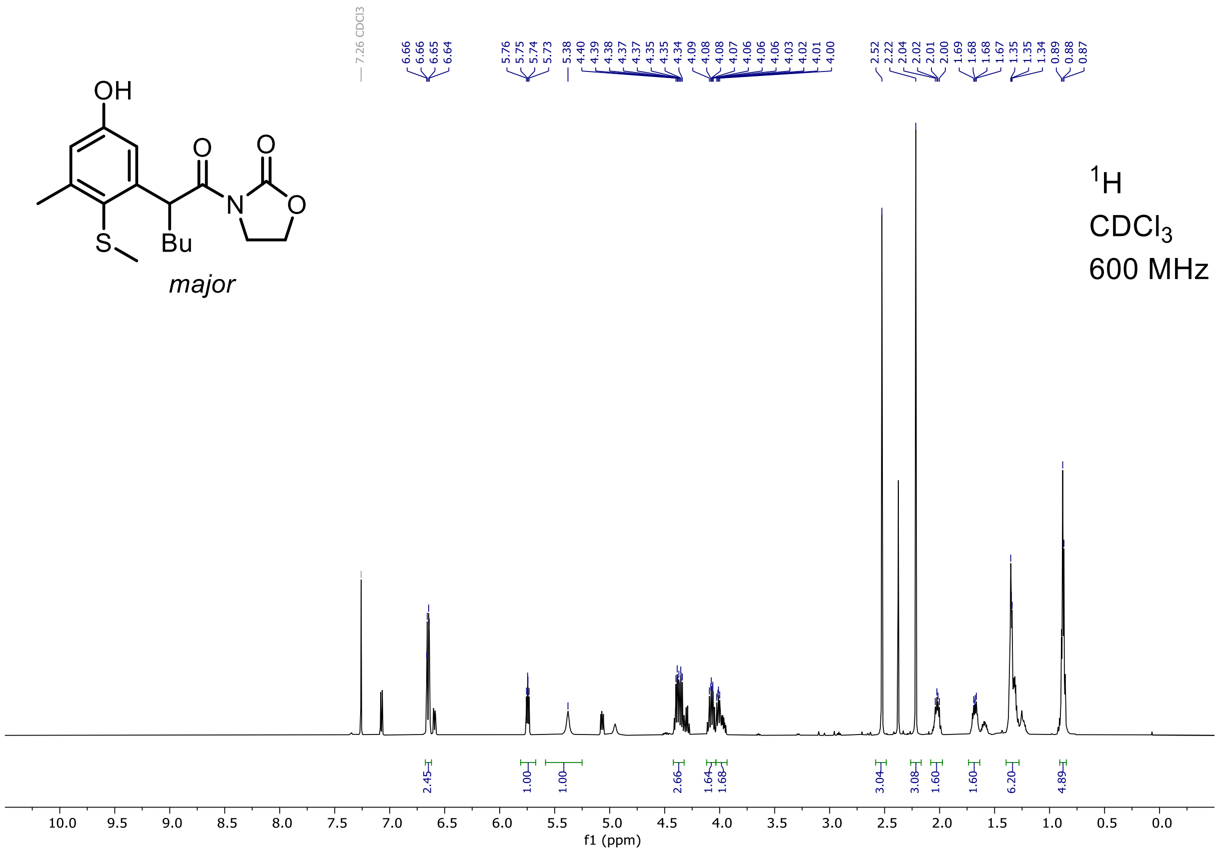

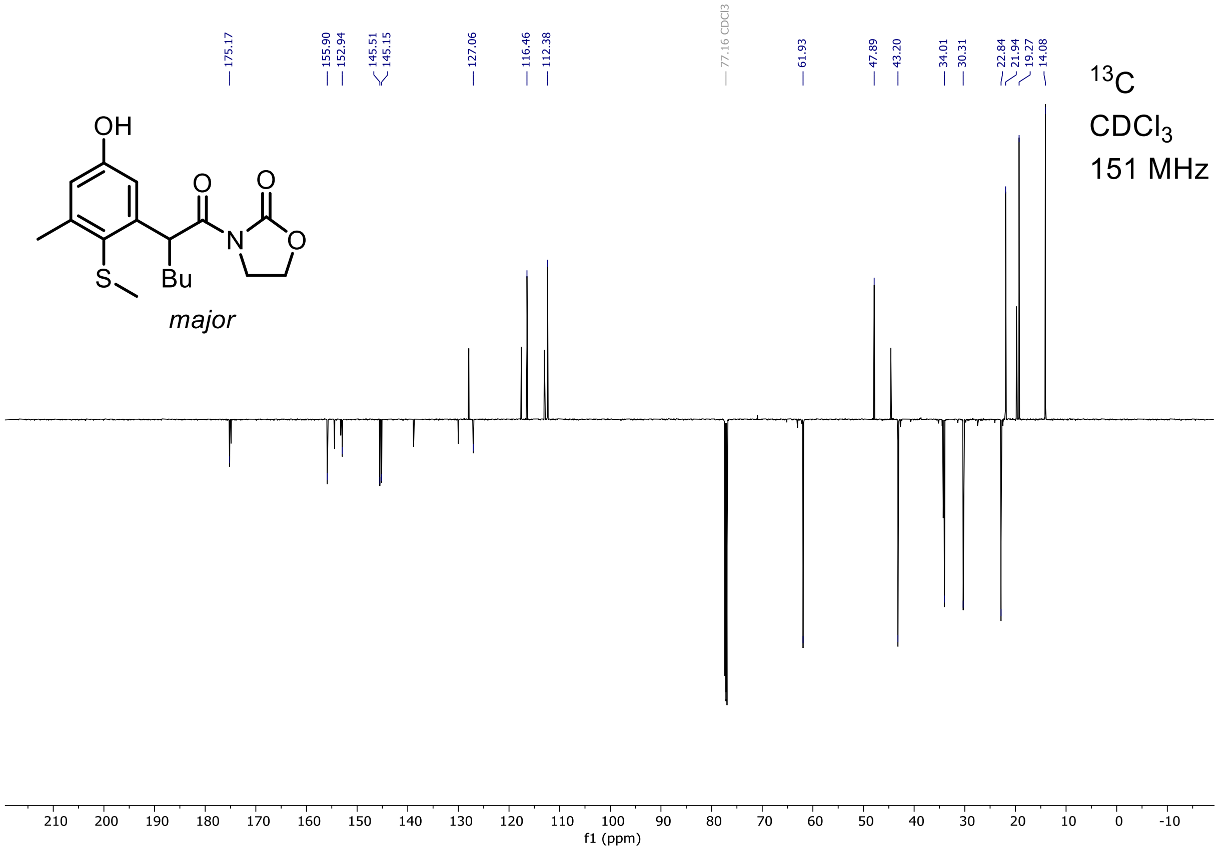

4pA – 3-[2-(4-hydroxyphenyl)hexanoyl]oxazolidin-2-one

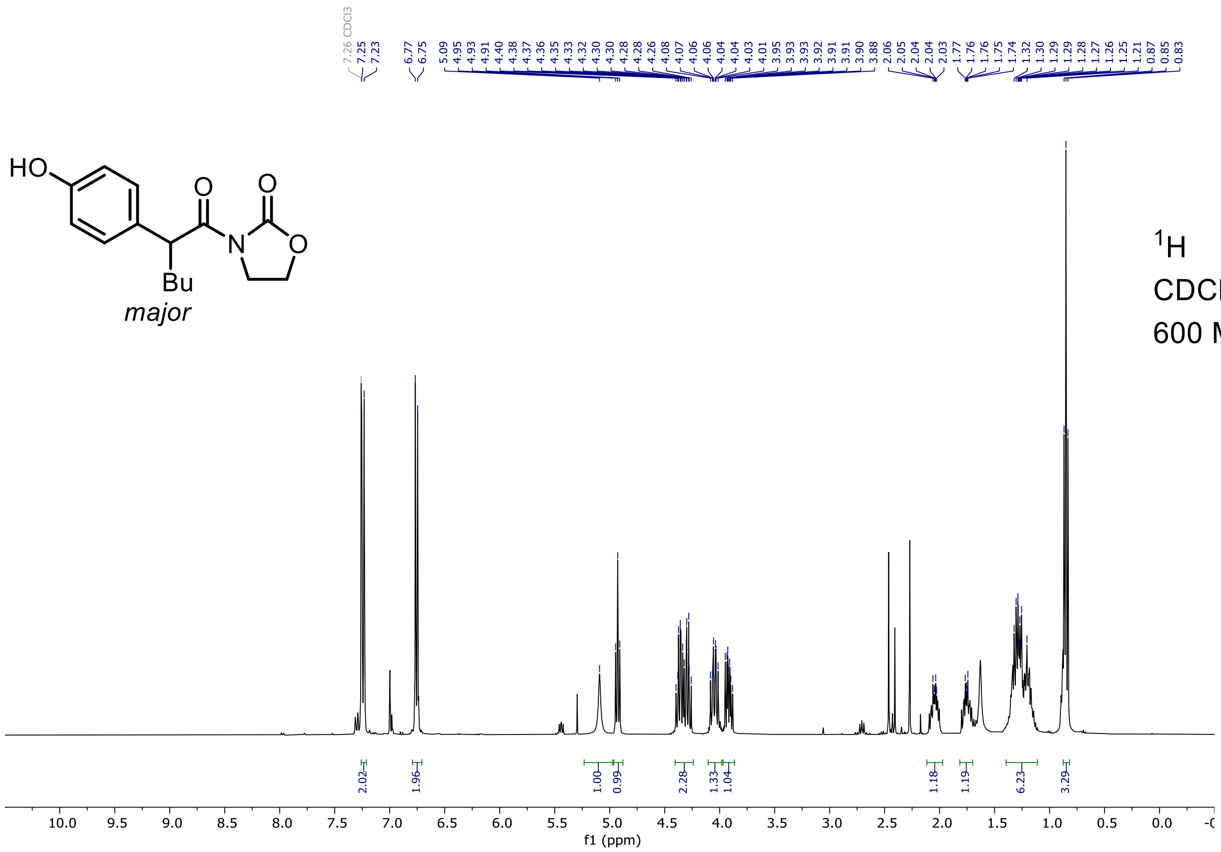

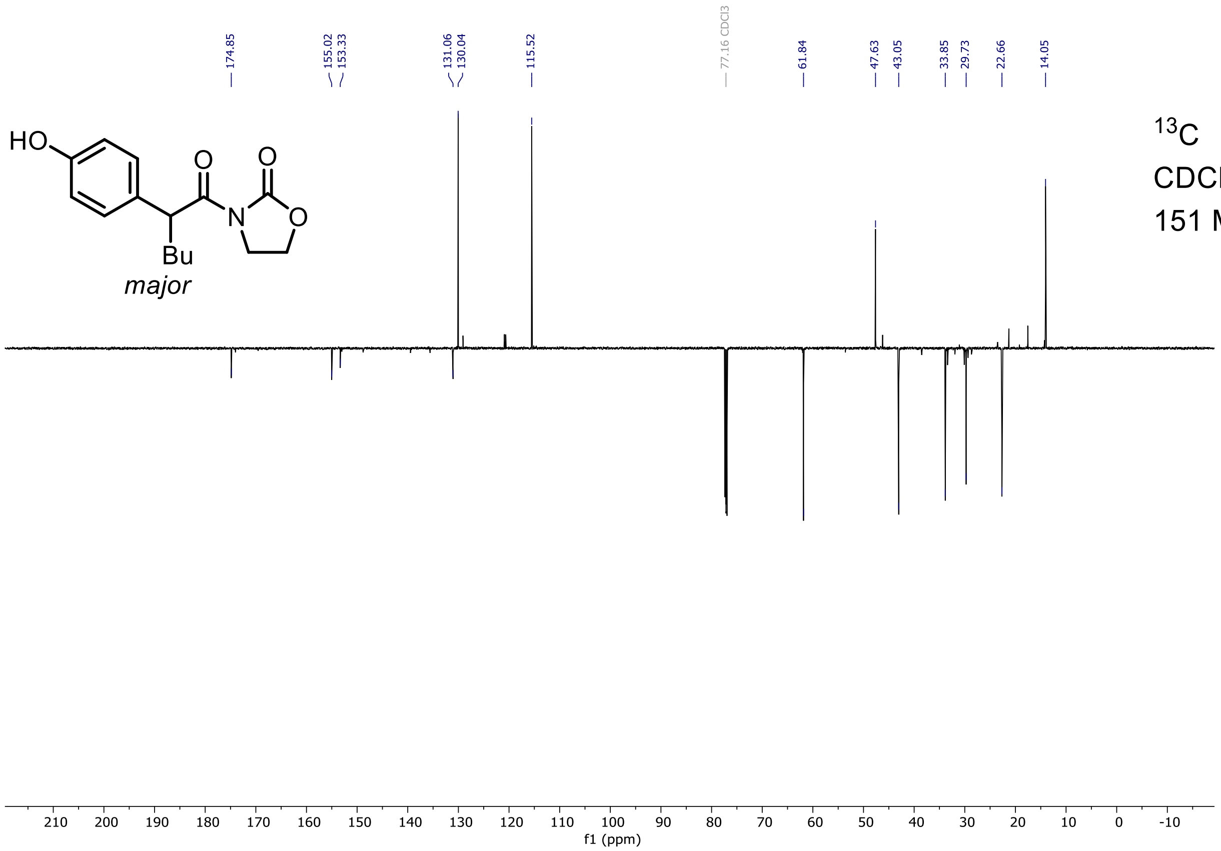

4pA' – 3-{2-[5-hydroxy-2-(methylthio)phenyl]hexanoyl}oxazolidin-2-one

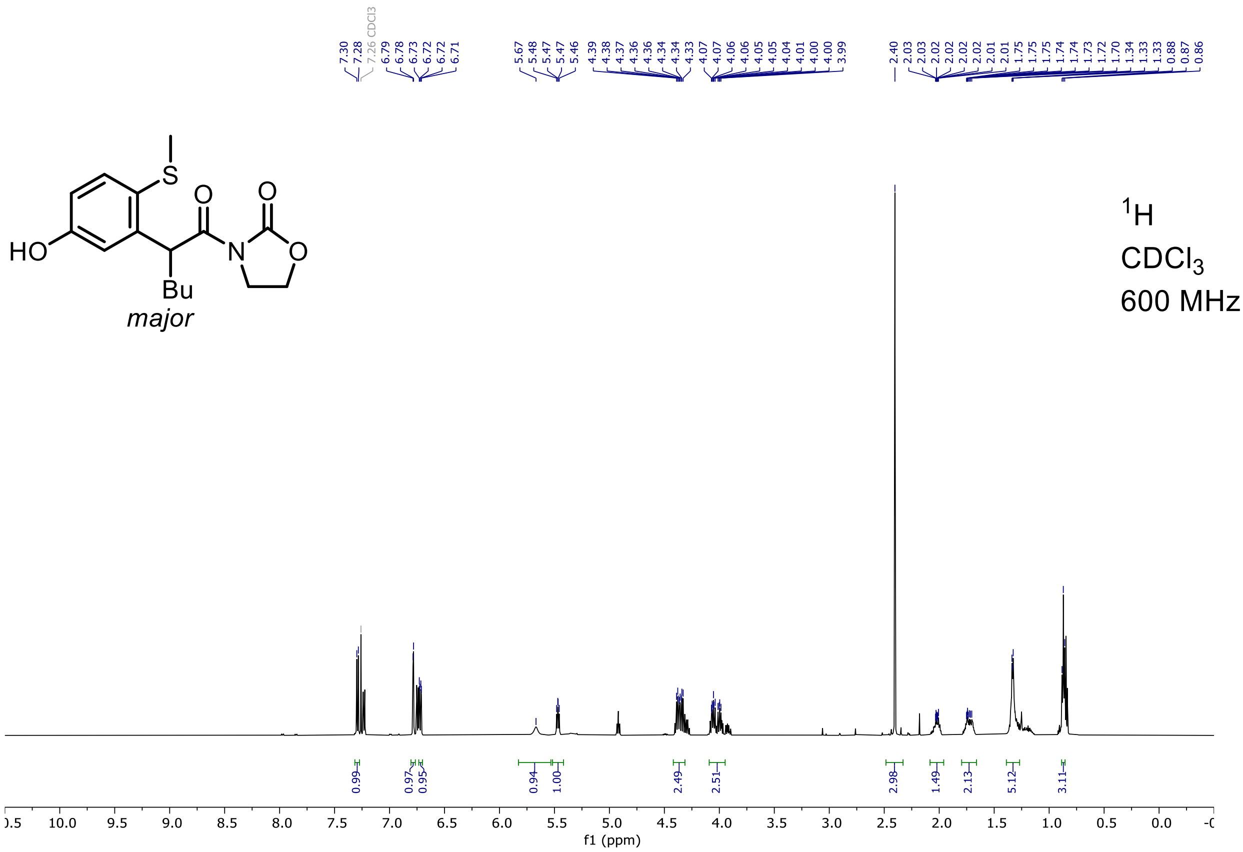

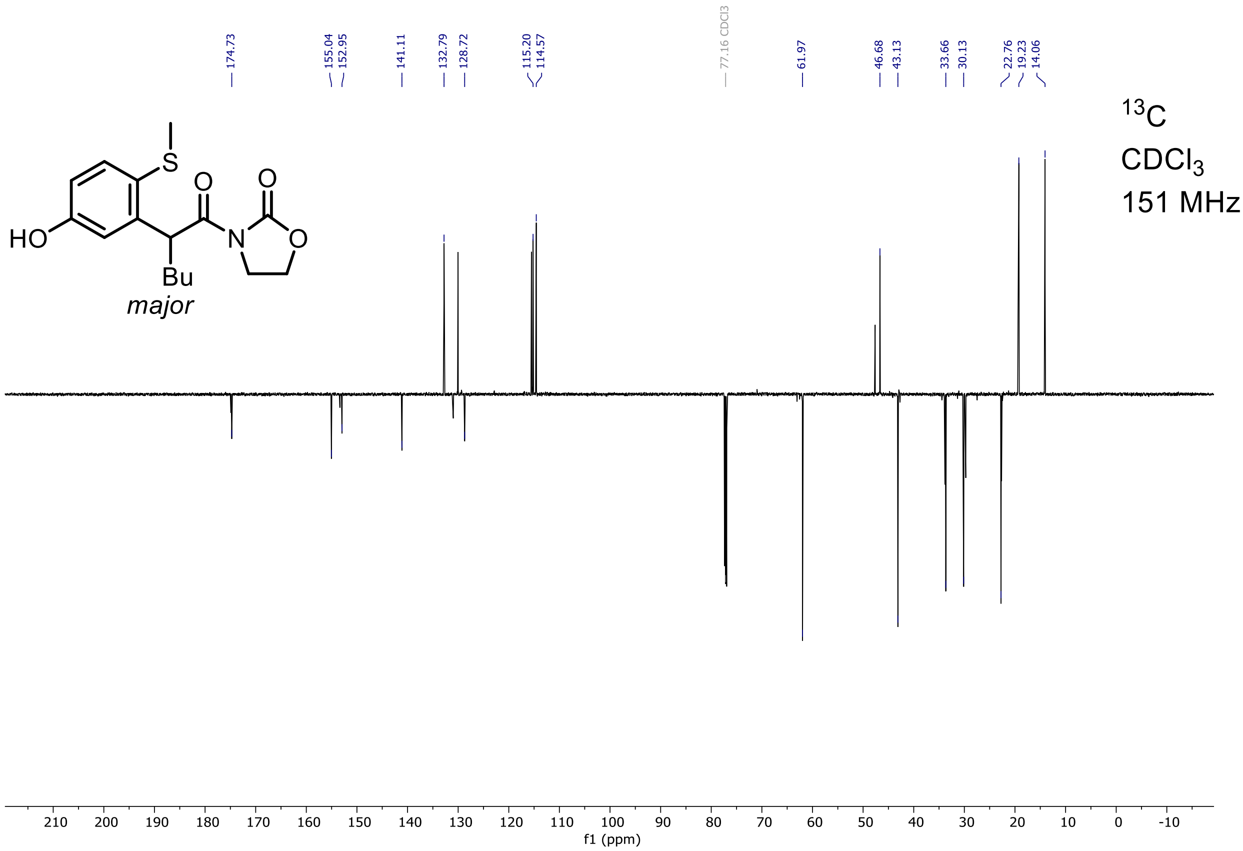

10. HPLC Traces

1a, (+)-1a and (−)-1a – 1,3-Dimethoxy-2-(methylsulfinyl)benzene

1a

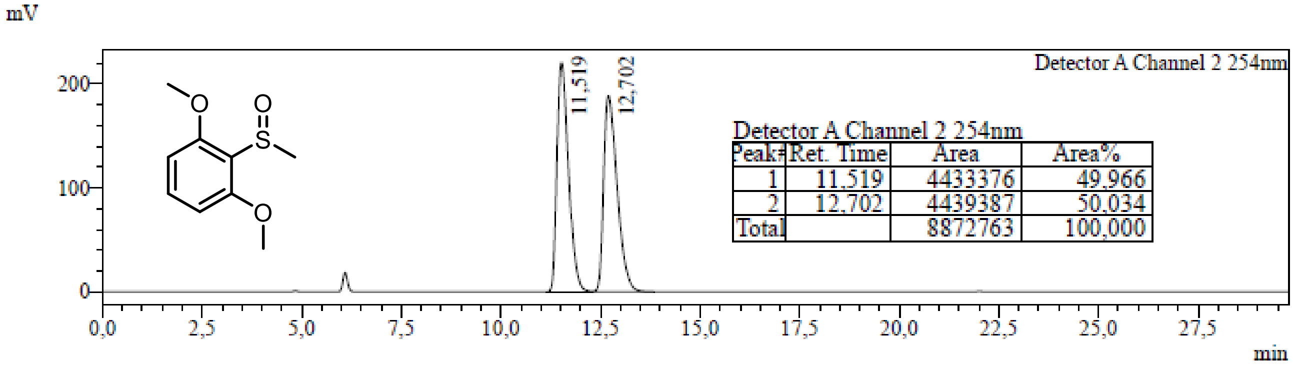

(+)-1a

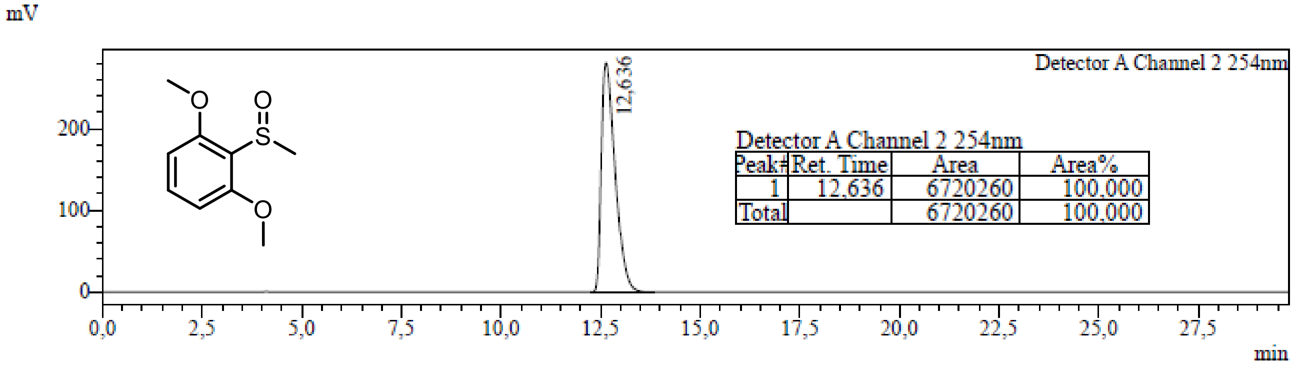

(−)-1a

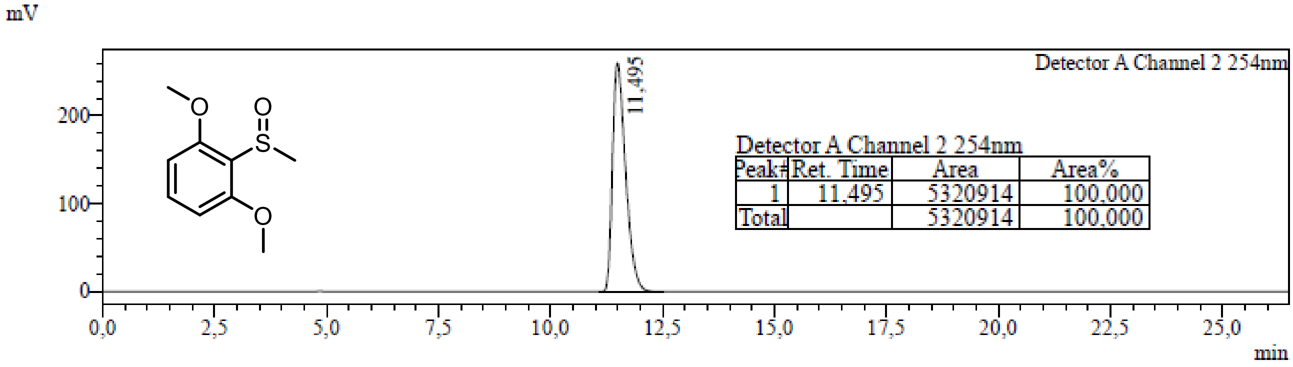

5aA and (−)-5aA – *S*-methyl 2-(2,6-dimethoxyphenyl)hexanethioate

5aA

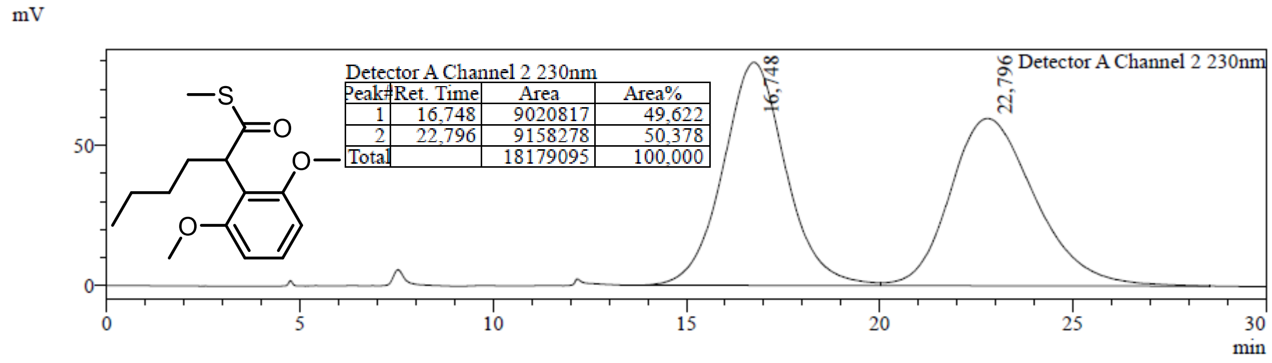

Detector A Channel 2 230nm

Peak#	Ret. Time	Area	Area%
1	16,748	9020817	49,622
2	22,796	9158278	50,378
Total		18179095	100,000

(−)-5aA

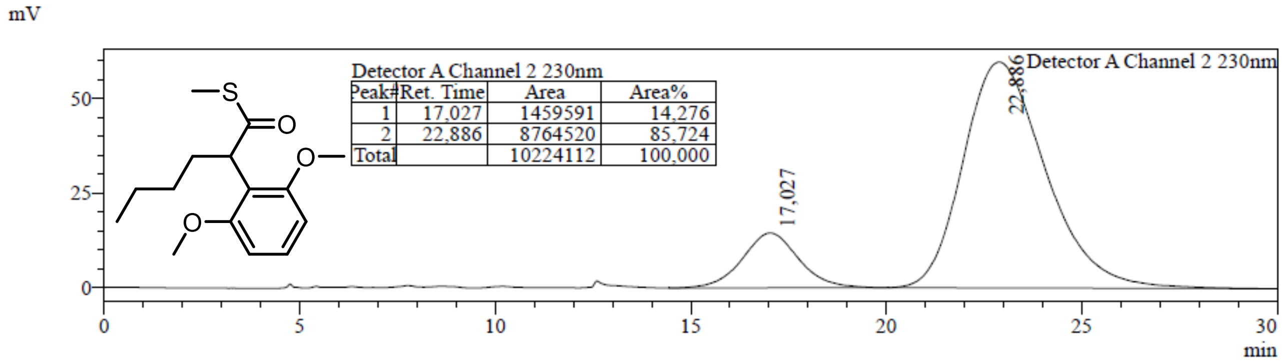

Detector A Channel 2 230nm

Peak#	Ret. Time	Area	Area%
1	17,027	1459591	14,276
2	22,886	8764520	85,724
Total		10224112	100,000

4aD and (+)-4aD – 3-(2-Cyclohexyl-2-(2,6-dimethoxyphenyl)acetyl)oxazolidin-2-one

4aD

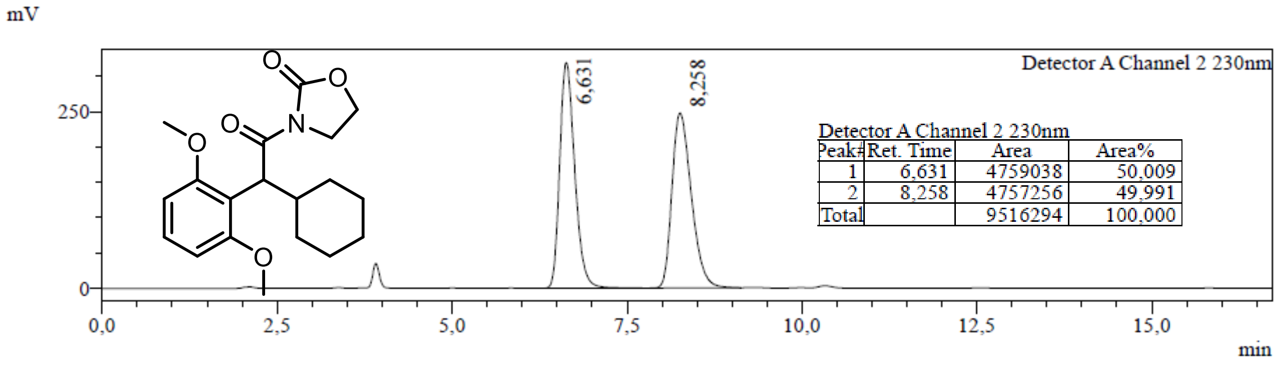

Detector A Channel 2 230nm

Peak#	Ret. Time	Area	Area%
1	6,631	4759038	50,009
2	8,258	4757256	49,991
Total		9516294	100,000

(+)-4aD

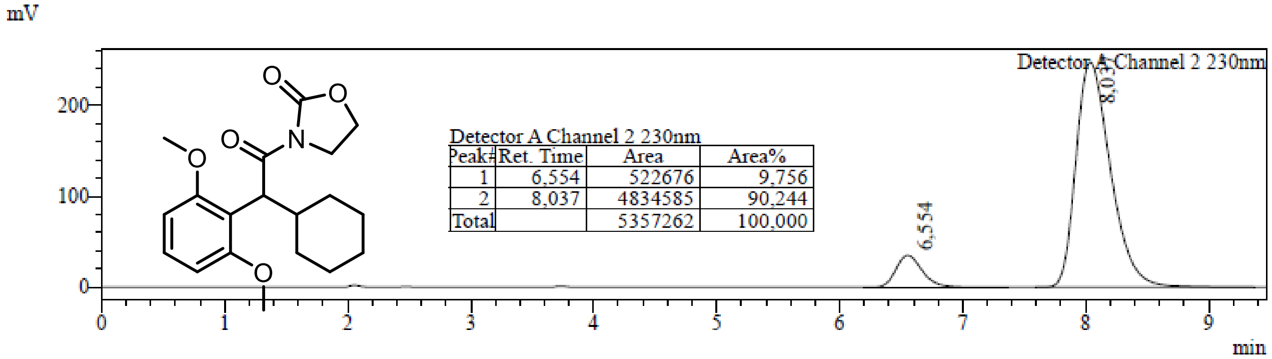

Detector A Channel 2 230nm

Peak#	Ret. Time	Area	Area%
1	6.554	522676	9.756
2	8.037	4834585	90.244
Total		5357262	100.000

11. X-Ray Analysis

The X-ray intensity data was measured on Bruker D8 Venture diffractometer equipped with multilayer monochromator, Mo K/α INCOATEC micro focus sealed tube and Oxford cooling system. The structure was solved by *Direct Methods*. Non-hydrogen atoms were refined with *anisotropic displacement parameters*. Hydrogen atoms were inserted at calculated positions and refined with riding model. The following software was used: *Bruker SAINT software package*[47] using a narrow-frame algorithm for frame integration, *SADABS*[48] for absorption correction, *OLEX2*[49] for structure solution, refinement, molecular diagrams and graphical user-interface, *Shelxle*[50] for refinement and graphical user-interface *SHELXS-2015*[51] for structure solution, *SHELXL-2015*[52] for refinement, *Platon*[53] for symmetry check. Experimental data and CCDC-Codes Experimental data (Available online: http://www.ccdc.cam.ac.uk/conts/retrieving.html) can be found in **Table S 9**. Crystal data, data collection parameters, and structure refinement details are given in **Table S 10**. Asymmetric Unit visualized in **Fig. S 11** gives information about the data quality.

Table S 9. Experimental parameters and CCDC-Code.

Sample	Machine	Source	Temp.	Detector Distance	Time/ Frame	#Frames	Frame width	CCDC
	Bruker		[K]	[mm]	[s]		[°]	
**4gA**	D8	Mo	100	37	30	821	0.8	2190622

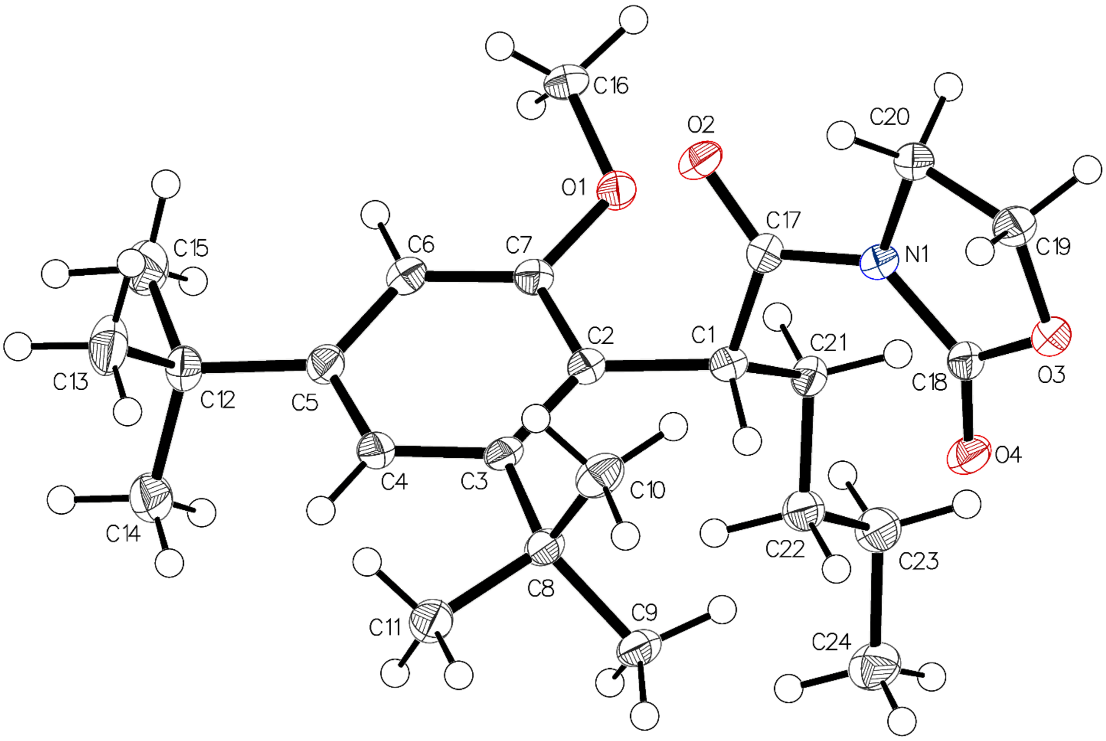

Fig. S 10: X-ray structure of 4gA. Asymmetric Unit of drawn with 50% displacement ellipsoid. The bond precision for C–C single bonds is 0.0026 Å

Table S 10. Sample and crystal data, Data collection and structure refinement.

dentification code	IK_1378_2_P21n
Empirical formula	C24H37NO4
Formula weight	403.54
Temperature/K	100.0
Crystal system	monoclinic
Space group	P21/n
a/Å	14.7184(15)
b/Å	9.8137(11)
c/Å	16.5738(18)

α/°	90
β/°	109.665(4)
γ/°	90
Volume/Å3	2254.3(4)
Z	4
ρcalcg/cm3	1.189
μ/mm-1	0.080
F(000)	880.0
Crystal size/mm3	0.17 × 0.05 × 0.01
Radiation	MoKα (λ = 0.71073)
2Θ range for data collection/°	4.54 to 60.146
Index ranges	-20 ≤ h ≤ 20, -13 ≤ k ≤ 13, -23 ≤ l ≤ 21
Reflections collected	48101
Independent reflections	6596 [Rint = 0.1146, Rsigma = 0.0665]
Data/restraints/parameters	6596/0/270
Goodness-of-fit on F2	1.015
Final R indexes [I>=2σ (I)]	R1 = 0.0578, wR2 = 0.1215
Final R indexes [all data]	R1 = 0.1056, wR2 = 0.1462
Largest diff. peak/hole / e Å-3	0.30/-0.28

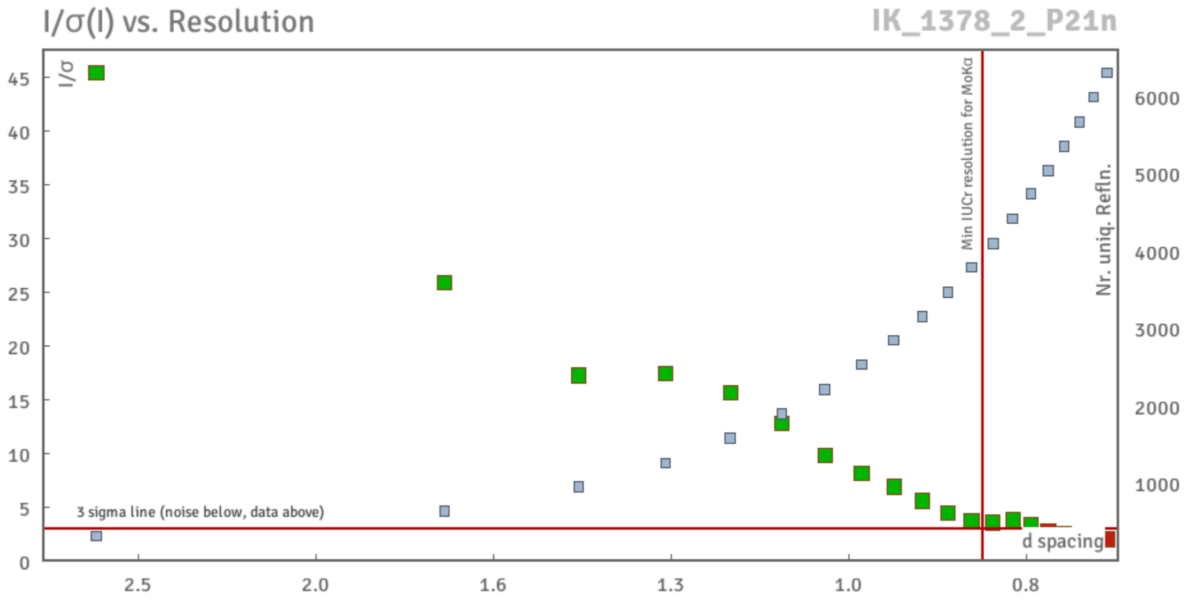

Fig. S 11: Data quality.

12. References

1. Brutiu, B. R.; Klose, I.; Maulide, N. Facile C–S Bond Cleavage of Aryl Sulfoxides Promoted by Brønsted Acid. *Synlett* **2020**, *32*, 488–490.
2. Todorović, U.; Klose, I.; Maulide, N. Straightforward Access to Thiocyanates via Dealkylative Cyanation of Sulfoxides. *Org. Lett.* **2021**, *23*, 2510–2513.
3. Bell, K.; Mccaffery, L. Use of Menthyl 2-Methoxynaphthalene-1-sulfinates in the Andersen Synthesis of Optically Active Sulfoxides. Facile Cleavage by Grignard Reagents of Some Aromatic Methyl Ethers. *Aust. J. Chem.* **1994**, *47*, 1925.
4. Buchanan, G. W.; Reyes-Zamora, C.; Cheung, C. Restricted rotation in hindered aryl methyl sulfoxides as detected by low-temperature proton magnetic resonance. *J. Org. Chem.* **1975**, *40*, 2537–2539.
5. Motsch, S.; Schütz, C.; Huy, P. H. Systematic Evaluation of Sulfoxides as Catalysts in Nucleophilic Substitutions of Alcohols: Systematic Evaluation of Sulfoxides as Catalysts in Nucleophilic Substitutions of Alcohols. *Eur. J. Org. Chem.* **2018**, 4541–4547.
6. Forchetta, M.; Sabuzi, F.; Stella, L.; Conta, V.; Galloni, P. KuQuinone as a Highly Stable and Reusable Organic Photocatalyst in Selective Oxidation of Thioethers to Sulfoxides. *J. Org. Chem.* **2022**, *87*, 14016–14025.
7. Takahashi, F.; Nogi, K.; Yorimitsu, H. B_2cat_2-Mediated Reduction of Sulfoxides to Sulfides. *Eur. J. Org. Chem.* **2020**, *2020*, 3009-3012.
8. Bell, K. Chlorosulfination of Aromatic Methyl Ethers with Thionyl Chloride. *Aust. J. Chem.* **1985**, *38*, 1209.
9. Farah, B. S.; Gilbert, E. E. Alkylmercaptophenols by Sulfenylation of Phenols. *J. Org. Chem.* **1963**, *28*, 2807–2809.
10. Bhonde, V. R.; O'Neill, B. T.; Buchwald, S. L. An Improved System for the Aqueous Lipshutz-Negishi Cross-Coupling of Alkyl Halides with Aryl Electrophiles. *Angew. Chem. Int. Ed.* **2016**, *55*, 1849–1853.
11. Peng, B.; Huang, X.; Xie, L.-G.: Maulide, N. A Brønsted Acid Catalyzed Redox Arylation. *Angew. Chem. Int. Ed.* **2014**, *53*, 8718–8721.
12. Collins, B. S. L.; Suero, M. G.; Gaunt, M. J. Copper-Catalyzed Arylative Meyer-Schuster Rearrangement of Propargylic Alcohols to Complex Enones Using Diaryliodonium Salts. *Angew. Chem. Int. Ed.* **2013**, *52*, 5799–5802.

13. Starkov, P.; Moore, J. T.; Duquette, D. C.; Stoltz, B. M.; Marek, I. Enantioselective Construction of Acyclic Quaternary Carbon Stereocenters: Palladium-Catalyzed Decarboxylative Allylic Alkylation of Fully Substituted Amide Enolates. *J. Am. Chem. Soc.* **2017**, *139*, 9615–9620.
14. Kaldre, D.; Klose, I.; Maulide, N. Stereodivergent synthesis of 1,4-dicarbonyls by traceless charge–accelerated sulfonium rearrangement. *Science* **2018**, *361*, 664–667.
15. Pinto, A.; Kaiser, D.; Maryasin, B.; Di Mauro, G.; González, L.; Maulide, N. Hydrative Aminoxylation of Ynamides: One Reaction, Two Mechanisms. *Chem. Eur. J.* **2018**, *24*, 2515–2519.
16. Mansfield, S. J.; Christensen, K. E.; Thompson, A. L.; Ma, K.; Jones, M. W.; Mekareeya, A.; Anderson, E. A. Copper-Catalyzed Synthesis and Applications of Yndiamides. *Angew. Chem. Int. Ed.* **2017**, *56*, 14428–14432.
17. Martínez-Esperón, M. F.; Rodríguez, D.; Castedo, L.; Saá, C. Coupling and cycloaddition of ynamides: homo- and Negishi coupling of tosylynamides and intramolecular [4+2] cycloaddition of N-(o-ethynyl)phenyl ynamides and arylynamides. *Tetrahedron* **2006**, *62*, 3843–3855.
18. Knochel, P.; Melzig, L.; Stemper, J. A Novel Palladium-Catalyzed Cross-Coupling of Thiomethylated Alkynes with Functionalized Organozinc Reagents. *Synthesis* **2010**, 2085–2091.
19. Xie, L.-G.; Shaaban, S.; Chen, X.; Maulide, N. Metal-Free Synthesis of Highly Substituted Pyridines by Formal [2+2+2] Cycloaddition under Mild Conditions. *Angew. Chem. Int. Ed.* **2016**, *55*, 12864–12867.
20. Bello, D.; O'Hagan, D. Lewis acid-promoted hydrofluorination of alkynyl sulfides to generate α-fluorovinyl thioethers. *Beilstein J. Org. Chem.* **2015**, *11*, 1902–1909.
21. Mauro, G. D.; Drescher, M.; Tkaczyk, S.; Maulide, N. Aminoxylation of Thioalkynes through Radical-Polar Crossover. *Synlett* **2019**, *31*, 592–594.
22. Meng, S.; Wang, Y.; Liu, J.; Zheng, J.; Wang, Q. Tandem Cross-Coupling of Alkynyl Sulfides and Alkynyl Sulfoxides/[3,3]-Sulfonium Rearrangement to Construct Tetrasubstituted Furans. *Org. Lett.* **2022**, *24*, 757–761.
23. Fujiki, K.; Tanifuji, N.; Sasaki, Y.; Yokoyama, T. New and Facile Synthesis of Thiosulfonates from Sulfinate/Disulfide/I2 System. *Synthesis* **2002**, 343–348.
24. Zhang, H.; Riomet, M.; Roller, A.; Maulide, N. Synthesis of Novel Heterocycles by Amide Activation and Umpolung Cyclization. *Org. Lett.* **2020**, *22*, 2376–2380.
25. Hamada, T.; Ye, X.; Stahl, S. S. Copper-Catalyzed Aerobic Oxidative Amidation of Terminal Alkynes: Efficient Synthesis of Ynamides. *J. Am. Chem. Soc.* **2008**, *130*, 833–835.

26. Kawasaki, K.; Masubuchi, M.; Hayase, T.; Komiyama, S.; Watanabe, F.; Fukuda, H.; Murata, T.; Matsubara, Y.; Koyama, K.; Shindoh, H.; Sakamoto, H.; Okamato, K.; Ohta, A.; Katsume, A.; Aoki, M.; Aoki, Y.; Shimma, N.; Sudoh, M.; Tsukuda, T. Enantioselective synthesis of derivatives and structure–activity relationship study in the development of NA255 as a novel host-targeting anti-HCV agent. *Bioorg. Med. Chem. Lett.* **2013**, *23*, 336–339.
27. Smith, J. M.; Qin, T.; Merchant, R. R.; Edwards, J. T.; Malins, L. R.; Liu, Z.; Che, G.; Shen, Z.; Shaw, S. A.; Eastgate, M. D.; Baran, P. S. Decarboxylative Alkynylation. *Angew. Chem. Int. Ed.* **2017**, *56*, 11906–11910.
28. Lempenauer, L.; Duñach, E.; Lemière, G. Tuning the Reactivity of Functionalized Diallylic Alcohols: Brønsted versus Lewis Acid Catalysis. *Chem. Eur. J.* **2017**, *23*, 10285–10288.
29. Kaldre, D.; Maryasin, B.; Kaiser, D.; Gajsek, O.; González, L.; Maulide, N. An Asymmetric Redox Arylation: Chirality Transfer from Sulfur to Carbon through a Sulfonium [3,3]-Sigmatropic Rearrangement. *Angew. Chem. Int. Ed.* **2017**, *56*, 2212–2215.
30. Kaiser, D.; Veiros, L. F.; Maulide, N. Brønsted Acid-Mediated Hydrative Arylation of Unactivated Alkynes. *Chem. Eur. J.* **2016**, *22*, 4727-4732.
31. Spicher, S.; Grimme, S. Robust Atomistic Modeling of Materials, Organometallic, and Biochemical Systems. *Angew. Chem. Int. Ed.* **2020**, *59*, 15665–15673.
32. Grimme, S. Exploration of Chemical Compound, Conformer, and Reaction Space with Meta-Dynamics Simulations Based on Tight-Binding Quantum Chemical Calculations. *J. Chen. Theory Comput.* **2019**, *15*, 2847–2862.
33. Pracht, P.; Bohle, F.; Grimme, S. Automated exploration of the low-energy chemical space with fast quantum chemical methods. *Phys. Chem. Chem. Phys.* **2020**, *22*, 7169–7192.
34. Hättig, C.; Hellweg, A.; Köhn, A. Distributed memory parallel implementation of energies and gradients for second-order Møller–Plesset perturbation theory with the resolution-of-the-identity approximation. *Phys. Chem. Chem. Phys.* **2006**, *8*, 1159.
35. Weigend, F.; Häser, M. RI-MP2: first derivatives and global consistency. *Theor. Chem. Acc.* **1997**, *97*, 331–340.
36. Weigend, F.; Häser, M.; Patzelt, H.; Ahlrichs, R. RI-MP2: optimized auxiliary basis sets and demonstration of efficiency. *Chem. Phys. Lett.* **1998**, *294*, 143–152.
37. Weigend, F.; Ahlrichs, R. Balanced basis sets of split valence, triple zeta valence and quadruple zeta valence quality for H to Rn: Design and assessment of accuracy. *Phys. Chem. Chem. Phys.* **2005**, *7*, 3297.

38. Weigend, F. Accurate Coulomb-fitting basis sets for H to Rn. *Phys. Chem. Chem. Phys.* **2006**, *8*, 1057.

39. Klamt, A.; Schüürmann, G. COSMO: a new approach to dielectric screening in solvents with explicit expressions for the screening energy and its gradient. *J. Chem. Soc., Perkin Trans. 2* **1993**, 799–805.

40. Perdew, J. P.; Burke, K.; Ernzerhof, M. Generalized Gradient Approximation Made Simple. *Phys. Rev. Lett.* **1996**, *77*, 3865–3868.

41. Adamo, C.; Barone, V. Toward reliable density functional methods without adjustable parameters: The PBE0 model. *J. Chem. Phys.* **1999**, *110*, 6158–6170.

42. Marenich, A. V.; Cramer, C. J.; Truhlar, D. G. Universal Solvation Model Based on Solute Electron Density and on a Continuum Model of the Solvent Defined by the Bulk Dielectric Constant and Atomic Surface Tensions. *J. Phys. Chem. B* **2009**, *113* (18), 6378–6396.

43. Barone, V.; Cossi, M. Quantum Calculation of Molecular Energies and Energy Gradients in Solution by a Conductor Solvent Model. *J. Phys. Chem. A* **1998**, *102* (11), 1995–2001.

44. TURBOMOLE V7.2 2017, a development of University of Karlsruhe and Forschungszentrum Karlsruhe GmbH, 1989-2007, TURBOMOLE GmbH, since 2007; available from http://www.turbomole.com.

45. Neese, F. The ORCA Program System. Wiley Interdiscip. *Rev. Comput. Mol. Sci.* **2012**, *2(1)*, 73–78.

46. Frisch, M. J.; Trucks, G. W.; Schlegel, H. B.; Scuseria, G. E.; Robb, M. A.; Cheeseman, J. R.; Scalmani, G.; Barone, V.; Petersson, G. A.; Nakatsuji, H.; Li, X.; Caricato, M.; Marenich, A. V.; Bloino, J.; Janesko, B. G.; Gomperts, R.; Mennucci, B.; Hratchian, H. P.; Ortiz, J. V.; Izmaylov, A. F.; Sonnenberg, J. L.; Williams-Young, D.; Ding, F.; Lipparini, F.; Egidi, F.; Goings, J.; Peng, B.; Petrone, A.; Henderson, T.; Ranasinghe, D.; Zakrzewski, V. G.; Gao, J.; Rega, N.; Zheng, G.; Liang, W.; Hada, M.; Ehara, M.; Toyota, K.; Fukuda, R.; Hasegawa, J.; Ishida, M.; Nakajima, T.; Honda, Y.; Kitao, O.; Nakai, H.; Vreven, T.; Throssell, K.; Montgomery, J. A., Jr.; Peralta, J. E.; Ogliaro, F.; Bearpark, M. J.; Heyd, J. J.; Brothers, E. N.; Kudin, K. N.; Staroverov, V. N.; Keith, T. A.; Kobayashi, R.; Normand, J.; Raghavachari, K.; Rendell, A. P.; Burant, J. C.; Iyengar, S. S.; Tomasi, J.; Cossi, M.; Millam, J. M.; Klene, M.; Adamo, C.; Cammi, R.; Ochterski, J. W.; Martin, R. L.; Morokuma, K.; Farkas, O.; Foresman, J. B.; Fox, D. J. Gaussian, Inc., Wallingford CT, 2016.

47. Bruker SAINT V8.38B Copyright © 2005-2015 Bruker AXS.

48. Sheldrick, G. M. SADABS, University of Göttingen, Germany. at (1996).

49. Dolomanov, O. V., Bourhis, L. J., Gildea, R. J., Howard, J. A. K. & Puschmann, H. OLEX2: a complete structure solution, refinement and analysis program. *J. Appl. Crystallogr.* **2009**, *42*, 339–341.
50. Hübschle, C. B., Sheldrick, G. M. & Dittrich, B. ShelXle: a Qt graphical user interface for SHELXL. *J. Appl. Crystallogr.* **2011**, *44*, 1281–1284.
51. Sheldrick, G. M. SHELXS v 2016/4, University of Göttingen, Germany. at (2015).
52. Sheldrick, G. M. SHELXL v 2016/4, University of Göttingen, Germany. at (2015).
53. Spek, A. L. Structure validation in chemical crystallography. *Acta Crystallogr. D* **2009**, *65*, 148–155.
